# Integrated omics profiling of dextran sodium sulfate-induced colitic mice supplemented with Wolfberry (*Lycium barbarum*)

**DOI:** 10.1038/s41538-020-0065-5

**Published:** 2020-03-31

**Authors:** Wanping Aw, Huijuan Jia, Weida Lyu, Shinji Fukuda, Masaru Tomita, Lila Otani, Hisanori Kato

**Affiliations:** 1 Health Nutrition, Graduate School of Agricultural and Life Sciences, the University of Tokyo, 1-1-1, Yayoi, Bunkyo-Ku, Tokyo 113-8657 Japan; 2 Institute for Advanced Biosciences, Keio University, 246-2 Mizukami, Kakuganji, Tsuruoka, Yamagata 997-0052 Japan; 3 Intestinal Microbiota Project, Kanagawa Institute of Industrial Science and Technology, 3-25-13 Tonomachi, Kawasaki-ku, Kawasaki, Kanagawa 210-0821 Japan; 4 Transborder Medical Research Center, University of Tsukuba, 1-1-1 Tennodai, Tsukuba, Ibaraki 305-8577 Japan; 5 PRESTO, Japan Science and Technology Agency, 4-1-8 Honcho Kawaguchi, Saitama, 332-0012 Japan; 6 Metabologenomics, Inc., 246-2 Mizukami, Kakuganji, Tsuruoka, Yamagata 997-0052 Japan

**Keywords:** Inflammatory bowel disease, Nutrition

## Abstract

We used a multi-omics profiling approach to investigate the suppressive effects of 2% Wolfberry (WOL)-enriched diets on dextran sodium sulfate (DSS)-induced colitis in mice. It was observed that in mice fed the WOL diet, the disease activity index, colon shortening, plasma concentrations of matrix metalloproteinase-3 and relative mesenteric fat weight were significantly improved as compared to the DSS group. Results from colon transcriptome and proteome profiles showed that WOL supplementation significantly ameliorated the expression of genes and proteins associated with the integrity of the colonic mucosal wall and colonic inflammation. Based on the hepatic transcriptome, proteome and metabolome data, genes involved in fatty acid metabolism, proteins involved in inflammation and metabolites related to glycolysis were downregulated in WOL mice, leading to lowered inflammation and changes in these molecules may have led to improvement in body weight loss. The integrated nutrigenomic approach thus revealed the molecular mechanisms underlying the ameliorative effect of whole WOL fruit consumption on inflammatory bowel disease.

## Introduction

Inflammatory bowel disease (IBD) is a group of debilitating inflammatory disorders that affect the gastrointestinal tract. IBD includes Crohn’s disease (CD) and ulcerative colitis (UC). Affected patients experience various symptoms associated with gut inflammation, ranging from abdominal pain and diarrhea to rectal bleeding and weight loss. Physicians usually recommend using anti-inflammatory steroids or immunosuppressants to reduce inflammation^1^. Dietary interventions^2^ using nutraceuticals^3^, probiotics, prebiotics^2^; lifestyle interventions and recently, fecal microbiota transplantation^4^ are commonly used as alternatives to medical therapy in IBD management^5–7^.

Wolfberry (WOL), the fruit of *Lycium barbarum* (member of Solanceae), is a sweet red berry that has been used traditionally as a medicinal food in China and other Asian countries like Vietnam, Japan and Korea^8,9^. WOL comprises of various polyphenols^10^ including phenylpropanoids, coumarins, lignans, flavonoids, isoflavonoids, chlorogenic acid derivatives^10^. It also consists of carotenoids (zeaxanthin and carotene)^11,12^, polysaccharides^13,14^ and small molecules such as betaine^15^, cerebroside^16^, β-sitosterol and various vitamins^17^. Valuable components of WOL include zeaxanthin, a non-provitamin A carotenoid shown to protect the eyes^18^ and to enhance immune response^19,20^. Studies have suggested that WOL consumption may have neuroprotective^21,22^, anti-aging^23^, prevention of Alzheimer’s Disease^22^, and immune-boosting properties^9,24,25^. WOL polysaccharides also have been reported to have anti-diabetic properties^26^, improve retinopathy in diabetic rats^27^ and improve diabetic testicular dysfunction^28^. It also has been documented that WOL polysaccharide fractions can protect against renal damage^29^ and reduce immunotoxicity^30^. It has been previously reported that a milk-based WOL preparation can suppress 2,4,6-trinitrobenzene sulfonic acid (TNBS)-induced colitis^31^ and WOL can suppress dextran sulfate sodium (DSS)-induced colitis^32^.

Although there are many reports on WOL constituents, there are in fact very few studies using whole WOL even though it is often consumed whole in cuisine, dessert or infused in hot water. In this study, we used a nutrigenomics-based multidimensional approach is used to determine the effects of whole WOL nutritional-intervention dietary signals on the status of genes, proteins and metabolites. We sought to identify the dietary signature of WOL interactions in murine hosts and to elucidate potential molecular mechanisms in a DSS-induced colitis mouse model via comprehensive transcriptome, proteome, and metabolome evaluations not only using colon and but also liver tissues, which is an important metabolic powerhouse in the host.

## Results

### General characteristics

**Fig. 1:**
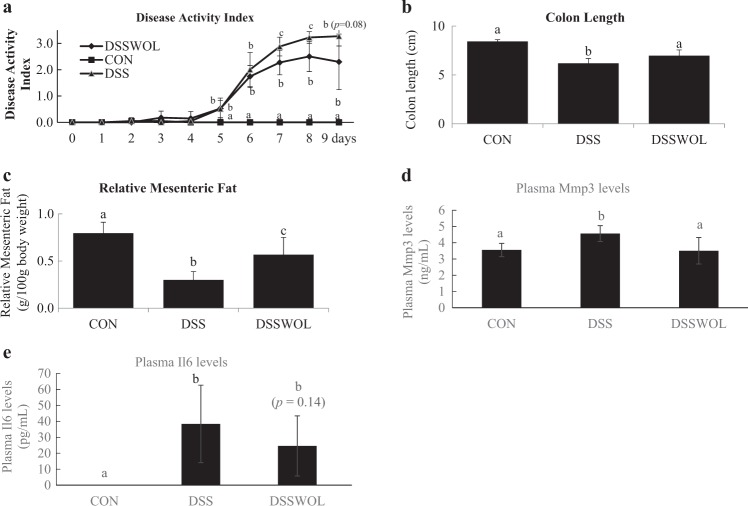
General characteristics. **a** The disease activity index (DAI) was calculated by combining the scores of body weight loss, stool consistency, and fecal blood, and then dividing the score by 3. Each score was determined as follows: change in body weight (0: <1%; 1: 1–5%; 2: 5–10%; 3: 10–15%; 4: >15%), fecal blood (0: no fecal blood observed; 2: ++; 4: +++) and stool consistency (0: normal; 2: soft; 4: diarrhea). **b** Length of colon. **c** Relative mesenteric fat. **d** Plasma Mmp3 levels. **e** Plasma Il6 concentrations. **f** H&E staining of the colon. All values are the means ± SD (*n* = 6–7). Data with different letters (a, b, c) are significantly different at *p* < 0.05 by Dunnett’s test. *p* value in the bracket is vs. DSS.

No significant differences were observed in food intake before DSS (CON: 27.0 ± 2.3; DSS: 27.7 ± 4.7; DSSWOL: 24.8 ± 2.3 g) or after DSS administration (CON: 40.6 ± 9.9; DSS: 37.1 ± 7.9; DSSWOL: 32.3 ± 2.3 g). Water intake during the DSS administration period are as follows: CON: 26.5 ± 0.6; DSS: 24.2 ± 1.5; DSSWOL: 24.8 ± 1.2 mL. The DSS intakes of the DSS and DSSWOL mice were thus not significantly different. The mice that were DSS-challenged presented with significant pathological changes, including severe body weight loss, presence of fecal occult blood, and diarrhea, resulting in a significant increase in the DAI compared to the CON group (Fig. 1a).

WOL supplementation suppressed these pathological conditions of IBD, resulting in a decrease in the DAI values from day 7 to day 9 (Fig. 1a). Mice from the DSS group had significantly shorter colon lengths (Fig. 1b) and lower mesenteric fat weight (Fig. 1c) compared to the control group, and WOL significantly ameliorated these decreases. The plasma Mmp3 (Fig. 1d) levels were significantly increased by DSS administration, and the WOL intervention significantly mitigated these increases. The concentrations of Il6 in the plasma (Fig. 1e) tended to be improved by WOL supplementation.

### Colonic microarray analysis

**Fig. 2:**
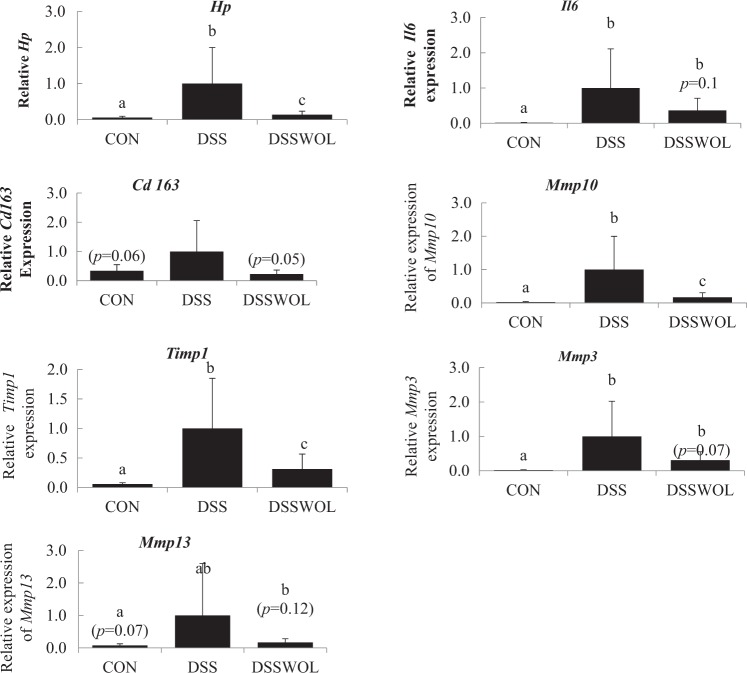
Colonic mRNA relative expressions of genes related to the breakdown of extracellular matrix and tissue remodeling, inflammation and IBD. Mmp3, Mmp10, Mmp13, Timp1, Hp, Il6, and Cd163 were measured by RT-PCR and normalized to Rplp1. All values are the means ± SD (*n* = 6–7). Data with different letters (a, b, c) are significantly different at *p* < 0.05 by Dunnett’s test. *p* value in the bracket is vs. DSS.

Messenger RNAs for 2189 genes were differentially expressed in the DSS mice compared to the controls. Among these genes, 271 genes were upregulated in DSSWOL and 207 genes were downregulated compared to the DSS (Supplementary Table 1). WOL decreased the gene expression levels of tissue inhibitor of metalloproteinase 1 (*Timp1*) and chemokines belonging to the chemokine (C–C motif) ligand (Ccl): *Ccl1* and *Ccl5*. In addition, a lower expression level of proinflammatory cytokine of interleukin 1 receptor type 1 (*Il1r1*) was observed. Other downregulated genes included secreted phosphoprotein 1 (*Spp1*), haptoglobin (*Hp*) and prostaglandin-endoperoxide synthase 2 (*Ptgs2*). Some of the downregulated genes were related to the breakdown of extracellular matrix and remodeling matrix metalloproteinase (MMP), i.e., *Mmp3*, *Mmp13* and *Mmp10*; cluster of differentiation (*Cd163*) and *Il6*, were selected for validation by real-time RT-PCR. DSS significantly upregulated the expressions of *Mmp10*, *Hp*, *Il6*, *Mmp3*, and *Timp1*. WOL supplementation significantly attenuated these increases in *Hp*, *Timp1*, and *Mmp10* and tended to improve these upregulations in *Il6*, *Cd163*, and *Mmp3* (Fig. 2).

### Hepatic microarray analysis

**Fig. 3:**
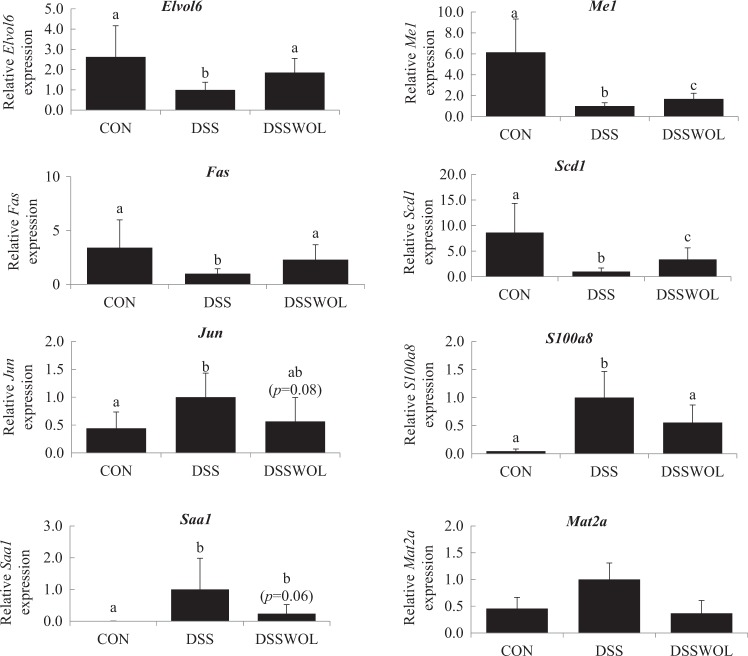
Hepatic mRNA expressions of genes related to fatty acid synthesis and to cancer markers, and genes involved in glycolysis. The relative mRNA expressions of *Elovl6*, *Fas*, *Me1*, *Scd1*, *Jun*, *Saa1*, *Mat2a*, and *S100A8* were measured by RT-PCR and normalized to *Ppia*. All values are the means ± SD (*n* = 6–7). Data with different letters (a, b, c) are significantly different at *p* < 0.05 by Dunnett’s test. *p* value in the bracket is vs. DSS.

In the liver, 12,838 genes were differentially expressed in DSS compared to the controls. In the DSSWOL group, 1630 genes were upregulated and 1793 genes were downregulated compared to the DSS mice (Supplementary Table 2). WOL supplementation decreased the expression of genes for molecules of cancer markers such as serum amyloid A1 (*Saa1*), c-Jun (*Jun*), and S100 calcium-binding protein A8 (*S100a8*). In addition, genes involved in the fatty acid synthesis, i.e., stearoyl-CoA desaturase-1 (*Scd1*), ELOVL family member 6, elongation of long chain fatty acids (*Elovl6*), fatty acid synthase (*Fas*), and NADP-dependent malic enzyme (*Me1*) were upregulated. The gene expression level of methionine adenosyltransferase II, alpha (*Mat2a*), which is involved in the methionine-recycling pathway, was decreased. Based on the results of the validation of gene expression by real-time RT-PCR (Fig. 3), DSS significantly regulated the expression of *Elovl6*, *Fas*, *Jun*, *Mat2*, *Me1*, *Saa1*, *Scd1*, and *S100A8*, and WOL supplementation significantly affected the expression of these genes—except for *Jun* and Saa1, on which WOL tended to have a suppressive effect.

### Comparative hepatic and colonic proteomics analysis by iTRAQ

We determined the global protein expression in the three groups by using iTRAQ labeling quantitative proteomic technology. We included a 1.2- or 0.8-fold cut-off from unique proteins quantified at the >95% confidence level when classifying proteins as highly up- or downregulated.

In the liver, we identified 222 proteins, of which 58 proteins were downregulated and 75 proteins were upregulated in DSSWOL compared to the DSS group (Supplementary Table 3). WOL supplementation decreased the levels of proteins that are known to be commonly upregulated in IBD patients: calreticulin (Calr)^33^, serotransferrin (Trfe)^34^, annexin A5 (Anxa5)^35^, beta-enolase (Enob)^36^, transthyretin (Tthy)^37^, selenium-binding protein 1 (Sbp1)^38^, and protein-glutamine gamma-glutamyltransferase 2 (Tgm2)^39^; proteins involved in fibrosis: fibrinogen beta chain (Fibb) and fibrinogen gamma chain (Fibg)^40^; and inflammation-related proteins: histone H4 (H4), superoxide dismutase [Cu-Zn] (Sodc)^41^, cystatin-B (Cytb)^42^, 10-kDa heat shock protein, mitochondrial (Ch10)^43^, thioredoxin (Thio)^44^, and alpha-enolase (Enoa)^45^.

In the colon, we detected a total of 2,111 proteins, of which 271 were upregulated and 217 were downregulated in the DSSWOL group compared to the DSS group (Supplementary Table 4). WOL supplementation increased the levels of several proteins, including trefoil factor 3 (Tff3), a protein involved in intestinal tight junction integrity and mucus protection^46^; anterior gradient protein 2 homolog (Agr2)^47^; the anti-inflammation-related protein carboxypeptidase E (Cbpe)^48^; and a protein related to DNA repair, 40S ribosomal protein S3 (Rs3)^49,50^.

Compared to the DSS group, the downregulated proteins in the DSSWOL group were: a negative modulator of leukocyte inflammatory responses, tyrosine-protein phosphatase non-receptor type substrate 1 (Shps1)^51^; a marker protein in IBD patients, isoform S-gicerin of cell surface glycoprotein MUC18 (Muc18)^52^; an IBD risk factor protein, apolipoprotein E (Apoe)^53^; neural cell adhesion molecule L1 (L1cam)^54^; a protein related to inflammation and tissue injury resistance, ceruloplasmin (Ceru)^55^; two proteins involved in tumor progression, annexin a3 (Anxa3)^56,57^ and complement C3 (Co3)^58^; and neutrophil gelatinase-associated lipocalin (Ngal)^59^.

### Plasma and hepatic metabolome evaluation

To evaluate metabolic alteration by WOL consumption, we performed liver and plasma metabolome analyses of the CON, DSS, and DSSWOL groups based on their *m/z* values and migration times. The numbers of metabolites detected in the liver tissue and plasma were 131 and 117, respectively. Normalized metabolomics data was used for hierarchical clustering for a heatmap representation and further analyzed by a principal component analysis (PCA) using MeV software.

**Fig. 4:**
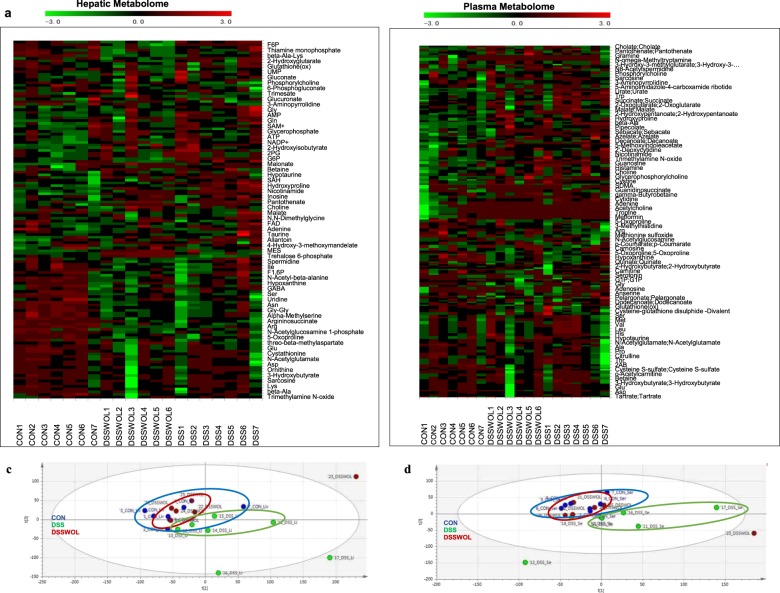
The normalized tabolomics data were hierarchically clustered on both the metabolite and sample axes for a heatmap representation and further analyzed by PCA using MeV and XLSTAT software. No distinct difference among the three groups was observed in the heatmap (**a**, **b**) but distinct clusters were formed among CON, DSS, and DSSWOL in the PCA data analysis (**c**, **d**) of both plasma and liver metabolites.

Although no obvious variations among the groups were observed in the heatmap (Fig. 4a, b), distinct clusters were formed among the CON, DSS, and DSSWOL groups (Fig. 4c, d). Ten metabolites in liver (Supplementary Table 5a) and four metabolites (Supplementary Table 5b) in plasma were significantly regulated by DSS administration and subsequently improved by WOL supplementation. These metabolites included hepatic metabolites involved in the pentose phosphate pathway, i.e., glucose-6-phosphate dehydrogenase (G6P) and fructose 6-phosphate (F6P); and in purine metabolism, i.e., adenosine monophosphate (AMP), inosine monophosphate (IMP) and guanosine monophosphate (GMP). The other metabolites were two molecules related to the methionine-recycling pathway, glutathione and S-lactoylglutathione, and a product of collagen degradation, i.e., hydroxyproline. The upregulation of 2-hydroxyisobutyrate, dodecanoate, and arginine was observed in plasma metabolome profiles.

## Discussion

We comprehensively examined the impact of WOL supplementation on the inflamed colon and liver transcriptome and proteome, as well as the plasma and liver metabolome profiles. Our results revealed that WOL dietary supplementation may ameliorate DSS-induced colonic injury and inflammation and enhance gut barrier defense by maintaining mucosal integrity, as described below. Our findings also revealed modulated metabolic pathways and potential key gene/protein regulatory hubs that may regulate these processes. Our experimental approach of WOL feeding prior to DSS challenge can be regarded as similar to the patterns of the consumption of various food products by IBD patients during a period of fluctuating disease activity^60^. To our knowledge, this is an unprecedented report of the use of pure whole WOL in IBD using a comprehensive nutrigenomics approach, particularly in UC.

Our colon gene expression data revealed that WOL intervention suppresses colonic inflammation. Inflammatory responses in IBD usually begin with an infiltration of neutrophils and macrophages^61^, and thereafter a mixture of inflammatory cytokines, including *IL6*, *IL1* and *TNF*^62^, is secreted. This results in the production of proteases, free radicals, and metalloproteinases which are responsible for colon shortening, tissue degradation, and lesion development^63^. In the present study, the global colon gene expression and RT-PCR validation revealed a tendency of downregulation of *Il6*, and its downstream factors, *Mmp10* and *Hp*, were reduced significantly by WOL intervention. The expression of *Hp* has been associated with inflammation is also significantly reduced in inflammatory conditions^64^. The downregulation of proteins related to inflammation and tissue injury (Ceru and Shps1) and the genes *Hp*, *Mmp10*, *Timp1* in our DSSWOL group suggests that the WOL intervention lowered the extent of inflammation in the colon induced by DSS.

WOL intake preserved the integrity of the mucosal barrier which is an important component of a healthy gut, as it represents the first line of defense between the luminal content and host tissues. A damaged mucosal barrier enables microbial interactions with the colonic mucosa, which can stimulate host innate and inflammatory immune responses. Downregulation of fibrosis-related proteins, i.e., Fibb and Fibg^65^, and genes of metalloproteinases were observed in DSSWOL mice as compared to DSS. The significantly downregulated expression level of *Mmp10* and plasma Mmp3 by WOL intervention could help prevent the degradation of a broad range of extracellular components including proteoglycans, type II, type IV, type IX and type XI collagens, laminin, and fibronectin^63^, thereby inhibiting the degradation of the mucosal wall that is manifested in IBD patients^66^.

WOL supplementation also improved mucosal barrier integrity via modulating the expression levels of the protein Tff3, which is commonly expressed in the gastrointestinal tract and in most of the mucosal membranes^67,68^, is able to maintain the integrity of the tight junctions and maintain intestinal barrier function by protecting the epithelial layer in combination with mucin^69^. This protein is also related to angiogenesis and anti-apoptosis by its combined action with mucins^67^. Intestinal goblet cells secrete not only the MUC2 mucin but also a number of typical mucus components including AGR2, and TFF3^70^. The upregulation of Tff3 in the present DSSWOL group indicates that WOL supplementation improved the integrity of the intestinal barrier function and tight junction that had been degraded by DSS administration. The protein Agr2 secreted by epithelial cells plays a vital function in the synthesis of the intestinal mucin Muc2, which is a type of cysteine-rich glycoprotein that develops the mucus gel lining the intestine. Agr2 deficiency is suggested to lead to susceptibility to IBD^71^. Prior in vivo research also revealed that Agr2 is related to the maintenance of epithelial integrity in a mouse model^47^. In our present investigation, WOL intervention enhanced not only the expression of Agr2 but also the colonic mucosal defense function in DSS-challenged mice.

Despite a relatively short DSS administration period, WOL intervention improved the expression levels of cancer-related proteins in the colon (Anxa3 and Co3) as well as oncogenic hepatic genes (*Saa1*, *Jun* and *S100a8*) as observed in the DSSWOL mice as compared to the DSS group. It has been reported that 2% of IBD cases progress to colorectal cancer (CRC) and account for 15% of CRC-related deaths. Axelrad et al. and Pedersen et al. also reported that in IBD patients, long-term exposure to chronic inflammation is the primary risk factor for CRC development as well as other types of cancers^72,73^. By lowering the expression of these cancer-related markers and proteins commonly expressed in IBD patients (Muc18, Apoe, L1cam), WOL intervention might delay the onset of IBD-related CRC.

WOL intake also improved the metabolic pathways involved in glycolysis. During inflammation, numerous immune cells are activated and migrate to inflammatory lesions. Phagocytosis, bacteria-killing and stimulated cell proliferation have high energy demands^74^. Neutrophils, macrophages, and dendritic cells primarily use glycolytic pathways to obtain energy, whereas B and T cells mainly use amino acids, glucose, and lipids to generate energy during oxidative phosphorylation. Nutrition and oxygen are rapidly limited or depleted in tissues with high inflammatory lesions and elicited immune activities^75^. Hepatic metabolites involved in glycolysis, i.e., G6P and F6P have been significantly upregulated by WOL supplementation, indicating that WOL plays an active role in glycolytic recovery. A significant upregulation of genes involved in fatty acid metabolism (*Fas*, *Me1*, *Scd1*, and *Elovl6*) by WOL supplementation as well as the metabolic shifts in glycolysis could have contributed to the improved body weight loss in the DSSWOL mice induced by DSS administration. This observation was in accord with the lowered expressions of inflammatory proteins in the liver, such as H4, Sodc, and Cytb.

Our data also showed that WOL intervention could prevent the onset of any early event prior to IBD. In most cases, an inhibitory effect of DSS treatment on *SCD1* in the liver has been characterized as an early event prior to IBD clinical symptoms^76^. The disruption of intestinal microflora and the release of endotoxins following DSS treatment may contribute to the acceleration of DSS-induced colitis^76^. Direct downstream targets of endotoxins and bacterial infection include proinflammatory cytokines, such as interleukins and *TNFα*, which can subdue *SCD1* expression. Since we observed that the *Il6* expression levels were dramatically upregulated by DSS treatment, it is likely that a portal vein delivery of inflammatory markers from the colon contributes to the suppression of *Scd1* expression in the liver. In IBD patients, hepatobiliary manifestations are frequently observed as the gastrointestinal tract and the hepatobiliary system is anatomically close, and due to the mesenteric venous drainage ascending via the portal vein into the liver^77^. WOL treatment downregulated the colonic expression of *Il6*, leading to a decrease in cytokines delivered to the liver, thereby resulting in the upregulation of *Scd1* and preventing the onset of an early event prior to IBD clinical symptoms.

**Fig. 5:**
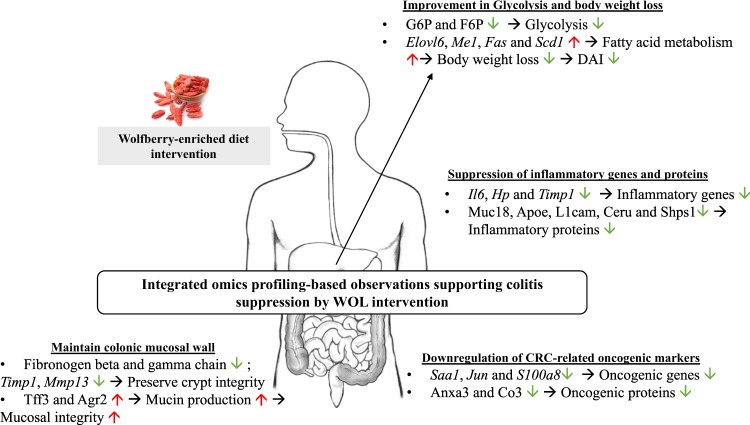
Schematic summary of the integrated omics profiling of WOL intervention in the DSS-colitic mouse model.

As demonstrated above, we employed a nutrigenomics approach to obtain evidence-based insights into the effect of whole WOL supplementation on IBD (Fig. 5). The integrated colon transcriptome and proteome profiles also indicated that 2% WOL supplementation in DSS-challenged mice significantly ameliorated inflammation in the colon, which maintained the integrity of the colonic mucosal wall and might have prevented the risk factors leading to the pathogenesis to CRC. The hepatic transcriptome, proteome, and metabolome data showed that in WOL-fed mice, genes involved in fatty acid metabolism, proteins involved in inflammation, and metabolites related to glycolysis were ameliorated, leading to lowered inflammation and could have contributed to the improvements in body weight. Taken together, our findings provide integrated nutrigenomics information regarding the effect of WOL, a traditional Chinese fruit, on a colitic model. Our results can be expected to be impactful, as the daily inclusion of WOL in a diet could be a dietary strategy to maintain gut health and reduce disease severity in mucosal damage-associated pathologies.

## Methods

### Animals and dietary treatment

Animal care and treatment were approved based on the institutional ethical guidelines at the University of Tokyo and approved by the animal experiment committee of the Faculty of Agriculture, The University of Tokyo (Approval no. P13–739). Seven-week-old male C57BL6/J mice (Charles River Japan, Tokyo) were housed individually in animal cages in a room with controlled temperature (23 ± 2 °C), humidity (50 ± 10%), and a 12-h light (08:00–20:00) and dark (20:00–08:00) cycle throughout the experimental period. After 3 days’ acclimatization, the mice were divided into three groups of 6–7 mice each with equivalent body weights, and provided with the following diets (Supplementary Table 6a) and drinking water: (1) AIN-93G basal diet and tap water for the course of the entire experiment (controls; CON), (2) AIN-93G basal diet and tap water for 1 week, after which colitis was induced by administering tap water containing 1.5% (w/v) DSS (molecular weight, 40 KDa; MP Biomedicals, Irvine, CA) (the DSS group), and (3) 2% WOL-supplemented AIN-93G basal diet throughout the experimental period, along with colitis induced after 1 week in the same manner as for the DSS group (DSSWOL). The pre-DSS period lasted for 7 days and DSS-drinking water was administered during the last 9 days of the experimental period. WOL powder (Natural Life, Inc., Tokyo) was made from grinding dried WOL. The nutritional content in 100 g dried WOL are as follows: 9.7 g protein, 0.4 g fat, 77.1 g carbohydrate, 45.6 g sugar, 13 g dietary fiber, 7.5 g moisture, and 0.8 g ash. The food intake, water intake, and DSS intake of each group were monitored carefully throughout the experiment.

### Evaluation of disease activity index (DAI)

During the DSS challenge, the body weight, stool consistency, and fecal blood were recorded daily as criteria for the DAI^78^. The DAI was calculated by combining these three scores and then dividing the score by 3. Each score was determined as follows: change in body weight (0: <1%; 1: 1–5%; 2: 5–10%; 3: 10–15%; 4: >15%), fecal blood (0: no fecal blood observed, 2: bleeding mixed with normal feces observed, 4: only blood observed) and stool consistency (0: normal, 2: soft, 4: diarrhea).

### Blood collection and tissue harvesting

Mice were sacrificed at the end of the experimental period with sodium pentobarbital followed by bleeding from the carotid artery, and plasma samples were stored at −80 °C after centrifugation (1000 × *g*, 15 min, 4 °C). The colon length (from the ileocecal junction to the proximal rectum) of each mouse was measured and used as a parameter for colitis severity. Excised colon and liver samples as well as mesenteric adipose tissues were snap-frozen in liquid nitrogen and stored at −80 °C until further analysis.

### Biochemical tests

Plasma concentrations of interleukin-(IL)6 and matrix metalloproteinase-3 (MMP3) were measured using enzyme-linked immunosorbent assay (ELISA) kits (Thermo Scientific, Rockford, IL and R&D Systems, Minneapolis, MN, respectively), according to the manufacturer’s instructions.

### Total RNA extraction and transcriptome analysis

Total RNA was extracted from the frozen liver and colon samples. Its concentration was determined from the optical density at 260 nm on a NanoDrop ND-1000 spectrophotometer (Thermo Scientific, Wilmington, DE, USA), and the purity was assessed by determining the A260/A280 ratio^79^. RNA from each respective group was pooled (*n* = 6–7), and colonic and hepatic transcriptome evaluations were carried out. We used Affymetrix GeneChip Command Console^®^ software to analyze the gene expression ratios of the DSS versus CON group data and the DSSWOL versus DSS group data. Array images were analyzed with Microarray Suite ver. 5.0 (MAS5) (Affymetrix). Genes whose expressions changed more than 1.2-fold between treatments were considered to be differentially expressed.

### Real-time reverse transcription polymerase chain reaction (RT-PCR)

To detect and validate the expression of differentially expressed genes related to inflammation and IBD, we carried out an RT-PCR using the real-time PCR detection system (Takara Bio, Madison, WI, USA)^79^. Final mixture (12.5 μL) for RT-PCR consisted of 1 × SYBR Premix Ex Taq Mix (Takara), 0.4 μM of each forward and reverse primers and 1 μL of cDNA. Primers were designed using PRIMER3, and their sequences are as detailed in Supplementary Table 6b. Relative mRNA expressions were normalized to 60S acidic ribosomal protein P1 (*Rplp1*) in the colon and peptidyl-prolyl cis-trans isomerase (*Ppia*) in the liver. Gene expression data are presented as the fold change of normalized mRNA amounts of each sample compared to those from the DSS group.

### Protein preparation, itraq labeling, and nanolc-ms/ms analysis for proteome analysis

Total hepatic and colonic protein were extracted and prepared according to manufacturer’s instructions (AB SCIEX, Framingham, MA). The preparation was then iTRAQ labeled and subjected to nanoscale liquid chromatography coupled to tandem mass spectrometry (nanoLC-MS/MS) analysis. We conducted the protein identification and quantification for iTRAQ samples using ProteinPilot software (ver. 4.0, AB SCIEX) with 95% confidence against switchProt. We used the Paragon Algorithm in the ProteinPilot software for the peptide identification and isoform specific quantification^78^.

### Metabolome analysis

The metabolome profiles were evaluated by extracting metabolites from frozen plasma and liver samples by adding 400 μL of methanol including the internal standards (20 μM each of methionine sulfone and D-camphor-10-sulfonic acid (CSA)) was added to the 40 μL of samples. Next, this mixture was then mixed with 120 μL of ultrapure water and 400 μL of chloroform before centrifuging at 10,000 × *g* for 3 min at 4 °C. Thereafter, proteins and lipids were removed by transferring the aqueous layer to a centrifugal filter tube (UltrafreeMC-PLHCC 250/pk for Metabolome Analysis, Human Metabolome Technologies). The filtrate was centrifugally concentrated and dissolved in 20 μL of ultrapure water that contained reference compounds (200 μM each of 3-aminopyrrolidine and trimesic acid) immediately before CE-TOFMS analysis^80^. All CE-TOFMS experiments were performed using the Agilent CE capillary electrophoresis system (Agilent Technologies). Annotation tables were produced from the measurement of standard compounds that were aligned with the datasets according to similar value and normalized migration time. Peak areas were then normalized against those of the internal standards methionine sulfone or CSA for cationic and anionic metabolites, respectively. The concentrations of each metabolite were calculated based on their relative peak areas and concentrations of standard compounds.

### Statistical analysis

All data relating to general characteristics, biochemical parameters, relative gene expression, and relative metabolite concentrations are presented as the mean ± standard deviation (SD). We analyzed the data by performing a one-way analysis of variance (ANOVA) and Dunnett’s test (BellCurve for Excel,Social Survey Research Information Co., Ltd). Differences were considered statistically significant at *p* < 0.05 compared to the DSS group.

## Supplementary information

Reporting-summary

Supplementary Table 1 Colon transcriptome

Supplementary Table 2 Liver transcriptome

Supplementary Table 3 Differentially regulated proteins in liver

Supplementary Table 4 Differentially expressed proteins in colon

Supplementary Table 5 Metabolome

Supplementary Table 6 PCR Primer Sequences

## Data Availability

The authors declare that [the/all other] data supporting the findings of this study are available within the paper (and its supplementary information files). The data that support the findings of this study are available from the corresponding author upon reasonable request.
