# Supplementary material for: Integrated omics profiling of dextran sodium sulfate-induced colitic mice supplemented with Wolfberry (Lycium barbarum)
Source: NPJ Sci Food. 2020 Mar 31;4:5. doi: 10.1038/s41538-020-0065-5 (PMC7109062; doi:10.1038/s41538-020-0065-5)
Supplement: Supplementary file 2 — Supplementary Table 1 Colon transcriptome [file 41538_2020_65_MOESM2_ESM.docx]

Supplementary Table 1 Differentially Expressed Genes in Colon

271 Up-regulated genes in DSSWOL as compared to DSS

|  | | | Fold Change | Fold Change |
| --- | --- | --- | --- | --- |
| Probe Set ID | Gene Title | Gene Symbol | DSS vs. CON | DSSWOL vs. DSS |
| 1442795_x_at | protease, serine, 37 | *Prss37* | 0.05 | 24.25 |
| 1457140_s_at | Ras association (RalGDS/AF-6) domain family (N-terminal) member 10 | *Rassf10* | 0.08 | 10.56 |
| 1455421_x_at | family with sequence similarity 131, member B | *Fam131b* | 0.04 | 9.85 |
| 1417275_at | myelin and lymphocyte protein, T-cell differentiation protein | *Mal* | 0.19 | 9.85 |
| 1455996_x_at | proline-rich acidic protein 1 | *Prap1* | 0.06 | 9.85 |
| 1421339_at | exostoses (multiple)-like 3 | *Extl3* | 0.27 | 9.19 |
| 1424797_a_at | paired-like homeodomain transcription factor 2 | *Pitx2* | 0.66 | 8.57 |
| 1433553_at | GTPase activating RANGAP domain-like 3 | *Garnl3* | 0.10 | 8.00 |
| 1448394_at | myosin, light polypeptide 2, regulatory, cardiac, slow | *Myl2* | 0.13 | 8.00 |
| 1419582_at | cytochrome P450, family 2, subfamily c, polypeptide 55 | *Cyp2c55* | 0.03 | 7.46 |
| 1425325_at | membrane-spanning 4-domains, subfamily A, member 4B | *Ms4a4b* | 0.14 | 7.46 |
| 1432329_a_at | megakaryocyte-associated tyrosine kinase | *Matk* | 0.12 | 6.96 |
| 1424451_at | acetyl-Coenzyme A acyltransferase 1B | *Acaa1b* | 0.06 | 6.50 |
| 1449416_at | frizzled homolog 4 (Drosophila) | *Fzd4* | 0.27 | 6.50 |
| 1419622_at | UDP glucuronosyltransferase 2 family, polypeptide B5 | *Ugt2b5* | 0.01 | 6.50 |
| 1439569_at | G protein-coupled receptor 83 | *Gpr83* | 0.03 | 6.06 |
| 1418480_at | pro-platelet basic protein | *Ppbp* | 2.83 | 5.66 |
| 1422611_s_at | insulin-like growth factor 2 mRNA binding protein 3 | *Igf2bp3* | 0.62 | 5.28 |
| 1453648_at | sushi domain containing 3 | *Susd3* | 0.87 | 5.28 |
| 1417745_at | carboxypeptidase N, polypeptide 1 | *Cpn1* | 0.13 | 4.29 |
| 1426001_at | eomesodermin homolog (Xenopus laevis) | *Eomes* | 0.76 | 4.29 |
| 1444228_s_at | hect (homologous to the E6-AP (UBE3A) carboxyl terminus) domain and RCC1 (CHC1)-like domain (RLD) 2 | *Herc2* | 0.23 | 4.00 |
| 1429548_at | testis expressed gene 22 | *Tex22* | 0.13 | 4.00 |
| 1427700_x_at | keratin 6A | *Krt6a* | 3.25 | 3.48 |
| 1436615_a_at | ornithine transcarbamylase | *Otc* | 0.09 | 3.25 |
| 1460606_at | hydroxysteroid (17-beta) dehydrogenase 13 | *Hsd17b13* | 0.15 | 3.03 |
| 1426174_s_at | immunoglobulin heavy chain 3 (serum IgG2b) | *Igh-3* | 1.41 | 3.03 |
| 1451481_s_at | organic solute transporter alpha | *Osta* | 0.06 | 3.03 |
| 1451660_a_at | homeobox B6 | *Hoxb6* | 0.16 | 2.83 |
| 1427837_at | Immunoglobulin kappa chain variable 32 (V32) | *Igk-V32* | 0.44 | 2.83 |
| 1432146_at | cation channel, sperm associated 3 | *Catsper3* | 5.66 | 2.64 |
| 1418438_at | fatty acid binding protein 2, intestinal | *Fabp2* | 0.14 | 2.64 |
| 1431077_at | alkaline phosphatase, intestinal | *Alpi* | 0.15 | 2.46 |
| 1460458_at | cysteine-rich secretory protein LCCL domain containing 2 | *Crispld2* | 3.73 | 2.46 |
| 1427184_at | T-cell receptor beta, joining region | *Tcrb-J* | 0.62 | 2.46 |
| 1419498_at | transmembrane and immunoglobulin domain containing 1 | *Tmigd1* | 0.13 | 2.46 |
| 1424266_s_at | carboxylesterase 1F | *Ces1f* | 0.25 | 2.30 |
| 1433600_at | adrenergic receptor, alpha 2a | *Adra2a* | 0.22 | 2.14 |
| 1441569_at | F-box and WD-40 domain protein 21 | *Fbxw21* | 0.76 | 2.14 |
| 1420466_at | mucin-like 1 | *Mucl1* | 1.07 | 2.14 |
| 1422860_at | neurotensin | *Nts* | 0.13 | 2.14 |
| 1435162_at | protein kinase, cGMP-dependent, type II | *Prkg2* | 0.07 | 2.14 |
| 1424556_at | pyrroline-5-carboxylate reductase 1 | *Pycr1* | 0.71 | 2.14 |
| 1437479_x_at | T-box 3 | *Tbx3* | 0.33 | 2.14 |
| 1417920_at | amnionless | *Amn* | 0.22 | 2.00 |
| 1427912_at | carbonyl reductase 3 | *Cbr3* | 1.41 | 2.00 |
| 1453220_at | family with sequence similarity 55, member B | *Fam55b* | 0.18 | 2.00 |
| 1425900_at | hexokinase domain containing 1 | *Hkdc1* | 0.09 | 2.00 |
| 1424631_a_at | Immunoglobulin heavy chain (gamma polypeptide) | *Ighg* | 1.07 | 2.00 |
| 1419167_at | proline-rich acidic protein 1 | *Prap1* | 0.09 | 2.00 |
| 1434188_at | solute carrier family 16 (monocarboxylic acid transporters), member 12 | *Slc16a12* | 0.41 | 2.00 |
| 1423858_a_at | 3-hydroxy-3-methylglutaryl-Coenzyme A synthase 2 | *Hmgcs2* | 0.11 | 1.87 |
| 1452528_a_at | NK2 transcription factor related, locus 3 (Drosophila) | *Nkx2-3* | 0.71 | 1.87 |
| 1429727_at | solute carrier family 16 (monocarboxylic acid transporters), member 9 | *Slc16a9* | 0.25 | 1.87 |
| 1424737_at | thyroid hormone responsive SPOT14 homolog (Rattus) | *Thrsp* | 1.32 | 1.87 |
| 1436245_at | ubiquitin specific peptidase 20 | *Usp20* | 0.62 | 1.87 |
| 1447845_s_at | vanin 1 | *Vnn1* | 0.27 | 1.87 |
| 1416468_at | aldehyde dehydrogenase family 1, subfamily A1 | *Aldh1a1* | 0.20 | 1.74 |
| 1419549_at | arginase, liver | *Arg1* | 2.30 | 1.74 |
| 1449486_at | carboxylesterase 1G | *Ces1g* | 0.12 | 1.74 |
| 1417714_x_at | hemoglobin alpha, adult chain 1 | *Hba-a1* | 2.46 | 1.74 |
| 1418356_at | mercaptopyruvate sulfurtransferase | *Mpst* | 0.57 | 1.74 |
| 1423627_at | NAD(P)H dehydrogenase, quinone 1 | *Nqo1* | 0.71 | 1.74 |
| 1418486_at | vanin 1 | *Vnn1* | 0.22 | 1.74 |
| 1438364_x_at | angiogenin, ribonuclease A family, member 4 | *Ang4* | 0.27 | 1.62 |
| 1438841_s_at | arginase type II | *Arg2* | 0.47 | 1.62 |
| 1427209_at | bromodomain adjacent to zinc finger domain, 2A | *Baz2a* | 0.47 | 1.62 |
| 1448683_at | cytochrome P450, family 2, subfamily d, polypeptide 26 | *Cyp2d26* | 0.18 | 1.62 |
| 1439122_at | DEAD (Asp-Glu-Ala-Asp) box polypeptide 6 | *Ddx6* | 0.50 | 1.62 |
| 1427946_s_at | dihydropyrimidine dehydrogenase | *Dpyd* | 0.13 | 1.62 |
| 1449073_at | filamin C, gamma | *Flnc* | 0.76 | 1.62 |
| 1417184_s_at | hemoglobin, beta adult major chain | *Hbb-b1* | 3.48 | 1.62 |
| 1416473_a_at | immunoglobulin superfamily, DCC subclass, member 4 | *Igdcc4* | 0.23 | 1.62 |
| 1427398_at | mucin 4 | *Muc4* | 0.71 | 1.62 |
| 1426876_at | peptidase M20 domain containing 1 | *Pm20d1* | 0.33 | 1.62 |
| 1425544_at | pleckstrin homology domain containing, family A member 5 | *Plekha5* | 0.50 | 1.62 |
| 1423619_at | RAS, dexamethasone-induced 1 | *Rasd1* | 0.76 | 1.62 |
| 1448290_at | regenerating islet-derived 3 beta | *Reg3b* | 1.07 | 1.62 |
| 1449509_at | small EDRK-rich factor 1 | *Serf1* | 0.27 | 1.62 |
| 1418095_at | small muscle protein, X-linked | *Smpx* | 0.29 | 1.62 |
| 1431188_a_at | target of myb1 homolog (chicken) | *Tom1* | 0.57 | 1.62 |
| 1455913_x_at | transthyretin | *Ttr* | 0.22 | 1.62 |
| 1424880_at | tribbles homolog 1 (Drosophila) | *Trib1* | 0.57 | 1.62 |
| 1440230_at | tsukushin | *Tsku* | 0.31 | 1.62 |
| 1439624_at | UDP glucuronosyltransferase 2 family, polypeptide B35 | *Ugt2b35* | 0.33 | 1.62 |
| 1419136_at | aldo-keto reductase family 1, member C18 | *Akr1c18* | 0.93 | 1.52 |
| 1432344_a_at | amyloid beta (A4) precursor-like protein 2 | *Aplp2* | 0.81 | 1.52 |
| 1455540_at | carbamoyl-phosphate synthetase 1 | *Cps1* | 0.41 | 1.52 |
| 1416193_at | carbonic anhydrase 1 | *Car1* | 0.47 | 1.52 |
| 1454023_a_at | chondroitin polymerizing factor | *Chpf* | 0.71 | 1.52 |
| 1416953_at | connective tissue growth factor | *Ctgf* | 0.41 | 1.52 |
| 1435435_at | cortactin binding protein 2 | *Cttnbp2* | 0.44 | 1.52 |
| 1423844_s_at | cystathionine beta-synthase | *Cbs* | 0.41 | 1.52 |
| 1419674_a_at | dipeptidase 1 (renal) | *Dpep1* | 0.27 | 1.52 |
| 1420998_at | ets variant gene 5 | *Etv5* | 0.93 | 1.52 |
| 1427037_at | eukaryotic translation initiation factor 4, gamma 1 | *Eif4g1* | 0.57 | 1.52 |
| 1440868_at | GA repeat binding protein, beta 2 | *Gabpb2* | 0.54 | 1.52 |
| 1423436_at | glutathione S-transferase, alpha 3 | *Gsta3* | 2.00 | 1.52 |
| 1435465_at | kelch repeat and BTB (POZ) domain containing 11 | *Kbtbd11* | 0.29 | 1.52 |
| 1435741_at | phosphodiesterase 8B | *Pde8b* | 0.31 | 1.52 |
| 1420352_at | protease, serine, 22 | *Prss22* | 2.00 | 1.52 |
| 1451830_a_at | spectrin beta 2 | *Spnb2* | 0.66 | 1.52 |
| 1417616_at | ST6 (alpha-N-acetyl-neuraminyl-2,3-beta-galactosyl-1,3)-N-acetylgalactosaminide alpha-2,6-sialyltransferase 2 | *St6galnac2* | 0.47 | 1.52 |
| 1415823_at | stearoyl-Coenzyme A desaturase 2 | *Scd2* | 0.93 | 1.52 |
| 1427345_a_at | sulfotransferase family 1A, phenol-preferring, member 1 | *Sult1a1* | 0.23 | 1.52 |
| 1416062_at | TBC1 domain family, member 15 | *Tbc1d15* | 0.62 | 1.52 |
| 1421989_s_at | 3'-phosphoadenosine 5'-phosphosulfate synthase 2 | *Papss2* | 0.57 | 1.41 |
| 1439183_at | alkaline ceramidase 1 | *Acer1* | 0.31 | 1.41 |
| 1424959_at | annexin A13 | *Anxa13* | 0.31 | 1.41 |
| 1423910_at | ArfGAP with GTPase domain, ankyrin repeat and PH domain 3 | *Agap3* | 0.76 | 1.41 |
| 1439853_at | beta-1,4-N-acetyl-galactosaminyl transferase 2 | *B4galnt2* | 0.31 | 1.41 |
| 1428485_at | carbonic anyhydrase 12 | *Car12* | 0.50 | 1.41 |
| 1421005_at | centrosomal protein 110 | *Cep110* | 0.66 | 1.41 |
| 1420517_at | chromatin modifying protein 4C | *Chmp4c* | 0.41 | 1.41 |
| 1426731_at | desmin | *Des* | 0.66 | 1.41 |
| 1427945_at | dihydropyrimidine dehydrogenase | *Dpyd* | 0.14 | 1.41 |
| 1423066_at | DNA methyltransferase 3A | *Dnmt3a* | 0.76 | 1.41 |
| 1416661_at | eukaryotic translation initiation factor 3, subunit A | *Eif3a* | 0.71 | 1.41 |
| 1427474_s_at | glutathione S-transferase, mu 3 | *Gstm3* | 0.57 | 1.41 |
| 1437756_at | GTPase, IMAP family member 9 | *Gimap9* | 1.00 | 1.41 |
| 1451229_at | histone deacetylase 11 | *Hdac11* | 0.35 | 1.41 |
| 1431916_at | hydroxy-delta-5-steroid dehydrogenase, 3 beta- and steroid delta-isomerase 3 | *Hsd3b3* | 0.27 | 1.41 |
| 1437650_at | hypothetical protein C730026J16 | *C730026J16* | 0.57 | 1.41 |
| 1451963_at | immunoglobulin heavy chain (J558 family) | *Igh-VJ558* | 1.41 | 1.41 |
| 1424931_s_at | immunoglobulin lambda chain, constant region 1 | *Igl-C1* | 1.00 | 1.41 |
| 1423557_at | interferon gamma receptor 2 | *Ifngr2* | 0.76 | 1.41 |
| 1447252_s_at | meprin 1 alpha | *Mep1a* | 0.23 | 1.41 |
| 1416980_at | methyltransferase like 7B | *Mettl7b* | 0.29 | 1.41 |
| 1450976_at | N-myc downstream regulated gene 1 | *Ndrg1* | 0.54 | 1.41 |
| 1423325_at | pinin | *Pnn* | 0.71 | 1.41 |
| 1417481_at | receptor (calcitonin) activity modifying protein 1 | *Ramp1* | 0.44 | 1.41 |
| 1416041_at | serum/glucocorticoid regulated kinase 1 | *Sgk1* | 0.54 | 1.41 |
| 1417623_at | solute carrier family 12, member 2 | *Slc12a2* | 0.54 | 1.41 |
| 1422786_at | solute carrier family 30 (zinc transporter), member 1 | *Slc30a1* | 1.07 | 1.41 |
| 1418547_at | tissue factor pathway inhibitor 2 | *Tfpi2* | 0.50 | 1.41 |
| 1416308_at | UDP-glucose dehydrogenase | *Ugdh* | 0.38 | 1.41 |
| 1436020_at | zinc finger protein 828 | *Zfp828* | 0.71 | 1.41 |
| 1429415_at | zinc finger, MYND-type containing 8 | *Zmynd8* | 0.62 | 1.41 |
| 1422340_a_at | actin, gamma 2, smooth muscle, enteric | *Actg2* | 0.71 | 1.32 |
| 1449363_at | activating transcription factor 3 | *Atf3* | 0.93 | 1.32 |
| 1422478_a_at | acyl-CoA synthetase short-chain family member 2 | *Acss2* | 0.44 | 1.32 |
| 1432436_a_at | adenylate kinase 3 | *Ak3* | 0.62 | 1.32 |
| 1451260_at | aldehyde dehydrogenase 1 family, member B1 | *Aldh1b1* | 0.47 | 1.32 |
| 1451194_at | aldolase B, fructose-bisphosphate | *Aldob* | 0.54 | 1.32 |
| 1438744_at | ankyrin repeat and SOCS box-containing 7 | *Asb7* | 0.66 | 1.32 |
| 1417889_at | apolipoprotein B mRNA editing enzyme, catalytic polypeptide 2 | *Apobec2* | 0.81 | 1.32 |
| 1422524_at | ATP-binding cassette, sub-family B (MDR/TAP), member 6 | *Abcb6* | 0.54 | 1.32 |
| 1425729_at | bestrophin 2 | *Best2* | 0.47 | 1.32 |
| 1431098_at | CAP-GLY domain containing linker protein 1 | *Clip1* | 0.71 | 1.32 |
| 1448573_a_at | carcinoembryonic antigen-related cell adhesion molecule 10 | *Ceacam10* | 1.52 | 1.32 |
| 1422075_at | caudal type homeobox 2 | *Cdx2* | 0.41 | 1.32 |
| 1456746_a_at | CD99 antigen-like 2 | *Cd99l2* | 0.54 | 1.32 |
| 1424528_at | cell growth regulator with EF hand domain 1 | *Cgref1* | 0.57 | 1.32 |
| 1424529_s_at | cell growth regulator with EF hand domain 1 | *Cgref1* | 0.62 | 1.32 |
| 1455791_at | chromatin licensing and DNA replication factor 1 | *Cdt1* | 0.41 | 1.32 |
| 1418920_at | claudin 15 | *Cldn15* | 0.62 | 1.32 |
| 1423805_at | disabled homolog 2 (Drosophila) | *Dab2* | 1.07 | 1.32 |
| 1441170_a_at | disabled homolog 2 (Drosophila) interacting protein | *Dab2ip* | 0.76 | 1.32 |
| 1417790_at | docking protein 1 | *Dok1* | 0.81 | 1.32 |
| 1417298_at | emopamil binding protein-like | *Ebpl* | 0.87 | 1.32 |
| 1425272_at | epithelial membrane protein 2 | *Emp2* | 0.50 | 1.32 |
| 1444380_at | expressed sequence AI844869 | *AI844869* | 0.76 | 1.32 |
| 1426656_at | family with sequence similarity 63, member A | *Fam63a* | 0.57 | 1.32 |
| 1454883_at | gasdermin C2 | *Gsdmc2* | 0.54 | 1.32 |
| 1448330_at | glutathione S-transferase, mu 1 | *Gstm1* | 0.66 | 1.32 |
| 1443375_at | GNAS (guanine nucleotide binding protein, alpha stimulating) complex locus | *Gnas* | 0.35 | 1.32 |
| 1417399_at | growth arrest specific 6 | *Gas6* | 0.66 | 1.32 |
| 1416905_at | guanylate cyclase activator 2a (guanylin) | *Guca2a* | 0.38 | 1.32 |
| 1423702_at | H1 histone family, member 0 | *H1f0* | 0.76 | 1.32 |
| 1418734_at | histocompatibility 2, Q region locus 2 | *H2-Q2* | 0.33 | 1.32 |
| 1430523_s_at | immunoglobulin lambda chain, variable 1 | *Igl-V1* | 1.41 | 1.32 |
| 1431922_at | intestine specific homeobox | *Isx* | 0.44 | 1.32 |
| 1451255_at | lipolysis stimulated lipoprotein receptor | *Lsr* | 0.66 | 1.32 |
| 1425302_at | major facilitator superfamily domain containing 6-like | *Mfsd6l* | 0.62 | 1.32 |
| 1421835_at | microtubule-associated protein 7 | *Mtap7* | 0.71 | 1.32 |
| 1451176_at | mitochondrial antiviral signaling protein | *Mavs* | 0.87 | 1.32 |
| 1417098_s_at | mitochondrial trans-2-enoyl-CoA reductase | *Mecr* | 0.81 | 1.32 |
| 1426851_a_at | nephroblastoma overexpressed gene | *Nov* | 0.76 | 1.32 |
| 1451678_at | nuclear prelamin A recognition factor | *Narf* | 0.76 | 1.32 |
| 1453187_at | OCIA domain containing 2 | *Ociad2* | 0.57 | 1.32 |
| 1429452_x_at | phosphatidylserine decarboxylase, pseudogene 3 | *Pisd-ps3* | 0.50 | 1.32 |
| 1447707_s_at | phosphodiesterase 2A, cGMP-stimulated | *Pde2a* | 0.54 | 1.32 |
| 1448432_at | phospholipase C, delta 1 | *Plcd1* | 0.62 | 1.32 |
| 1452190_at | prolylcarboxypeptidase (angiotensinase C) | *Prcp* | 0.71 | 1.32 |
| 1427699_a_at | protein tyrosine phosphatase, non-receptor type 11 | *Ptpn11* | 0.71 | 1.32 |
| 1422178_a_at | RAB17, member RAS oncogene family | *Rab17* | 0.47 | 1.32 |
| 1418931_at | regenerating islet-derived family, member 4 | *Reg4* | 0.87 | 1.32 |
| 1422198_a_at | serine hydroxymethyltransferase 1 (soluble) | *Shmt1* | 1.07 | 1.32 |
| 1434159_at | serine/threonine kinase 4 | *Stk4* | 0.71 | 1.32 |
| 1423852_at | shisa homolog 2 (Xenopus laevis) | *Shisa2* | 0.29 | 1.32 |
| 1438824_at | solute carrier family 20, member 1 | *Slc20a1* | 0.41 | 1.32 |
| 1424441_at | solute carrier family 27 (fatty acid transporter), member 4 | *Slc27a4* | 0.54 | 1.32 |
| 1455876_at | solute carrier family 4, sodium bicarbonate cotransporter, member 7 | *Slc4a7* | 0.81 | 1.32 |
| 1450769_s_at | StAR-related lipid transfer (START) domain containing 5 | *Stard5* | 0.76 | 1.32 |
| 1441206_at | synaptopodin 2 | *Synpo2* | 0.57 | 1.32 |
| 1428597_at | TBC1 domain family, member 9B | *Tbc1d9b* | 0.76 | 1.32 |
| 1427232_at | teashirt zinc finger family member 1 | *Tshz1* | 0.57 | 1.32 |
| 1454604_s_at | tetraspanin 12 | *Tspan12* | 0.50 | 1.32 |
| 1425634_a_at | tyrosine kinase, non-receptor, 1 | *Tnk1* | 0.54 | 1.32 |
| 1426261_s_at | UDP glucuronosyltransferase 1 family, polypeptide A1 glucuronosyltransferase 1 family, polypeptide A6A /// UDP glucuronosyltransferase 1 family, polypeptide A6B /// UDP glucuronosyltransferase 1 family, polypeptide A7C /// UDP glucuronosyltransferase 1 family, polypeptide A9 | *Ugt1a1* | 0.41 | 1.32 |
| 1428145_at | acetyl-Coenzyme A acyltransferase 2 (mitochondrial 3-oxoacyl-Coenzyme A thiolase) | *Acaa2* | 0.57 | 1.23 |
| 1426574_a_at | adducin 3 (gamma) | *Add3* | 0.62 | 1.23 |
| 1455454_at | aldo-keto reductase family 1, member C19 | *Akr1c19* | 0.44 | 1.23 |
| 1438937_x_at | angiogenin, ribonuclease, RNase A family, 5 | *Ang* | 0.57 | 1.23 |
| 1425326_at | ATP citrate lyase | *Acly* | 0.87 | 1.23 |
| 1448272_at | B-cell translocation gene 2, anti-proliferative | *Btg2* | 0.57 | 1.23 |
| 1422452_at | BCL2-associated athanogene 3 | *Bag3* | 1.00 | 1.23 |
| 1437210_a_at | bromodomain containing 2 | *Brd2* | 0.76 | 1.23 |
| 1452050_at | calcium/calmodulin-dependent protein kinase ID | *Camk1d* | 0.50 | 1.23 |
| 1427482_a_at | carbonic anhydrase 8 | *Car8* | 0.20 | 1.23 |
| 1460196_at | carbonyl reductase 1 | *Cbr1* | 0.93 | 1.23 |
| 1448182_a_at | CD24a antigen | *Cd24a* | 0.66 | 1.23 |
| 1421187_at | chemokine (C-C motif) receptor 2 | *Ccr2* | 1.23 | 1.23 |
| 1435446_a_at | choline phosphotransferase 1 | *Chpt1* | 0.62 | 1.23 |
| 1454149_a_at | cyclin L2 | *Ccnl2* | 0.62 | 1.23 |
| 1426243_at | cystathionase (cystathionine gamma-lyase) | *Cth* | 0.76 | 1.23 |
| 1432264_x_at | cytochrome c oxidase subunit VIIa polypeptide 2-like | *Cox7a2l* | 0.76 | 1.23 |
| 1423244_at | cytochrome P450, family 2, subfamily c, polypeptide 68 | *Cyp2c68* | 0.41 | 1.23 |
| 1430172_a_at | cytochrome P450, family 4, subfamily f, polypeptide 16 | *Cyp4f16* | 0.71 | 1.23 |
| 1417991_at | deiodinase, iodothyronine, type I | *Dio1* | 0.47 | 1.23 |
| 1460380_at | desmoglein 2 | *Dsg2* | 0.54 | 1.23 |
| 1417382_at | ectonucleoside triphosphate diphosphohydrolase 5 | *Entpd5* | 0.57 | 1.23 |
| 1416529_at | epithelial membrane protein 1 | *Emp1* | 0.54 | 1.23 |
| 1416892_s_at | family with sequence similarity 107, member B | *Fam107b* | 0.57 | 1.23 |
| 1439422_a_at | family with sequence similarity 132, member A | *Fam132a* | 0.66 | 1.23 |
| 1451385_at | family with sequence similarity 162, member A | *Fam162a* | 0.87 | 1.23 |
| 1416411_at | glutathione S-transferase, mu 2 | *Gstm2* | 0.81 | 1.23 |
| 1419330_a_at | glycoprotein A33 (transmembrane) | *Gpa33* | 0.87 | 1.23 |
| 1451619_at | golgi phosphoprotein 3-like | *Golph3l* | 0.76 | 1.23 |
| 1425159_at | golgi transport 1 homolog A (S. cerevisiae) | *Golt1a* | 0.54 | 1.23 |
| 1452318_a_at | heat shock protein 1B | *Hspa1b* | 0.93 | 1.23 |
| 1460180_at | hexosaminidase B | *Hexb* | 0.50 | 1.23 |
| 1425763_x_at | immunoglobulin heavy chain 2 (serum IgA) | *Igh-2* | 1.07 | 1.23 |
| 1433941_at | inositol polyphosphate 5-phosphatase J | *Inpp5j* | 0.76 | 1.23 |
| 1417982_at | insulin induced gene 2 | *Insig2* | 0.81 | 1.23 |
| 1447541_s_at | integrin alpha E, epithelial-associated | *Itgae* | 1.00 | 1.23 |
| 1439765_x_at | keratin 42 | *Krt42* | 0.76 | 1.23 |
| 1438477_a_at | methylmalonyl CoA epimerase | *Mcee* | 0.87 | 1.23 |
| 1448663_s_at | mevalonate (diphospho) decarboxylase | *Mvd* | 1.32 | 1.23 |
| 1437494_at | mitogen-activated protein kinase-activated protein kinase 3 | *Mapkapk3* | 0.81 | 1.23 |
| 1450391_a_at | monoglyceride lipase | *Mgll* | 0.71 | 1.23 |
| 1453263_at | N(alpha)-acetyltransferase 35, NatC auxiliary subunit | *Naa35* | 1.00 | 1.23 |
| 1423974_at | nuclear mitotic apparatus protein 1 | *Numa1* | 0.76 | 1.23 |
| 1419663_at | osteoglycin | *Ogn* | 0.62 | 1.23 |
| 1420493_a_at | phosphate cytidylyltransferase 2, ethanolamine | *Pcyt2* | 1.00 | 1.23 |
| 1448995_at | platelet factor 4 | *Pf4* | 1.87 | 1.23 |
| 1460459_at | progestin and adipoQ receptor family member V | *Paqr5* | 0.57 | 1.23 |
| 1433870_at | proline rich 15-like | *Prr15l* | 0.71 | 1.23 |
| 1450969_at | propionyl Coenzyme A carboxylase, beta polypeptide | *Pccb* | 0.57 | 1.23 |
| 1437453_s_at | proprotein convertase subtilisin/kexin type 9 | *Pcsk9* | 2.00 | 1.23 |
| 1424650_at | protein disulfide isomerase associated 5 | *Pdia5* | 1.00 | 1.23 |
| 1420831_at | quiescin Q6 sulfhydryl oxidase 1 | *Qsox1* | 0.57 | 1.23 |
| 1444409_at | rabphilin 3A-like (without C2 domains) | *Rph3al* | 0.62 | 1.23 |
| 1419814_s_at | S100 calcium binding protein A1 | *S100a1* | 1.00 | 1.23 |
| 1416191_at | Sec61 alpha 1 subunit (S. cerevisiae) | *Sec61a1* | 0.93 | 1.23 |
| 1434776_at | sema domain, seven thrombospondin repeats (type 1 and type 1-like), transmembrane domain (TM) and short cytoplasmic domain, (semaphorin) 5A | *Sema5a* | 0.47 | 1.23 |
| 1452885_at | serine/arginine-rich splicing factor 2, interacting protein | *Srsf2ip* | 0.71 | 1.23 |
| 1426587_a_at | signal transducer and activator of transcription 3 | *Stat3* | 1.23 | 1.23 |
| 1434473_at | solute carrier family 16 (monocarboxylic acid transporters), member 5 | *Slc16a5* | 0.62 | 1.23 |
| 1420503_at | solute carrier family 6 (neurotransmitter transporter), member 14 | *Slc6a14* | 0.71 | 1.23 |
| 1448973_at | sulfotransferase family 1D, member 1 | *Sult1d1* | 0.44 | 1.23 |
| 1415943_at | syndecan 1 | *Sdc1* | 0.81 | 1.23 |
| 1455618_x_at | tetraspanin 33 | *Tspan33* | 0.50 | 1.23 |
| 1424486_a_at | thioredoxin reductase 1 | *Txnrd1* | 0.93 | 1.23 |
| 1431827_a_at | tousled-like kinase 2 (Arabidopsis) | *Tlk2* | 0.66 | 1.23 |
| 1417034_at | trafficking protein particle complex 6A | *Trappc6a* | 0.87 | 1.23 |
| 1425546_a_at | transferrin | *Trf* | 1.74 | 1.23 |
| 1460377_a_at | transmembrane protein 8 (five membrane-spanning domains) | *Tmem8* | 0.87 | 1.23 |
| 1426227_s_at | vacuolar protein sorting 37C (yeast) | *Vps37c* | 1.15 | 1.23 |
| 1436746_at | WNK lysine deficient protein kinase 1 | *Wnk1* | 0.81 | 1.23 |
| 1428687_at | zinc finger protein 687 | *Zfp687* | 0.66 | 1.23 |
