# Supplementary material for: Integrated omics profiling of dextran sodium sulfate-induced colitic mice supplemented with Wolfberry (Lycium barbarum)
Source: NPJ Sci Food. 2020 Mar 31;4:5. doi: 10.1038/s41538-020-0065-5 (PMC7109062; doi:10.1038/s41538-020-0065-5)
Supplement: Supplementary file 3 — Supplementary Table 2 Liver transcriptome [file 41538_2020_65_MOESM3_ESM.docx]

Supplementary Table 2 Differentially Expressed Genes in Liver

1630 Up-regulated genes in DSSWOL as compared to DSS

|  |  |  | Fold Change | Fold Change |
| --- | --- | --- | --- | --- |
| Probe Set ID | Gene Title | Gene Symbol | DSS vs. CON | DSSWOL vs. DSS |
| 1422230_s_at | cytochrome P450, family 2, subfamily a, polypeptide 4 | *Cyp2a4* | 0.07 | 5.81 |
| 1417880_at | glucose-6-phosphatase, catalytic | *G6pc* | 0.10 | 4.15 |
| 1429740_at | tetratricopeptide repeat domain 7 | *Ttc7* | 0.36 | 3.98 |
| 1443838_x_at | fatty acid desaturase 2 | *Fads2* | 0.30 | 3.44 |
| 1454803_a_at | histone deacetylase 11 | *Hdac11* | 0.69 | 3.35 |
| 1422973_a_at | thyroid hormone responsive SPOT14 homolog (Rattus) | *Thrsp* | 0.12 | 3.34 |
| 1419468_at | C-type lectin domain family 14, member a | *Clec14a* | 0.33 | 3.27 |
| 1426037_a_at | regulator of G-protein signaling 16 | *Rgs16* | 0.17 | 3.23 |
| 1420405_at | solute carrier organic anion transporter family, member 1a4 | *Slco1a4* | 0.46 | 3.20 |
| 1442349_at | aminoglycoside phosphotransferase domain containing 1 | *Agphd1* | 0.40 | 3.02 |
| 1425627_x_at | glutathione S-transferase, mu 1 | *Gstm1* | 0.39 | 3.01 |
| 1436643_x_at | hepcidin antimicrobial peptide 2 | *Hamp2* | 0.06 | 3.00 |
| 1426651_at | mitochondrial ribosomal protein L44 | *Mrpl44* | 0.34 | 2.99 |
| 1416416_x_at | glutathione S-transferase, mu 1 | *Gstm1* | 0.34 | 2.99 |
| 1424759_at | arrestin domain containing 4 | *Arrdc4* | 0.54 | 2.93 |
| 1455265_a_at | regulator of G-protein signaling 16 | *Rgs16* | 0.19 | 2.85 |
| 1416368_at | glutathione S-transferase, alpha 4 | *Gsta4* | 0.23 | 2.76 |
| 1415965_at | stearoyl-Coenzyme A desaturase 1 | *Scd1* | 0.20 | 2.73 |
| 1435371_x_at | carboxylesterase 1D | *Ces1d* | 0.25 | 2.72 |
| 1436454_x_at | flap structure specific endonuclease 1 | *Fen1* | 0.40 | 2.70 |
| 1431232_a_at | MAX gene associated | *Mga* | 0.37 | 2.70 |
| 1421411_at | proline-serine-threonine phosphatase-interacting protein 2 | *Pstpip2* | 0.34 | 2.70 |
| 1433899_x_at | TSC22 domain family, member 1 | *Tsc22d1* | 0.23 | 2.69 |
| 1419213_at | N-acetyltransferase 6 | *Nat6* | 0.35 | 2.68 |
| 1423828_at | fatty acid synthase | *Fasn* | 0.25 | 2.66 |
| 1425201_a_at | hydroxypyruvate isomerase homolog (E. coli) | *Hyi* | 0.34 | 2.66 |
| 1426594_at | FERM domain containing 4B | *Frmd4b* | 0.37 | 2.62 |
| 1433646_at | mitochondrial ribosomal protein S27 | *Mrps27* | 0.43 | 2.60 |
| 1439443_x_at | transketolase | *Tkt* | 0.36 | 2.59 |
| 1453840_at | poly(A) binding protein, cytoplasmic 1 | *Pabpc1* | 0.58 | 2.58 |
| 1441727_s_at | zinc finger protein 467 | *Zfp467* | 0.44 | 2.58 |
| 1426726_at | protein phosphatase 1, regulatory subunit 10 pseudogene | *Ppp1r10* | 0.62 | 2.57 |
| 1455282_x_at | aminolevulinic acid synthase 1 | *Alas1* | 0.53 | 2.57 |
| 1448330_at | glutathione S-transferase, mu 1 | *Gstm1* | 0.49 | 2.56 |
| 1449439_at | Kruppel-like factor 7 (ubiquitous) | *Klf7* | 0.63 | 2.55 |
| 1424737_at | thyroid hormone responsive SPOT14 homolog (Rattus) | *Thrsp* | 0.23 | 2.55 |
| 1450970_at | glutamate oxaloacetate transaminase 1, soluble | *Got1* | 0.25 | 2.55 |
| 1419377_at | mediator of RNA polymerase II transcription, subunit 9 homolog (yeast) | *Med9* | 0.59 | 2.55 |
| 1416283_at | phosphoribosylglycinamide formyltransferase | *Gart* | 0.51 | 2.53 |
| 1424834_s_at | inositol 1,4,5-triphosphate receptor 2 | *Itpr2* | 0.50 | 2.52 |
| 1448582_at | catenin, beta like 1 | *Ctnnbl1* | 0.34 | 2.50 |
| 1449375_at | carboxylesterase 2A | *Ces2a* | 0.23 | 2.49 |
| 1422198_a_at | serine hydroxymethyltransferase 1 (soluble) | *Shmt1* | 0.28 | 2.48 |
| 1423797_at | acetoacetyl-CoA synthetase | *Aacs* | 0.27 | 2.44 |
| 1417883_at | glutathione S-transferase, theta 2 | *Gstt2* | 0.35 | 2.43 |
| 1455401_at | calcium/calmodulin-dependent protein kinase kinase 2, beta | *Camkk2* | 0.32 | 2.41 |
| 1452432_at | tissue factor pathway inhibitor | *Tfpi* | 0.62 | 2.41 |
| 1439360_x_at | non-SMC condensin II complex, subunit H2 | *Ncaph2* | 0.59 | 2.38 |
| 1451167_at | coiled-coil domain containing 101 | *Ccdc101* | 0.54 | 2.38 |
| 1426059_at | glucokinase regulatory protein | *Gckr* | 0.41 | 2.36 |
| 1426959_at | 3-hydroxybutyrate dehydrogenase, type 1 | *Bdh1* | 0.50 | 2.35 |
| 1428656_at | ribonuclease III, nuclear | *Rnasen* | 0.55 | 2.34 |
| 1417254_at | spermatogenesis associated 5 | *Spata5* | 0.51 | 2.34 |
| 1429267_at | acyl-CoA thioesterase 11 | *Acot11* | 0.36 | 2.34 |
| 1418033_s_at | zinc finger with KRAB and SCAN domains 6 | *Zkscan6* | 0.64 | 2.33 |
| 1423764_s_at | mitochondrial ribosomal protein L37 | *Mrpl37* | 0.53 | 2.32 |
| 1420722_at | elongation of very long chain fatty acids (FEN1/Elo2, SUR4/Elo3, yeast)-like 3 | *Elovl3* | 0.10 | 2.32 |
| 1420123_at | T-cell leukemia translocation altered gene | *Tcta* | 0.43 | 2.31 |
| 1447816_x_at | oxidoreductase NAD-binding domain containing 1 | *Oxnad1* | 0.39 | 2.31 |
| 1427137_at | carboxylesterase 2E | *Ces2e* | 0.26 | 2.30 |
| 1417823_at | glycine C-acetyltransferase (2-amino-3-ketobutyrate-coenzyme A ligase) | *Gcat* | 0.43 | 2.30 |
| 1450722_at | nucleoporin 50 | *Nup50* | 0.49 | 2.29 |
| 1451150_at | zinc finger protein 410 | *Zfp410* | 0.63 | 2.29 |
| 1456037_x_at | prolactin regulatory element binding | *Preb* | 0.59 | 2.29 |
| 1416127_a_at | aspartyl aminopeptidase | *Dnpep* | 0.52 | 2.28 |
| 1419031_at | fatty acid desaturase 2 | *Fads2* | 0.48 | 2.28 |
| 1434796_at | vesicle-associated membrane protein 4 | *Vamp4* | 0.53 | 2.27 |
| 1422072_a_at | glutathione S-transferase, mu 6 | *Gstm6* | 0.24 | 2.27 |
| 1436374_x_at | F11 receptor | *F11r* | 0.39 | 2.27 |
| 1419582_at | cytochrome P450, family 2, subfamily c, polypeptide 55 | *Cyp2c55* | 0.31 | 2.27 |
| 1418739_at | serum/glucocorticoid regulated kinase 2 | *Sgk2* | 0.32 | 2.26 |
| 1448478_at | mediator complex subunit 20 | *Med20* | 0.59 | 2.25 |
| 1424140_at | galactose-4-epimerase, UDP | *Gale* | 0.48 | 2.24 |
| 1438890_at | Lysine (K)-specific demethylase 2A | *Kdm2a* | 0.61 | 2.23 |
| 1454699_at | sestrin 1 | *Sesn1* | 0.52 | 2.23 |
| 1445729_at | Proteasome (prosome, macropain) 26S subunit, non-ATPase, 13 | *Psmd13* | 0.36 | 2.22 |
| 1450725_s_at | carbonic anhydrase 14 | *Car14* | 0.32 | 2.22 |
| 1453724_a_at | serine (or cysteine) peptidase inhibitor, clade F, member 1 | *Serpinf1* | 0.32 | 2.21 |
| 1418652_at | transmembrane protein 100 | *Tmem100* | 0.33 | 2.20 |
| 1424377_at | ribosomal L24 domain containing 1 | *Rsl24d1* | 0.50 | 2.19 |
| 1421386_at | ankyrin repeat domain 6 | *Ankrd6* | 0.47 | 2.19 |
| 1435787_at | protein phosphatase 1 (formerly 2C)-like | *Ppm1l* | 0.43 | 2.19 |
| 1444640_at | phosphatidylinositol glycan anchor biosynthesis, class G | *Pigg* | 0.36 | 2.18 |
| 1451780_at | B-cell linker | *Blnk* | 0.59 | 2.16 |
| 1438559_x_at | solute carrier family 44, member 2 | *Slc44a2* | 0.42 | 2.15 |
| 1430088_at | zinc finger protein 619 | *Zfp619* | 0.49 | 2.15 |
| 1443466_s_at | polymerase (RNA) III (DNA directed) polypeptide B | *Polr3b* | 0.54 | 2.15 |
| 1448385_at | solute carrier family 15, member 4 | *Slc15a4* | 0.51 | 2.13 |
| 1451103_at | HAUS augmin-like complex, subunit 4 | *Haus4* | 0.45 | 2.13 |
| 1417828_at | aquaporin 8 | *Aqp8* | 0.24 | 2.10 |
| 1429298_at | dimethylarginine dimethylaminohydrolase 1 | *Ddah1* | 0.27 | 2.10 |
| 1417404_at | ELOVL family member 6, elongation of long chain fatty acids (yeast) | *Elovl6* | 0.23 | 2.09 |
| 1421051_s_at | vacuolar protein sorting 25 (yeast) | *Vps25* | 0.55 | 2.09 |
| 1422500_at | isocitrate dehydrogenase 3 (NAD+) alpha | *Idh3a* | 0.61 | 2.09 |
| 1451716_at | v-maf musculoaponeurotic fibrosarcoma oncogene family, protein B (avian) | *Mafb* | 0.24 | 2.08 |
| 1460445_at | serine/arginine-rich splicing factor 2, interacting protein | *Srsf2ip* | 0.74 | 2.07 |
| 1438938_x_at | prohibitin 2 | *Phb2* | 0.75 | 2.06 |
| 1451460_a_at | solute carrier family 22 (organic anion transporter), member 7 | *Slc22a7* | 0.31 | 2.05 |
| 1456213_x_at | glutaminyl-tRNA synthetase | *Qars* | 0.46 | 2.05 |
| 1455400_at | dimethylarginine dimethylaminohydrolase 1 | *Ddah1* | 0.29 | 2.05 |
| 1417260_at | U2 small nuclear ribonucleoprotein auxiliary factor (U2AF) 2 | *U2af2* | 0.37 | 2.04 |
| 1455745_at | ceroid-lipofuscinosis, neuronal 8 | *Cln8* | 0.46 | 2.04 |
| 1453836_a_at | monoglyceride lipase | *Mgll* | 0.34 | 2.04 |
| 1419101_at | transcriptional regulator, SIN3A (yeast) | *Sin3a* | 0.63 | 2.04 |
| 1424451_at | acetyl-Coenzyme A acyltransferase 1B | *Acaa1b* | 0.18 | 2.03 |
| 1416685_s_at | fibrillarin | *Fbl* | 0.46 | 2.03 |
| 1448830_at | dual specificity phosphatase 1 | *Dusp1* | 0.34 | 2.02 |
| 1418040_at | transmembrane protein 186 | *Tmem186* | 0.48 | 2.02 |
| 1452000_s_at | seryl-aminoacyl-tRNA synthetase | *Sars* | 0.60 | 2.02 |
| 1451731_at | ATP-binding cassette, sub-family A (ABC1), member 3 | *Abca3* | 0.60 | 2.01 |
| 1452021_a_at | hairy and enhancer of split 6 (Drosophila) | *Hes6* | 0.32 | 2.01 |
| 1416833_at | kidney expressed gene 1 | *Keg1* | 0.27 | 2.01 |
| 1424238_at | sirtuin 7 (silent mating type information regulation 2, homolog) 7 (S. cerevisiae) | *Sirt7* | 0.53 | 2.00 |
| 1437715_x_at | apurinic/apyrimidinic endonuclease 1 | *Apex1* | 0.71 | 2.00 |
| 1424399_at | uridine-cytidine kinase 1 | *Uck1* | 0.44 | 2.00 |
| 1416387_at | phosphatidylinositol-5-phosphate 4-kinase, type II, gamma | *Pip4k2c* | 0.68 | 2.00 |
| 1418645_at | histidine ammonia lyase | *Hal* | 0.31 | 2.00 |
| 1456466_x_at | ataxin 10 | *Atxn10* | 0.71 | 2.00 |
| 1432099_a_at | proline dehydrogenase (oxidase) 2 | *Prodh2* | 0.61 | 1.98 |
| 1419185_a_at | MLX interacting protein-like | *Mlxipl* | 0.52 | 1.98 |
| 1455042_at | transducin (beta)-like 1 X-linked | *Tbl1x* | 0.38 | 1.97 |
| 1424259_at | lipase maturation factor 1 | *Lmf1* | 0.69 | 1.97 |
| 1436765_at | par-3 (partitioning defective 3) homolog (C. elegans) | *Pard3* | 0.48 | 1.97 |
| 1426023_a_at | rabaptin, RAB GTPase binding effector protein 1 | *Rabep1* | 0.56 | 1.97 |
| 1437171_x_at | gelsolin | *Gsn* | 0.62 | 1.96 |
| 1460256_at | carbonic anhydrase 3 | *Car3* | 0.10 | 1.96 |
| 1460352_s_at | phosphatidylinositol 3 kinase, regulatory subunit, polypeptide 4, p150 | *Pik3r4* | 0.49 | 1.96 |
| 1424744_at | serine dehydratase | *Sds* | 0.45 | 1.96 |
| 1448350_at | argininosuccinate lyase | *Asl* | 0.59 | 1.96 |
| 1435103_x_at | phenylalanyl-tRNA synthetase, beta subunit | *Farsb* | 0.60 | 1.95 |
| 1448275_at | transmembrane protein 19 | *Tmem19* | 0.50 | 1.95 |
| 1447752_x_at | developmentally regulated GTP binding protein 1 | *Drg1* | 0.70 | 1.95 |
| 1448629_at | Hermansky-Pudlak syndrome 4 homolog (human) | *Hps4* | 0.72 | 1.95 |
| 1438711_at | pyruvate kinase liver and red blood cell | *Pklr* | 0.23 | 1.95 |
| 1426952_at | Rho GTPase activating protein 18 | *Arhgap18* | 0.56 | 1.95 |
| 1423907_a_at | NADH dehydrogenase (ubiquinone) Fe-S protein 8 | *Ndufs8* | 0.67 | 1.95 |
| 1415975_at | calcium regulated heat stable protein 1 | *Carhsp1* | 0.39 | 1.94 |
| 1437906_x_at | thioredoxin-like 1 | *Txnl1* | 0.28 | 1.94 |
| 1427897_s_at | suppressor of defective silencing 3 homolog (S. cerevisiae) | *Suds3* | 0.75 | 1.94 |
| 1451253_at | PX domain containing serine/threonine kinase | *Pxk* | 0.52 | 1.94 |
| 1418288_at | lipin 1 | *Lpin1* | 0.38 | 1.94 |
| 1416418_at | gamma-aminobutyric acid (GABA) A receptor-associated protein-like 1 | *Gabarapl1* | 0.54 | 1.93 |
| 1416335_at | macrophage migration inhibitory factor | *Mif* | 0.51 | 1.93 |
| 1448212_at | proteasome (prosome, macropain) assembly chaperone 2 | *Psmg2* | 0.63 | 1.93 |
| 1448302_at | potassium channel tetramerisation domain containing 20 | *Kctd20* | 0.75 | 1.92 |
| 1448811_at | mitochondrial ribosomal protein L2 | *Mrpl2* | 0.52 | 1.92 |
| 1449202_at | sema domain, immunoglobulin domain (Ig), transmembrane domain (TM) and short cytoplasmic domain, (semaphorin) 4G | *Sema4g* | 0.42 | 1.92 |
| 1428728_at | DEAD (Asp-Glu-Ala-Asp) box polypeptide 51 | *Ddx51* | 0.42 | 1.92 |
| 1420541_at | retinol dehydrogenase 16 | *Rdh16* | 0.32 | 1.92 |
| 1419994_s_at | anaphase promoting complex subunit 16 | *Anapc16* | 0.52 | 1.91 |
| 1433677_at | splicing factor, suppressor of white-apricot homolog (Drosophila) | *Sfswap* | 0.47 | 1.90 |
| 1416582_a_at | BCL2-associated agonist of cell death | *Bad* | 0.65 | 1.90 |
| 1449325_at | fatty acid desaturase 2 | *Fads2* | 0.46 | 1.89 |
| 1434987_at | aldehyde dehydrogenase 2, mitochondrial | *Aldh2* | 0.53 | 1.89 |
| 1422438_at | epoxide hydrolase 1, microsomal | *Ephx1* | 0.41 | 1.89 |
| 1460669_at | interleukin enhancer binding factor 3 | *Ilf3* | 0.51 | 1.89 |
| 1433719_at | solute carrier family 9 (sodium/hydrogen exchanger), member 9 | *Slc9a9* | 0.72 | 1.89 |
| 1448110_at | sema domain, immunoglobulin domain (Ig), transmembrane domain (TM) and short cytoplasmic domain, (semaphorin) 4A | *Sema4a* | 0.79 | 1.89 |
| 1422807_at | ADP-ribosylation factor 5 | *Arf5* | 0.65 | 1.88 |
| 1452896_at | gene trap locus 3 | *Gtl3* | 0.47 | 1.88 |
| 1423710_at | dihydrolipoamide S-succinyltransferase (E2 component of 2-oxo-glutarate complex) | *Dlst* | 0.46 | 1.88 |
| 1423664_at | quinoid dihydropteridine reductase | *Qdpr* | 0.44 | 1.88 |
| 1423692_at | NADH dehydrogenase (ubiquinone) 1 alpha subcomplex, 8 | *Ndufa8* | 0.54 | 1.88 |
| 1419670_at | formiminotransferase cyclodeaminase | *Ftcd* | 0.49 | 1.87 |
| 1418841_s_at | cyclin-dependent kinase 11B | *Cdk11b* | 0.57 | 1.87 |
| 1437462_x_at | matrix metallopeptidase 15 | *Mmp15* | 0.43 | 1.86 |
| 1416946_a_at | acetyl-Coenzyme A acyltransferase 1A | *Acaa1a* | 0.16 | 1.86 |
| 1427816_at | serine/arginine-rich splicing factor 2 | *Srsf2* | 0.76 | 1.86 |
| 1418186_at | glutathione S-transferase, theta 1 | *Gstt1* | 0.37 | 1.85 |
| 1448506_at | serine (or cysteine) peptidase inhibitor, clade A, member 6 | *Serpina6* | 0.57 | 1.85 |
| 1419687_at | MACRO domain containing 1 | *Macrod1* | 0.35 | 1.84 |
| 1420654_a_at | glucan (1,4-alpha-), branching enzyme 1 | *Gbe1* | 0.41 | 1.84 |
| 1423709_s_at | phenylalanyl-tRNA synthetase, beta subunit | *Farsb* | 0.50 | 1.84 |
| 1426313_at | brain and reproductive organ-expressed protein | *Bre* | 0.65 | 1.84 |
| 1416569_at | actin-like 6A | *Actl6a* | 0.69 | 1.83 |
| 1426814_at | SEC16 homolog A (S. cerevisiae) | *Sec16a* | 0.57 | 1.83 |
| 1423418_at | farnesyl diphosphate synthetase | *Fdps* | 0.75 | 1.83 |
| 1428164_at | nudix (nucleoside diphosphate linked moiety X)-type motif 9 | *Nudt9* | 0.51 | 1.82 |
| 1454046_x_at | phosphatidylglycerophosphate synthase 1 | *Pgs1* | 0.78 | 1.82 |
| 1449062_at | ketohexokinase | *Khk* | 0.49 | 1.82 |
| 1425706_a_at | damage specific DNA binding protein 2 | *Ddb2* | 0.41 | 1.82 |
| 1451434_s_at | G patch domain containing 8 | *Gpatch8* | 0.36 | 1.81 |
| 1438315_x_at | aldo-keto reductase family 7, member A5 (aflatoxin aldehyde reductase) | *Akr7a5* | 0.37 | 1.81 |
| 1452034_at | prolyl endopeptidase-like | *Prepl* | 0.73 | 1.81 |
| 1424669_at | zinc finger, FYVE domain containing 21 | *Zfyve21* | 0.59 | 1.81 |
| 1454971_x_at | TSC22 domain family, member 1 | *Tsc22d1* | 0.19 | 1.81 |
| 1433518_at | leucine carboxyl methyltransferase 2 | *Lcmt2* | 0.55 | 1.81 |
| 1434544_at | bolA-like 2 (E. coli) | *Bola2* | 0.70 | 1.81 |
| 1431811_a_at | F-box protein 34 | *Fbxo34* | 0.36 | 1.81 |
| 1431147_at | RAD50 interactor 1 | *Rint1* | 0.65 | 1.80 |
| 1435245_at | glutaminase 2 (liver, mitochondrial) | *Gls2* | 0.38 | 1.80 |
| 1416789_at | isocitrate dehydrogenase 3 (NAD+), gamma | *Idh3g* | 0.42 | 1.80 |
| 1438839_a_at | tyrosine 3-monooxygenase/tryptophan 5-monooxygenase activation protein, epsilon polypeptide | *Ywhae* | 0.63 | 1.80 |
| 1456974_at | one cut domain, family member 1 | *Onecut1* | 0.38 | 1.79 |
| 1423743_at | archain 1 | *Arcn1* | 0.67 | 1.79 |
| 1433520_at | SREBF chaperone | *Scap* | 0.44 | 1.79 |
| 1437902_s_at | retinoic acid receptor responder (tazarotene induced) 2 | *Rarres2* | 0.46 | 1.79 |
| 1416452_at | ornithine aminotransferase | *Oat* | 0.29 | 1.79 |
| 1419316_s_at | guanine nucleotide binding protein (G protein), beta polypeptide 1-like | *Gnb1l* | 0.54 | 1.79 |
| 1417146_at | methylthioribose-1-phosphate isomerase homolog (S. cerevisiae) | *Mri1* | 0.69 | 1.79 |
| 1420183_at | loricrin | *Lor* | 0.63 | 1.79 |
| 1423283_at | phosphatidylinositol transfer protein, alpha | *Pitpna* | 0.68 | 1.78 |
| 1434305_at | transmembrane protein 149 | *Tmem149* | 0.73 | 1.78 |
| 1447805_s_at | SLU7 splicing factor homolog (S. cerevisiae) | *Slu7* | 0.52 | 1.78 |
| 1437193_s_at | small nuclear ribonucleoprotein B | *Snrpb* | 0.72 | 1.78 |
| 1416239_at | argininosuccinate synthetase 1 | *Ass1* | 0.38 | 1.77 |
| 1426981_at | proprotein convertase subtilisin/kexin type 6 | *Pcsk6* | 0.49 | 1.77 |
| 1418528_a_at | defender against cell death 1 | *Dad1* | 0.72 | 1.77 |
| 1419461_at | ribonuclease P 14 subunit (human) | *Rpp14* | 0.50 | 1.77 |
| 1428882_at | WD repeat domain, phosphoinositide interacting 2 | *Wipi2* | 0.45 | 1.77 |
| 1415780_a_at | armadillo repeat containing, X-linked 2 | *Armcx2* | 0.70 | 1.77 |
| 1419559_at | cytochrome P450, family 4, subfamily f, polypeptide 14 | *Cyp4f14* | 0.47 | 1.77 |
| 1416849_at | CDGSH iron sulfur domain 1 | *Cisd1* | 0.47 | 1.77 |
| 1428857_at | Sin3A associated protein | *Sap130* | 0.79 | 1.77 |
| 1420997_a_at | glucose phosphate isomerase 1 | *Gpi1* | 0.51 | 1.77 |
| 1425127_at | hydroxy-delta-5-steroid dehydrogenase, 3 beta- and steroid delta-isomerase 2 | *Hsd3b2* | 0.38 | 1.76 |
| 1418013_at | camello-like 1 | *Cml1* | 0.52 | 1.76 |
| 1427982_s_at | synaptic nuclear envelope 2 | *Syne2* | 0.36 | 1.76 |
| 1426546_at | testis-specific kinase 2 | *Tesk2* | 0.75 | 1.76 |
| 1450839_at | DNA segment, human D4S114 | *D0H4S114* | 0.44 | 1.76 |
| 1439406_x_at | phenylalanine-tRNA synthetase 2 (mitochondrial) | *Fars2* | 0.65 | 1.76 |
| 1433991_x_at | diazepam binding inhibitor | *Dbi* | 0.67 | 1.76 |
| 1460706_s_at | RER1 retention in endoplasmic reticulum 1 homolog (S. cerevisiae) | *Rer1* | 0.61 | 1.76 |
| 1416647_at | branched chain ketoacid dehydrogenase E1, alpha polypeptide | *Bckdha* | 0.59 | 1.75 |
| 1426502_s_at | glutamic pyruvic transaminase, soluble | *Gpt* | 0.57 | 1.75 |
| 1416327_at | ubiquitin-fold modifier conjugating enzyme 1 | *Ufc1* | 0.74 | 1.75 |
| 1434299_x_at | intraflagellar transport 27 homolog (Chlamydomonas) | *Ift27* | 0.66 | 1.75 |
| 1448262_at | proteasome (prosome, macropain) subunit, beta type 2 | *Psmb2* | 0.56 | 1.75 |
| 1428090_at | pentatricopeptide repeat domain 3 | *Ptcd3* | 0.52 | 1.75 |
| 1424979_at | anterior pharynx defective 1a homolog (C. elegans) | *Aph1a* | 0.80 | 1.75 |
| 1416411_at | glutathione S-transferase, mu 2 | *Gstm2* | 0.47 | 1.75 |
| 1434790_a_at | leukotriene A4 hydrolase | *Lta4h* | 0.63 | 1.75 |
| 1448324_at | ribonucleic acid binding protein S1 | *Rnps1* | 0.70 | 1.75 |
| 1424147_at | AHA1, activator of heat shock protein ATPase homolog 1 (yeast) | *Ahsa1* | 0.67 | 1.74 |
| 1447961_s_at | mitochondrial ribosomal protein L38 | *Mrpl38* | 0.57 | 1.74 |
| 1417167_at | exosome component 5 | *Exosc5* | 0.65 | 1.74 |
| 1417221_at | protein phosphatase 1A, magnesium dependent, alpha isoform | *Ppm1a* | 0.36 | 1.74 |
| 1425419_a_at | v-raf-leukemia viral oncogene 1 | *Raf1* | 0.49 | 1.74 |
| 1417904_at | DNA cross-link repair 1A, PSO2 homolog (S. cerevisiae) | *Dclre1a* | 0.58 | 1.74 |
| 1418352_at | hydroxysteroid (17-beta) dehydrogenase 2 | *Hsd17b2* | 0.51 | 1.74 |
| 1459913_at | tumor necrosis factor (ligand) superfamily, member 10 | *Tnfsf10* | 0.55 | 1.74 |
| 1417566_at | abhydrolase domain containing 5 | *Abhd5* | 0.53 | 1.74 |
| 1427127_x_at | heat shock protein 1B | *Hspa1b* | 0.48 | 1.74 |
| 1424527_at | protein phosphatase 2, regulatory subunit B, delta isoform | *Ppp2r2d* | 0.63 | 1.74 |
| 1435975_at | DENN/MADD domain containing 4A | *Dennd4a* | 0.44 | 1.74 |
| 1454758_a_at | TSC22 domain family, member 1 | *Tsc22d1* | 0.19 | 1.74 |
| 1416364_at | heat shock protein 90 alpha (cytosolic), class B member 1 | *Hsp90ab1* | 0.72 | 1.74 |
| 1448538_a_at | DNA segment, Chr 4, Wayne State University 53, expressed | *D4Wsu53e* | 0.53 | 1.73 |
| 1426516_a_at | lipin 1 | *Lpin1* | 0.30 | 1.73 |
| 1449419_at | dedicator of cytokinesis 8 | *Dock8* | 0.36 | 1.73 |
| 1435081_at | synaptophysin-like protein | *Sypl* | 0.60 | 1.73 |
| 1423687_a_at | mannosidase, alpha, class 2C, member 1 | *Man2c1* | 0.55 | 1.73 |
| 1434586_a_at | phosphatidylserine synthase 2 | *Ptdss2* | 0.55 | 1.72 |
| 1426690_a_at | sterol regulatory element binding transcription factor 1 | *Srebf1* | 0.58 | 1.72 |
| 1415996_at | thioredoxin interacting protein | *Txnip* | 0.46 | 1.72 |
| 1444518_at | Acyl-Coenzyme A oxidase 1, palmitoyl | *Acox1* | 0.33 | 1.72 |
| 1438769_a_at | thymocyte nuclear protein 1 | *Thyn1* | 0.48 | 1.72 |
| 1430307_a_at | malic enzyme 1, NADP(+)-dependent, cytosolic | *Me1* | 0.11 | 1.72 |
| 1424176_a_at | annexin A4 | *Anxa4* | 0.76 | 1.72 |
| 1417644_at | sarcospan | *Sspn* | 0.55 | 1.72 |
| 1454630_at | sterile alpha motif domain-containing protein 14-like | *Samd14* | 0.32 | 1.72 |
| 1424469_a_at | cleavage and polyadenylation specific factor 4 | *Cpsf4* | 0.59 | 1.71 |
| 1419584_at | tetratricopeptide repeat domain 28 | *Ttc28* | 0.66 | 1.71 |
| 1451166_a_at | coiled-coil domain containing 101 | *Ccdc101* | 0.59 | 1.71 |
| 1424835_at | glutathione S-transferase, mu 4 | *Gstm4* | 0.72 | 1.71 |
| 1416116_at | origin recognition complex, subunit 3 | *Orc3* | 0.63 | 1.71 |
| 1419404_s_at | seven in absentia 1A | *Siah1a* | 0.56 | 1.71 |
| 1423439_at | phosphoenolpyruvate carboxykinase 1, cytosolic | *Pck1* | 0.56 | 1.71 |
| 1449983_a_at | NAD(P)H dehydrogenase, quinone 2 | *Nqo2* | 0.44 | 1.71 |
| 1449025_at | interferon-induced protein with tetratricopeptide repeats 3 | *Ifit3* | 0.57 | 1.71 |
| 1441056_at | ubiquitin specific peptidase 3 | *Usp3* | 0.64 | 1.71 |
| 1451104_a_at | small nuclear ribonucleoprotein 70 (U1) | *Snrnp70* | 0.65 | 1.71 |
| 1423815_at | DEAD (Asp-Glu-Ala-Asp) box polypeptide 56 | *Ddx56* | 0.75 | 1.70 |
| 1417745_at | carboxypeptidase N, polypeptide 1 | *Cpn1* | 0.77 | 1.70 |
| 1434962_x_at | chemokine (C-C motif) ligand 27A | *Ccl27a* | 0.65 | 1.70 |
| 1427207_s_at | AFG3(ATPase family gene 3)-like 2 (yeast) | *Afg3l2* | 0.60 | 1.70 |
| 1428538_s_at | retinoic acid receptor responder (tazarotene induced) 2 | *Rarres2* | 0.50 | 1.70 |
| 1455940_x_at | WD repeat domain 6 | *Wdr6* | 0.63 | 1.70 |
| 1441876_x_at | zinc finger protein 93 | *Zfp93* | 0.63 | 1.70 |
| 1415917_at | methylenetetrahydrofolate dehydrogenase (NADP+ dependent), methenyltetrahydrofolate cyclohydrolase, formyltetrahydrofolate synthase | *Mthfd1* | 0.53 | 1.70 |
| 1455563_at | DEAD (Asp-Glu-Ala-Asp) box polypeptide 49 | *Ddx49* | 0.70 | 1.70 |
| 1452593_a_at | transcription elongation factor B (SIII), polypeptide 1 | *Tceb1* | 0.72 | 1.69 |
| 1427377_x_at | hydroxy-delta-5-steroid dehydrogenase, 3 beta- and steroid delta-isomerase 3 | *Hsd3b3* | 0.44 | 1.69 |
| 1434799_x_at | aldolase A, fructose-bisphosphate | *Aldoa* | 0.63 | 1.69 |
| 1423706_a_at | phosphogluconate dehydrogenase | *Pgd* | 0.47 | 1.69 |
| 1452622_a_at | TNFRSF1A-associated via death domain | *Tradd* | 0.77 | 1.69 |
| 1434621_at | transmembrane protein 204 | *Tmem204* | 0.46 | 1.69 |
| 1428449_at | general transcription factor IIIC, polypeptide 2, beta | *Gtf3c2* | 0.76 | 1.69 |
| 1449309_at | cytochrome P450, family 8, subfamily b, polypeptide 1 | *Cyp8b1* | 0.17 | 1.69 |
| 1428049_a_at | nudix (nucleoside diphosphate linked moiety X)-type motif 16-like 1 | *Nudt16l1* | 0.79 | 1.69 |
| 1417103_at | D-dopachrome tautomerase | *Ddt* | 0.50 | 1.69 |
| 1421135_a_at | CCR4-NOT transcription complex, subunit 8 | *Cnot8* | 0.50 | 1.69 |
| 1436070_at | glyoxalase 1 | *Glo1* | 0.36 | 1.69 |
| 1450776_at | 1-acylglycerol-3-phosphate O-acyltransferase 6 (lysophosphatidic acid acyltransferase, zeta) | *Agpat6* | 0.56 | 1.68 |
| 1423223_a_at | peroxiredoxin 6 | *Prdx6* | 0.45 | 1.68 |
| 1450854_at | proliferation-associated 2G4 | *Pa2g4* | 0.43 | 1.68 |
| 1419553_a_at | RAB geranylgeranyl transferase, b subunit | *Rabggtb* | 0.46 | 1.68 |
| 1450983_at | A kinase (PRKA) anchor protein 8 | *Akap8* | 0.53 | 1.68 |
| 1420629_a_at | DnaJ (Hsp40) homolog, subfamily A, member 3 | *Dnaja3* | 0.61 | 1.68 |
| 1434561_at | additional sex combs like 1 (Drosophila) | *Asxl1* | 0.72 | 1.68 |
| 1454995_at | dimethylarginine dimethylaminohydrolase 1 | *Ddah1* | 0.32 | 1.68 |
| 1435902_at | nudix (nucleoside diphosphate linked moiety X)-type motif 18 | *Nudt18* | 0.79 | 1.68 |
| 1427153_at | branched chain ketoacid dehydrogenase E1, beta polypeptide | *Bckdhb* | 0.58 | 1.67 |
| 1422686_s_at | exocyst complex component 4 | *Exoc4* | 0.43 | 1.67 |
| 1431085_a_at | protein-L-isoaspartate (D-aspartate) O-methyltransferase 1 | *Pcmt1* | 0.71 | 1.67 |
| 1431867_a_at | protein disulfide isomerase-like, testis expressed | *Pdilt* | 0.56 | 1.67 |
| 1416615_at | caseinolytic peptidase, ATP-dependent, proteolytic subunit homolog (E. coli) | *Clpp* | 0.76 | 1.67 |
| 1424126_at | aminolevulinic acid synthase 1 | *Alas1* | 0.57 | 1.67 |
| 1421945_a_at | ribosome production factor 2 homolog (S. cerevisiae) | *Rpf2* | 0.43 | 1.67 |
| 1443822_s_at | CDGSH iron sulfur domain 1 | *Cisd1* | 0.45 | 1.67 |
| 1417407_at | F-box and leucine-rich repeat protein 14 | *Fbxl14* | 0.62 | 1.67 |
| 1456018_at | bromodomain containing 9 | *Brd9* | 0.77 | 1.67 |
| 1456395_at | peroxisome proliferative activated receptor, gamma, coactivator 1 alpha | *Ppargc1a* | 0.60 | 1.67 |
| 1422058_at | nodal | *Nodal* | 0.68 | 1.66 |
| 1428082_at | acyl-CoA synthetase long-chain family member 5 | *Acsl5* | 0.57 | 1.66 |
| 1447993_a_at | small nucleolar RNA host gene (non-protein coding) 10 | *Snhg10* | 0.73 | 1.66 |
| 1416178_a_at | pleckstrin homology domain containing, family B (evectins) member 1 | *Plekhb1* | 0.75 | 1.66 |
| 1429613_at | dual specificity phosphatase 28 | *Dusp28* | 0.68 | 1.66 |
| 1455084_x_at | serine hydroxymethyltransferase 2 (mitochondrial) | *Shmt2* | 0.48 | 1.66 |
| 1416980_at | methyltransferase like 7B | *Mettl7b* | 0.54 | 1.66 |
| 1424639_a_at | 3-hydroxy-3-methylglutaryl-Coenzyme A lyase | *Hmgcl* | 0.71 | 1.66 |
| 1460557_at | suppressor of var1, 3-like 1 (S. cerevisiae) | *Supv3l1* | 0.49 | 1.66 |
| 1434814_x_at | glucose phosphate isomerase 1 | *Gpi1* | 0.49 | 1.66 |
| 1416362_a_at | FK506 binding protein 4 | *Fkbp4* | 0.76 | 1.66 |
| 1451098_at | chromatin modifying protein 1A | *Chmp1a* | 0.63 | 1.66 |
| 1448450_at | adenylate kinase 2 | *Ak2* | 0.46 | 1.65 |
| 1435279_at | seizure threshold 2 | *Szt2* | 0.36 | 1.65 |
| 1439617_s_at | phosphoenolpyruvate carboxykinase 1, cytosolic | *Pck1* | 0.52 | 1.65 |
| 1435419_at | retrotransposon gag domain containing 4 | *Rgag4* | 0.45 | 1.65 |
| 1436979_x_at | RNA binding motif protein 14 | *Rbm14* | 0.75 | 1.65 |
| 1456109_a_at | mitochondrial ribosomal protein S15 | *Mrps15* | 0.77 | 1.65 |
| 1425365_a_at | cytochrome P450, family 2, subfamily d, polypeptide 13 | *Cyp2d13* | 0.56 | 1.65 |
| 1455486_at | protein inhibitor of activated STAT 1 | *Pias1* | 0.78 | 1.65 |
| 1453782_at | ankyrin repeat domain 33B | *Ankrd33b* | 0.55 | 1.65 |
| 1448311_at | ubiquitin specific peptidase 5 (isopeptidase T) | *Usp5* | 0.65 | 1.65 |
| 1416605_at | NHP2 ribonucleoprotein homolog (yeast) | *Nhp2* | 0.72 | 1.65 |
| 1418706_at | solute carrier family 38, member 3 | *Slc38a3* | 0.61 | 1.65 |
| 1421969_a_at | fatty acid amide hydrolase | *Faah* | 0.71 | 1.65 |
| 1416948_at | mitochondrial ribosomal protein L23 | *Mrpl23* | 0.77 | 1.65 |
| 1436753_at | aarF domain containing kinase 5 | *Adck5* | 0.43 | 1.64 |
| 1422480_at | sorting nexin 3 | *Snx3* | 0.73 | 1.64 |
| 1448286_at | hydroxysteroid (17-beta) dehydrogenase 10 | *Hsd17b10* | 0.47 | 1.64 |
| 1416662_at | sarcosine dehydrogenase | *Sardh* | 0.58 | 1.64 |
| 1448563_at | prohibitin | *Phb* | 0.59 | 1.64 |
| 1450330_at | interleukin 10 | *Il10* | 0.51 | 1.64 |
| 1425300_at | dihydroxyacetone kinase 2 homolog (yeast) | *Dak* | 0.49 | 1.64 |
| 1438234_at | WD repeat domain 26 | *Wdr26* | 0.45 | 1.64 |
| 1428364_at | sodium channel modifier 1 | *Scnm1* | 0.68 | 1.63 |
| 1423811_at | splicing factor 3a, subunit 3 | *Sf3a3* | 0.76 | 1.63 |
| 1451227_a_at | solute carrier family 10 (sodium/bile acid cotransporter family), member 3 | *Slc10a3* | 0.65 | 1.63 |
| 1448287_at | polymerase (RNA) I polypeptide D | *Polr1d* | 0.66 | 1.63 |
| 1442402_at | SH3 domain containing ring finger 1 | *Sh3rf1* | 0.79 | 1.63 |
| 1455354_at | DDB1 and CUL4 associated factor 5 | *Dcaf5* | 0.56 | 1.63 |
| 1448307_at | proteasome (prosome, macropain) assembly chaperone 1 | *Psmg1* | 0.60 | 1.63 |
| 1437852_x_at | cleavage and polyadenylation specificity factor 3 | *Cpsf3* | 0.66 | 1.63 |
| 1460356_at | endothelial cell-specific adhesion molecule | *Esam* | 0.70 | 1.63 |
| 1450103_a_at | cytohesin 2 | *Cyth2* | 0.53 | 1.63 |
| 1426881_at | ubiquitin protein ligase E3C | *Ube3c* | 0.45 | 1.63 |
| 1429110_a_at | NOL1/NOP2/Sun domain family, member 4 | *Nsun4* | 0.42 | 1.63 |
| 1443586_at | FIP1 like 1 (S. cerevisiae) | *Fip1l1* | 0.76 | 1.63 |
| 1423711_at | NADH dehydrogenase (ubiquinone) 1 alpha subcomplex, assembly factor 1 | *Ndufaf1* | 0.61 | 1.63 |
| 1428281_at | TruB pseudouridine (psi) synthase homolog 1 (E. coli) | *Trub1* | 0.68 | 1.62 |
| 1424339_at | 2'-5' oligoadenylate synthetase-like 1 | *Oasl1* | 0.50 | 1.62 |
| 1436518_at | ubiquitin specific peptidase 46 | *Usp46* | 0.56 | 1.62 |
| 1451666_at | ATP citrate lyase | *Acly* | 0.38 | 1.62 |
| 1419277_at | ubiquitin specific peptidase 48 | *Usp48* | 0.70 | 1.62 |
| 1428182_at | phosphoribosyl pyrophosphate synthetase-associated protein 1 | *Prpsap1* | 0.62 | 1.62 |
| 1415727_at | apolipoprotein A-I binding protein | *Apoa1bp* | 0.66 | 1.62 |
| 1420493_a_at | phosphate cytidylyltransferase 2, ethanolamine | *Pcyt2* | 0.55 | 1.62 |
| 1434899_s_at | trinucleotide repeat containing 6a | *Tnrc6a* | 0.54 | 1.62 |
| 1452196_a_at | NCK-associated protein 1 | *Nckap1* | 0.72 | 1.62 |
| 1431464_a_at | phosphomannomutase 2 | *Pmm2* | 0.50 | 1.62 |
| 1433575_at | SRY-box containing gene 4 | *Sox4* | 0.64 | 1.62 |
| 1453145_at | phosphatidylserine decarboxylase, pseudogene 3 | *Pisd-ps3* | 0.49 | 1.62 |
| 1448848_at | torsin family 1, member B | *Tor1b* | 0.76 | 1.62 |
| 1433632_at | interferon regulatory factor 2 binding protein 2 | *Irf2bp2* | 0.77 | 1.62 |
| 1415776_at | aldehyde dehydrogenase family 3, subfamily A2 | *Aldh3a2* | 0.30 | 1.62 |
| 1424510_at | nudix (nucleoside diphosphate linked moiety X)-type motif 6 | *Nudt6* | 0.64 | 1.62 |
| 1425001_at | ring finger protein 146 | *Rnf146* | 0.75 | 1.62 |
| 1438155_x_at | phosphatidylinositol glycan anchor biosynthesis, class O | *Pigo* | 0.58 | 1.61 |
| 1455391_at | RAD23a homolog (S. cerevisiae) | *Rad23a* | 0.44 | 1.61 |
| 1417294_at | aldo-keto reductase family 7, member A5 (aflatoxin aldehyde reductase) | *Akr7a5* | 0.56 | 1.61 |
| 1428738_a_at | DNA segment, Chr 14, ERATO Doi 449, expressed | *D14Ertd449e* | 0.68 | 1.61 |
| 1452974_at | nucleolar protein 8 | *Nol8* | 0.46 | 1.61 |
| 1421820_a_at | neurofibromatosis 2 | *Nf2* | 0.58 | 1.61 |
| 1415677_at | dehydrogenase/reductase (SDR family) member 1 | *Dhrs1* | 0.66 | 1.61 |
| 1418522_at | metaxin 1 | *Mtx1* | 0.79 | 1.61 |
| 1440151_s_at | endothelial differentiation-related factor 1 | *Edf1* | 0.75 | 1.61 |
| 1416170_at | TNF receptor-associated protein 1 | *Trap1* | 0.61 | 1.61 |
| 1460167_at | aldehyde dehydrogenase family 7, member A1 | *Aldh7a1* | 0.39 | 1.61 |
| 1451380_at | zinc finger, FYVE domain containing 19 | *Zfyve19* | 0.74 | 1.61 |
| 1423729_a_at | ring finger protein 181 | *Rnf181* | 0.55 | 1.61 |
| 1418232_s_at | LIM and senescent cell antigen-like domains 1 | *Lims1* | 0.80 | 1.61 |
| 1424048_a_at | cytochrome b5 reductase 1 | *Cyb5r1* | 0.70 | 1.61 |
| 1419322_at | FYVE, RhoGEF and PH domain containing 6 | *Fgd6* | 0.49 | 1.60 |
| 1443486_at | component of oligomeric golgi complex 7 | *Cog7* | 0.49 | 1.60 |
| 1424839_a_at | NOL1/NOP2/Sun domain family, member 4 | *Nsun4* | 0.52 | 1.60 |
| 1460647_a_at | nuclear receptor subfamily 2, group F, member 6 | *Nr2f6* | 0.66 | 1.60 |
| 1457058_at | a disintegrin-like and metallopeptidase (reprolysin type) with thrombospondin type 1 motif, 2 | *Adamts2* | 0.51 | 1.60 |
| 1416902_a_at | cytochrome c oxidase, subunit Vb | *Cox5b* | 0.62 | 1.60 |
| 1430029_a_at | tetraspanin 31 | *Tspan31* | 0.56 | 1.60 |
| 1437452_x_at | voltage-dependent anion channel 1 | *Vdac1* | 0.56 | 1.60 |
| 1435626_a_at | homocysteine-inducible, endoplasmic reticulum stress-inducible, ubiquitin-like domain member 1 | *Herpud1* | 0.60 | 1.60 |
| 1428159_s_at | NADH dehydrogenase (ubiquinone) 1, alpha/beta subcomplex, 1 | *Ndufab1* | 0.65 | 1.60 |
| 1418250_at | ADP-ribosylation factor-like 4D | *Arl4d* | 0.43 | 1.60 |
| 1421262_at | lipase, endothelial | *Lipg* | 0.52 | 1.60 |
| 1416300_a_at | solute carrier family 25 (mitochondrial carrier, phosphate carrier), member 3 | *Slc25a3* | 0.50 | 1.60 |
| 1434387_at | integrin alpha FG-GAP repeat containing 3 | *Itfg3* | 0.42 | 1.60 |
| 1448537_at | tetratricopeptide repeat domain 1 | *Ttc1* | 0.77 | 1.60 |
| 1438931_s_at | sestrin 1 | *Sesn1* | 0.63 | 1.60 |
| 1428868_a_at | ornithine decarboxylase antizyme 1 | *Oaz1* | 0.65 | 1.60 |
| 1435344_at | transcription factor Dp 2 | *Tfdp2* | 0.56 | 1.60 |
| 1436481_at | ATPase, Cu++ transporting, beta polypeptide | *Atp7b* | 0.51 | 1.60 |
| 1435007_s_at | solute carrier family 25, member 47 | *Slc25a47* | 0.70 | 1.60 |
| 1416425_at | peroxisomal biogenesis factor 19 | *Pex19* | 0.57 | 1.60 |
| 1448448_a_at | choline kinase beta | *Chkb* | 0.52 | 1.59 |
| 1423479_at | nucleolar protein 11 | *Nol11* | 0.53 | 1.59 |
| 1422558_at | guanidinoacetate methyltransferase | *Gamt* | 0.64 | 1.59 |
| 1421082_s_at | barrier to autointegration factor 1 | *Banf1* | 0.64 | 1.59 |
| 1426740_s_at | armadillo repeat containing 5 | *Armc5* | 0.68 | 1.59 |
| 1450534_x_at | histocompatibility 2, K1, K region | *H2-K1* | 0.49 | 1.59 |
| 1418034_at | mitochondrial ribosomal protein S9 | *Mrps9* | 0.51 | 1.59 |
| 1436715_s_at | CDP-diacylglycerol--inositol 3-phosphatidyltransferase (phosphatidylinositol synthase) | *Cdipt* | 0.76 | 1.59 |
| 1453052_at | transmembrane protein 192 | *Tmem192* | 0.80 | 1.59 |
| 1416522_a_at | gene rich cluster, C10 gene | *Grcc10* | 0.70 | 1.59 |
| 1452501_at | cytochrome P450, family 2, subfamily c, polypeptide 38 | *Cyp2c38* | 0.65 | 1.59 |
| 1440849_at | RAS protein-specific guanine nucleotide-releasing factor 2 | *Rasgrf2* | 0.45 | 1.59 |
| 1448171_at | seven in absentia 2 | *Siah2* | 0.71 | 1.59 |
| 1452070_at | death effector domain-containing DNA binding protein 2 | *Dedd2* | 0.54 | 1.59 |
| 1460189_at | DDB1 and CUL4 associated factor 11 | *Dcaf11* | 0.53 | 1.58 |
| 1451992_at | adrenergic receptor kinase, beta 1 | *Adrbk1* | 0.50 | 1.58 |
| 1436632_at | ubinuclein 2 | *Ubn2* | 0.75 | 1.58 |
| 1451015_at | transketolase | *Tkt* | 0.54 | 1.58 |
| 1418079_at | proteaseome (prosome, macropain) 28 subunit, 3 | *Psme3* | 0.76 | 1.58 |
| 1448140_at | cytokine induced apoptosis inhibitor 1 | *Ciapin1* | 0.61 | 1.58 |
| 1451190_a_at | SH3-binding kinase 1 | *Sbk1* | 0.67 | 1.58 |
| 1424929_a_at | tripartite motif-containing 26 | *Trim26* | 0.72 | 1.58 |
| 1452078_a_at | solute carrier family 11 (proton-coupled divalent metal ion transporters), member 2 | *Slc11a2* | 0.74 | 1.58 |
| 1415831_at | proteasome (prosome, macropain) 26S subunit, non-ATPase, 2 | *Psmd2* | 0.59 | 1.58 |
| 1431008_at | chemokine (C-X-C motif) ligand 9 | *Cxcl9* | 0.79 | 1.58 |
| 1432492_a_at | 3-hydroxyanthranilate 3,4-dioxygenase | *Haao* | 0.57 | 1.58 |
| 1431189_a_at | fumarylacetoacetate hydrolase domain containing 2A | *Fahd2a* | 0.53 | 1.58 |
| 1451401_a_at | mitochondrial ubiquitin ligase activator of NFKB 1 | *Mul1* | 0.44 | 1.58 |
| 1423728_at | eukaryotic translation initiation factor 3, subunit L | *Eif3l* | 0.68 | 1.58 |
| 1450372_a_at | ribosomal protein L18 | *Rpl18* | 0.76 | 1.58 |
| 1451196_at | yippee-like 5 (Drosophila) | *Ypel5* | 0.52 | 1.58 |
| 1450957_a_at | sequestosome 1 | *Sqstm1* | 0.57 | 1.58 |
| 1425330_a_at | protein phosphatase 1B, magnesium dependent, beta isoform | *Ppm1b* | 0.43 | 1.58 |
| 1416205_at | galactosidase, beta 1 | *Glb1* | 0.68 | 1.58 |
| 1415830_at | origin recognition complex, subunit 5 | *Orc5* | 0.67 | 1.58 |
| 1437281_x_at | XPA binding protein 2 | *Xab2* | 0.36 | 1.58 |
| 1422478_a_at | acyl-CoA synthetase short-chain family member 2 | *Acss2* | 0.37 | 1.58 |
| 1451164_a_at | mitochondrial ribosomal protein S18B | *Mrps18b* | 0.64 | 1.58 |
| 1429538_a_at | serine/arginine-rich splicing factor 18 | *Sfrs18* | 0.59 | 1.58 |
| 1424815_at | glycogen synthase 2 | *Gys2* | 0.37 | 1.57 |
| 1434341_x_at | ubiquinol-cytochrome c reductase, complex III subunit X | *Uqcr10* | 0.58 | 1.57 |
| 1417705_at | OTU domain, ubiquitin aldehyde binding 1 | *Otub1* | 0.63 | 1.57 |
| 1422186_s_at | cytochrome b5 reductase 3 | *Cyb5r3* | 0.34 | 1.57 |
| 1419809_s_at | component of oligomeric golgi complex 4 | *Cog4* | 0.69 | 1.57 |
| 1424312_at | adiponectin receptor 1 | *Adipor1* | 0.56 | 1.57 |
| 1429473_at | zinc finger protein 869 | *Zfp869* | 0.65 | 1.57 |
| 1416990_at | retinoid X receptor beta | *Rxrb* | 0.75 | 1.57 |
| 1452986_at | homogentisate 1, 2-dioxygenase | *Hgd* | 0.45 | 1.57 |
| 1449213_at | L antigen family, member 3 | *Lage3* | 0.60 | 1.57 |
| 1434538_x_at | eukaryotic translation initiation factor 2B, subunit 2 beta | *Eif2b2* | 0.71 | 1.57 |
| 1425129_a_at | transaldolase 1 | *Taldo1* | 0.72 | 1.57 |
| 1451205_at | proteasome (prosome, macropain) subunit, beta type 4 | *Psmb4* | 0.71 | 1.57 |
| 1423912_at | alveolar soft part sarcoma chromosome region, candidate 1 (human) | *Aspscr1* | 0.54 | 1.57 |
| 1431125_a_at | threonyl-tRNA synthetase 2, mitochondrial (putative) | *Tars2* | 0.49 | 1.56 |
| 1423799_at | eukaryotic translation initiation factor 1 | *Eif1* | 0.49 | 1.56 |
| 1431190_x_at | fumarylacetoacetate hydrolase domain containing 2A | *Fahd2a* | 0.52 | 1.56 |
| 1429390_at | acid phosphatase-like 2 | *Acpl2* | 0.69 | 1.56 |
| 1415866_at | unc-45 homolog A (C. elegans) | *Unc45a* | 0.44 | 1.56 |
| 1418789_at | syntrophin, gamma 2 | *Sntg2* | 0.64 | 1.56 |
| 1426556_at | zinc finger protein 280D | *Zfp280d* | 0.60 | 1.56 |
| 1456086_x_at | polyglutamine-binding protein 1-like | *Pqbp1* | 0.67 | 1.56 |
| 1450380_at | ependymin related protein 1 (zebrafish) | *Epdr1* | 0.60 | 1.56 |
| 1418836_at | quinolinate phosphoribosyltransferase | *Qprt* | 0.78 | 1.56 |
| 1417666_at | deoxynucleotidyltransferase, terminal, interacting protein 1 | *Dnttip1* | 0.76 | 1.56 |
| 1416432_at | 6-phosphofructo-2-kinase/fructose-2,6-biphosphatase 3 | *Pfkfb3* | 0.73 | 1.56 |
| 1448365_at | exosome component 7 | *Exosc7* | 0.50 | 1.56 |
| 1451457_at | sterol-C5-desaturase (fungal ERG3, delta-5-desaturase) homolog (S. cerevisae) | *Sc5d* | 0.68 | 1.56 |
| 1460701_a_at | mitochondrial ribosomal protein L52 | *Mrpl52* | 0.41 | 1.56 |
| 1449347_a_at | x-linked lymphocyte-regulated protein 3A-like /// X-linked lymphocyte-regulated 4A /// X-linked lymphocyte-regulated 4B /// X-linked lymphocyte-regulated 4C | *LOC100505359 /// Xlr4a /// Xlr4b /// Xlr4c* | 0.79 | 1.56 |
| 1421943_at | transforming growth factor alpha | *Tgfa* | 0.65 | 1.56 |
| 1418347_at | coiled-coil domain containing 22 | *Ccdc22* | 0.74 | 1.56 |
| 1416168_at | serine (or cysteine) peptidase inhibitor, clade F, member 1 | *Serpinf1* | 0.44 | 1.56 |
| 1421874_a_at | mitochondrial ribosomal protein S23 | *Mrps23* | 0.77 | 1.56 |
| 1438649_x_at | phosphatidylethanolamine binding protein 1 | *Pebp1* | 0.72 | 1.56 |
| 1451093_at | polymerase (RNA) II (DNA directed) polypeptide E | *Polr2e* | 0.57 | 1.56 |
| 1426215_at | dopa decarboxylase | *Ddc* | 0.20 | 1.56 |
| 1426064_at | cytochrome P450, family 3, subfamily a, polypeptide 44 | *Cyp3a44* | 0.55 | 1.56 |
| 1418564_s_at | serpine1 mRNA binding protein 1 | *Serbp1* | 0.76 | 1.56 |
| 1426955_at | complement component 3 | *C3* | 0.57 | 1.56 |
| 1429972_s_at | thioredoxin reductase 2 | *Txnrd2* | 0.65 | 1.56 |
| 1439452_x_at | aspartyl aminopeptidase | *Dnpep* | 0.73 | 1.56 |
| 1428509_at | myosin IE | *Myo1e* | 0.79 | 1.55 |
| 1424670_s_at | zinc finger, FYVE domain containing 21 | *Zfyve21* | 0.67 | 1.55 |
| 1425891_a_at | GH regulated TBC protein 1 | *Grtp1* | 0.66 | 1.55 |
| 1439251_at | Iduronidase, alpha-L- | *Idua* | 0.74 | 1.55 |
| 1425373_a_at | proteasome assembly chaperone 2-like | *Psmg2* | 0.53 | 1.55 |
| 1450667_a_at | citrate synthase | *Cs* | 0.64 | 1.55 |
| 1416840_at | Mid1 interacting protein 1 (gastrulation specific G12-like (zebrafish)) | *Mid1ip1* | 0.53 | 1.55 |
| 1426586_at | solute carrier family 25 (mitochondrial carrier oxoglutarate carrier), member 11 | *Slc25a11* | 0.43 | 1.55 |
| 1430124_x_at | aldo-keto reductase family 1, member A4 (aldehyde reductase) | *Akr1a4* | 0.71 | 1.55 |
| 1434091_at | fatty acid amide hydrolase | *Faah* | 0.61 | 1.55 |
| 1419351_a_at | lethal, Chr 7, Rinchik 6 | *l7Rn6* | 0.70 | 1.55 |
| 1460228_at | upstream transcription factor 2 | *Usf2* | 0.48 | 1.55 |
| 1428365_a_at | lon peptidase 1, mitochondrial | *Lonp1* | 0.69 | 1.55 |
| 1443815_x_at | STE20-like kinase (yeast) | *Slk* | 0.72 | 1.55 |
| 1427370_at | amidohydrolase domain containing 1 | *Amdhd1* | 0.47 | 1.55 |
| 1430125_s_at | PQ loop repeat containing 1 | *Pqlc1* | 0.60 | 1.55 |
| 1436934_s_at | aconitase 2, mitochondrial | *Aco2* | 0.52 | 1.55 |
| 1451248_at | protein arginine N-methyltransferase 7 | *Prmt7* | 0.46 | 1.55 |
| 1417892_a_at | sirtuin 3 (silent mating type information regulation 2, homolog) 3 (S. cerevisiae) | *Sirt3* | 0.61 | 1.54 |
| 1425023_at | ubiquitin specific peptidase 3 | *Usp3* | 0.58 | 1.54 |
| 1416020_a_at | ATP synthase, H+ transporting, mitochondrial F0 complex, subunit c1 (subunit 9) | *Atp5g1* | 0.72 | 1.54 |
| 1451312_at | NADH dehydrogenase (ubiquinone) Fe-S protein 7 | *Ndufs7* | 0.62 | 1.54 |
| 1435548_at | MRS2 magnesium homeostasis factor homolog (S. cerevisiae) | *Mrs2* | 0.56 | 1.54 |
| 1423765_at | ATH1, acid trehalase-like 1 (yeast) | *Athl1* | 0.64 | 1.54 |
| 1427214_at | agmatine ureohydrolase (agmatinase) | *Agmat* | 0.57 | 1.54 |
| 1436884_x_at | Ewing sarcoma breakpoint region 1 | *Ewsr1* | 0.68 | 1.54 |
| 1427121_at | F-box protein 4 | *Fbxo4* | 0.75 | 1.54 |
| 1456036_x_at | glutathione S-transferase omega 1 | *Gsto1* | 0.59 | 1.54 |
| 1447894_x_at | Vacuolar protein sorting 52 (yeast) | *Vps52* | 0.76 | 1.54 |
| 1450985_a_at | tight junction protein 2 | *Tjp2* | 0.74 | 1.54 |
| 1459937_at | Argininosuccinate synthetase 1 | *Ass1* | 0.43 | 1.54 |
| 1417042_at | solute carrier family 37 (glucose-6-phosphate transporter), member 4 | *Slc37a4* | 0.48 | 1.54 |
| 1451266_at | mitochondrial ribosomal protein L50 | *Mrpl50* | 0.62 | 1.54 |
| 1449081_at | carboxylesterase 1D | *Ces1d* | 0.43 | 1.54 |
| 1436223_at | integrin beta 8 | *Itgb8* | 0.78 | 1.54 |
| 1418833_at | alanine-glyoxylate aminotransferase | *Agxt* | 0.70 | 1.54 |
| 1424266_s_at | carboxylesterase 1F | *Ces1f* | 0.37 | 1.54 |
| 1448214_at | pyruvate dehydrogenase (lipoamide) beta | *Pdhb* | 0.53 | 1.54 |
| 1437345_a_at | Bernardinelli-Seip congenital lipodystrophy 2 homolog (human) | *Bscl2* | 0.68 | 1.54 |
| 1424078_s_at | peroxisomal biogenesis factor 6 | *Pex6* | 0.71 | 1.53 |
| 1423724_at | ZW10 interactor | *Zwint* | 0.69 | 1.53 |
| 1416443_a_at | SUMO1 activating enzyme subunit 1 | *Sae1* | 0.66 | 1.53 |
| 1418395_at | solute carrier family 47, member 1 | *Slc47a1* | 0.48 | 1.53 |
| 1452266_at | LAS1-like (S. cerevisiae) | *Las1l* | 0.67 | 1.53 |
| 1426440_at | dehydrogenase/reductase (SDR family) member 7 | *Dhrs7* | 0.45 | 1.53 |
| 1416096_at | VPS33B interacting protein, apical-basolateral polarity regulator | *Vipar* | 0.54 | 1.53 |
| 1417060_at | protein phosphatase 1, regulatory (inhibitor) subunit 11 | *Ppp1r11* | 0.74 | 1.53 |
| 1452984_at | cyclin Y | *Ccny* | 0.50 | 1.53 |
| 1416733_at | muskelin 1, intracellular mediator containing kelch motifs | *Mkln1* | 0.50 | 1.53 |
| 1436298_x_at | phosphoribosylaminoimidazole carboxylase, phosphoribosylaminoribosylaminoimidazole, succinocarboxamide synthetase | *Paics* | 0.60 | 1.53 |
| 1439459_x_at | ATP citrate lyase | *Acly* | 0.44 | 1.53 |
| 1416728_at | casein kinase 2, beta polypeptide | *Csnk2b* | 0.55 | 1.53 |
| 1450444_a_at | nuclear receptor subfamily 1, group H, member 3 | *Nr1h3* | 0.79 | 1.53 |
| 1425281_a_at | TSC22 domain family, member 3 | *Tsc22d3* | 0.68 | 1.53 |
| 1421092_at | serine (or cysteine) peptidase inhibitor, clade A (alpha-1 antiproteinase, antitrypsin), member 12 | *Serpina12* | 0.26 | 1.53 |
| 1435101_at | Der1-like domain family, member 2 | *Derl2* | 0.60 | 1.53 |
| 1417223_at | CD2 antigen (cytoplasmic tail) binding protein 2 | *Cd2bp2* | 0.65 | 1.53 |
| 1423948_at | BCL2-associated athanogene 2 | *Bag2* | 0.57 | 1.52 |
| 1454792_s_at | selenophosphate synthetase 1 | *Sephs1* | 0.68 | 1.52 |
| 1423748_at | pyruvate dehydrogenase kinase, isoenzyme 1 | *Pdk1* | 0.45 | 1.52 |
| 1418858_at | aldehyde oxidase 3 | *Aox3* | 0.46 | 1.52 |
| 1416451_s_at | TAF8 RNA polymerase II, TATA box binding protein (TBP)-associated factorq | *Taf8* | 0.72 | 1.52 |
| 1448494_at | growth arrest specific 1 | *Gas1* | 0.42 | 1.52 |
| 1427248_at | Wolf-Hirschhorn syndrome candidate 2 (human) | *Whsc2* | 0.71 | 1.52 |
| 1415840_at | ELOVL family member 5, elongation of long chain fatty acids (yeast) | *Elovl5* | 0.40 | 1.52 |
| 1427332_at | mediator of RNA polymerase II transcription, subunit 19 homolog (yeast) | *Med19* | 0.72 | 1.52 |
| 1416826_a_at | mediator complex subunit 20 | *Med20* | 0.78 | 1.52 |
| 1426883_at | solute carrier family 25, member 45 | *Slc25a45* | 0.64 | 1.52 |
| 1444908_at | hyaluronic acid binding protein 4 | *Habp4* | 0.78 | 1.52 |
| 1425676_a_at | elongation of very long chain fatty acids (FEN1/Elo2, SUR4/Elo3, yeast)-like 1 | *Elovl1* | 0.67 | 1.52 |
| 1448792_a_at | cytochrome P450, family 2, subfamily f, polypeptide 2 | *Cyp2f2* | 0.34 | 1.52 |
| 1417965_at | pleckstrin homology domain containing, family A (phosphoinositide binding specific) member 1 | *Plekha1* | 0.64 | 1.52 |
| 1417203_at | ethylmalonic encephalopathy 1 | *Ethe1* | 0.52 | 1.52 |
| 1416554_at | PDZ and LIM domain 1 (elfin) | *Pdlim1* | 0.59 | 1.52 |
| 1434865_a_at | exocyst complex component 7 | *Exoc7* | 0.70 | 1.52 |
| 1448908_at | phosphatidic acid phosphatase type 2B | *Ppap2b* | 0.55 | 1.52 |
| 1416578_at | ring-box 1 | *Rbx1* | 0.61 | 1.52 |
| 1417741_at | liver glycogen phosphorylase | *Pygl* | 0.41 | 1.52 |
| 1434435_s_at | cytochrome c oxidase, subunit XVII assembly protein homolog (yeast) | *Cox17* | 0.76 | 1.52 |
| 1421873_s_at | RAB24, member RAS oncogene family | *Rab24* | 0.61 | 1.52 |
| 1448844_at | cytochrome b5 type B | *Cyb5b* | 0.78 | 1.52 |
| 1428315_at | EBNA1 binding protein 2 | *Ebna1bp2* | 0.68 | 1.52 |
| 1426223_at | tetratricopeptide repeat domain 39C | *Ttc39c* | 0.42 | 1.52 |
| 1423564_a_at | phosphoribosylaminoimidazole carboxylase, phosphoribosylaminoribosylaminoimidazole, succinocarboxamide synthetase | *Paics* | 0.62 | 1.52 |
| 1428134_at | coenzyme Q9 homolog (yeast) | *Coq9* | 0.62 | 1.52 |
| 1434510_at | 3'-phosphoadenosine 5'-phosphosulfate synthase 2 | *Papss2* | 0.46 | 1.51 |
| 1449026_at | interferon (alpha and beta) receptor 1 | *Ifnar1* | 0.80 | 1.51 |
| 1438029_at | regulation of nuclear pre-mRNA domain containing 2 | *Rprd2* | 0.56 | 1.51 |
| 1419158_a_at | histidyl-tRNA synthetase 2, mitochondrial (putative) | *Hars2* | 0.47 | 1.51 |
| 1416531_at | glutathione S-transferase omega 1 | *Gsto1* | 0.50 | 1.51 |
| 1424369_at | proteasome (prosome, macropain) inhibitor subunit 1 | *Psmf1* | 0.60 | 1.51 |
| 1415748_a_at | dynactin 5 | *Dctn5* | 0.74 | 1.51 |
| 1441814_s_at | RPA interacting protein | *Rpain* | 0.78 | 1.51 |
| 1428916_s_at | sirtuin 5 (silent mating type information regulation 2 homolog) 5 (S. cerevisiae) | *Sirt5* | 0.53 | 1.51 |
| 1453083_at | vomeronasal 2, receptor 29 | *Vmn2r29* | 0.53 | 1.51 |
| 1438391_x_at | hydroxysteroid (17-beta) dehydrogenase 10 | *Hsd17b10* | 0.45 | 1.51 |
| 1450668_s_at | heat shock protein 1 (chaperonin 10) | *Hspe1* | 0.75 | 1.51 |
| 1443729_at | metastasis suppressor 1 | *Mtss1* | 0.48 | 1.51 |
| 1426277_at | transmembrane protein 203 | *Tmem203* | 0.55 | 1.51 |
| 1423806_at | integrator complex subunit 4 | *Ints4* | 0.59 | 1.51 |
| 1451727_at | SLU7 splicing factor homolog (S. cerevisiae) | *Slu7* | 0.57 | 1.51 |
| 1436012_s_at | secernin 2 | *Scrn2* | 0.70 | 1.51 |
| 1454765_at | general transcription factor IIIC, polypeptide 3 | *Gtf3c3* | 0.78 | 1.51 |
| 1417220_at | fumarylacetoacetate hydrolase | *Fah* | 0.65 | 1.51 |
| 1423716_s_at | ATP synthase, H+ transporting, mitochondrial F1 complex, delta subunit | *Atp5d* | 0.69 | 1.51 |
| 1416684_at | fibrillarin | *Fbl* | 0.66 | 1.51 |
| 1426332_a_at | claudin 3 | *Cldn3* | 0.77 | 1.51 |
| 1416773_at | WEE 1 homolog 1 (S. pombe) | *Wee1* | 0.70 | 1.50 |
| 1424968_at | carboxylesterase 2G | *Ces2g* | 0.56 | 1.50 |
| 1447452_at | Receptor accessory protein 3 | *Reep3* | 0.56 | 1.50 |
| 1446957_s_at | NEDD4 binding protein 1 | *N4bp1* | 0.62 | 1.50 |
| 1421278_s_at | spectrin alpha 1 | *Spna1* | 0.59 | 1.50 |
| 1431002_x_at | fumarylacetoacetate hydrolase domain containing 2A | *Fahd2a* | 0.36 | 1.50 |
| 1421985_a_at | eukaryotic translation initiation factor 4E member 2 | *Eif4e2* | 0.62 | 1.50 |
| 1440822_x_at | RalBP1 associated Eps domain containing protein | *Reps1* | 0.67 | 1.50 |
| 1452044_at | actin related protein 2/3 complex, subunit 5-like | *Arpc5l* | 0.75 | 1.50 |
| 1455752_a_at | transmembrane protein 134 | *Tmem134* | 0.69 | 1.50 |
| 1428553_at | glutaredoxin 5 homolog (S. cerevisiae) | *Glrx5* | 0.56 | 1.50 |
| 1451299_at | protein kinase, X-linked | *Prkx* | 0.78 | 1.50 |
| 1418804_at | succinate receptor 1 | *Sucnr1* | 0.29 | 1.50 |
| 1418590_at | karyopherin (importin) alpha 6 | *Kpna6* | 0.59 | 1.50 |
| 1424352_at | cytochrome P450, family 4, subfamily a, polypeptide 12a | *Cyp4a12a* | 0.07 | 1.50 |
| 1425006_a_at | vaccinia related kinase 1 | *Vrk1* | 0.63 | 1.50 |
| 1437151_at | ubiquitin specific peptidase 22 | *Usp22* | 0.63 | 1.50 |
| 1452064_at | mediator complex subunit 23 | *Med23* | 0.60 | 1.50 |
| 1456169_at | thiosulfate sulfurtransferase (rhodanese)-like domain containing 1 | *Tstd1* | 0.55 | 1.50 |
| 1425117_at | aspartate dehydrogenase domain containing | *Aspdh* | 0.65 | 1.50 |
| 1433659_at | tubulin, gamma complex associated protein 4 | *Tubgcp4* | 0.62 | 1.50 |
| 1428081_at | kelch-like 21 (Drosophila) | *Klhl21* | 0.53 | 1.50 |
| 1449413_at | Mpv17 transgene, kidney disease mutant-like | *Mpv17l* | 0.54 | 1.50 |
| 1452832_s_at | CDP-diacylglycerol synthase (phosphatidate cytidylyltransferase) 2 | *Cds2* | 0.46 | 1.49 |
| 1452790_x_at | NADH dehydrogenase (ubiquinone) 1 alpha subcomplex, 3 | *Ndufa3* | 0.62 | 1.49 |
| 1436050_x_at | hairy and enhancer of split 6 (Drosophila) | *Hes6* | 0.44 | 1.49 |
| 1417689_a_at | PDZK1 interacting protein 1 | *Pdzk1ip1* | 0.46 | 1.49 |
| 1416039_x_at | cysteine rich protein 61 | *Cyr61* | 0.52 | 1.49 |
| 1437131_x_at | mitochondrial ribosomal protein L11 | *Mrpl11* | 0.75 | 1.49 |
| 1455951_at | methionine-tRNA synthetase | *Mars* | 0.67 | 1.49 |
| 1448297_a_at | tyrosine kinase, non-receptor, 2 | *Tnk2* | 0.53 | 1.49 |
| 1455101_at | phosphatase and actin regulator 2 | *Phactr2* | 0.48 | 1.49 |
| 1433604_x_at | aldolase A, fructose-bisphosphate | *Aldoa* | 0.56 | 1.49 |
| 1428344_at | phosphatidic acid phosphatase type 2 domain containing 2 | *Ppapdc2* | 0.64 | 1.49 |
| 1441856_x_at | trypsin domain containing 1 | *Tysnd1* | 0.68 | 1.49 |
| 1433817_at | 1-acylglycerol-3-phosphate O-acyltransferase 3 | *Agpat3* | 0.77 | 1.49 |
| 1436703_x_at | small nuclear RNA activating complex, polypeptide 2 | *Snapc2* | 0.65 | 1.49 |
| 1454889_x_at | transmembrane and coiled coil domains 3 | *Tmcc3* | 0.68 | 1.49 |
| 1433878_at | mitochondrial ribosomal protein S26 | *Mrps26* | 0.72 | 1.49 |
| 1427941_at | Dicer1, Dcr-1 homolog (Drosophila) | *Dicer1* | 0.71 | 1.49 |
| 1424687_at | HEAT repeat containing 6 | *Heatr6* | 0.32 | 1.49 |
| 1424296_at | glutamate-cysteine ligase, catalytic subunit | *Gclc* | 0.46 | 1.49 |
| 1455928_x_at | leucine-zipper-like transcriptional regulator, 1 | *Lztr1* | 0.43 | 1.49 |
| 1460165_at | protein phosphatase 1, catalytic subunit, alpha isoform | *Ppp1ca* | 0.76 | 1.49 |
| 1448172_at | malate dehydrogenase 1, NAD (soluble) | *Mdh1* | 0.52 | 1.49 |
| 1428666_at | asparaginyl-tRNA synthetase | *Nars* | 0.63 | 1.48 |
| 1427903_at | phosphohistidine phosphatase 1 | *Phpt1* | 0.80 | 1.48 |
| 1423491_at | F-box protein 3 | *Fbxo3* | 0.56 | 1.48 |
| 1423858_a_at | 3-hydroxy-3-methylglutaryl-Coenzyme A synthase 2 | *Hmgcs2* | 0.48 | 1.48 |
| 1421374_a_at | FXYD domain-containing ion transport regulator 1 | *Fxyd1* | 0.77 | 1.48 |
| 1423277_at | protein tyrosine phosphatase, receptor type, K | *Ptprk* | 0.72 | 1.48 |
| 1428146_s_at | acetyl-Coenzyme A acyltransferase 2 (mitochondrial 3-oxoacyl-Coenzyme A thiolase) | *Acaa2* | 0.65 | 1.48 |
| 1423111_at | ATP synthase, H+ transporting, mitochondrial F1 complex, alpha subunit 1 | *Atp5a1* | 0.55 | 1.48 |
| 1419081_at | autophagy-related 10 (yeast) | *Atg10* | 0.69 | 1.48 |
| 1416667_at | phenylalkylamine Ca2+ antagonist (emopamil) binding protein | *Ebp* | 0.69 | 1.48 |
| 1429107_at | ubiquitin protein ligase E3 component n-recognin 3 | *Ubr3* | 0.44 | 1.48 |
| 1437377_a_at | polymerase (RNA) mitochondrial (DNA directed) | *Polrmt* | 0.59 | 1.48 |
| 1425742_a_at | TSC22 domain family, member 1 | *Tsc22d1* | 0.22 | 1.48 |
| 1424754_at | membrane-spanning 4-domains, subfamily A, member 7 | *Ms4a7* | 0.68 | 1.48 |
| 1451548_at | uridine phosphorylase 2 | *Upp2* | 0.60 | 1.48 |
| 1417836_at | glutathione peroxidase 7 | *Gpx7* | 0.57 | 1.48 |
| 1432264_x_at | cytochrome c oxidase subunit VIIa polypeptide 2-like | *Cox7a2l* | 0.74 | 1.48 |
| 1451383_a_at | conserved helix-loop-helix ubiquitous kinase | *Chuk* | 0.55 | 1.48 |
| 1452799_at | FGGY carbohydrate kinase domain containing | *Fggy* | 0.65 | 1.48 |
| 1451438_s_at | C-type lectin domain family 2, member h | *Clec2h* | 0.25 | 1.48 |
| 1448322_a_at | cytochrome c oxidase subunit IV isoform 1 | *Cox4i1* | 0.75 | 1.48 |
| 1460360_at | asparaginase like 1 | *Asrgl1* | 0.51 | 1.48 |
| 1428769_at | TatD DNase domain containing 3 | *Tatdn3* | 0.48 | 1.47 |
| 1415980_at | ATP synthase, H+ transporting, mitochondrial F0 complex, subunit C2 (subunit 9) | *Atp5g2* | 0.78 | 1.47 |
| 1451501_a_at | growth hormone receptor | *Ghr* | 0.41 | 1.47 |
| 1418174_at | D site albumin promoter binding protein | *Dbp* | 0.44 | 1.47 |
| 1455590_at | NAD(P)H dehydrogenase, quinone 2 | *Nqo2* | 0.42 | 1.47 |
| 1444150_at | erythrocyte protein band 4.1 | *Epb4.1* | 0.41 | 1.47 |
| 1430287_s_at | HemK methyltransferase family member 1 | *Hemk1* | 0.59 | 1.47 |
| 1453198_at | zinc finger protein 955B | *Zfp955b* | 0.41 | 1.47 |
| 1418886_s_at | isocitrate dehydrogenase 3 (NAD+) beta | *Idh3b* | 0.48 | 1.47 |
| 1460341_at | pleckstrin homology domain containing, family B (evectins) member 2 | *Plekhb2* | 0.75 | 1.47 |
| 1425859_a_at | proteasome (prosome, macropain) 26S subunit, non-ATPase, 4 | *Psmd4* | 0.73 | 1.47 |
| 1417064_at | jagunal homolog 1 (Drosophila) | *Jagn1* | 0.71 | 1.47 |
| 1424724_a_at | DNA segment, Chr 16, ERATO Doi 472, expressed | *D16Ertd472e* | 0.64 | 1.47 |
| 1419747_at | asialoglycoprotein receptor 2 | *Asgr2* | 0.78 | 1.47 |
| 1435750_at | GTP cyclohydrolase I feedback regulator | *Gchfr* | 0.69 | 1.47 |
| 1422888_at | ring finger protein 5 | *Rnf5* | 0.45 | 1.47 |
| 1417919_at | protein phosphatase 1, regulatory (inhibitor) subunit 7 | *Ppp1r7* | 0.65 | 1.47 |
| 1435865_at | histone cluster 3, H2a | *Hist3h2a* | 0.77 | 1.47 |
| 1418458_at | anaphase promoting complex subunit 7 | *Anapc7* | 0.76 | 1.47 |
| 1426243_at | cystathionase (cystathionine gamma-lyase) | *Cth* | 0.70 | 1.47 |
| 1417573_at | methylmalonic aciduria (cobalamin deficiency) cblD type, with homocystinuria | *Mmadhc* | 0.63 | 1.47 |
| 1423766_at | PAK1 interacting protein 1 | *Pak1ip1* | 0.59 | 1.47 |
| 1451197_s_at | GATA zinc finger domain containing 2A | *Gatad2a* | 0.63 | 1.47 |
| 1448222_x_at | cytochrome c oxidase, subunit VIIIa | *Cox8a* | 0.59 | 1.47 |
| 1417767_at | cytochrome b5 type B | *Cyb5b* | 0.52 | 1.47 |
| 1451974_at | oxysterol binding protein-like 2 | *Osbpl2* | 0.63 | 1.47 |
| 1435881_at | poly(rC) binding protein 2 | *Pcbp2* | 0.58 | 1.47 |
| 1448400_a_at | SWI/SNF related, matrix associated, actin dependent regulator of chromatin, subfamily d, member 2 | *Smarcd2* | 0.73 | 1.47 |
| 1416377_at | programmed cell death 7 | *Pdcd7* | 0.49 | 1.47 |
| 1433488_x_at | glucosamine (N-acetyl)-6-sulfatase | *Gns* | 0.66 | 1.47 |
| 1418364_a_at | ferritin light chain 1 | *Ftl1* | 0.74 | 1.47 |
| 1416963_at | ubiquitin associated domain containing 1 | *Ubac1* | 0.59 | 1.47 |
| 1437630_at | large subunit GTPase 1 homolog (S. cerevisiae) | *Lsg1* | 0.59 | 1.47 |
| 1428506_at | 5-aminoimidazole-4-carboxamide ribonucleotide formyltransferase/IMP cyclohydrolase | *Atic* | 0.61 | 1.47 |
| 1452464_a_at | methionyl aminopeptidase type 1D (mitochondrial) | *Metap1d* | 0.60 | 1.46 |
| 1428782_a_at | ubiquinol-cytochrome c reductase core protein 1 | *Uqcrc1* | 0.47 | 1.46 |
| 1436291_a_at | dihydropyrimidinase | *Dpys* | 0.61 | 1.46 |
| 1415950_a_at | phosphatidylethanolamine binding protein 1 | *Pebp1* | 0.63 | 1.46 |
| 1453462_at | Fas apoptotic inhibitory molecule 3 | *Faim3* | 0.61 | 1.46 |
| 1428682_at | zinc finger CCCH type containing 6 | *Zc3h6* | 0.58 | 1.46 |
| 1436532_at | doublecortin-like kinase 3 | *Dclk3* | 0.49 | 1.46 |
| 1460366_at | echinoderm microtubule associated protein like 3 | *Eml3* | 0.66 | 1.46 |
| 1417649_at | cyclin-dependent kinase inhibitor 1C (P57) | *Cdkn1c* | 0.80 | 1.46 |
| 1427896_at | suppressor of defective silencing 3 homolog (S. cerevisiae) | *Suds3* | 0.73 | 1.46 |
| 1437045_at | mitogen-activated protein kinase 8 | *Mapk8* | 0.76 | 1.46 |
| 1448615_at | copper chaperone for superoxide dismutase | *Ccs* | 0.70 | 1.46 |
| 1448805_at | upstream transcription factor 1 | *Usf1* | 0.73 | 1.46 |
| 1451348_at | DEP domain containing 6 | *Depdc6* | 0.54 | 1.46 |
| 1423548_s_at | ERGIC and golgi 3 | *Ergic3* | 0.63 | 1.46 |
| 1422859_a_at | ribosomal protein L23 | *Rpl23* | 0.73 | 1.46 |
| 1423827_s_at | nucleolar complex associated 4 homolog (S. cerevisiae) | *Noc4l* | 0.61 | 1.46 |
| 1451361_a_at | patatin-like phospholipase domain containing 7 | *Pnpla7* | 0.56 | 1.46 |
| 1451873_a_at | MAD homolog 5 (Drosophila) | *Smad5* | 0.61 | 1.46 |
| 1449349_at | nudix (nucleoside diphosphate linked moiety X)-type motif 1 | *Nudt1* | 0.53 | 1.46 |
| 1451297_at | gulonolactone (L-) oxidase | *Gulo* | 0.79 | 1.46 |
| 1420984_at | phosphatidylcholine transfer protein | *Pctp* | 0.47 | 1.46 |
| 1416570_s_at | G elongation factor, mitochondrial 1 | *Gfm1* | 0.49 | 1.46 |
| 1437984_x_at | HLA-B-associated transcript 1A | *Bat1a* | 0.67 | 1.45 |
| 1423663_at | folliculin | *Flcn* | 0.75 | 1.45 |
| 1460387_a_at | sialic acid acetylesterase | *Siae* | 0.56 | 1.45 |
| 1434821_at | bromodomain containing 1 | *Brd1* | 0.58 | 1.45 |
| 1425211_at | glutamyl-tRNA(Gln) amidotransferase, subunit C homolog (bacterial) | *Gatc* | 0.56 | 1.45 |
| 1453099_at | casein kinase 2, alpha prime polypeptide | *Csnk2a2* | 0.79 | 1.45 |
| 1449072_a_at | N-6 adenine-specific DNA methyltransferase 2 (putative) | *N6amt2* | 0.78 | 1.45 |
| 1457455_at | zinc finger protein 280D | *Zfp280d* | 0.65 | 1.45 |
| 1448999_at | trafficking protein particle complex 5 | *Trappc5* | 0.61 | 1.45 |
| 1424647_at | gamma-aminobutyric acid (GABA) A receptor, pi | *Gabrp* | 0.36 | 1.45 |
| 1426785_s_at | monoglyceride lipase | *Mgll* | 0.56 | 1.45 |
| 1436456_at | solute carrier family 38, member 9 | *Slc38a9* | 0.68 | 1.45 |
| 1460444_at | arrestin, beta 1 | *Arrb1* | 0.79 | 1.45 |
| 1438055_at | retinoic acid receptor responder (tazarotene induced) 1 | *Rarres1* | 0.38 | 1.45 |
| 1448465_at | 4-nitrophenylphosphatase domain and non-neuronal SNAP25-like protein homolog 1 (C. elegans) | *Nipsnap1* | 0.51 | 1.45 |
| 1424792_at | ribonuclease P 40 subunit (human) | *Rpp40* | 0.54 | 1.45 |
| 1416037_a_at | chaperonin containing Tcp1, subunit 2 (beta) | *Cct2* | 0.68 | 1.45 |
| 1449460_at | ankyrin repeat and SOCS box-containing 13 | *Asb13* | 0.77 | 1.45 |
| 1455805_x_at | coiled-coil domain containing 22 | *Ccdc22* | 0.70 | 1.45 |
| 1433958_at | centrobin, centrosomal BRCA2 interacting protein | *Cntrob* | 0.48 | 1.45 |
| 1434035_at | DnaJ (Hsp40) homolog, subfamily B, member 6 | *Dnajb6* | 0.58 | 1.44 |
| 1457696_at | Rab interacting lysosomal protein | *Rilp* | 0.78 | 1.44 |
| 1422650_a_at | RIO kinase 3 (yeast) | *Riok3* | 0.73 | 1.44 |
| 1415888_at | hepatoma-derived growth factor | *Hdgf* | 0.71 | 1.44 |
| 1422665_a_at | protein-L-isoaspartate (D-aspartate) O-methyltransferase 1 | *Pcmt1* | 0.56 | 1.44 |
| 1416890_at | WD repeat domain 74 | *Wdr74* | 0.73 | 1.44 |
| 1419510_at | carboxylesterase 1E | *Ces1e* | 0.31 | 1.44 |
| 1420726_x_at | trimethyllysine hydroxylase, epsilon | *Tmlhe* | 0.66 | 1.44 |
| 1415703_at | HECT, UBA and WWE domain containing 1 | *Huwe1* | 0.70 | 1.44 |
| 1454860_x_at | defender against cell death 1 | *Dad1* | 0.78 | 1.44 |
| 1416526_a_at | Parkinson disease (autosomal recessive, early onset) 7 | *Park7* | 0.65 | 1.44 |
| 1417242_at | eukaryotic translation initiation factor 4A3 | *Eif4a3* | 0.69 | 1.44 |
| 1429125_at | zinc finger and BTB domain containing 9 | *Zbtb9* | 0.76 | 1.44 |
| 1424512_a_at | thioredoxin domain containing 9 | *Txndc9* | 0.68 | 1.44 |
| 1449635_at | PRP19/PSO4 pre-mRNA processing factor 19 homolog (S. cerevisiae) | *Prpf19* | 0.63 | 1.44 |
| 1423644_at | aconitase 1 | *Aco1* | 0.51 | 1.44 |
| 1415744_at | H2-K region expressed gene 2 | *H2-Ke2* | 0.61 | 1.44 |
| 1418697_at | indolethylamine N-methyltransferase | *Inmt* | 0.56 | 1.44 |
| 1417515_at | U7 snRNP-specific Sm-like protein LSM10 | *Lsm10* | 0.68 | 1.44 |
| 1419964_s_at | hepatoma-derived growth factor | *Hdgf* | 0.80 | 1.44 |
| 1438550_x_at | serine racemase | *Srr* | 0.50 | 1.44 |
| 1416679_at | ATP-binding cassette, sub-family D (ALD), member 3 | *Abcd3* | 0.38 | 1.44 |
| 1455061_a_at | acetyl-Coenzyme A acyltransferase 2 (mitochondrial 3-oxoacyl-Coenzyme A thiolase) | *Acaa2* | 0.52 | 1.44 |
| 1449209_a_at | retinol dehydrogenase 11 | *Rdh11* | 0.63 | 1.44 |
| 1424355_a_at | transcriptional regulator, SIN3B (yeast) | *Sin3b* | 0.68 | 1.43 |
| 1418300_a_at | MAP kinase-interacting serine/threonine kinase 2 | *Mknk2* | 0.71 | 1.43 |
| 1417651_at | cytochrome P450, family 2, subfamily c, polypeptide 29 | *Cyp2c29* | 0.53 | 1.43 |
| 1418767_at | cytochrome P450, family 4, subfamily f, polypeptide 13 | *Cyp4f13* | 0.47 | 1.43 |
| 1426735_at | isoleucine-tRNA synthetase 2, mitochondrial | *Iars2* | 0.44 | 1.43 |
| 1416964_at | eukaryotic elongation factor, selenocysteine-tRNA-specific | *Eefsec* | 0.55 | 1.43 |
| 1437194_x_at | leucine-rich repeats and WD repeat domain containing 1 | *Lrwd1* | 0.77 | 1.43 |
| 1431087_at | SPC24, NDC80 kinetochore complex component, homolog (S. cerevisiae) | *Spc24* | 0.64 | 1.43 |
| 1451407_at | immunoglobulin superfamily, member 5 | *Igsf5* | 0.77 | 1.43 |
| 1422524_at | ATP-binding cassette, sub-family B (MDR/TAP), member 6 | *Abcb6* | 0.64 | 1.43 |
| 1442154_at | zinc finger (CCCH type), RNA binding motif and serine/arginine rich 2 | *Zrsr2* | 0.77 | 1.43 |
| 1432472_a_at | methylcrotonoyl-Coenzyme A carboxylase 2 (beta) | *Mccc2* | 0.65 | 1.43 |
| 1416202_at | prohibitin 2 | *Phb2* | 0.64 | 1.43 |
| 1450048_a_at | isocitrate dehydrogenase 2 (NADP+), mitochondrial | *Idh2* | 0.73 | 1.43 |
| 1419456_at | dicarbonyl L-xylulose reductase | *Dcxr* | 0.56 | 1.43 |
| 1456279_a_at | B-cell receptor-associated protein 31 | *Bcap31* | 0.73 | 1.43 |
| 1460232_s_at | hydroxy-delta-5-steroid dehydrogenase, 3 beta- and steroid delta-isomerase 2 | *Hsd3b2* | 0.55 | 1.43 |
| 1424899_at | nicotinamide nucleotide adenylyltransferase 3 | *Nmnat3* | 0.54 | 1.43 |
| 1455654_at | Holliday junction recognition protein | *Hjurp* | 0.58 | 1.43 |
| 1456580_s_at | ATP synthase, H+ transporting, mitochondrial F1 complex, delta subunit | *Atp5d* | 0.61 | 1.43 |
| 1438991_x_at | protein phosphatase 2 (formerly 2A), regulatory subunit A (PR 65), alpha isoform | *Ppp2r1a* | 0.73 | 1.43 |
| 1438211_s_at | D site albumin promoter binding protein | *Dbp* | 0.41 | 1.43 |
| 1434138_at | prune homolog (Drosophila) | *Prune* | 0.42 | 1.43 |
| 1417661_at | RAD52 motif 1 | *Rdm1* | 0.69 | 1.42 |
| 1450098_at | dihydrodipicolinate synthase-like, mitochondrial | *Dhdpsl* | 0.65 | 1.42 |
| 1428431_at | zinc finger, CCHC domain containing 24 | *Zcchc24* | 0.64 | 1.42 |
| 1417112_at | ADP-ribosylation factor-like 2 binding protein | *Arl2bp* | 0.74 | 1.42 |
| 1451114_at | CKLF-like MARVEL transmembrane domain containing 6 | *Cmtm6* | 0.45 | 1.42 |
| 1425704_at | dehydrogenase/reductase (SDR family) member 11 | *Dhrs11* | 0.76 | 1.42 |
| 1428145_at | acetyl-Coenzyme A acyltransferase 2 (mitochondrial 3-oxoacyl-Coenzyme A thiolase) | *Acaa2* | 0.61 | 1.42 |
| 1435732_x_at | ATPase, H+ transporting, lysosomal V0 subunit C | *Atp6v0c* | 0.69 | 1.42 |
| 1448418_s_at | DDB1 and CUL4 associated factor 11 | *Dcaf11* | 0.65 | 1.42 |
| 1427440_a_at | afamin | *Afm* | 0.77 | 1.42 |
| 1423067_at | CDK5 regulatory subunit associated protein 3 | *Cdk5rap3* | 0.79 | 1.42 |
| 1426862_at | aftiphilin | *Aftph* | 0.76 | 1.42 |
| 1416112_at | cytochrome c oxidase, subunit VIIIa | *Cox8a* | 0.68 | 1.42 |
| 1424843_a_at | growth arrest specific 5 | *Gas5* | 0.75 | 1.42 |
| 1416349_at | mitochondrial ribosomal protein L34 | *Mrpl34* | 0.76 | 1.42 |
| 1456748_a_at | 4-nitrophenylphosphatase domain and non-neuronal SNAP25-like protein homolog 1 (C. elegans) | *Nipsnap1* | 0.51 | 1.42 |
| 1437947_x_at | voltage-dependent anion channel 1 | *Vdac1* | 0.77 | 1.42 |
| 1448910_at | peroxisomal trans-2-enoyl-CoA reductase | *Pecr* | 0.44 | 1.42 |
| 1437406_x_at | insulin-like growth factor binding protein 4 | *Igfbp4* | 0.72 | 1.42 |
| 1417071_s_at | cytochrome P450, family 4, subfamily v, polypeptide 3 | *Cyp4v3* | 0.28 | 1.42 |
| 1431947_at | low density lipoprotein receptor | *Ldlr* | 0.74 | 1.42 |
| 1423191_at | formin binding protein 4 | *Fnbp4* | 0.65 | 1.42 |
| 1456374_x_at | eukaryotic translation initiation factor 3, subunit C | *Eif3c* | 0.73 | 1.42 |
| 1430856_at | peroxisomal biogenesis factor 11 gamma | *Pex11c* | 0.55 | 1.42 |
| 1429407_at | peroxisomal biogenesis factor 11 gamma | *Pex11c* | 0.59 | 1.42 |
| 1416155_at | high mobility group box 3 | *Hmgb3* | 0.61 | 1.42 |
| 1418713_at | pterin 4 alpha carbinolamine dehydratase/dimerization cofactor of hepatocyte nuclear factor 1 alpha (TCF1) 1 | *Pcbd1* | 0.78 | 1.42 |
| 1437437_x_at | aspartyl aminopeptidase | *Dnpep* | 0.74 | 1.42 |
| 1416829_at | ATP synthase, H+ transporting mitochondrial F1 complex, beta subunit | *Atp5b* | 0.59 | 1.42 |
| 1424955_at | HAUS augmin-like complex, subunit 1 | *Haus1* | 0.51 | 1.42 |
| 1447521_x_at | Rho GTPase activating protein 39 | *Arhgap39* | 0.45 | 1.42 |
| 1448332_at | peroxisomal biogenesis factor 19 | *Pex19* | 0.61 | 1.42 |
| 1424776_a_at | solute carrier family 25, member 28 | *Slc25a28* | 0.55 | 1.42 |
| 1416921_x_at | aldolase A, fructose-bisphosphate | *Aldoa* | 0.71 | 1.42 |
| 1425189_a_at | mitochondrial ribosomal protein L15 | *Mrpl15* | 0.72 | 1.42 |
| 1439590_at | X Kell blood group precursor related family member 8 homolog | *Xkr8* | 0.56 | 1.42 |
| 1416697_at | dipeptidylpeptidase 4 | *Dpp4* | 0.60 | 1.42 |
| 1452123_s_at | FERM domain containing 4B | *Frmd4b* | 0.77 | 1.42 |
| 1451149_at | phosphoglucomutase 2 | *Pgm2* | 0.54 | 1.42 |
| 1448398_s_at | ribosomal protein L22 | *Rpl22* | 0.76 | 1.41 |
| 1438961_s_at | bleomycin hydrolase | *Blmh* | 0.65 | 1.41 |
| 1451573_a_at | syntaxin 4A (placental) | *Stx4a* | 0.80 | 1.41 |
| 1453399_at | cyclin T2 | *Ccnt2* | 0.79 | 1.41 |
| 1423749_s_at | RAN GTPase activating protein 1 | *Rangap1* | 0.78 | 1.41 |
| 1418850_at | enhancer of polycomb homolog 1 (Drosophila) | *Epc1* | 0.55 | 1.41 |
| 1427564_at | diaphanous homolog 2 (Drosophila) | *Diap2* | 0.70 | 1.41 |
| 1426460_a_at | UDP-glucose pyrophosphorylase 2 | *Ugp2* | 0.37 | 1.41 |
| 1433570_s_at | N(alpha)-acetyltransferase 35, NatC auxiliary subunit | *Naa35* | 0.61 | 1.41 |
| 1443583_at | protein phosphatase 4, regulatory subunit 1-like, pseudogene | *Ppp4r1l-ps* | 0.58 | 1.41 |
| 1451113_a_at | IK cytokine | *Ik* | 0.71 | 1.41 |
| 1451049_at | B-cell receptor-associated protein 31 | *Bcap31* | 0.61 | 1.41 |
| 1427526_at | FGFR1 oncogene partner 2 | *Fgfr1op2* | 0.69 | 1.41 |
| 1422797_at | roadblock domain containing 3 | *Robld3* | 0.77 | 1.41 |
| 1423651_at | iron-sulfur cluster assembly 1 homolog (S. cerevisiae) | *Isca1* | 0.70 | 1.41 |
| 1417429_at | flavin containing monooxygenase 1 | *Fmo1* | 0.69 | 1.41 |
| 1418005_at | succinate dehydrogenase complex, subunit B, iron sulfur (Ip) | *Sdhb* | 0.51 | 1.41 |
| 1451326_at | abhydrolase domain containing 14b | *Abhd14b* | 0.65 | 1.41 |
| 1452975_at | alanine-glyoxylate aminotransferase 2-like 1 | *Agxt2l1* | 0.11 | 1.41 |
| 1419103_a_at | abhydrolase domain containing 6 | *Abhd6* | 0.53 | 1.41 |
| 1416123_at | cyclin D2 | *Ccnd2* | 0.63 | 1.41 |
| 1425107_a_at | leukemia inhibitory factor receptor | *Lifr* | 0.44 | 1.41 |
| 1421922_at | SH3-domain binding protein 5 (BTK-associated) | *Sh3bp5* | 0.58 | 1.41 |
| 1434702_at | DDRGK domain containing 1 | *Ddrgk1* | 0.78 | 1.41 |
| 1437070_at | CDC14 cell division cycle 14 homolog B (S. cerevisiae) | *Cdc14b* | 0.43 | 1.41 |
| 1455405_at | proline-serine-threonine phosphatase-interacting protein 2 | *Pstpip2* | 0.60 | 1.41 |
| 1456155_x_at | fucosidase, alpha-L- 1, tissue | *Fuca1* | 0.65 | 1.41 |
| 1418765_at | T-cell immunoglobulin and mucin domain containing 2 | *Timd2* | 0.58 | 1.41 |
| 1421709_a_at | flavin containing monooxygenase 5 | *Fmo5* | 0.73 | 1.41 |
| 1417422_at | glycine N-methyltransferase | *Gnmt* | 0.66 | 1.41 |
| 1451046_at | zinc finger protein, multitype 1 | *Zfpm1* | 0.42 | 1.41 |
| 1438383_x_at | protein phosphatase 2 (formerly 2A), regulatory subunit A (PR 65), alpha isoform | *Ppp2r1a* | 0.67 | 1.41 |
| 1418168_at | zinc finger, CCHC domain containing 14 | *Zcchc14* | 0.50 | 1.40 |
| 1416005_at | protease (prosome, macropain) 26S subunit, ATPase 1 | *Psmc1* | 0.65 | 1.40 |
| 1450391_a_at | monoglyceride lipase | *Mgll* | 0.49 | 1.40 |
| 1418113_at | cytochrome P450, family 2, subfamily d, polypeptide 10 | *Cyp2d10* | 0.69 | 1.40 |
| 1418405_at | hepatocyte growth factor activator | *Hgfac* | 0.76 | 1.40 |
| 1424400_a_at | aldehyde dehydrogenase 1 family, member L1 | *Aldh1l1* | 0.54 | 1.40 |
| 1430526_a_at | SWI/SNF related, matrix associated, actin dependent regulator of chromatin, subfamily a, member 2 | *Smarca2* | 0.48 | 1.40 |
| 1415990_at | voltage-dependent anion channel 2 | *Vdac2* | 0.61 | 1.40 |
| 1421197_a_at | apoptotic chromatin condensation inducer 1 | *Acin1* | 0.70 | 1.40 |
| 1452532_x_at | carcinoembryonic antigen-related cell adhesion molecule 1 | *Ceacam1* | 0.65 | 1.40 |
| 1434997_at | cyclin-dependent kinase 19 | *Cdk19* | 0.55 | 1.40 |
| 1441945_s_at | abhydrolase domain containing 14A | *Abhd14a* | 0.75 | 1.40 |
| 1417882_at | solute carrier family 39 (zinc transporter), member 3 | *Slc39a3* | 0.80 | 1.40 |
| 1418760_at | retinol dehydrogenase 11 | *Rdh11* | 0.67 | 1.40 |
| 1419260_a_at | small nuclear ribonucleoprotein B | *Snrpb* | 0.58 | 1.40 |
| 1421605_a_at | aquaporin 9 | *Aqp9* | 0.71 | 1.40 |
| 1450086_at | glucocorticoid modulatory element binding protein 1 | *Gmeb1* | 0.51 | 1.40 |
| 1426535_at | protein O-glucosyltransferase 1 | *Poglut1* | 0.75 | 1.40 |
| 1432411_a_at | F-box and WD-40 domain protein 2 | *Fbxw2* | 0.59 | 1.40 |
| 1424948_x_at | histocompatibility 2, D region locus 1 | *H2-D1* | 0.45 | 1.40 |
| 1429474_at | prostaglandin reductase 2 | *Ptgr2* | 0.78 | 1.40 |
| 1443829_x_at | Coenzyme A synthase | *Coasy* | 0.71 | 1.40 |
| 1436830_at | MARVEL (membrane-associating) domain containing 1 | *Marveld1* | 0.50 | 1.40 |
| 1443783_x_at | histocompatibility 2, class II antigen A, alpha | *H2-Aa* | 0.67 | 1.40 |
| 1418134_at | solute carrier family 25, member 46 | *Slc25a46* | 0.59 | 1.40 |
| 1416877_a_at | mitochondrial ribosomal protein L51 | *Mrpl51* | 0.64 | 1.40 |
| 1451345_at | insulin-like growth factor binding protein 4 | *Igfbp4* | 0.72 | 1.40 |
| 1424408_at | LIM and senescent cell antigen like domains 2 | *Lims2* | 0.65 | 1.40 |
| 1436344_at | C2 calcium-dependent domain containing 2 | *C2cd2* | 0.55 | 1.40 |
| 1452341_at | enoyl Coenzyme A hydratase, short chain, 1, mitochondrial | *Echs1* | 0.67 | 1.40 |
| 1425937_a_at | hexamethylene bis-acetamide inducible 1 | *Hexim1* | 0.74 | 1.40 |
| 1450699_at | selenium binding protein 1 | *Selenbp1* | 0.65 | 1.40 |
| 1416984_at | mitochondrial ribosomal protein S18A | *Mrps18a* | 0.51 | 1.40 |
| 1426481_at | kelch-like 22 (Drosophila) | *Klhl22* | 0.80 | 1.40 |
| 1447837_x_at | polymerase (DNA directed), eta (RAD 30 related) | *Polh* | 0.78 | 1.40 |
| 1426257_a_at | seryl-aminoacyl-tRNA synthetase | *Sars* | 0.72 | 1.40 |
| 1434251_at | CCR4-NOT transcription complex, subunit 1 | *Cnot1* | 0.76 | 1.39 |
| 1419385_a_at | ubiquilin 1 | *Ubqln1* | 0.62 | 1.39 |
| 1450969_at | propionyl Coenzyme A carboxylase, beta polypeptide | *Pccb* | 0.66 | 1.39 |
| 1437592_x_at | coiled-coil domain containing 101 | *Ccdc101* | 0.67 | 1.39 |
| 1430527_a_at | ring finger protein 167 | *Rnf167* | 0.61 | 1.39 |
| 1438656_x_at | translocase of inner mitochondrial membrane 17b | *Timm17b* | 0.71 | 1.39 |
| 1438971_x_at | ubiquitin-conjugating enzyme E2H | *Ube2h* | 0.51 | 1.39 |
| 1419036_at | casein kinase 2, alpha 1 polypeptide | *Csnk2a1* | 0.58 | 1.39 |
| 1453155_at | transmembrane protein 50A | *Tmem50a* | 0.64 | 1.39 |
| 1418746_at | paroxysmal nonkinesiogenic dyskinesia | *Pnkd* | 0.57 | 1.39 |
| 1458268_s_at | insulin-like growth factor binding protein 3 | *Igfbp3* | 0.69 | 1.39 |
| 1416430_at | catalase | *Cat* | 0.47 | 1.39 |
| 1448921_a_at | mitochondrial ribosomal protein S9 | *Mrps9* | 0.69 | 1.39 |
| 1428070_at | synovial apoptosis inhibitor 1, synoviolin | *Syvn1* | 0.75 | 1.39 |
| 1435900_at | zinc finger and BTB domain containing 43 | *Zbtb43* | 0.78 | 1.39 |
| 1451285_at | fusion, derived from t(12;16) malignant liposarcoma (human) | *Fus* | 0.41 | 1.39 |
| 1434485_a_at | UDP-glucose pyrophosphorylase 2 | *Ugp2* | 0.35 | 1.39 |
| 1447278_at | centrosomal protein 164 | *Cep164* | 0.63 | 1.39 |
| 1427475_a_at | PDZ and LIM domain 5 | *Pdlim5* | 0.63 | 1.39 |
| 1428505_at | coiled-coil domain containing 90B | *Ccdc90b* | 0.70 | 1.39 |
| 1423030_at | valosin containing protein | *Vcp* | 0.70 | 1.39 |
| 1438097_at | RAB20, member RAS oncogene family | *Rab20* | 0.78 | 1.39 |
| 1416663_at | NADH dehydrogenase (ubiquinone) 1 alpha subcomplex, 9 | *Ndufa9* | 0.55 | 1.39 |
| 1448976_at | tuftelin interacting protein 11 | *Tfip11* | 0.80 | 1.39 |
| 1460333_at | DEAD (Asp-Glu-Ala-Asp) box polypeptide 59 | *Ddx59* | 0.47 | 1.39 |
| 1418844_at | asparagine-linked glycosylation 9 homolog (yeast, alpha 1,2 mannosyltransferase) | *Alg9* | 0.76 | 1.39 |
| 1449027_at | ras homolog gene family, member U | *Rhou* | 0.80 | 1.39 |
| 1420745_a_at | cyclin D-type binding-protein 1 | *Ccndbp1* | 0.61 | 1.39 |
| 1424505_at | required for meiotic nuclear division 1 homolog (S. cerevisiae) | *Rmnd1* | 0.55 | 1.39 |
| 1437343_x_at | ATPase family, AAA domain containing 3A | *Atad3a* | 0.61 | 1.39 |
| 1424036_at | PRP6 pre-mRNA splicing factor 6 homolog (yeast) | *Prpf6* | 0.55 | 1.39 |
| 1416172_at | pescadillo homolog 1, containing BRCT domain (zebrafish) | *Pes1* | 0.73 | 1.39 |
| 1451330_a_at | inositol polyphosphate-5-phosphatase B | *Inpp5b* | 0.67 | 1.39 |
| 1441445_at | period homolog 3 (Drosophila) | *Per3* | 0.63 | 1.39 |
| 1429272_a_at | apolipoprotein L 7a | *Apol7a* | 0.63 | 1.39 |
| 1429002_at | SNW domain containing 1 | *Snw1* | 0.75 | 1.39 |
| 1428160_at | NADH dehydrogenase (ubiquinone) 1, alpha/beta subcomplex, 1 | *Ndufab1* | 0.72 | 1.39 |
| 1430857_s_at | peroxisomal biogenesis factor 11 gamma | *Pex11c* | 0.66 | 1.38 |
| 1416618_at | protoporphyrinogen oxidase | *Ppox* | 0.59 | 1.38 |
| 1424628_a_at | NADH dehydrogenase (ubiquinone) flavoprotein 3 | *Ndufv3* | 0.65 | 1.38 |
| 1416772_at | carnitine palmitoyltransferase 2 | *Cpt2* | 0.52 | 1.38 |
| 1415770_at | WD repeat domain 6 | *Wdr6* | 0.57 | 1.38 |
| 1434801_x_at | solute carrier family 25 (mitochondrial carrier, adenine nucleotide translocator), member 5 | *Slc25a5* | 0.67 | 1.38 |
| 1424356_a_at | meteorin, glial cell differentiation regulator-like | *Metrnl* | 0.65 | 1.38 |
| 1427951_s_at | coiled-coil domain containing 28A | *Ccdc28a* | 0.61 | 1.38 |
| 1429971_at | thioredoxin reductase 2 | *Txnrd2* | 0.42 | 1.38 |
| 1424109_a_at | glyoxalase 1 | *Glo1* | 0.57 | 1.38 |
| 1429363_at | vacuolar protein sorting 37A (yeast) | *Vps37a* | 0.78 | 1.38 |
| 1428464_at | NADH dehydrogenase (ubiquinone) 1 alpha subcomplex, 3 | *Ndufa3* | 0.72 | 1.38 |
| 1417374_at | tubulin, alpha 4A | *Tuba4a* | 0.68 | 1.38 |
| 1450953_at | cytosolic iron-sulfur protein assembly 1 homolog (S. cerevisiae) | *Ciao1* | 0.75 | 1.38 |
| 1454828_at | G protein-coupled receptor 107 | *Gpr107* | 0.55 | 1.38 |
| 1424760_a_at | SET and MYND domain containing 2 | *Smyd2* | 0.63 | 1.38 |
| 1428689_at | trypsin domain containing 1 | *Tysnd1* | 0.77 | 1.38 |
| 1422578_at | citrate synthase | *Cs* | 0.45 | 1.38 |
| 1416538_at | sialic acid acetylesterase | *Siae* | 0.50 | 1.38 |
| 1455439_a_at | lectin, galactose binding, soluble 1 | *Lgals1* | 0.78 | 1.38 |
| 1420731_a_at | cysteine and glycine-rich protein 2 | *Csrp2* | 0.79 | 1.38 |
| 1447380_at | amidohydrolase domain containing 1 | *Amdhd1* | 0.47 | 1.38 |
| 1442878_at | peroxiredoxin 6 | *Prdx6* | 0.72 | 1.38 |
| 1425803_a_at | methyl-CpG binding domain protein 2 | *Mbd2* | 0.50 | 1.38 |
| 1448774_at | stomatin (Epb7.2)-like 2 | *Stoml2* | 0.77 | 1.38 |
| 1434517_at | WD repeat and FYVE domain containing 2 | *Wdfy2* | 0.66 | 1.38 |
| 1415775_at | retinoblastoma binding protein 7 | *Rbbp7* | 0.65 | 1.38 |
| 1434354_at | monoamine oxidase B | *Maob* | 0.54 | 1.38 |
| 1460544_at | N(alpha)-acetyltransferase 35, NatC auxiliary subunit | *Naa35* | 0.57 | 1.38 |
| 1455286_at | BTB (POZ) domain containing 1 | *Btbd1* | 0.62 | 1.38 |
| 1438922_x_at | solute carrier family 25 (mitochondrial carrier, adenine nucleotide translocator), member 5 | *Slc25a5* | 0.52 | 1.38 |
| 1420642_a_at | reactive oxygen species modulator 1 | *Romo1* | 0.74 | 1.38 |
| 1417265_s_at | coenzyme Q5 homolog, methyltransferase (yeast) | *Coq5* | 0.76 | 1.37 |
| 1417066_at | aarF domain containing kinase 3 | *Adck3* | 0.36 | 1.37 |
| 1419525_at | carbonic anhydrase 5a, mitochondrial | *Car5a* | 0.57 | 1.37 |
| 1416070_a_at | DEAD (Asp-Glu-Ala-Asp) box polypeptide 18 | *Ddx18* | 0.67 | 1.37 |
| 1456081_a_at | acetoacetyl-CoA synthetase | *Aacs* | 0.62 | 1.37 |
| 1452823_at | glutathione S-transferase kappa 1 | *Gstk1* | 0.44 | 1.37 |
| 1423849_a_at | CDC-like kinase 3 | *Clk3* | 0.44 | 1.37 |
| 1430896_s_at | nudix (nucleoside diphosphate linked moiety X)-type motif 7 | *Nudt7* | 0.39 | 1.37 |
| 1423736_a_at | dymeclin | *Dym* | 0.53 | 1.37 |
| 1422896_at | vesicle-associated membrane protein 4 | *Vamp4* | 0.62 | 1.37 |
| 1427889_at | spectrin alpha 2 | *Spna2* | 0.61 | 1.37 |
| 1419462_s_at | gene trap locus 3 | *Gtl3* | 0.55 | 1.37 |
| 1421835_at | microtubule-associated protein 7 | *Mtap7* | 0.71 | 1.37 |
| 1455446_x_at | acyl-Coenzyme A dehydrogenase, short/branched chain | *Acadsb* | 0.49 | 1.37 |
| 1422464_at | mitochondrial ribosomal protein L3 | *Mrpl3* | 0.72 | 1.37 |
| 1435669_at | zinc finger protein 532 | *Zfp532* | 0.59 | 1.37 |
| 1448318_at | perilipin 2 | *Plin2* | 0.63 | 1.37 |
| 1416339_a_at | protein kinase C substrate 80K-H | *Prkcsh* | 0.61 | 1.37 |
| 1432136_s_at | zinc finger, DHHC domain containing 4 | *Zdhhc4* | 0.72 | 1.37 |
| 1424138_at | rhomboid family 1 (Drosophila) | *Rhbdf1* | 0.60 | 1.37 |
| 1420624_a_at | vesicle-associated membrane protein 8 | *Vamp8* | 0.75 | 1.37 |
| 1429819_at | nicotinamide nucleotide adenylyltransferase 1 | *Nmnat1* | 0.57 | 1.37 |
| 1416604_at | cytochrome c-1 | *Cyc1* | 0.73 | 1.37 |
| 1433611_s_at | BUD31 homolog (yeast) | *Bud31* | 0.70 | 1.37 |
| 1438557_x_at | aspartyl aminopeptidase | *Dnpep* | 0.71 | 1.37 |
| 1455174_at | ribosomal protein S19 binding protein 1 | *Rps19bp1* | 0.57 | 1.37 |
| 1448277_at | polymerase (DNA directed), delta 2, regulatory subunit | *Pold2* | 0.64 | 1.37 |
| 1423299_at | thioredoxin-like 1 | *Txnl1* | 0.69 | 1.37 |
| 1438130_at | TAF15 RNA polymerase II, TATA box binding protein (TBP)-associated factor | *Taf15* | 0.69 | 1.37 |
| 1425830_a_at | cyclin-dependent kinase 2 interacting protein | *Cinp* | 0.63 | 1.37 |
| 1423902_s_at | Rho guanine nucleotide exchange factor (GEF) 12 | *Arhgef12* | 0.76 | 1.37 |
| 1424165_a_at | SHANK-associated RH domain interacting protein | *Sharpin* | 0.72 | 1.37 |
| 1415966_a_at | NADH dehydrogenase (ubiquinone) flavoprotein 1 | *Ndufv1* | 0.66 | 1.37 |
| 1418117_at | NADH dehydrogenase (ubiquinone) Fe-S protein 4 | *Ndufs4* | 0.70 | 1.37 |
| 1424436_at | phosphoribosylglycinamide formyltransferase | *Gart* | 0.59 | 1.37 |
| 1434057_at | NADH dehydrogenase (ubiquinone) 1 beta subcomplex, 6 | *Ndufb6* | 0.66 | 1.37 |
| 1428406_s_at | host cell factor C1 regulator 1 (XPO1-dependent) | *Hcfc1r1* | 0.62 | 1.37 |
| 1447780_x_at | Tu translation elongation factor, mitochondrial | *Tufm* | 0.58 | 1.36 |
| 1434516_at | phosphoseryl-tRNA kinase | *Pstk* | 0.59 | 1.36 |
| 1429477_at | non-SMC condensin II complex, subunit H2 | *Ncaph2* | 0.60 | 1.36 |
| 1456663_x_at | TM2 domain containing 2 | *Tm2d2* | 0.76 | 1.36 |
| 1416254_a_at | vacuolar protein sorting 16 (yeast) | *Vps16* | 0.46 | 1.36 |
| 1416372_at | phosphatidylserine synthase 1 | *Ptdss1* | 0.73 | 1.36 |
| 1416068_at | lysyl-tRNA synthetase | *Kars* | 0.76 | 1.36 |
| 1415707_at | anaphase promoting complex subunit 2 | *Anapc2* | 0.64 | 1.36 |
| 1445787_at | coiled-coil domain containing 162 | *Ccdc162* | 0.64 | 1.36 |
| 1438239_at | midline 1 | *Mid1* | 0.71 | 1.36 |
| 1455800_x_at | sorting and assembly machinery component 50 homolog (S. cerevisiae) | *Samm50* | 0.65 | 1.36 |
| 1448266_at | endothelial differentiation-related factor 1 | *Edf1* | 0.72 | 1.36 |
| 1423701_at | Coenzyme A synthase | *Coasy* | 0.75 | 1.36 |
| 1453725_a_at | mitchondrial ribosomal protein S7 | *Mrps7* | 0.75 | 1.36 |
| 1436516_at | abhydrolase domain containing 13 | *Abhd13* | 0.57 | 1.36 |
| 1449112_at | solute carrier family 27 (fatty acid transporter), member 5 | *Slc27a5* | 0.61 | 1.36 |
| 1453358_s_at | antagonist of mitotic exit network 1 homolog (S. cerevisiae) | *Amn1* | 0.79 | 1.36 |
| 1416175_a_at | voltage-dependent anion channel 3 | *Vdac3* | 0.73 | 1.36 |
| 1433563_s_at | Der1-like domain family, member 1 | *Derl1* | 0.72 | 1.36 |
| 1428276_at | abhydrolase domain containing 13 | *Abhd13* | 0.41 | 1.36 |
| 1417602_at | period homolog 2 (Drosophila) | *Per2* | 0.76 | 1.36 |
| 1416665_at | demethyl-Q 7 | *Coq7* | 0.69 | 1.36 |
| 1451971_at | cullin 4A | *Cul4a* | 0.59 | 1.36 |
| 1448499_a_at | epoxide hydrolase 2, cytoplasmic | *Ephx2* | 0.38 | 1.36 |
| 1423031_at | macrophage erythroblast attacher | *Maea* | 0.75 | 1.36 |
| 1458384_at | bolA-like 3 (E. coli) | *Bola3* | 0.76 | 1.35 |
| 1456083_x_at | eukaryotic translation initiation factor 3, subunit C | *Eif3c* | 0.62 | 1.35 |
| 1436959_x_at | nasal embryonic LHRH factor | *Nelf* | 0.59 | 1.35 |
| 1424656_s_at | ubiquitin specific peptidase 19 | *Usp19* | 0.54 | 1.35 |
| 1431784_a_at | ribosome production factor 1 homolog (S. cerevisiae) | *Rpf1* | 0.66 | 1.35 |
| 1416565_at | cytochrome c oxidase, subunit VIb polypeptide 1 | *Cox6b1* | 0.62 | 1.35 |
| 1416978_at | Fc receptor, IgG, alpha chain transporter | *Fcgrt* | 0.58 | 1.35 |
| 1424506_at | zinc finger protein 768 | *Zfp768* | 0.77 | 1.35 |
| 1426367_at | calcium binding protein 39-like | *Cab39l* | 0.79 | 1.35 |
| 1423398_at | TAF12 RNA polymerase II, TATA box binding protein (TBP)-associated factor | *Taf12* | 0.72 | 1.35 |
| 1417742_a_at | DNA methyltransferase 1-associated protein 1 | *Dmap1* | 0.50 | 1.35 |
| 1434500_at | tweety homolog 2 (Drosophila) | *Ttyh2* | 0.76 | 1.35 |
| 1426548_a_at | ATP binding domain 4 | *Atpbd4* | 0.75 | 1.35 |
| 1436992_x_at | voltage-dependent anion channel 1 | *Vdac1* | 0.73 | 1.35 |
| 1427342_at | FAST kinase domains 1 | *Fastkd1* | 0.54 | 1.35 |
| 1424016_at | alpha- and gamma-adaptin binding protein | *Aagab* | 0.54 | 1.35 |
| 1436343_at | chromodomain helicase DNA binding protein 4 | *Chd4* | 0.56 | 1.35 |
| 1418089_at | syntaxin 8 | *Stx8* | 0.63 | 1.35 |
| 1426815_s_at | SEC16 homolog A (S. cerevisiae) | *Sec16a* | 0.59 | 1.35 |
| 1438317_a_at | endonuclease G | *Endog* | 0.77 | 1.35 |
| 1452209_at | plakophilin 4 | *Pkp4* | 0.66 | 1.35 |
| 1418238_at | isovaleryl coenzyme A dehydrogenase | *Ivd* | 0.45 | 1.35 |
| 1426261_s_at | UDP glucuronosyltransferase 1 family, polypeptide A1 | *Ugt1a1* | 0.65 | 1.35 |
| 1438249_at | ubiquitin specific peptidase 7 | *Usp7* | 0.78 | 1.35 |
| 1426264_at | dihydrolipoamide S-acetyltransferase (E2 component of pyruvate dehydrogenase complex) | *Dlat* | 0.62 | 1.35 |
| 1423817_s_at | unconventional SNARE in the ER 1 homolog (S. cerevisiae) | *Use1* | 0.77 | 1.35 |
| 1452853_at | sedoheptulokinase | *Shpk* | 0.67 | 1.35 |
| 1421817_at | glutathione reductase | *Gsr* | 0.60 | 1.35 |
| 1424353_at | leucine-rich PPR-motif containing | *Lrpprc* | 0.59 | 1.35 |
| 1454738_x_at | peroxisomal biogenesis factor 6 | *Pex6* | 0.66 | 1.35 |
| 1441380_at | tetratricopeptide repeat domain 39C | *Ttc39c* | 0.41 | 1.35 |
| 1438390_s_at | pituitary tumor-transforming gene 1 | *Pttg1* | 0.56 | 1.35 |
| 1455011_at | StAR-related lipid transfer (START) domain containing 4 | *Stard4* | 0.56 | 1.35 |
| 1450715_at | cytochrome P450, family 1, subfamily a, polypeptide 2 | *Cyp1a2* | 0.28 | 1.35 |
| 1425314_at | G protein-coupled receptor 98 | *Gpr98* | 0.41 | 1.35 |
| 1435003_at | phosphatidylinositol 4-kinase, catalytic, alpha polypeptide | *Pi4ka* | 0.78 | 1.35 |
| 1418253_a_at | heat shock protein 4 like | *Hspa4l* | 0.66 | 1.35 |
| 1415683_at | N-myristoyltransferase 1 | *Nmt1* | 0.71 | 1.35 |
| 1417912_at | transmembrane protein 93 | *Tmem93* | 0.78 | 1.35 |
| 1416632_at | malic enzyme 1, NADP(+)-dependent, cytosolic | *Me1* | 0.17 | 1.35 |
| 1450846_at | basic leucine zipper and W2 domains 1 | *Bzw1* | 0.80 | 1.35 |
| 1424867_a_at | glycine-N-acyltransferase | *Glyat* | 0.53 | 1.35 |
| 1415991_a_at | kelch domain containing 3 | *Klhdc3* | 0.66 | 1.34 |
| 1418649_at | EGL nine homolog 3 (C. elegans) | *Egln3* | 0.64 | 1.34 |
| 1426679_at | zinc finger protein 706 | *Zfp706* | 0.78 | 1.34 |
| 1424504_at | RAB22A, member RAS oncogene family | *Rab22a* | 0.44 | 1.34 |
| 1426654_at | zinc finger, C3HC type 1 | *Zc3hc1* | 0.72 | 1.34 |
| 1417785_at | chemokine (C-X-C motif) ligand 1 | *Cxcl1* | 0.66 | 1.34 |
| 1449067_at | solute carrier family 2 (facilitated glucose transporter), member 2 | *Slc2a2* | 0.41 | 1.34 |
| 1418764_a_at | bisphosphate 3'-nucleotidase 1 | *Bpnt1* | 0.55 | 1.34 |
| 1423686_a_at | proline rich 13 | *Prr13* | 0.74 | 1.34 |
| 1452311_at | dimethylglycine dehydrogenase precursor | *Dmgdh* | 0.49 | 1.34 |
| 1448131_at | mitofusin 2 | *Mfn2* | 0.45 | 1.34 |
| 1448368_at | dynactin 6 | *Dctn6* | 0.69 | 1.34 |
| 1416207_at | tafazzin | *Taz* | 0.56 | 1.34 |
| 1428447_at | transmembrane protein 14A | *Tmem14a* | 0.49 | 1.34 |
| 1416210_at | IMP3, U3 small nucleolar ribonucleoprotein, homolog (yeast) | *Imp3* | 0.67 | 1.34 |
| 1416090_at | pyruvate dehydrogenase (lipoamide) beta | *Pdhb* | 0.69 | 1.34 |
| 1452173_at | hydroxyacyl-Coenzyme A dehydrogenase/3-ketoacyl-Coenzyme A thiolase/enoyl-Coenzyme A hydratase (trifunctional protein), alpha subunit | *Hadha* | 0.73 | 1.34 |
| 1418248_at | galactosidase, alpha | *Gla* | 0.75 | 1.34 |
| 1417629_at | proline dehydrogenase | *Prodh* | 0.48 | 1.34 |
| 1449692_at | protein kinase C, zeta | *Prkcz* | 0.66 | 1.34 |
| 1450561_a_at | surfeit gene 1 | *Surf1* | 0.70 | 1.34 |
| 1455457_at | cytochrome P450, family 2, subfamily c, polypeptide 54 | *Cyp2c54* | 0.58 | 1.34 |
| 1435613_x_at | cytochrome c oxidase, subunit Vb | *Cox5b* | 0.58 | 1.34 |
| 1452434_s_at | DiGeorge syndrome critical region gene 6 | *Dgcr6* | 0.72 | 1.34 |
| 1426602_at | v-raf murine sarcoma 3611 viral oncogene homolog | *Araf* | 0.76 | 1.34 |
| 1417094_at | acyl-CoA thioesterase 7 | *Acot7* | 0.54 | 1.34 |
| 1436736_x_at | DNA segment, human D4S114 | *D0H4S114* | 0.40 | 1.34 |
| 1450127_a_at | glucagon receptor | *Gcgr* | 0.79 | 1.34 |
| 1424421_at | RFad1, flavin adenine dinucleotide synthetase, homolog (yeast) | *Flad1* | 0.70 | 1.33 |
| 1448274_at | complement component 1, q subcomponent binding protein | *C1qbp* | 0.67 | 1.33 |
| 1424973_at | cytochrome P450, family 3, subfamily a, polypeptide 25 | *Cyp3a25* | 0.70 | 1.33 |
| 1416291_at | proteasome (prosome, macropain) 26S subunit, ATPase, 4 | *Psmc4* | 0.71 | 1.33 |
| 1448836_s_at | GPN-loop GTPase 2 | *Gpn2* | 0.67 | 1.33 |
| 1456194_a_at | Parkinson disease (autosomal recessive, early onset) 7 | *Park7* | 0.58 | 1.33 |
| 1460320_at | beclin 1, autophagy related | *Becn1* | 0.78 | 1.33 |
| 1430889_a_at | thiopurine methyltransferase | *Tpmt* | 0.65 | 1.33 |
| 1451742_a_at | UDP-glucose pyrophosphorylase 2 | *Ugp2* | 0.37 | 1.33 |
| 1451552_at | lipoyltransferase 1 | *Lipt1* | 0.36 | 1.33 |
| 1431302_a_at | nudix (nucleoside diphosphate linked moiety X)-type motif 7 | *Nudt7* | 0.55 | 1.33 |
| 1426977_at | ubiquitin specific peptidase 47 | *Usp47* | 0.62 | 1.33 |
| 1426236_a_at | glutamate-ammonia ligase (glutamine synthetase) | *Glul* | 0.60 | 1.33 |
| 1438545_at | solute carrier family 25 (mitochondrial carrier, adenine nucleotide translocator), member 5 | *Slc25a5* | 0.64 | 1.33 |
| 1428859_at | polyamine oxidase (exo-N4-amino) | *Paox* | 0.73 | 1.33 |
| 1449183_at | catechol-O-methyltransferase 1 | *Comt1* | 0.64 | 1.33 |
| 1438934_x_at | sema domain, immunoglobulin domain (Ig), transmembrane domain (TM) and short cytoplasmic domain, (semaphorin) 4A | *Sema4a* | 0.67 | 1.33 |
| 1448181_at | Kruppel-like factor 15 | *Klf15* | 0.66 | 1.33 |
| 1424282_at | PET112-like (yeast) | *Pet112l* | 0.66 | 1.33 |
| 1429534_a_at | inner membrane protein, mitochondrial | *Immt* | 0.62 | 1.33 |
| 1439185_x_at | mitochondrial antiviral signaling protein | *Mavs* | 0.75 | 1.33 |
| 1455285_at | solute carrier family 31, member 1 | *Slc31a1* | 0.78 | 1.33 |
| 1426804_at | SWI/SNF related, matrix associated, actin dependent regulator of chromatin, subfamily a, member 4 | *Smarca4* | 0.77 | 1.33 |
| 1451172_at | transformation related protein 63 regulated like | *Tprgl* | 0.64 | 1.33 |
| 1416954_at | solute carrier family 25 (mitochondrial carrier, dicarboxylate transporter), member 10 | *Slc25a10* | 0.65 | 1.33 |
| 1434866_x_at | carnitine palmitoyltransferase 1a, liver | *Cpt1a* | 0.40 | 1.33 |
| 1423588_at | actin related protein 2/3 complex, subunit 4 | *Arpc4* | 0.71 | 1.33 |
| 1451675_a_at | aminolevulinic acid synthase 2, erythroid | *Alas2* | 0.60 | 1.33 |
| 1416027_at | programmed cell death 6 | *Pdcd6* | 0.71 | 1.33 |
| 1417418_s_at | cytochrome c oxidase, subunit VI a, polypeptide 1 | *Cox6a1* | 0.77 | 1.33 |
| 1436704_x_at | methylenetetrahydrofolate dehydrogenase (NADP+ dependent), methenyltetrahydrofolate cyclohydrolase, formyltetrahydrofolate synthase | *Mthfd1* | 0.54 | 1.33 |
| 1454929_s_at | scaffold attachment factor B | *Safb* | 0.73 | 1.33 |
| 1416494_at | NADH dehydrogenase (ubiquinone) Fe-S protein 5 | *Ndufs5* | 0.51 | 1.33 |
| 1417809_at | solute carrier family 22 (organic cation transporter), member 18 | *Slc22a18* | 0.62 | 1.32 |
| 1421009_at | radical S-adenosyl methionine domain containing 2 | *Rsad2* | 0.80 | 1.32 |
| 1448233_at | prion protein | *Prnp* | 0.53 | 1.32 |
| 1428302_at | mitochondrial ribosomal protein L48 | *Mrpl48* | 0.76 | 1.32 |
| 1417316_at | acyl-CoA thioesterase 13 | *Acot13* | 0.60 | 1.32 |
| 1452601_a_at | acyl-Coenzyme A binding domain containing 6 | *Acbd6* | 0.53 | 1.32 |
| 1433912_at | TIP41, TOR signalling pathway regulator-like (S. cerevisiae) | *Tiprl* | 0.67 | 1.32 |
| 1447915_x_at | transmembrane protein 204 | *Tmem204* | 0.73 | 1.32 |
| 1425073_at | pleckstrin homology domain containing, family G (with RhoGef domain) member 6 | *Plekhg6* | 0.48 | 1.32 |
| 1452673_at | RAN binding protein 3 | *Ranbp3* | 0.68 | 1.32 |
| 1426688_at | succinate dehydrogenase complex, subunit A, flavoprotein (Fp) | *Sdha* | 0.46 | 1.32 |
| 1421821_at | low density lipoprotein receptor | *Ldlr* | 0.66 | 1.32 |
| 1437405_a_at | insulin-like growth factor binding protein 4 | *Igfbp4* | 0.78 | 1.32 |
| 1417559_at | sideroflexin 1 | *Sfxn1* | 0.78 | 1.32 |
| 1437379_x_at | TNF receptor-associated protein 1 | *Trap1* | 0.65 | 1.32 |
| 1434053_x_at | ATP synthase, H+ transporting, mitochondrial F1F0 complex, subunit e | *Atp5k* | 0.74 | 1.32 |
| 1424576_s_at | cytochrome P450, family 2, subfamily c, polypeptide 44 | *Cyp2c44* | 0.50 | 1.32 |
| 1452111_at | mitochondrial ribosomal protein S35 | *Mrps35* | 0.64 | 1.32 |
| 1433916_at | vesicle-associated membrane protein 3 | *Vamp3* | 0.78 | 1.32 |
| 1418249_at | calcitonin gene-related peptide-receptor component protein | *Crcp* | 0.75 | 1.32 |
| 1450112_a_at | growth arrest specific 2 | *Gas2* | 0.73 | 1.32 |
| 1426716_at | tudor domain containing 7 | *Tdrd7* | 0.72 | 1.32 |
| 1448722_s_at | phosphopantothenoylcysteine synthetase | *Ppcs* | 0.78 | 1.32 |
| 1429453_a_at | mitochondrial ribosomal protein L55 | *Mrpl55* | 0.70 | 1.32 |
| 1455806_x_at | NADH dehydrogenase (ubiquinone) 1 alpha subcomplex, 12 | *Ndufa12* | 0.60 | 1.32 |
| 1428737_s_at | GRAM domain containing 3 | *Gramd3* | 0.44 | 1.32 |
| 1448717_at | glutaryl-Coenzyme A dehydrogenase | *Gcdh* | 0.69 | 1.32 |
| 1460323_at | threonyl-tRNA synthetase | *Tars* | 0.73 | 1.32 |
| 1415890_at | 3'-phosphoadenosine 5'-phosphosulfate synthase 1 | *Papss1* | 0.62 | 1.32 |
| 1422966_a_at | chemokine (C-X-C motif) ligand 10 | *Cxcl10* | 0.57 | 1.32 |
| 1425195_a_at | acetyl-Coenzyme A acetyltransferase 2 | *Acat2* | 0.56 | 1.31 |
| 1419393_at | ATP-binding cassette, sub-family G (WHITE), member 5 | *Abcg5* | 0.67 | 1.31 |
| 1455655_a_at | TAR DNA binding protein | *Tardbp* | 0.78 | 1.31 |
| 1449818_at | ATP-binding cassette, sub-family B (MDR/TAP), member 4 | *Abcb4* | 0.53 | 1.31 |
| 1423244_at | cytochrome P450, family 2, subfamily c, polypeptide 68 | *Cyp2c68* | 0.61 | 1.31 |
| 1428141_at | golgi associated, gamma adaptin ear containing, ARF binding protein 2 | *Gga2* | 0.60 | 1.31 |
| 1419112_at | nemo like kinase | *Nlk* | 0.67 | 1.31 |
| 1423953_at | CDK5 regulatory subunit associated protein 1-like 1 | *Cdkal1* | 0.72 | 1.31 |
| 1455112_at | apoptosis-inducing factor, mitochondrion-associated 2 | *Aifm2* | 0.78 | 1.31 |
| 1449486_at | carboxylesterase 1G | *Ces1g* | 0.69 | 1.31 |
| 1425021_a_at | peroxisomal biogenesis factor 16 | *Pex16* | 0.59 | 1.31 |
| 1425678_a_at | SNF related kinase | *Snrk* | 0.60 | 1.31 |
| 1416247_at | dynactin 3 | *Dctn3* | 0.69 | 1.31 |
| 1451714_a_at | mitogen-activated protein kinase kinase 3 | *Map2k3* | 0.78 | 1.31 |
| 1436342_a_at | UBX domain protein 1 | *Ubxn1* | 0.66 | 1.31 |
| 1419663_at | osteoglycin | *Ogn* | 0.58 | 1.31 |
| 1433546_at | glucosamine (N-acetyl)-6-sulfatase | *Gns* | 0.61 | 1.31 |
| 1451274_at | oxoglutarate dehydrogenase (lipoamide) | *Ogdh* | 0.57 | 1.31 |
| 1436757_a_at | cytochrome c oxidase, subunit VIb polypeptide 1 | *Cox6b1* | 0.65 | 1.31 |
| 1433984_a_at | malate dehydrogenase 2, NAD (mitochondrial) | *Mdh2* | 0.77 | 1.31 |
| 1433975_at | cyclin-dependent kinase 10 | *Cdk10* | 0.67 | 1.31 |
| 1427746_x_at | histocompatibility 2, K1, K region | *H2-K1* | 0.76 | 1.31 |
| 1416284_at | mitochondrial ribosomal protein L28 | *Mrpl28* | 0.74 | 1.31 |
| 1448133_at | NMD3 homolog (S. cerevisiae) | *Nmd3* | 0.56 | 1.31 |
| 1454647_at | acyl-Coenzyme A dehydrogenase family, member 11 | *Acad11* | 0.58 | 1.31 |
| 1428950_s_at | nucleolar protein 8 | *Nol8* | 0.79 | 1.31 |
| 1419557_a_at | transmembrane protein 9 | *Tmem9* | 0.72 | 1.31 |
| 1428676_at | transmembrane serine protease 6 | *Tmprss6* | 0.74 | 1.31 |
| 1415785_a_at | chaperonin containing Tcp1, subunit 8 (theta) | *Cct8* | 0.74 | 1.31 |
| 1417199_at | transmembrane protein 183A | *Tmem183a* | 0.71 | 1.31 |
| 1438170_x_at | adhesion regulating molecule 1 | *Adrm1* | 0.48 | 1.31 |
| 1418077_at | tripartite motif-containing 21 | *Trim21* | 0.80 | 1.31 |
| 1441019_at | F-box protein 3 | *Fbxo3* | 0.73 | 1.31 |
| 1433448_at | solute carrier family 25, member 44 | *Slc25a44* | 0.72 | 1.31 |
| 1434916_at | vitamin K epoxide reductase complex, subunit 1-like 1 | *Vkorc1l1* | 0.63 | 1.31 |
| 1449125_at | tumor necrosis factor, alpha-induced protein 8-like 1 | *Tnfaip8l1* | 0.52 | 1.31 |
| 1418325_at | selenophosphate synthetase 2 | *Sephs2* | 0.63 | 1.31 |
| 1433506_at | leucine rich repeat containing 8D | *Lrrc8d* | 0.55 | 1.31 |
| 1455854_a_at | slingshot homolog 1 (Drosophila) | *Ssh1* | 0.65 | 1.31 |
| 1451267_at | SHANK-associated RH domain interacting protein | *Sharpin* | 0.69 | 1.31 |
| 1437749_s_at | mitochondrial ribosomal protein L9 | *Mrpl9* | 0.64 | 1.31 |
| 1450522_a_at | H1 histone family, member 0 | *H1f0* | 0.76 | 1.31 |
| 1435452_at | transmembrane protein 20 | *Tmem20* | 0.67 | 1.30 |
| 1428247_at | vacuolar protein sorting 26 homolog B (yeast) | *Vps26b* | 0.68 | 1.30 |
| 1448482_at | solute carrier family 39 (metal ion transporter), member 8 | *Slc39a8* | 0.41 | 1.30 |
| 1448154_at | N-myc downstream regulated gene 2 | *Ndrg2* | 0.65 | 1.30 |
| 1450261_a_at | solute carrier family 10 (sodium/bile acid cotransporter family), member 1 | *Slc10a1* | 0.39 | 1.30 |
| 1451042_a_at | myc induced nuclear antigen | *Mina* | 0.61 | 1.30 |
| 1423533_a_at | ras homolog gene family, member T1 | *Rhot1* | 0.72 | 1.30 |
| 1423680_at | fatty acid desaturase 1 | *Fads1* | 0.70 | 1.30 |
| 1431609_a_at | acid phosphatase 5, tartrate resistant | *Acp5* | 0.79 | 1.30 |
| 1454840_at | methylcrotonoyl-Coenzyme A carboxylase 2 (beta) | *Mccc2* | 0.63 | 1.30 |
| 1423896_a_at | ring finger protein 187 | *Rnf187* | 0.79 | 1.30 |
| 1455985_x_at | serine hydroxymethyltransferase 2 (mitochondrial) | *Shmt2* | 0.67 | 1.30 |
| 1417473_a_at | phosphopantothenoylcysteine synthetase | *Ppcs* | 0.78 | 1.30 |
| 1423717_at | adenylate kinase 3 | *Ak3* | 0.73 | 1.30 |
| 1419193_a_at | glia maturation factor, gamma | *Gmfg* | 0.74 | 1.30 |
| 1451455_at | threonine synthase-like 2 (bacterial) | *Thnsl2* | 0.69 | 1.30 |
| 1451405_at | propionyl-Coenzyme A carboxylase, alpha polypeptide | *Pcca* | 0.54 | 1.30 |
| 1432416_a_at | nucleophosmin 1 | *Npm1* | 0.71 | 1.30 |
| 1428707_at | parathymosin | *Ptms* | 0.63 | 1.30 |
| 1423187_at | gamma-aminobutyric acid (GABA) A receptor-associated protein-like 2 | *Gabarapl2* | 0.69 | 1.30 |
| 1429942_at | glucocorticoid modulatory element binding protein 1 | *Gmeb1* | 0.76 | 1.30 |
| 1425688_a_at | dihydropyrimidinase | *Dpys* | 0.51 | 1.30 |
| 1448819_at | eukaryotic translation initiation factor 2, subunit 2 (beta) | *Eif2s2* | 0.76 | 1.30 |
| 1434338_at | RGP1 retrograde golgi transport homolog (S. cerevisiae) | *Rgp1* | 0.77 | 1.30 |
| 1437237_x_at | zinc finger protein 110 | *Zfp110* | 0.65 | 1.30 |
| 1419453_at | ubiquitin carboxyl-terminal esterase L5 | *Uchl5* | 0.63 | 1.30 |
| 1429160_at | mitochondrial translational initiation factor 3 | *Mtif3* | 0.70 | 1.30 |
| 1427908_at | BCL2/adenovirus E1B interacting protein 1 | *Bnip1* | 0.70 | 1.30 |
| 1417715_a_at | glutamate oxaloacetate transaminase 2, mitochondrial | *Got2* | 0.74 | 1.30 |
| 1450860_at | leucine aminopeptidase 3 | *Lap3* | 0.74 | 1.30 |
| 1429131_at | ubiquitin-conjugating enzyme E2 variant 2 | *Ube2v2* | 0.79 | 1.30 |
| 1416915_at | mutS homolog 6 (E. coli) | *Msh6* | 0.78 | 1.30 |
| 1415836_at | aldehyde dehydrogenase 18 family, member A1 | *Aldh18a1* | 0.65 | 1.29 |
| 1424456_at | poliovirus receptor-related 2 | *Pvrl2* | 0.58 | 1.29 |
| 1438727_at | DDB1 and CUL4 associated factor 10 | *Dcaf10* | 0.54 | 1.29 |
| 1448897_at | makorin, ring finger protein, 2 | *Mkrn2* | 0.70 | 1.29 |
| 1424785_at | angiopoietin-like 6 | *Angptl6* | 0.69 | 1.29 |
| 1426118_a_at | translocase of outer mitochondrial membrane 40 homolog (yeast) | *Tomm40* | 0.72 | 1.29 |
| 1415685_at | mitochondrial translational initiation factor 2 | *Mtif2* | 0.74 | 1.29 |
| 1417693_a_at | growth factor receptor bound protein 2-associated protein 1 | *Gab1* | 0.65 | 1.29 |
| 1448753_at | signal recognition particle 9 | *Srp9* | 0.74 | 1.29 |
| 1427929_a_at | pyridoxal (pyridoxine, vitamin B6) kinase | *Pdxk* | 0.68 | 1.29 |
| 1426567_a_at | PQ loop repeat containing 1 | *Pqlc1* | 0.70 | 1.29 |
| 1417288_at | pleckstrin homology domain-containing, family A (phosphoinositide binding specific) member 2 | *Plekha2* | 0.64 | 1.29 |
| 1423784_at | glycyl-tRNA synthetase | *Gars* | 0.71 | 1.29 |
| 1440178_x_at | zeta-chain (TCR) associated protein kinase | *Zap70* | 0.73 | 1.29 |
| 1434750_at | exocyst complex component 3 | *Exoc3* | 0.73 | 1.29 |
| 1439482_at | UHRF1 (ICBP90) binding protein 1 | *Uhrf1bp1* | 0.67 | 1.29 |
| 1419363_a_at | mitochondrial ribosomal protein L35 | *Mrpl35* | 0.75 | 1.29 |
| 1416478_a_at | malate dehydrogenase 2, NAD (mitochondrial) | *Mdh2* | 0.68 | 1.29 |
| 1455095_at | histone cluster 2, H2be | *Hist2h2be* | 0.58 | 1.29 |
| 1455454_at | aldo-keto reductase family 1, member C19 | *Akr1c19* | 0.53 | 1.29 |
| 1428322_a_at | NADH dehydrogenase (ubiquinone) 1 beta subcomplex, 10 | *Ndufb10* | 0.60 | 1.29 |
| 1423495_at | 2-4-dienoyl-Coenzyme A reductase 2, peroxisomal | *Decr2* | 0.55 | 1.29 |
| 1424489_a_at | tRNA isopentenyltransferase 1 | *Trit1* | 0.69 | 1.29 |
| 1422526_at | acyl-CoA synthetase long-chain family member 1 | *Acsl1* | 0.31 | 1.29 |
| 1434454_at | adenylate cyclase 9 | *Adcy9* | 0.62 | 1.29 |
| 1458097_at | Cobl-like 1 | *Cobll1* | 0.80 | 1.29 |
| 1453065_at | aldhehyde dehydrogenase family 5, subfamily A1 | *Aldh5a1* | 0.51 | 1.29 |
| 1419367_at | 2,4-dienoyl CoA reductase 1, mitochondrial | *Decr1* | 0.64 | 1.29 |
| 1448657_a_at | DnaJ (Hsp40) homolog, subfamily B, member 2 | *Dnajb2* | 0.76 | 1.29 |
| 1426669_at | calcineurin-like phosphoesterase domain containing 1 | *Cpped1* | 0.79 | 1.29 |
| 1448343_a_at | neighbor of Brca1 gene 1 | *Nbr1* | 0.67 | 1.29 |
| 1448399_at | Tax1 (human T-cell leukemia virus type I) binding protein 1 | *Tax1bp1* | 0.64 | 1.29 |
| 1423196_at | neural precursor cell expressed, developmentally down-regulated gene 1 | *Nedd1* | 0.70 | 1.29 |
| 1438980_x_at | peptidase M20 domain containing 1 | *Pm20d1* | 0.51 | 1.29 |
| 1424389_at | nucleoporin like 1 | *Nupl1* | 0.71 | 1.29 |
| 1455741_a_at | endothelin converting enzyme 1 | *Ece1* | 0.74 | 1.29 |
| 1417189_at | proteasome (prosome, macropain) 28 subunit, beta | *Psme2* | 0.77 | 1.29 |
| 1450898_at | hippocampus abundant gene transcript 1 | *Hiat1* | 0.68 | 1.29 |
| 1421097_at | endonuclease G | *Endog* | 0.71 | 1.28 |
| 1433729_x_at | peptidase (mitochondrial processing) beta | *Pmpcb* | 0.77 | 1.28 |
| 1454716_x_at | cytochrome c oxidase, subunit Vb | *Cox5b* | 0.60 | 1.28 |
| 1428214_at | translocase of outer mitochondrial membrane 7 homolog (yeast) | *Tomm7* | 0.69 | 1.28 |
| 1416389_a_at | regulator of chromosome condensation (RCC1) and BTB (POZ) domain containing protein 2 | *Rcbtb2* | 0.68 | 1.28 |
| 1434145_s_at | serine hydrolase-like | *Serhl* | 0.73 | 1.28 |
| 1451754_a_at | WD repeat domain 45 | *Wdr45* | 0.66 | 1.28 |
| 1460216_at | acyl-Coenzyme A dehydrogenase, short chain | *Acads* | 0.73 | 1.28 |
| 1416059_at | SEC23B (S. cerevisiae) | *Sec23b* | 0.75 | 1.28 |
| 1451339_at | sulfite oxidase | *Suox* | 0.57 | 1.28 |
| 1460396_at | DEAD (Asp-Glu-Ala-Asp) box polypeptide 54 | *Ddx54* | 0.66 | 1.28 |
| 1437666_x_at | ubiquitin C | *Ubc* | 0.61 | 1.28 |
| 1427011_a_at | LanC (bacterial lantibiotic synthetase component C)-like 1 | *Lancl1* | 0.72 | 1.28 |
| 1454603_a_at | CCR4-NOT transcription complex, subunit 2 | *Cnot2* | 0.77 | 1.28 |
| 1433660_at | tubulin, gamma complex associated protein 4 | *Tubgcp4* | 0.68 | 1.28 |
| 1430034_at | chaperonin containing Tcp1, subunit 4 (delta) | *Cct4* | 0.67 | 1.28 |
| 1420710_at | reticuloendotheliosis oncogene | *Rel* | 0.66 | 1.28 |
| 1448824_at | ubiquitin-conjugating enzyme E2, J1 | *Ube2j1* | 0.68 | 1.28 |
| 1452254_at | myotubularin related protein 9 | *Mtmr9* | 0.57 | 1.28 |
| 1456424_s_at | phospholipid transfer protein | *Pltp* | 0.74 | 1.28 |
| 1416903_at | nucleobindin 1 | *Nucb1* | 0.65 | 1.28 |
| 1434420_x_at | translocase of outer mitochondrial membrane 22 homolog (yeast) | *Tomm22* | 0.80 | 1.28 |
| 1424275_s_at | tripartite motif-containing 41 | *Trim41* | 0.67 | 1.28 |
| 1453988_a_at | insulin degrading enzyme | *Ide* | 0.67 | 1.28 |
| 1425364_a_at | solute carrier family 3 (activators of dibasic and neutral amino acid transport), member 2 | *Slc3a2* | 0.73 | 1.28 |
| 1460342_s_at | myosin phosphatase Rho interacting protein | *Mprip* | 0.61 | 1.28 |
| 1429352_at | molybdenum cofactor sulfurase | *Mocos* | 0.62 | 1.28 |
| 1418881_at | N-terminal EF-hand calcium binding protein 2 | *Necab2* | 0.51 | 1.28 |
| 1455305_x_at | heterogeneous nuclear ribonucleoprotein A1 | *Hnrnpa1* | 0.68 | 1.28 |
| 1456273_x_at | thiopurine methyltransferase | *Tpmt* | 0.80 | 1.28 |
| 1435139_at | N(alpha)-acetyltransferase 15, NatA auxiliary subunit | *Naa15* | 0.53 | 1.28 |
| 1423780_at | 3-hydroxyisobutyrate dehydrogenase | *Hibadh* | 0.58 | 1.28 |
| 1415907_at | cyclin D3 | *Ccnd3* | 0.58 | 1.28 |
| 1423844_s_at | cystathionine beta-synthase | *Cbs* | 0.78 | 1.28 |
| 1447753_at | cell division cycle 37 homolog (S. cerevisiae)-like 1 | *Cdc37l1* | 0.57 | 1.28 |
| 1452583_s_at | galactose mutarotase | *Galm* | 0.62 | 1.28 |
| 1420157_s_at | ATP-binding cassette, sub-family F (GCN20), member 1 | *Abcf1* | 0.77 | 1.28 |
| 1429284_at | MOB1, Mps One Binder kinase activator-like 2B (yeast) | *Mobkl2b* | 0.74 | 1.28 |
| 1434065_at | CWF19-like 1, cell cycle control (S. pombe) | *Cwf19l1* | 0.44 | 1.28 |
| 1437519_x_at | hydroxyacyl glutathione hydrolase | *Hagh* | 0.62 | 1.28 |
| 1416648_at | dynein cytoplasmic 1 heavy chain 1 | *Dync1h1* | 0.77 | 1.28 |
| 1459976_s_at | superoxide dismutase 1, soluble | *Sod1* | 0.71 | 1.27 |
| 1424323_at | nucleolar complex associated 2 homolog (S. cerevisiae) | *Noc2l* | 0.69 | 1.27 |
| 1424751_at | activator of basal transcription 1 | *Abt1* | 0.65 | 1.27 |
| 1437662_at | acyl-CoA synthetase medium-chain family member 5 | *Acsm5* | 0.73 | 1.27 |
| 1423818_a_at | ADP-ribosylation factor-like 6 interacting protein 1 | *Arl6ip1* | 0.78 | 1.27 |
| 1427033_at | dynamin binding protein | *Dnmbp* | 0.60 | 1.27 |
| 1448351_at | coronin, actin binding protein 1B | *Coro1b* | 0.75 | 1.27 |
| 1450081_x_at | glucose phosphate isomerase 1 | *Gpi1* | 0.66 | 1.27 |
| 1433839_at | asparaginyl-tRNA synthetase 2 (mitochondrial)(putative) | *Nars2* | 0.68 | 1.27 |
| 1428924_at | molybdenum cofactor synthesis 3 | *Mocs3* | 0.76 | 1.27 |
| 1448362_at | DnaJ (Hsp40) homolog, subfamily C, member 7 | *Dnajc7* | 0.79 | 1.27 |
| 1421830_at | adenylate kinase 4 | *Ak4* | 0.64 | 1.27 |
| 1429396_at | autophagy related 16 like 2 (S. cerevisiae) | *Atg16l2* | 0.61 | 1.27 |
| 1423737_at | NADH dehydrogenase (ubiquinone) Fe-S protein 3 | *Ndufs3* | 0.62 | 1.27 |
| 1449362_a_at | misshapen-like kinase 1 (zebrafish) | *Mink1* | 0.76 | 1.27 |
| 1456378_s_at | F-box and leucine-rich repeat protein 20 | *Fbxl20* | 0.57 | 1.27 |
| 1449048_s_at | RAB4A, member RAS oncogene family | *Rab4a* | 0.77 | 1.27 |
| 1427888_a_at | spectrin alpha 2 | *Spna2* | 0.67 | 1.27 |
| 1453739_at | transmembrane protein 126B | *Tmem126b* | 0.76 | 1.27 |
| 1417610_at | apolipoprotein A-V | *Apoa5* | 0.79 | 1.27 |
| 1422978_at | glycoprotein m6b | *Gpm6b* | 0.77 | 1.27 |
| 1457067_at | vacuolar protein sorting 4b (yeast) | *Vps4b* | 0.56 | 1.27 |
| 1418653_at | cytochrome P450, family 2, subfamily c, polypeptide 50 | *Cyp2c50* | 0.58 | 1.27 |
| 1418537_at | isochorismatase domain containing 2b | *Isoc2b* | 0.76 | 1.27 |
| 1419356_at | Kruppel-like factor 7 (ubiquitous) | *Klf7* | 0.63 | 1.27 |
| 1416312_at | arginyl-tRNA synthetase | *Rars* | 0.72 | 1.27 |
| 1453604_a_at | Hbs1-like (S. cerevisiae) | *Hbs1l* | 0.54 | 1.27 |
| 1428290_at | mitochondrial intermediate peptidase | *Mipep* | 0.54 | 1.27 |
| 1422000_at | aldo-keto reductase family 1, member C12 | *Akr1c12* | 0.58 | 1.27 |
| 1451271_a_at | acetyl-Coenzyme A acetyltransferase 1 | *Acat1* | 0.65 | 1.27 |
| 1425623_a_at | cystathionine beta-synthase | *Cbs* | 0.64 | 1.27 |
| 1457793_a_at | Wolf-Hirschhorn syndrome candidate 1-like 1 (human) | *Whsc1l1* | 0.53 | 1.27 |
| 1421209_s_at | inhibitor of kappaB kinase gamma | *Ikbkg* | 0.75 | 1.27 |
| 1431028_a_at | pantothenate kinase 1 | *Pank1* | 0.69 | 1.27 |
| 1448589_at | NADH dehydrogenase (ubiquinone) 1 beta subcomplex, 5 | *Ndufb5* | 0.52 | 1.27 |
| 1439461_x_at | non-SMC element 4 homolog A (S. cerevisiae) | *Nsmce4a* | 0.72 | 1.27 |
| 1437156_at | N-terminal EF-hand calcium binding protein 1 | *Necab1* | 0.63 | 1.27 |
| 1416709_a_at | neugrin, neurite outgrowth associated | *Ngrn* | 0.75 | 1.27 |
| 1415722_a_at | Vps20-associated 1 homolog (S. cerevisiae) | *Vta1* | 0.61 | 1.27 |
| 1453744_a_at | ankyrin repeat domain 40 | *Ankrd40* | 0.79 | 1.27 |
| 1428789_at | Ral GEF with PH domain and SH3 binding motif 2 | *Ralgps2* | 0.75 | 1.27 |
| 1441866_s_at | phosphatidylserine synthase 1 | *Ptdss1* | 0.78 | 1.27 |
| 1434398_at | NF-kappaB repressing factor | *Nkrf* | 0.72 | 1.27 |
| 1427261_at | WW, C2 and coiled-coil domain containing 1 | *Wwc1* | 0.73 | 1.27 |
| 1428556_at | phosphatidylinositol glycan anchor biosynthesis, class Y | *Pigy* | 0.68 | 1.27 |
| 1415699_a_at | G protein pathway suppressor 1 | *Gps1* | 0.73 | 1.27 |
| 1460702_at | TP53 regulated inhibitor of apoptosis 1 | *Triap1* | 0.72 | 1.26 |
| 1451084_at | electron transferring flavoprotein, dehydrogenase | *Etfdh* | 0.49 | 1.26 |
| 1423411_at | RNA binding motif protein 47 | *Rbm47* | 0.38 | 1.26 |
| 1416884_at | chromobox homolog 3 (Drosophila HP1 gamma) | *Cbx3* | 0.79 | 1.26 |
| 1444297_at | serine (or cysteine) peptidase inhibitor, clade A, member 4, pseudogene 1 | *Serpina4-ps1* | 0.30 | 1.26 |
| 1434832_at | forkhead box O3 | *Foxo3* | 0.51 | 1.26 |
| 1451002_at | aconitase 2, mitochondrial | *Aco2* | 0.56 | 1.26 |
| 1456113_at | TBC1 domain family, member 25 | *Tbc1d25* | 0.77 | 1.26 |
| 1448488_at | mitochondrial ribosomal protein S5 | *Mrps5* | 0.59 | 1.26 |
| 1448337_at | ubiquinol-cytochrome c reductase complex chaperone, CBP3 homolog (yeast) | *Uqcc* | 0.65 | 1.26 |
| 1425966_x_at | ubiquitin C | *Ubc* | 0.53 | 1.26 |
| 1438758_at | acireductone dioxygenase 1 | *Adi1* | 0.63 | 1.26 |
| 1456015_x_at | NADH dehydrogenase (ubiquinone) flavoprotein 1 | *Ndufv1* | 0.69 | 1.26 |
| 1424309_a_at | molybdenum cofactor synthesis 2 | *Mocs2* | 0.60 | 1.26 |
| 1424087_at | proteasome (prosome, macropain) assembly chaperone 3 | *Psmg3* | 0.51 | 1.26 |
| 1424557_at | zinc finger, CCHC domain containing 9 | *Zcchc9* | 0.63 | 1.26 |
| 1438969_x_at | DEAH (Asp-Glu-Ala-His) box polypeptide 30 | *Dhx30* | 0.75 | 1.26 |
| 1439381_x_at | MARVEL (membrane-associating) domain containing 1 | *Marveld1* | 0.59 | 1.26 |
| 1452710_at | RNA pseudouridylate synthase domain containing 4 | *Rpusd4* | 0.77 | 1.26 |
| 1424329_a_at | proline-rich Gla (G-carboxyglutamic acid) polypeptide 2 | *Prrg2* | 0.76 | 1.26 |
| 1426662_at | cytidine monophospho-N-acetylneuraminic acid synthetase | *Cmas* | 0.67 | 1.26 |
| 1427413_a_at | CUGBP, Elav-like family member 1 | *Celf1* | 0.63 | 1.26 |
| 1452694_at | inositol hexaphosphate kinase 1 | *Ip6k1* | 0.80 | 1.26 |
| 1424101_at | heterogeneous nuclear ribonucleoprotein L | *Hnrnpl* | 0.75 | 1.26 |
| 1424174_at | Sh3kbp1 binding protein 1 | *Shkbp1* | 0.71 | 1.26 |
| 1432827_x_at | ubiquitin C | *Ubc* | 0.54 | 1.26 |
| 1439249_at | WW domain containing adaptor with coiled-coil | *Wac* | 0.76 | 1.26 |
| 1458327_x_at | solute carrier family 26 (sulfate transporter), member 1 | *Slc26a1* | 0.74 | 1.26 |
| 1449118_at | dihydrolipoamide branched chain transacylase E2 | *Dbt* | 0.57 | 1.26 |
| 1423769_at | pentatricopeptide repeat domain 2 | *Ptcd2* | 0.77 | 1.26 |
| 1417877_at | endonuclease/exonuclease/phosphatase family domain containing 1 | *Eepd1* | 0.69 | 1.26 |
| 1416794_at | atlastin GTPase 2 | *Atl2* | 0.63 | 1.26 |
| 1423739_x_at | amyloid beta (A4) precursor-like protein 2 | *Aplp2* | 0.79 | 1.26 |
| 1429739_a_at | POZ (BTB) and AT hook containing zinc finger 1 | *Patz1* | 0.57 | 1.26 |
| 1448153_at | cytochrome c oxidase, subunit Va | *Cox5a* | 0.74 | 1.26 |
| 1448261_at | cadherin 1 | *Cdh1* | 0.76 | 1.26 |
| 1455312_at | polyhomeotic-like 3 (Drosophila) | *Phc3* | 0.74 | 1.26 |
| 1435638_at | glycogen synthase kinase 3 alpha | *Gsk3a* | 0.77 | 1.26 |
| 1418087_at | ubiquitin fusion degradation 1 like | *Ufd1l* | 0.54 | 1.26 |
| 1416838_at | methylmalonyl-Coenzyme A mutase | *Mut* | 0.54 | 1.26 |
| 1433731_at | insulin-like growth factor 2 mRNA binding protein 3 | *Igf2bp3* | 0.58 | 1.26 |
| 1450400_at | trimethylguanosine synthase homolog (S. cerevisiae) | *Tgs1* | 0.55 | 1.26 |
| 1448889_at | solute carrier family 38, member 4 | *Slc38a4* | 0.74 | 1.25 |
| 1424011_at | aquaporin 9 | *Aqp9* | 0.79 | 1.25 |
| 1429639_at | glycerophosphocholine phosphodiesterase GDE1 homolog (S. cerevisiae) | *Gpcpd1* | 0.57 | 1.25 |
| 1436189_at | NAD(P)H dehydrogenase, quinone 2 | *Nqo2* | 0.53 | 1.25 |
| 1417382_at | ectonucleoside triphosphate diphosphohydrolase 5 | *Entpd5* | 0.59 | 1.25 |
| 1425057_at | phenazine biosynthesis-like protein domain containing | *Pbld* | 0.63 | 1.25 |
| 1448265_x_at | myelin protein zero-like 2 | *Mpzl2* | 0.68 | 1.25 |
| 1418179_at | autophagy-related 3 (yeast) | *Atg3* | 0.55 | 1.25 |
| 1447320_x_at | polymerase (RNA) I polypeptide D | *Polr1d* | 0.70 | 1.25 |
| 1436763_a_at | Kruppel-like factor 9 | *Klf9* | 0.62 | 1.25 |
| 1449457_at | acyl-CoA thioesterase 12 | *Acot12* | 0.49 | 1.25 |
| 1420712_a_at | hepsin | *Hpn* | 0.80 | 1.25 |
| 1448243_at | N-ethylmaleimide sensitive fusion protein attachment protein alpha | *Napa* | 0.73 | 1.25 |
| 1453080_at | apolipoprotein L 7a | *Apol7a* | 0.54 | 1.25 |
| 1448101_s_at | tripartite motif-containing 27 | *Trim27* | 0.80 | 1.25 |
| 1436970_a_at | platelet derived growth factor receptor, beta polypeptide | *Pdgfrb* | 0.58 | 1.25 |
| 1419188_s_at | chemokine (C-C motif) ligand 27A | *Ccl27a* | 0.56 | 1.25 |
| 1433470_a_at | inner membrane protein, mitochondrial | *Immt* | 0.60 | 1.25 |
| 1424196_at | Yip1 domain family, member 1 | *Yipf1* | 0.77 | 1.25 |
| 1455511_at | selenophosphate synthetase 1 | *Sephs1* | 0.66 | 1.25 |
| 1428507_at | haloacid dehalogenase-like hydrolase domain containing 2 | *Hdhd2* | 0.74 | 1.25 |
| 1417580_s_at | selenium binding protein 1 | *Selenbp1* | 0.58 | 1.25 |
| 1417707_at | NEDD4 binding protein 2-like 1 | *N4bp2l1* | 0.56 | 1.25 |
| 1443787_x_at | caspase 14 | *Casp14* | 0.45 | 1.25 |
| 1426385_x_at | tyrosine 3-monooxygenase/tryptophan 5-monooxygenase activation protein, epsilon polypeptide | *Ywhae* | 0.78 | 1.25 |
| 1416730_at | RNA terminal phosphate cyclase-like 1 | *Rcl1* | 0.67 | 1.25 |
| 1423436_at | glutathione S-transferase, alpha 3 | *Gsta3* | 0.60 | 1.25 |
| 1449374_at | pipecolic acid oxidase | *Pipox* | 0.73 | 1.25 |
| 1442042_at | hypothetical LOC100504637 | *LOC100504637* | 0.79 | 1.25 |
| 1452944_at | arylformamidase | *Afmid* | 0.39 | 1.25 |
| 1448579_at | golgi apparatus protein 1 | *Glg1* | 0.76 | 1.25 |
| 1455733_at | TAO kinase 3 | *Taok3* | 0.73 | 1.25 |
| 1421622_a_at | Rap guanine nucleotide exchange factor (GEF) 4 | *Rapgef4* | 0.64 | 1.25 |
| 1448092_x_at | serine (or cysteine) peptidase inhibitor, clade A, member 4, pseudogene 1 | *Serpina4-ps1* | 0.31 | 1.25 |
| 1427371_at | ATP-binding cassette, sub-family A (ABC1), member 8a | *Abca8a* | 0.46 | 1.25 |
| 1416100_at | eukaryotic translation initiation factor 3, subunit D | *Eif3d* | 0.73 | 1.25 |
| 1422288_at | 5-hydroxytryptamine (serotonin) receptor 1B | *Htr1b* | 0.42 | 1.24 |
| 1456150_at | jumonji C domain-containing histone demethylase 1 homolog D (S. cerevisiae) | *Jhdm1d* | 0.59 | 1.24 |
| 1422576_at | ataxin 10 | *Atxn10* | 0.68 | 1.24 |
| 1419435_at | aldehyde oxidase 1 | *Aox1* | 0.69 | 1.24 |
| 1418788_at | endothelial-specific receptor tyrosine kinase | *Tek* | 0.65 | 1.24 |
| 1460594_a_at | GDP-mannose pyrophosphorylase A | *Gmppa* | 0.73 | 1.24 |
| 1433567_at | guanine monophosphate synthetase | *Gmps* | 0.77 | 1.24 |
| 1416058_s_at | ATP synthase, H+ transporting, mitochondrial F1 complex, gamma polypeptide 1 | *Atp5c1* | 0.66 | 1.24 |
| 1444085_at | prenyl (solanesyl) diphosphate synthase, subunit 2 | *Pdss2* | 0.63 | 1.24 |
| 1448902_at | tetratricopeptide repeat domain 23 | *Ttc23* | 0.69 | 1.24 |
| 1438759_x_at | acireductone dioxygenase 1 | *Adi1* | 0.68 | 1.24 |
| 1429139_at | OTU domain containing 7B | *Otud7b* | 0.72 | 1.24 |
| 1425869_a_at | presenilin 2 | *Psen2* | 0.69 | 1.24 |
| 1456728_x_at | aconitase 1 | *Aco1* | 0.65 | 1.24 |
| 1433513_x_at | NADH dehydrogenase (ubiquinone) 1 alpha subcomplex, 12 | *Ndufa12* | 0.62 | 1.24 |
| 1455155_at | LSM14 homolog B (SCD6, S. cerevisiae) | *Lsm14b* | 0.66 | 1.24 |
| 1423975_s_at | nuclear mitotic apparatus protein 1 | *Numa1* | 0.51 | 1.24 |
| 1455841_s_at | glutamate-rich WD repeat containing 1 | *Grwd1* | 0.58 | 1.24 |
| 1422751_at | transducin-like enhancer of split 1, homolog of Drosophila E(spl) | *Tle1* | 0.60 | 1.24 |
| 1422788_at | solute carrier family 43, member 3 | *Slc43a3* | 0.60 | 1.24 |
| 1453061_at | elaC homolog 1 (E. coli) | *Elac1* | 0.57 | 1.24 |
| 1430474_a_at | mitochondrial carrier homolog 2 (C. elegans) | *Mtch2* | 0.63 | 1.24 |
| 1430522_a_at | vesicle-associated membrane protein 5 | *Vamp5* | 0.77 | 1.24 |
| 1434254_at | guanine nucleotide binding protein, alpha 11 | *Gna11* | 0.73 | 1.24 |
| 1427042_at | mal, T-cell differentiation protein 2 | *Mal2* | 0.78 | 1.24 |
| 1428798_s_at | SET domain containing 6 | *Setd6* | 0.64 | 1.24 |
| 1448323_a_at | biglycan | *Bgn* | 0.75 | 1.24 |
| 1448990_a_at | myosin IB | *Myo1b* | 0.62 | 1.24 |
| 1432526_a_at | SNF8, ESCRT-II complex subunit, homolog (S. cerevisiae) | *Snf8* | 0.71 | 1.24 |
| 1421938_at | serine hydrolase-like | *Serhl* | 0.80 | 1.23 |
| 1431833_a_at | 3-hydroxy-3-methylglutaryl-Coenzyme A synthase 2 | *Hmgcs2* | 0.41 | 1.23 |
| 1419471_a_at | nuclear distribution gene C homolog (Aspergillus) | *Nudc* | 0.53 | 1.23 |
| 1419177_at | vacuolar protein sorting 37A (yeast) | *Vps37a* | 0.75 | 1.23 |
| 1428257_s_at | dynein light chain roadblock-type 1 | *Dynlrb1* | 0.57 | 1.23 |
| 1424715_at | retinol saturase (all trans retinol 13,14 reductase) | *Retsat* | 0.28 | 1.23 |
| 1455490_at | polymeric immunoglobulin receptor | *Pigr* | 0.71 | 1.23 |
| 1433490_s_at | erythrocyte protein band 4.1-like 2 | *Epb4.1l2* | 0.64 | 1.23 |
| 1433970_at | bolA-like 3 (E. coli) | *Bola3* | 0.71 | 1.23 |
| 1426378_at | eukaryotic translation initiation factor 4B | *Eif4b* | 0.70 | 1.23 |
| 1421877_at | mitogen-activated protein kinase 9 | *Mapk9* | 0.77 | 1.23 |
| 1424783_a_at | UDP glucuronosyltransferase 1 family, polypeptide A1 | *Ugt1a1* | 0.74 | 1.23 |
| 1435370_a_at | carboxylesterase 1D | *Ces1d* | 0.62 | 1.23 |
| 1426750_at | filamin, beta | *Flnb* | 0.44 | 1.23 |
| 1460684_at | transmembrane 7 superfamily member 2 | *Tm7sf2* | 0.65 | 1.23 |
| 1424531_a_at | transcription elongation factor A (SII), 3 | *Tcea3* | 0.49 | 1.23 |
| 1447783_x_at | solute carrier family 25, member 39 | *Slc25a39* | 0.77 | 1.23 |
| 1447112_s_at | crystallin, lambda 1 | *Cryl1* | 0.41 | 1.23 |
| 1460556_at | microtubule associated monoxygenase, calponin and LIM domain containing -like 1 | *Micall1* | 0.57 | 1.23 |
| 1423594_a_at | endothelin receptor type B | *Ednrb* | 0.62 | 1.23 |
| 1435478_at | WD repeat domain 26 | *Wdr26* | 0.63 | 1.23 |
| 1417271_a_at | endoglin | *Eng* | 0.77 | 1.23 |
| 1427020_at | scavenger receptor class A, member 3 | *Scara3* | 0.56 | 1.23 |
| 1419547_at | fumarylacetoacetate hydrolase domain containing 1 | *Fahd1* | 0.74 | 1.23 |
| 1452890_at | tubulin tyrosine ligase-like family, member 5 | *Ttll5* | 0.68 | 1.23 |
| 1459728_at | ISY1 splicing factor homolog (S. cerevisiae) | *Isy1* | 0.78 | 1.23 |
| 1427297_at | mitochondrial ribosomal protein L9 | *Mrpl9* | 0.60 | 1.23 |
| 1419491_at | defensin beta 1 | *Defb1* | 0.68 | 1.23 |
| 1447084_at | nuclear factor of activated T-cells, cytoplasmic, calcineurin-dependent 1 | *Nfatc1* | 0.51 | 1.23 |
| 1419664_at | serine racemase | *Srr* | 0.70 | 1.23 |
| 1448630_a_at | succinate dehydrogenase complex, subunit C, integral membrane protein | *Sdhc* | 0.57 | 1.23 |
| 1448426_at | sarcosine dehydrogenase | *Sardh* | 0.75 | 1.23 |
| 1457800_at | Solute carrier family 1 (glial high affinity glutamate transporter), member 2 | *Slc1a2* | 0.68 | 1.23 |
| 1427964_at | CKLF-like MARVEL transmembrane domain containing 8 | *Cmtm8* | 0.66 | 1.23 |
| 1419520_at | N-acetyltransferase 8 (GCN5-related, putative) | *Nat8* | 0.61 | 1.23 |
| 1417499_at | translocase of inner mitochondrial membrane 10 homolog (yeast) | *Timm10* | 0.64 | 1.23 |
| 1433725_at | activin A receptor, type 1B | *Acvr1b* | 0.54 | 1.23 |
| 1430021_a_at | SUMO1 activating enzyme subunit 1 | *Sae1* | 0.74 | 1.23 |
| 1447943_x_at | YEATS domain containing 2 | *Yeats2* | 0.79 | 1.23 |
| 1437211_x_at | ELOVL family member 5, elongation of long chain fatty acids (yeast) | *Elovl5* | 0.48 | 1.23 |
| 1418242_at | Fas-associated factor 1 | *Faf1* | 0.73 | 1.23 |
| 1452686_s_at | transmembrane protein 222 | *Tmem222* | 0.78 | 1.23 |
| 1450133_at | UDP glucuronosyltransferase 2 family, polypeptide A3 | *Ugt2a3* | 0.64 | 1.23 |
| 1416918_at | discs, large homolog 3 (Drosophila) | *Dlg3* | 0.76 | 1.23 |
| 1425350_a_at | myelin basic protein expression factor 2, repressor | *Myef2* | 0.79 | 1.23 |
| 1419094_at | cytochrome P450, family 2. subfamily c, polypeptide 37 | *Cyp2c37* | 0.38 | 1.23 |
| 1451315_at | transmembrane protein 101 | *Tmem101* | 0.73 | 1.23 |
| 1429199_s_at | serum amyloid A-like 1 | *Saal1* | 0.63 | 1.23 |
| 1454646_at | t-complex 11 (mouse) like 2 | *Tcp11l2* | 0.78 | 1.22 |
| 1431251_at | leucine rich repeat containing 3 | *Lrrc3* | 0.72 | 1.22 |
| 1449316_at | cytochrome P450, family 4, subfamily f, polypeptide 15 | *Cyp4f15* | 0.61 | 1.22 |
| 1450014_at | claudin 1 | *Cldn1* | 0.48 | 1.22 |
| 1418016_at | pumilio 2 (Drosophila) | *Pum2* | 0.60 | 1.22 |
| 1451988_s_at | chromatin modifying protein 4B | *Chmp4b* | 0.79 | 1.22 |
| 1460230_at | synapsin II | *Syn2* | 0.59 | 1.22 |
| 1428576_at | hypoxia-inducible factor 1, alpha subunit inhibitor | *Hif1an* | 0.52 | 1.22 |
| 1437507_at | GATA zinc finger domain containing 2B | *Gatad2b* | 0.47 | 1.22 |
| 1435772_at | kinesin family member 21B | *Kif21b* | 0.77 | 1.22 |
| 1430128_a_at | receptor accessory protein 6 | *Reep6* | 0.56 | 1.22 |
| 1423063_at | DNA methyltransferase 3A | *Dnmt3a* | 0.59 | 1.22 |
| 1424002_at | phosducin-like 3 | *Pdcl3* | 0.76 | 1.22 |
| 1425179_at | serine hydroxymethyltransferase 1 (soluble) | *Shmt1* | 0.55 | 1.22 |
| 1433672_at | TRM2 tRNA methyltransferase 2 homolog B (S. cerevisiae) | *Trmt2b* | 0.68 | 1.22 |
| 1452257_at | 3-hydroxybutyrate dehydrogenase, type 1 | *Bdh1* | 0.78 | 1.22 |
| 1450816_at | polymerase (DNA directed), gamma 2, accessory subunit | *Polg2* | 0.50 | 1.22 |
| 1429681_a_at | trans-2,3-enoyl-CoA reductase | *Tecr* | 0.57 | 1.22 |
| 1432212_at | APOBEC1 complementation factor | *A1cf* | 0.75 | 1.22 |
| 1452009_at | tetratricopeptide repeat domain 39B | *Ttc39b* | 0.62 | 1.22 |
| 1426696_at | low density lipoprotein receptor-related protein associated protein 1 | *Lrpap1* | 0.71 | 1.22 |
| 1426163_x_at | ribosomal protein L7 | *Rpl7* | 0.75 | 1.22 |
| 1432181_s_at | SCO cytochrome oxidase deficient homolog 2 (yeast) | *Sco2* | 0.72 | 1.22 |
| 1449087_at | ring finger protein 141 | *Rnf141* | 0.59 | 1.22 |
| 1426445_at | CTAGE family, member 5 | *Ctage5* | 0.56 | 1.22 |
| 1427552_a_at | glutathione transferase zeta 1 (maleylacetoacetate isomerase) | *Gstz1* | 0.66 | 1.22 |
| 1426345_at | prolyl endopeptidase-like | *Prepl* | 0.76 | 1.22 |
| 1429239_a_at | StAR-related lipid transfer (START) domain containing 4 | *Stard4* | 0.48 | 1.22 |
| 1421334_x_at | myoneurin | *Mynn* | 0.69 | 1.22 |
| 1423633_at | small nuclear ribonucleoprotein 48 (U11/U12) | *Snrnp48* | 0.65 | 1.22 |
| 1418644_a_at | serine/threonine kinase 11 | *Stk11* | 0.74 | 1.22 |
| 1450908_at | eukaryotic translation initiation factor 4E | *Eif4e* | 0.71 | 1.22 |
| 1433408_a_at | minichromosome maintenance deficient 10 (S. cerevisiae) | *Mcm10* | 0.45 | 1.22 |
| 1449518_at | glutaminyl-peptide cyclotransferase-like | *Qpctl* | 0.76 | 1.22 |
| 1454649_at | steroid 5 alpha-reductase 1 | *Srd5a1* | 0.30 | 1.22 |
| 1426117_a_at | solute carrier family 19 (thiamine transporter), member 2 | *Slc19a2* | 0.77 | 1.22 |
| 1418321_at | dodecenoyl-Coenzyme A delta isomerase (3,2 trans-enoyl-Coenyme A isomerase) | *Dci* | 0.52 | 1.22 |
| 1429178_at | odd Oz/ten-m homolog 3 (Drosophila) | *Odz3* | 0.58 | 1.22 |
| 1421212_at | ATP-binding cassette, sub-family C (CFTR/MRP), member 6 | *Abcc6* | 0.70 | 1.22 |
| 1436173_at | deleted in liver cancer 1 | *Dlc1* | 0.71 | 1.22 |
| 1417329_at | solute carrier family 23 (nucleobase transporters), member 2 | *Slc23a2* | 0.56 | 1.22 |
| 1456541_x_at | ATPase family, AAA domain containing 3A | *Atad3a* | 0.79 | 1.22 |
| 1421857_at | a disintegrin and metallopeptidase domain 17 | *Adam17* | 0.62 | 1.22 |
| 1419349_a_at | cytochrome P450, family 2, subfamily d, polypeptide 9 | *Cyp2d9* | 0.46 | 1.22 |
| 1420494_x_at | ubiquitin C | *Ubc* | 0.72 | 1.22 |
| 1422147_a_at | phospholipase A2, group VI | *Pla2g6* | 0.56 | 1.22 |
| 1429778_at | optineurin | *Optn* | 0.69 | 1.22 |
| 1450769_s_at | StAR-related lipid transfer (START) domain containing 5 | *Stard5* | 0.76 | 1.22 |
| 1437398_a_at | aldehyde dehydrogenase 9, subfamily A1 | *Aldh9a1* | 0.69 | 1.22 |
| 1419283_s_at | tensin 1 | *Tns1* | 0.67 | 1.22 |
| 1448856_a_at | methionine sulfoxide reductase A | *Msra* | 0.71 | 1.21 |
| 1428367_at | N-deacetylase/N-sulfotransferase (heparan glucosaminyl) 1 | *Ndst1* | 0.70 | 1.21 |
| 1423757_x_at | transmembrane protein 48 | *Tmem48* | 0.73 | 1.21 |
| 1422855_at | cleavage and polyadenylation specificity factor 3 | *Cpsf3* | 0.72 | 1.21 |
| 1436644_x_at | transmembrane protein 25 | *Tmem25* | 0.75 | 1.21 |
| 1460024_at | trinucleotide repeat containing 6b | *Tnrc6b* | 0.62 | 1.21 |
| 1418067_at | cofilin 2, muscle | *Cfl2* | 0.75 | 1.21 |
| 1451307_at | mitochondrial ribosomal protein L14 | *Mrpl14* | 0.71 | 1.21 |
| 1436991_x_at | gelsolin | *Gsn* | 0.61 | 1.21 |
| 1426584_a_at | sorbitol dehydrogenase | *Sord* | 0.68 | 1.21 |
| 1425266_a_at | RAP1, GTP-GDP dissociation stimulator 1 | *Rap1gds1* | 0.72 | 1.21 |
| 1436426_at | coiled-coil and C2 domain containing 2A | *Cc2d2a* | 0.52 | 1.21 |
| 1449628_s_at | START domain containing 7 | *Stard7* | 0.67 | 1.21 |
| 1434807_s_at | metaxin 3 | *Mtx3* | 0.67 | 1.21 |
| 1434185_at | acetyl-Coenzyme A carboxylase alpha | *Acaca* | 0.49 | 1.21 |
| 1433858_at | leucine rich repeat containing 28 | *Lrrc28* | 0.67 | 1.21 |
| 1437455_a_at | B-cell translocation gene 1, anti-proliferative | *Btg1* | 0.72 | 1.21 |
| 1440260_at | coiled-coil domain containing 58 | *Ccdc58* | 0.57 | 1.21 |
| 1450643_s_at | acyl-CoA synthetase long-chain family member 1 | *Acsl1* | 0.29 | 1.21 |
| 1452307_at | CDK5 and Abl enzyme substrate 2 | *Cables2* | 0.50 | 1.21 |
| 1451956_a_at | sigma non-opioid intracellular receptor 1 | *Sigmar1* | 0.56 | 1.21 |
| 1448442_a_at | proteasome (prosome, macropain) subunit, alpha type 3 | *Psma3* | 0.77 | 1.21 |
| 1424430_at | MTERF domain containing 2 | *Mterfd2* | 0.63 | 1.21 |
| 1419816_s_at | ERBB receptor feedback inhibitor 1 | *Errfi1* | 0.68 | 1.21 |
| 1424459_at | lysophosphatidylcholine acyltransferase 1 | *Lpcat1* | 0.77 | 1.21 |
| 1420973_at | AT rich interactive domain 5B (MRF1-like) | *Arid5b* | 0.37 | 1.21 |
| 1418025_at | basic helix-loop-helix family, member e40 | *Bhlhe40* | 0.63 | 1.21 |
| 1428308_at | p53 and DNA damage regulated 1 | *Pdrg1* | 0.73 | 1.21 |
| 1448652_at | intraflagellar transport 88 homolog (Chlamydomonas) | *Ift88* | 0.68 | 1.21 |
| 1424364_a_at | ubiquinol-cytochrome c reductase, complex III subunit X | *Uqcr10* | 0.72 | 1.21 |
| 1421939_a_at | stromal antigen 1 | *Stag1* | 0.57 | 1.21 |
| 1436209_at | DnaJ (Hsp40) homolog, subfamily C, member 16 | *Dnajc16* | 0.80 | 1.21 |
| 1450624_at | betaine-homocysteine methyltransferase | *Bhmt* | 0.77 | 1.21 |
| 1418002_at | HIG1 domain family, member 2A | *Higd2a* | 0.71 | 1.21 |
| 1423242_at | mitochondrial ribosomal protein S36 | *Mrps36* | 0.63 | 1.21 |
| 1450768_at | discs, large homolog 1 (Drosophila) | *Dlg1* | 0.45 | 1.21 |
| 1424221_at | sushi domain containing 4 | *Susd4* | 0.63 | 1.21 |
| 1430570_at | kynureninase (L-kynurenine hydrolase) | *Kynu* | 0.58 | 1.21 |
| 1450998_at | zinc finger protein 110 | *Zfp110* | 0.66 | 1.21 |
| 1423460_at | GRB10 interacting GYF protein 1 | *Gigyf1* | 0.75 | 1.21 |
| 1428255_at | Luc7 homolog (S. cerevisiae)-like | *Luc7l* | 0.75 | 1.20 |
| 1416133_at | EFR3 homolog A (S. cerevisiae) | *Efr3a* | 0.72 | 1.20 |
| 1416391_at | pentatricopeptide repeat domain 1 | *Ptcd1* | 0.77 | 1.20 |
| 1433505_a_at | leucine rich repeat containing 8D | *Lrrc8d* | 0.67 | 1.20 |
| 1448470_at | fructose bisphosphatase 1 | *Fbp1* | 0.58 | 1.20 |
| 1422869_at | c-mer proto-oncogene tyrosine kinase | *Mertk* | 0.68 | 1.20 |
| 1449710_s_at | ATP synthase, H+ transporting, mitochondrial F1 complex, alpha subunit 1 | *Atp5a1* | 0.60 | 1.20 |
| 1421870_at | tripartite motif-containing 44 | *Trim44* | 0.62 | 1.20 |
| 1418927_a_at | hyaluronic acid binding protein 4 | *Habp4* | 0.73 | 1.20 |
| 1437500_at | nucleolar complex associated 3 homolog (S. cerevisiae) | *Noc3l* | 0.68 | 1.20 |
| 1415881_at | growth hormone inducible transmembrane protein | *Ghitm* | 0.65 | 1.20 |
| 1418439_at | mitochondrial ribosomal protein L42 | *Mrpl42* | 0.78 | 1.20 |
| 1424841_s_at | ribokinase | *Rbks* | 0.69 | 1.20 |
| 1434486_x_at | UDP-glucose pyrophosphorylase 2 | *Ugp2* | 0.53 | 1.20 |
| 1448451_at | adenylate kinase 2 | *Ak2* | 0.56 | 1.20 |
| 1453042_at | ankyrin repeat and sterile alpha motif domain containing 4B | *Anks4b* | 0.61 | 1.20 |
| 1423772_x_at | solute carrier family 25 (mitochondrial carrier, adenine nucleotide translocator), member 5 | *Slc25a5* | 0.74 | 1.20 |
| 1416765_s_at | presequence translocase-asssociated motor 16 homolog (S. cerevisiae) | *Pam16* | 0.78 | 1.20 |
| 1428623_at | plexin A1 | *Plxna1* | 0.55 | 1.20 |
| 1424391_at | nardilysin, N-arginine dibasic convertase, NRD convertase 1 | *Nrd1* | 0.79 | 1.20 |
| 1434056_a_at | NADH dehydrogenase (ubiquinone) 1 beta subcomplex, 6 | *Ndufb6* | 0.68 | 1.20 |

1793 Down-regulated genes in DSSWOL as compared to DSS

|  |  |  | Fold Change | Fold Change |
| --- | --- | --- | --- | --- |
| Probe Set ID | Gene Title | Gene Symbol | DSS vs. CON | DSSWOL vs. DSS |
| 1424769_s_at | caldesmon 1 | *Cald1* | 1.41 | 0.80 |
| 1448471_a_at | cytotoxic T lymphocyte-associated protein 2 alpha | *Ctla2a* | 2.92 | 0.80 |
| 1433951_at | ADP-ribosylation factor-like 5A | *Arl5a* | 1.21 | 0.80 |
| 1453053_at | centromere protein W | *Cenpw* | 2.39 | 0.80 |
| 1428014_at | histone cluster 1, H4h | *Hist1h4h* | 1.55 | 0.80 |
| 1417110_at | mannosidase 1, alpha | *Man1a* | 1.28 | 0.80 |
| 1441989_at | BCL2/adenovirus E1B interacting protein 2 | *Bnip2* | 1.55 | 0.80 |
| 1419235_s_at | helicase (DNA) B | *Helb* | 1.63 | 0.80 |
| 1453920_a_at | motile sperm domain containing 2 | *Mospd2* | 1.23 | 0.80 |
| 1457088_at | pallidin | *Pldn* | 1.36 | 0.80 |
| 1452058_a_at | ring finger protein 11 | *Rnf11* | 1.23 | 0.80 |
| 1455608_at | sodium channel and clathrin linker 1 | *Sclt1* | 1.73 | 0.80 |
| 1448939_at | ubiquitin specific peptidase 25 | *Usp25* | 1.34 | 0.80 |
| 1435771_at | phospholipase C, beta 4 | *Plcb4* | 2.51 | 0.80 |
| 1417478_a_at | protein phosphatase 2, regulatory subunit B'', gamma | *Ppp2r3c* | 1.41 | 0.80 |
| 1434424_at | major facilitator superfamily domain containing 7B | *Mfsd7b* | 2.11 | 0.80 |
| 1460339_at | proteasome (prosome, macropain) subunit, alpha type 4 | *Psma4* | 1.31 | 0.80 |
| 1433709_at | calcium activated nucleotidase 1 | *Cant1* | 1.61 | 0.80 |
| 1433547_s_at | NudC domain containing 1 | *Nudcd1* | 1.30 | 0.80 |
| 1422603_at | ribonuclease, RNase A family 4 | *Rnase4* | 1.99 | 0.80 |
| 1436584_at | sprouty homolog 2 (Drosophila) | *Spry2* | 1.36 | 0.80 |
| 1431109_at | proline rich 16 | *Prr16* | 1.25 | 0.80 |
| 1423114_at | ubiquitin-conjugating enzyme E2D 3 (UBC4/5 homolog, yeast) | *Ube2d3* | 1.24 | 0.80 |
| 1418501_a_at | oxidation resistance 1 | *Oxr1* | 1.26 | 0.80 |
| 1448881_at | transferrin receptor | *Tfrc* | 2.09 | 0.80 |
| 1434303_at | Ras association (RalGDS/AF-6) and pleckstrin homology domains 1 | *Raph1* | 1.49 | 0.80 |
| 1433428_x_at | transglutaminase 2, C polypeptide | *Tgm2* | 2.10 | 0.80 |
| 1452176_at | nucleoporin 153 | *Nup153* | 1.51 | 0.80 |
| 1428107_at | SH3-binding domain glutamic acid-rich protein like | *Sh3bgrl* | 1.95 | 0.80 |
| 1454826_at | zinc finger and BTB domain containing 11 | *Zbtb11* | 2.04 | 0.80 |
| 1419233_x_at | apolipoprotein A-I | *Apoa1* | 1.29 | 0.80 |
| 1426880_at | enhancer trap locus 4 | *Etl4* | 1.58 | 0.80 |
| 1438750_at | Alpha thalassemia/mental retardation syndrome X-linked homolog (human) | *Atrx* | 1.89 | 0.80 |
| 1424020_at | ADP-ribosylation factor-like 6 interacting protein 6 | *Arl6ip6* | 1.79 | 0.80 |
| 1460295_s_at | interleukin 6 signal transducer | *Il6st* | 1.31 | 0.80 |
| 1425486_s_at | myotubularin related protein 6 | *Mtmr6* | 1.30 | 0.80 |
| 1425346_at | zinc finger protein 318 | *Zfp318* | 1.38 | 0.80 |
| 1451867_x_at | Rho GTPase activating protein 6 | *Arhgap6* | 1.22 | 0.80 |
| 1421430_at | RAD51-like 1 (S. cerevisiae) | *Rad51l1* | 5.26 | 0.80 |
| 1438163_x_at | rhomboid domain containing 2 | *Rhbdd2* | 1.65 | 0.80 |
| 1443733_x_at | polymerase (DNA-directed), delta 3, accessory subunit | *Pold3* | 1.44 | 0.80 |
| 1452318_a_at | heat shock protein 1B | *Hspa1b* | 1.22 | 0.80 |
| 1458343_x_at | mediator of RNA polymerase II transcription, subunit 6 homolog (yeast) | *Med6* | 1.38 | 0.80 |
| 1419578_at | mannose-binding lectin (protein A) 1 | *Mbl1* | 2.53 | 0.80 |
| 1456359_at | peptidylprolyl isomerase domain and WD repeat containing 1 | *Ppwd1* | 1.42 | 0.80 |
| 1450021_at | ubiquilin 2 | *Ubqln2* | 1.88 | 0.80 |
| 1427407_s_at | thyroid hormone receptor interactor 11 | *Trip11* | 1.39 | 0.79 |
| 1455968_x_at | transmembrane emp24 domain trafficking protein 2 | *Tmed2* | 1.36 | 0.79 |
| 1449401_at | complement component 1, q subcomponent, C chain | *C1qc* | 2.41 | 0.79 |
| 1454783_at | interleukin 13 receptor, alpha 1 | *Il13ra1* | 2.68 | 0.79 |
| 1415993_at | squalene epoxidase | *Sqle* | 2.28 | 0.79 |
| 1460403_at | PC4 and SFRS1 interacting protein 1 | *Psip1* | 1.38 | 0.79 |
| 1416145_at | DEAH (Asp-Glu-Ala-His) box polypeptide 15 | *Dhx15* | 1.32 | 0.79 |
| 1427235_at | 4lysine (K)-specific demethylase 6A | *Kdm6a* | 2.32 | 0.79 |
| 1455204_at | phosphatidylinositol transfer protein, cytoplasmic 1 | *Pitpnc1* | 1.29 | 0.79 |
| 1460552_at | small nuclear ribonucleoprotein 200 (U5) | *Snrnp200* | 1.27 | 0.79 |
| 1418911_s_at | chemokine (C-X-C motif) ligand 3 | *Cxcl3* | 1.35 | 0.79 |
| 1452767_at | ribosome binding protein 1 | *Rrbp1* | 1.56 | 0.79 |
| 1425977_a_at | STE20-like kinase (yeast) | *Slk* | 1.37 | 0.79 |
| 1447651_x_at | mitochondrial ribosomal protein L30 | *Mrpl30* | 13.82 | 0.79 |
| 1427658_at | chitobiase, di-N-acetyl- | *Ctbs* | 2.78 | 0.79 |
| 1438650_x_at | gap junction protein, alpha 1 | *Gja1* | 2.07 | 0.79 |
| 1421855_at | fibrinogen-like protein 2 | *Fgl2* | 2.57 | 0.79 |
| 1426813_at | LTV1 homolog (S. cerevisiae) | *Ltv1* | 1.30 | 0.79 |
| 1455072_at | centrosomal protein 350 | *Cep350* | 1.49 | 0.79 |
| 1418133_at | B-cell leukemia/lymphoma 3 | *Bcl3* | 2.37 | 0.79 |
| 1415863_at | eukaryotic translation initiation factor 4, gamma 2 | *Eif4g2* | 1.48 | 0.79 |
| 1420832_at | quiescin Q6 sulfhydryl oxidase 1 | *Qsox1* | 2.18 | 0.79 |
| 1453570_x_at | blocked early in transport 1 homolog (S. cerevisiae)-like | *Bet1l* | 1.38 | 0.79 |
| 1416401_at | CD82 antigen | *Cd82* | 1.25 | 0.79 |
| 1455403_at | mannosidase, endo-alpha | *Manea* | 1.47 | 0.79 |
| 1439012_a_at | deoxycytidine kinase | *Dck* | 1.95 | 0.79 |
| 1435127_a_at | O-sialoglycoprotein endopeptidase-like 1 | *Osgepl1* | 1.20 | 0.79 |
| 1416148_at | lysosomal-associated protein transmembrane 4B | *Laptm4b* | 1.39 | 0.79 |
| 1456059_at | proteasome (prosome, macropain) 26S subunit, non-ATPase, 11 | *Psmd11* | 1.20 | 0.79 |
| 1437457_a_at | myotrophin | *Mtpn* | 2.02 | 0.79 |
| 1433835_at | protein phosphatase 3, catalytic subunit, beta isoform | *Ppp3cb* | 1.91 | 0.79 |
| 1416025_at | fibrinogen gamma chain | *Fgg* | 1.55 | 0.79 |
| 1454626_at | clathrin, heavy polypeptide (Hc) | *Cltc* | 1.59 | 0.79 |
| 1423088_at | tropomodulin 3 | *Tmod3* | 1.27 | 0.79 |
| 1435769_at | A kinase (PRKA) anchor protein (yotiao) 9 | *Akap9* | 1.21 | 0.79 |
| 1456229_at | homeobox B3 | *Hoxb3* | 1.31 | 0.79 |
| 1437628_s_at | ras homolog gene family, member A | *Rhoa* | 1.40 | 0.79 |
| 1434556_at | transmembrane protein 170B | *Tmem170b* | 1.32 | 0.79 |
| 1429737_a_at | cardiolipin synthase 1 | *Crls1* | 1.65 | 0.79 |
| 1416642_a_at | tumor protein, translationally-controlled 1 | *Tpt1* | 1.26 | 0.79 |
| 1426643_at | elongation protein 3 homolog (S. cerevisiae) | *Elp3* | 1.46 | 0.79 |
| 1418397_at | zinc finger protein 275 | *Zfp275* | 1.21 | 0.79 |
| 1455396_at | ATPase, class I, type 8B, member 1 | *Atp8b1* | 1.89 | 0.79 |
| 1452247_at | fragile X mental retardation gene 1, autosomal homolog | *Fxr1* | 1.42 | 0.79 |
| 1428531_at | integrator complex subunit 7 | *Ints7* | 1.21 | 0.79 |
| 1418038_s_at | dual specificity phosphatase 19 | *Dusp19* | 1.25 | 0.79 |
| 1422849_a_at | poly(A) binding protein, nuclear 1 | *Pabpn1* | 1.22 | 0.79 |
| 1455868_a_at | tubulin, gamma complex associated protein 2 | *Tubgcp2* | 1.38 | 0.79 |
| 1427544_a_at | poly (A) polymerase alpha | *Papola* | 1.51 | 0.79 |
| 1433914_at | lipase, member O1 | *Lipo1* | 1.70 | 0.79 |
| 1455656_at | B and T lymphocyte associated | *Btla* | 2.14 | 0.79 |
| 1433973_at | selenophosphate synthetase 1 | *Sephs1* | 1.31 | 0.79 |
| 1417222_a_at | transmembrane protein 123 | *Tmem123* | 1.21 | 0.79 |
| 1439530_a_at | protein phosphatase 1, regulatory (inhibitor) subunit 13 like | *Ppp1r13l* | 2.03 | 0.79 |
| 1449200_at | nucleoporin 155 | *Nup155* | 1.52 | 0.79 |
| 1423522_at | nucleoplasmin 3 | *Npm3* | 2.13 | 0.79 |
| 1424264_at | mediator of RNA polymerase II transcription, subunit 6 homolog (yeast) | *Med6* | 1.38 | 0.79 |
| 1438487_s_at | zinc finger, ZZ domain containing 3 | *Zzz3* | 1.73 | 0.79 |
| 1422470_at | BCL2/adenovirus E1B interacting protein 3 | *Bnip3* | 1.44 | 0.79 |
| 1418014_a_at | UDP-Gal:betaGlcNAc beta 1,4- galactosyltransferase, polypeptide 1 | *B4galt1* | 1.38 | 0.79 |
| 1427985_at | spindlin family, member 4 | *Spin4* | 4.87 | 0.79 |
| 1418428_at | kinesin family member 5B | *Kif5b* | 1.38 | 0.79 |
| 1436550_at | F-box protein 30 | *Fbxo30* | 2.30 | 0.79 |
| 1456041_at | sorting nexin 16 | *Snx16* | 1.73 | 0.79 |
| 1433985_at | abl-interactor 2 | *Abi2* | 1.32 | 0.79 |
| 1433549_x_at | ribosomal protein S21 | *Rps21* | 1.49 | 0.79 |
| 1448268_at | transmembrane emp24 protein transport domain containing 9 | *Tmed9* | 1.55 | 0.79 |
| 1453386_at | tumor suppressor candidate 1 | *Tusc1* | 1.53 | 0.79 |
| 1426205_at | protein phosphatase 1, catalytic subunit, beta isoform | *Ppp1cb* | 1.28 | 0.79 |
| 1429497_s_at | sorting nexin 6 | *Snx6* | 1.30 | 0.79 |
| 1452141_a_at | selenoprotein P, plasma, 1 | *Sepp1* | 1.27 | 0.79 |
| 1452232_at | UDP-N-acetyl-alpha-D-galactosamine: polypeptide N-acetylgalactosaminyltransferase 7 | *Galnt7* | 1.80 | 0.79 |
| 1434690_at | lysocardiolipin acyltransferase 1 | *Lclat1* | 1.24 | 0.79 |
| 1452661_at | vasoactive intestinal polypeptide | *Vip* | 3.12 | 0.79 |
| 1447234_s_at | sorting nexin 6 | *Snx6* | 1.47 | 0.79 |
| 1426476_at | RAS p21 protein activator 1 | *Rasa1* | 1.59 | 0.79 |
| 1434768_at | tripeptidyl peptidase I | *Tpp1* | 1.41 | 0.79 |
| 1424970_at | purine-rich element binding protein G | *Purg* | 1.24 | 0.79 |
| 1448733_at | Bmi1 polycomb ring finger oncogene | *Bmi1* | 1.98 | 0.78 |
| 1449036_at | ring finger protein 128 | *Rnf128* | 1.47 | 0.78 |
| 1448896_at | phosphatidylinositol glycan anchor biosynthesis, class F | *Pigf* | 1.53 | 0.78 |
| 1417126_a_at | ribosomal protein L22 like 1 | *Rpl22l1* | 2.38 | 0.78 |
| 1450986_at | NOP58 ribonucleoprotein homolog (yeast) | *Nop58* | 1.32 | 0.78 |
| 1423607_at | lumican | *Lum* | 2.39 | 0.78 |
| 1417166_at | PC4 and SFRS1 interacting protein 1 | *Psip1* | 1.55 | 0.78 |
| 1419186_a_at | ST8 alpha-N-acetyl-neuraminide alpha-2,8-sialyltransferase 4 | *St8sia4* | 2.22 | 0.78 |
| 1418483_a_at | glycoprotein galactosyltransferase alpha 1, 3 | *Ggta1* | 1.95 | 0.78 |
| 1417541_at | helicase, lymphoid specific | *Hells* | 2.90 | 0.78 |
| 1438300_at | CDKN2A interacting protein | *Cdkn2aip* | 1.51 | 0.78 |
| 1423667_at | methionine adenosyltransferase II, alpha | *Mat2a* | 1.30 | 0.78 |
| 1448655_at | low density lipoprotein receptor-related protein 1 | *Lrp1* | 2.69 | 0.78 |
| 1419252_at | epidermal growth factor receptor pathway substrate 15 | *Eps15* | 1.83 | 0.78 |
| 1425498_at | PRP4 pre-mRNA processing factor 4 homolog B (yeast) | *Prpf4b* | 1.92 | 0.78 |
| 1425034_at | solute carrier family 17 (sodium phosphate), member 2 | *Slc17a2* | 1.42 | 0.78 |
| 1453583_at | zinc finger, ZZ-type with EF hand domain 1 | *Zzef1* | 1.65 | 0.78 |
| 1436995_a_at | ribosomal protein L26 | *Rpl26* | 1.44 | 0.78 |
| 1438606_a_at | chloride intracellular channel 4 (mitochondrial) | *Clic4* | 2.04 | 0.78 |
| 1419194_s_at | glia maturation factor, gamma | *Gmfg* | 1.38 | 0.78 |
| 1453804_a_at | origin recognition complex, subunit 4 | *Orc4* | 1.71 | 0.78 |
| 1441811_x_at | transmembrane protein 176A | *Tmem176a* | 3.80 | 0.78 |
| 1452843_at | interleukin 6 signal transducer | *Il6st* | 2.17 | 0.78 |
| 1448958_at | torsin A interacting protein 2 | *Tor1aip2* | 1.71 | 0.78 |
| 1427075_s_at | protein-L-isoaspartate (D-aspartate) O-methyltransferase domain containing 2 | *Pcmtd2* | 1.22 | 0.78 |
| 1437290_at | inositol monophosphatase domain containing 1 | *Impad1* | 1.31 | 0.78 |
| 1429954_at | C-type lectin domain family 4, member a3 | *Clec4a3* | 1.34 | 0.78 |
| 1434192_at | zinc finger, ZZ-type with EF hand domain 1 | *Zzef1* | 1.51 | 0.78 |
| 1423805_at | disabled homolog 2 (Drosophila) | *Dab2* | 1.45 | 0.78 |
| 1417740_at | cell division cycle 37 homolog (S. cerevisiae)-like 1 | *Cdc37l1* | 1.32 | 0.78 |
| 1416440_at | CD164 antigen | *Cd164* | 1.27 | 0.78 |
| 1427949_at | listerin E3 ubiquitin protein ligase 1 | *Ltn1* | 1.24 | 0.78 |
| 1419914_s_at | nuclear undecaprenyl pyrophosphate synthase 1 homolog (S. cerevisiae) | *Nus1* | 1.72 | 0.78 |
| 1424989_at | ORAI calcium release-activated calcium modulator 1 | *Orai1* | 1.38 | 0.78 |
| 1448937_at | solute carrier family 35, member B3 | *Slc35b3* | 1.93 | 0.78 |
| 1420170_at | myosin, heavy polypeptide 9, non-muscle | *Myh9* | 1.50 | 0.78 |
| 1455321_at | DDHD domain containing 1 | *Ddhd1* | 1.27 | 0.78 |
| 1451018_at | leptin receptor overlapping transcript-like 1 | *Leprotl1* | 1.80 | 0.78 |
| 1454831_at | forkhead box N2 | *Foxn2* | 1.74 | 0.78 |
| 1426946_at | importin 5 | *Ipo5* | 1.46 | 0.78 |
| 1440339_at | ectonucleotide pyrophosphatase/phosphodiesterase 1 | *Enpp1* | 2.21 | 0.78 |
| 1426377_at | zinc finger protein 281 | *Zfp281* | 1.61 | 0.78 |
| 1435834_at | Fanconi anemia, complementation group F | *Fancf* | 1.28 | 0.78 |
| 1437834_s_at | protein kinase C and casein kinase substrate in neurons 3 | *Pacsin3* | 1.72 | 0.78 |
| 1438079_at | zinc finger protein 867 | *Zfp867* | 1.38 | 0.78 |
| 1416807_at | ribosomal protein L36A | *Rpl36a* | 1.44 | 0.78 |
| 1437688_x_at | ATPase, H+ transporting, lysosomal accessory protein 2 | *Atp6ap2* | 2.08 | 0.78 |
| 1454794_at | spastin | *Spast* | 1.30 | 0.78 |
| 1434860_at | dpy-19-like 4 (C. elegans) | *Dpy19l4* | 1.41 | 0.78 |
| 1450690_at | RAN binding protein 2 | *Ranbp2* | 1.79 | 0.78 |
| 1426686_s_at | mitogen-activated protein kinase kinase kinase 3 | *Map3k3* | 1.39 | 0.78 |
| 1428175_at | transmembrane protein 161B | *Tmem161b* | 1.27 | 0.78 |
| 1455142_at | suppressor of cytokine signaling 4 | *Socs4* | 1.28 | 0.78 |
| 1424758_s_at | serine (or cysteine) peptidase inhibitor, clade A (alpha-1 antiproteinase, antitrypsin), member 10 | *Serpina10* | 3.45 | 0.78 |
| 1423167_at | MOB1, Mps One Binder kinase activator-like 3 (yeast) | *Mobkl3* | 1.48 | 0.78 |
| 1423672_at | tetratricopeptide repeat domain 30B | *Ttc30b* | 1.35 | 0.78 |
| 1426598_at | ubiquitously transcribed tetratricopeptide repeat gene, Y chromosome | *Uty* | 2.02 | 0.78 |
| 1423944_at | hemopexin | *Hpx* | 1.54 | 0.78 |
| 1458439_a_at | DAZ interacting protein 3, zinc finger | *Dzip3* | 1.29 | 0.78 |
| 1435450_at | copine III | *Cpne3* | 1.42 | 0.78 |
| 1454993_a_at | serine/arginine-rich splicing factor 3 | *Srsf3* | 1.53 | 0.78 |
| 1418604_at | arginine vasopressin receptor 1A | *Avpr1a* | 1.80 | 0.78 |
| 1455153_at | zinc finger protein 236 | *Zfp236* | 1.85 | 0.78 |
| 1448135_at | activating transcription factor 4 | *Atf4* | 1.42 | 0.78 |
| 1435074_at | transmembrane protein 106B | *Tmem106b* | 1.33 | 0.78 |
| 1452038_at | capping protein (actin filament) muscle Z-line, alpha 1 | *Capza1* | 1.49 | 0.78 |
| 1437347_at | endothelin receptor type B | *Ednrb* | 1.30 | 0.78 |
| 1418236_s_at | autophagy-related 5 (yeast) | *Atg5* | 1.41 | 0.78 |
| 1450941_at | syndecan binding protein | *Sdcbp* | 1.48 | 0.78 |
| 1422557_s_at | metallothionein 1 | *Mt1* | 46.49 | 0.78 |
| 1417791_a_at | zinc finger, matrin-like | *Zfml* | 1.51 | 0.78 |
| 1422460_at | MAD2 mitotic arrest deficient-like 1 (yeast) | *Mad2l1* | 2.52 | 0.78 |
| 1420459_at | ripply3 homolog (zebrafish) | *Ripply3* | 1.44 | 0.78 |
| 1438945_x_at | gap junction protein, alpha 1 | *Gja1* | 3.67 | 0.78 |
| 1447830_s_at | regulator of G-protein signaling 2 | *Rgs2* | 2.58 | 0.78 |
| 1421946_at | C-reactive protein, pentraxin-related | *Crp* | 1.49 | 0.78 |
| 1424599_at | fibrinogen-like protein 1 | *Fgl1* | 9.24 | 0.78 |
| 1448558_a_at | phospholipase A2, group IVA (cytosolic, calcium-dependent) | *Pla2g4a* | 3.94 | 0.78 |
| 1416958_at | nuclear receptor subfamily 1, group D, member 2 | *Nr1d2* | 1.38 | 0.78 |
| 1425255_s_at | heterogeneous nuclear ribonucleoprotein L-like | *Hnrpll* | 1.38 | 0.78 |
| 1417183_at | DnaJ (Hsp40) homolog, subfamily A, member 2 | *Dnaja2* | 1.42 | 0.78 |
| 1430147_a_at | TATA box binding protein (Tbp)-associated factor, RNA polymerase I, D | *Taf1d* | 2.09 | 0.78 |
| 1456628_x_at | ribosomal protein S24 | *Rps24* | 1.31 | 0.78 |
| 1448823_at | chemokine (C-X-C motif) ligand 12 | *Cxcl12* | 1.98 | 0.78 |
| 1448208_at | MAD homolog 1 (Drosophila) | *Smad1* | 1.53 | 0.78 |
| 1426536_at | NMDA receptor-regulated gene 2 | *Narg2* | 1.47 | 0.78 |
| 1448647_at | mannosidase 2, alpha 1 | *Man2a1* | 1.42 | 0.78 |
| 1416557_a_at | elongation factor Tu GTP binding domain containing 2 | *Eftud2* | 1.26 | 0.78 |
| 1434778_at | wings apart-like homolog (Drosophila) | *Wapal* | 1.42 | 0.78 |
| 1438951_x_at | nucleoporin 54 | *Nup54* | 1.56 | 0.78 |
| 1427305_at | phosphatidylinositol glycan anchor biosynthesis, class A | *Piga* | 1.45 | 0.78 |
| 1434380_at | guanylate binding protein 6 | *Gbp6* | 1.82 | 0.78 |
| 1439030_at | GDP-mannose pyrophosphorylase B | *Gmppb* | 1.93 | 0.78 |
| 1429005_at | malignant fibrous histiocytoma amplified sequence 1 | *Mfhas1* | 1.50 | 0.78 |
| 1438992_x_at | activating transcription factor 4 | *Atf4* | 1.51 | 0.78 |
| 1426569_a_at | fyn-related kinase | *Frk* | 1.33 | 0.78 |
| 1434949_at | armadillo repeat containing 8 | *Armc8* | 3.23 | 0.78 |
| 1432384_a_at | methyltransferase like 6 | *Mettl6* | 1.32 | 0.78 |
| 1428677_at | WD repeat domain 73 | *Wdr73* | 1.34 | 0.78 |
| 1415870_at | calumenin | *Calu* | 2.05 | 0.78 |
| 1447903_x_at | adaptor-related protein complex 1, sigma 2 subunit | *Ap1s2* | 2.24 | 0.78 |
| 1433772_at | heat shock protein 70 family, member 13 | *Hspa13* | 1.91 | 0.78 |
| 1429710_at | serine/threonine/tyrosine interaction protein | *Styx* | 1.22 | 0.78 |
| 1434694_at | leucine rich repeat containing 8A | *Lrrc8a* | 1.42 | 0.78 |
| 1452739_at | F-box protein 7 | *Fbxo7* | 1.30 | 0.77 |
| 1448536_at | LSM3 homolog, U6 small nuclear RNA associated (S. cerevisiae) | *Lsm3* | 1.26 | 0.77 |
| 1422487_at | MAD homolog 4 (Drosophila) | *Smad4* | 1.27 | 0.77 |
| 1456388_at | ATPase, class VI, type 11A | *Atp11a* | 3.57 | 0.77 |
| 1437723_s_at | Der1-like domain family, member 1 | *Derl1* | 1.26 | 0.77 |
| 1415897_a_at | microsomal glutathione S-transferase 1 | *Mgst1* | 1.34 | 0.77 |
| 1417441_at | DnaJ (Hsp40) homolog, subfamily C, member 12 | *Dnajc12* | 10.72 | 0.77 |
| 1427990_at | ubiquitin specific petidase 45 | *Usp45* | 1.80 | 0.77 |
| 1423369_at | fragile X mental retardation syndrome 1 homolog | *Fmr1* | 1.67 | 0.77 |
| 1451639_at | CCAAT/enhancer binding protein (C/EBP), gamma | *Cebpg* | 1.83 | 0.77 |
| 1418530_at | nucleoporin 160 | *Nup160* | 1.33 | 0.77 |
| 1460547_a_at | heterogeneous nuclear ribonucleoprotein K | *Hnrnpk* | 1.47 | 0.77 |
| 1426769_s_at | mastermind like 1 (Drosophila) | *Maml1* | 1.21 | 0.77 |
| 1434187_at | asparagine-linked glycosylation 11 homolog (yeast, alpha-1,2-mannosyltransferase) | *Alg11* | 1.87 | 0.77 |
| 1449095_at | vacuolar protein sorting 54 (yeast) | *Vps54* | 1.44 | 0.77 |
| 1427006_at | Rap guanine nucleotide exchange factor (GEF) 1 | *Rapgef1* | 1.70 | 0.77 |
| 1417307_at | dystrophin, muscular dystrophy | *Dmd* | 1.26 | 0.77 |
| 1453230_at | zinc finger protein 74 | *Zfp74* | 1.44 | 0.77 |
| 1428772_at | exportin, tRNA (nuclear export receptor for tRNAs) | *Xpot* | 1.37 | 0.77 |
| 1416779_at | serum deprivation response | *Sdpr* | 1.38 | 0.77 |
| 1447852_x_at | Rab interacting lysosomal protein-like 1 | *Rilpl1* | 1.30 | 0.77 |
| 1434945_at | lysophosphatidylcholine acyltransferase 2 | *Lpcat2* | 1.58 | 0.77 |
| 1437634_at | THO complex 2 | *Thoc2* | 1.24 | 0.77 |
| 1422498_at | melanoma antigen, family H, 1 | *Mageh1* | 1.86 | 0.77 |
| 1431721_a_at | protein Z, vitamin K-dependent plasma glycoprotein | *Proz* | 1.47 | 0.77 |
| 1439266_a_at | polymerase (RNA) III (DNA directed) polypeptide K | *Polr3k* | 1.60 | 0.77 |
| 1424215_at | FUN14 domain containing 1 | *Fundc1* | 1.49 | 0.77 |
| 1450137_at | protein O-fucosyltransferase 1 | *Pofut1* | 1.28 | 0.77 |
| 1436762_x_at | Elongation protein 3 homolog (S. cerevisiae) | *Elp3* | 1.21 | 0.77 |
| 1421375_a_at | S100 calcium binding protein A6 (calcyclin) | *S100a6* | 2.13 | 0.77 |
| 1427229_at | 3-hydroxy-3-methylglutaryl-Coenzyme A reductase | *Hmgcr* | 1.39 | 0.77 |
| 1417654_at | syndecan 4 | *Sdc4* | 1.67 | 0.77 |
| 1425553_s_at | huntingtin interacting protein 1 related | *Hip1r* | 1.44 | 0.77 |
| 1434547_at | carboxypeptidase D | *Cpd* | 1.92 | 0.77 |
| 1452125_at | thyroid hormone receptor associated protein 3 | *Thrap3* | 1.63 | 0.77 |
| 1428786_at | NCK associated protein 1 like | *Nckap1l* | 1.42 | 0.77 |
| 1419975_at | Sterol carrier protein 2, liver | *Scp2* | 1.41 | 0.77 |
| 1416093_a_at | mitochondrial ribosomal protein L20 | *Mrpl20* | 1.24 | 0.77 |
| 1452352_at | cytotoxic T lymphocyte-associated protein 2 beta | *Ctla2b* | 4.13 | 0.77 |
| 1459737_s_at | transthyretin | *Ttr* | 1.22 | 0.77 |
| 1438037_at | hect domain and RLD 6 | *Herc6* | 2.26 | 0.77 |
| 1427185_at | myocyte enhancer factor 2A | *Mef2a* | 1.33 | 0.77 |
| 1450136_at | CD38 antigen | *Cd38* | 2.20 | 0.77 |
| 1440300_at | vav 1 oncogene | *Vav1* | 1.29 | 0.77 |
| 1423982_at | serine/arginine-rich splicing factor 10 | *Srsf10* | 2.47 | 0.77 |
| 1416423_x_at | Sjogren syndrome antigen B | *Ssb* | 1.59 | 0.77 |
| 1419595_a_at | gamma-glutamyl hydrolase | *Ggh* | 1.65 | 0.77 |
| 1435303_at | TAF4B RNA polymerase II, TATA box binding protein (TBP)-associated factor | *Taf4b* | 1.65 | 0.77 |
| 1426597_s_at | intermediate filament family orphan 2 | *Iffo2* | 1.87 | 0.77 |
| 1417410_s_at | protein kinase C, iota | *Prkci* | 1.21 | 0.77 |
| 1448893_at | nuclear receptor co-repressor 2 | *Ncor2* | 1.32 | 0.77 |
| 1423455_at | prothymosin alpha | *Ptma* | 1.47 | 0.77 |
| 1452143_at | spectrin beta 2 | *Spnb2* | 1.29 | 0.77 |
| 1446834_at | Cathepsin C | *Ctsc* | 3.61 | 0.77 |
| 1434036_at | metastasis suppressor 1 | *Mtss1* | 1.23 | 0.77 |
| 1460357_at | YTH domain family 2 | *Ythdf2* | 1.61 | 0.77 |
| 1448734_at | arachidonate 5-lipoxygenase activating protein | *Alox5ap* | 2.27 | 0.77 |
| 1449333_at | splicing factor 3a, subunit 1 | *Sf3a1* | 1.65 | 0.77 |
| 1456500_at | anterior pharynx defective 1b homolog (C. elegans) | *Aph1b* | 2.43 | 0.77 |
| 1438026_at | zinc finger protein 560 | *Zfp560* | 1.43 | 0.77 |
| 1439405_x_at | Cyclin N-terminal domain containing 1 | *Cntd1* | 1.49 | 0.77 |
| 1434423_at | GULP, engulfment adaptor PTB domain containing 1 | *Gulp1* | 1.38 | 0.77 |
| 1435926_at | choroideremia-like | *Chml* | 3.14 | 0.77 |
| 1454947_a_at | ubiquitin-like domain containing CTD phosphatase 1 | *Ublcp1* | 1.28 | 0.77 |
| 1451652_a_at | transmembrane protein 188 | *Tmem188* | 1.40 | 0.77 |
| 1460428_at | ankyrin repeat domain 13a | *Ankrd13a* | 1.22 | 0.77 |
| 1422818_at | neural precursor cell expressed, developmentally down-regulated gene 9 | *Nedd9* | 1.66 | 0.77 |
| 1419044_at | contactin associated protein-like 4 | *Cntnap4* | 1.30 | 0.77 |
| 1426759_at | mitogen-activated protein kinase kinase kinase kinase 3 | *Map4k3* | 1.55 | 0.77 |
| 1451146_at | zinc finger protein 386 (Kruppel-like) | *Zfp386* | 1.33 | 0.77 |
| 1448025_at | transmembrane protein 173 | *Tmem173* | 4.04 | 0.76 |
| 1455729_at | guanine nucleotide binding protein, alpha q polypeptide | *Gnaq* | 1.43 | 0.76 |
| 1456212_x_at | suppressor of cytokine signaling 3 | *Socs3* | 2.99 | 0.76 |
| 1449446_at | coiled-coil domain containing 59 | *Ccdc59* | 1.33 | 0.76 |
| 1426642_at | fibronectin 1 | *Fn1* | 1.59 | 0.76 |
| 1424254_at | acyl-CoA synthetase long-chain family member 4 | *Acsl4* | 1.78 | 0.76 |
| 1435251_at | sorting nexin 13 | *Snx13* | 1.23 | 0.76 |
| 1417535_at | F-box protein 25 | *Fbxo25* | 1.39 | 0.76 |
| 1439226_at | dedicator of cytokinesis 8 | *Dock8* | 1.52 | 0.76 |
| 1435476_a_at | Fc receptor, IgG, low affinity IIb | *Fcgr2b* | 1.38 | 0.76 |
| 1453582_at | choline kinase alpha | *Chka* | 1.44 | 0.76 |
| 1448213_at | annexin A1 | *Anxa1* | 1.98 | 0.76 |
| 1454976_at | superoxide dismutase 2, mitochondrial | *Sod2* | 1.36 | 0.76 |
| 1416625_at | serine (or cysteine) peptidase inhibitor, clade G, member 1 | *Serping1* | 1.77 | 0.76 |
| 1452422_a_at | U2 small nuclear ribonucleoprotein B | *Snrpb2* | 1.29 | 0.76 |
| 1436356_at | sterile alpha motif domain containing 4 | *Samd4* | 1.59 | 0.76 |
| 1417023_a_at | fatty acid binding protein 4, adipocyte | *Fabp4* | 2.57 | 0.76 |
| 1433937_at | transformation related protein 53 binding protein 2 | *Trp53bp2* | 1.24 | 0.76 |
| 1452758_s_at | eukaryotic translation initiation factor 4, gamma 2 | *Eif4g2* | 1.67 | 0.76 |
| 1429787_x_at | ZW10 interactor | *Zwint* | 1.23 | 0.76 |
| 1437382_at | activin receptor IIA | *Acvr2a* | 1.28 | 0.76 |
| 1416345_at | translocase of inner mitochondrial membrane 8 homolog a1 (yeast) | *Timm8a1* | 1.27 | 0.76 |
| 1454990_at | AT rich interactive domain 2 (ARID, RFX-like) | *Arid2* | 1.59 | 0.76 |
| 1449579_at | Sh3 domain YSC-like 1 | *Sh3yl1* | 1.21 | 0.76 |
| 1434735_at | hepatic leukemia factor | *Hlf* | 1.40 | 0.76 |
| 1450891_at | signal recognition particle 19 | *Srp19* | 1.26 | 0.76 |
| 1435384_at | ubiquitin-conjugating enzyme E2N | *Ube2n* | 2.09 | 0.76 |
| 1417508_at | ring finger protein 19A | *Rnf19a* | 1.41 | 0.76 |
| 1428613_at | lactate dehydrogenase D | *Ldhd* | 1.21 | 0.76 |
| 1435001_at | phospholipase A2, activating protein | *Plaa* | 2.20 | 0.76 |
| 1460456_at | prolyl-tRNA synthetase domain containing 1 | *Prorsd1* | 1.46 | 0.76 |
| 1430133_at | TBC1 domain family, member 8B | *Tbc1d8b* | 1.91 | 0.76 |
| 1460558_at | coiled-coil domain containing 32 | *Ccdc32* | 1.29 | 0.76 |
| 1452700_s_at | kelch repeat and BTB (POZ) domain containing 7 | *Kbtbd7* | 1.86 | 0.76 |
| 1460578_at | FYVE, RhoGEF and PH domain containing 5 | *Fgd5* | 1.60 | 0.76 |
| 1433576_at | methionine adenosyltransferase II, alpha | *Mat2a* | 1.49 | 0.76 |
| 1424826_s_at | metastasis suppressor 1 | *Mtss1* | 1.27 | 0.76 |
| 1460211_a_at | KDEL (Lys-Asp-Glu-Leu) endoplasmic reticulum protein retention receptor 1 | *Kdelr1* | 1.44 | 0.76 |
| 1433528_at | general transcription factor II A, 2 | *Gtf2a2* | 2.36 | 0.76 |
| 1427125_s_at | leucine rich repeat containing 41 | *Lrrc41* | 1.66 | 0.76 |
| 1423796_at | splicing factor proline/glutamine rich (polypyrimidine tract binding protein associated) | *Sfpq* | 2.30 | 0.76 |
| 1449002_at | pleckstrin homology-like domain, family A, member 3 | *Phlda3* | 2.67 | 0.76 |
| 1423649_at | transmembrane protein 68 | *Tmem68* | 1.42 | 0.76 |
| 1427983_at | zinc finger protein 280C | *Zfp280c* | 1.32 | 0.76 |
| 1460628_at | essential meiotic endonuclease 1 homolog 2 (S. pombe) | *Eme2* | 1.62 | 0.76 |
| 1455002_at | protein tyrosine phosphatase 4a1 | *Ptp4a1* | 1.78 | 0.76 |
| 1423391_at | G protein-coupled receptor kinase-interactor 2 | *Git2* | 1.26 | 0.76 |
| 1417818_at | WW domain containing transcription regulator 1 | *Wwtr1* | 1.42 | 0.76 |
| 1436405_at | dedicator of cytokinesis 4 | *Dock4* | 2.06 | 0.76 |
| 1424562_a_at | solute carrier family 25 (mitochondrial carrier, adenine nucleotide translocator), member 4 | *Slc25a4* | 1.77 | 0.76 |
| 1423954_at | phospholipase A1 member A | *Pla1a* | 2.09 | 0.76 |
| 1416944_a_at | tousled-like kinase 2 (Arabidopsis) | *Tlk2* | 1.21 | 0.76 |
| 1433514_at | ethanolamine kinase 1 | *Etnk1* | 1.85 | 0.76 |
| 1426018_a_at | SRY-box containing gene 6 | *Sox6* | 1.77 | 0.76 |
| 1434513_at | ATPase type 13A3 | *Atp13a3* | 1.37 | 0.76 |
| 1445597_s_at | phospholipase A2, group XVI | *Pla2g16* | 1.65 | 0.76 |
| 1451193_x_at | tetratricopeptide repeat domain 4 | *Ttc4* | 1.50 | 0.76 |
| 1417537_at | zinc finger, matrin type 2 | *Zmat2* | 1.45 | 0.76 |
| 1434956_at | ring finger protein 170 | *Rnf170* | 1.67 | 0.76 |
| 1429763_at | cornichon homolog 4 (Drosophila) | *Cnih4* | 1.21 | 0.76 |
| 1449455_at | hemopoietic cell kinase | *Hck* | 3.12 | 0.76 |
| 1419298_at | paraoxonase 3 | *Pon3* | 1.22 | 0.76 |
| 1428231_at | cleavage and polyadenylation specific factor 6 | *Cpsf6* | 1.71 | 0.76 |
| 1454666_at | Kruppel-like factor 3 (basic) | *Klf3* | 1.43 | 0.76 |
| 1428219_at | RING1 and YY1 binding protein | *Rybp* | 1.28 | 0.76 |
| 1423248_at | natural killer tumor recognition sequence | *Nktr* | 1.31 | 0.76 |
| 1423918_at | rhomboid domain containing 1 | *Rhbdd1* | 1.23 | 0.76 |
| 1425260_at | albumin | *Alb* | 1.27 | 0.76 |
| 1426903_at | fibronectin type III domain containing 3A | *Fndc3a* | 2.06 | 0.76 |
| 1437256_at | DCN1, defective in cullin neddylation 1, domain containing 5 (S. cerevisiae) | *Dcun1d5* | 1.29 | 0.76 |
| 1438377_x_at | solute carrier family 13 (sodium-dependent dicarboxylate transporter), member 3 | *Slc13a3* | 2.40 | 0.76 |
| 1426426_at | MAK16 homolog (S. cerevisiae) | *Mak16* | 1.28 | 0.76 |
| 1448529_at | thrombomodulin | *Thbd* | 1.59 | 0.76 |
| 1425010_at | zinc finger protein 119a | *Zfp119a* | 1.82 | 0.76 |
| 1420986_s_at | kinesin family member 3B | *Kif3b* | 1.22 | 0.76 |
| 1455643_s_at | TSR1, 20S rRNA accumulation, homolog (yeast) | *Tsr1* | 1.35 | 0.76 |
| 1448555_at | RNA polymerase II associated protein 3 | *Rpap3* | 11.02 | 0.76 |
| 1452665_at | tetratricopeptide repeat domain 27 | *Ttc27* | 1.28 | 0.76 |
| 1417010_at | zinc finger protein 238 | *Zfp238* | 1.63 | 0.76 |
| 1451459_at | AT hook containing transcription factor 1 | *Ahctf1* | 1.47 | 0.76 |
| 1428754_at | tRNA methyltransferase 6 homolog (S. cerevisiae) | *Trmt6* | 1.60 | 0.76 |
| 1421935_at | ribosomal protein S20 | *Rps20* | 1.32 | 0.76 |
| 1421871_at | SH3-binding domain glutamic acid-rich protein like | *Sh3bgrl* | 1.76 | 0.76 |
| 1451327_a_at | transmembrane emp24 domain containing 1 | *Tmed1* | 1.38 | 0.76 |
| 1435235_at | thioredoxin-like 1 | *Txnl1* | 1.51 | 0.76 |
| 1456218_at | sorting nexin 22 | *Snx22* | 2.33 | 0.76 |
| 1433622_at | gem (nuclear organelle) associated protein 4 | *Gemin4* | 1.35 | 0.76 |
| 1417090_at | reticulocalbin 1 | *Rcn1* | 1.58 | 0.76 |
| 1433960_at | interferon stimulated exonuclease gene 20-like 2 | *Isg20l2* | 1.22 | 0.76 |
| 1421094_at | zinc finger and BTB domain containing 33 | *Zbtb33* | 1.33 | 0.76 |
| 1417068_a_at | protein tyrosine phosphatase, non-receptor type 1 | *Ptpn1* | 1.65 | 0.76 |
| 1425082_s_at | PDS5, regulator of cohesion maintenance, homolog A (S. cerevisiae) | *Pds5a* | 1.55 | 0.76 |
| 1429491_s_at | Rap1 interacting factor 1 homolog (yeast) | *Rif1* | 2.04 | 0.76 |
| 1448919_at | CD302 antigen | *Cd302* | 2.24 | 0.76 |
| 1424642_at | THO complex 1 | *Thoc1* | 1.75 | 0.76 |
| 1448136_at | ectonucleotide pyrophosphatase/phosphodiesterase 2 | *Enpp2* | 1.54 | 0.75 |
| 1451621_at | PPPDE peptidase domain containing 1 | *Pppde1* | 1.83 | 0.75 |
| 1416530_a_at | purine-nucleoside phosphorylase | *Pnp* | 2.01 | 0.75 |
| 1434541_x_at | KH domain containing, RNA binding, signal transduction associated 1 | *Khdrbs1* | 1.36 | 0.75 |
| 1455083_at | ATPase, class VI, type 11C | *Atp11c* | 1.62 | 0.75 |
| 1437671_x_at | protease, serine, 23 | *Prss23* | 1.43 | 0.75 |
| 1440348_at | zinc finger, FYVE domain containing 9 | *Zfyve9* | 1.43 | 0.75 |
| 1449519_at | growth arrest and DNA-damage-inducible 45 alpha | *Gadd45a* | 1.41 | 0.75 |
| 1420613_at | protein tyrosine phosphatase 4a2 | *Ptp4a2* | 1.24 | 0.75 |
| 1424594_at | sterile alpha motif domain containing 4 | *Samd4* | 1.33 | 0.75 |
| 1451355_at | alkaline ceramidase 2 | *Acer2* | 2.17 | 0.75 |
| 1433500_at | DENN/MADD domain containing 2A | *Dennd2a* | 1.23 | 0.75 |
| 1449055_x_at | poly(rC) binding protein 4 | *Pcbp4* | 1.86 | 0.75 |
| 1418410_at | jerky | *Jrk* | 2.99 | 0.75 |
| 1450454_at | torsin family 3, member A | *Tor3a* | 1.45 | 0.75 |
| 1448026_at | chromodomain helicase DNA binding protein 7 | *Chd7* | 1.59 | 0.75 |
| 1436509_at | malectin | *Mlec* | 1.95 | 0.75 |
| 1448898_at | plasminogen activator, tissue | *Plat* | 1.55 | 0.75 |
| 1418515_at | metal response element binding transcription factor 2 | *Mtf2* | 1.37 | 0.75 |
| 1433623_at | zinc finger protein 367 | *Zfp367* | 1.32 | 0.75 |
| 1437176_at | NLR family, CARD domain containing 5 | *Nlrc5* | 1.87 | 0.75 |
| 1422704_at | glycerol kinase | *Gyk* | 1.73 | 0.75 |
| 1427131_s_at | leucine rich repeat containing 58 | *Lrrc58* | 1.27 | 0.75 |
| 1434174_at | LysM, putative peptidoglycan-binding, domain containing 3 | *Lysmd3* | 2.11 | 0.75 |
| 1449326_x_at | serum amyloid A 2 | *Saa2* | 25.07 | 0.75 |
| 1428011_a_at | Erbb2 interacting protein | *Erbb2ip* | 1.33 | 0.75 |
| 1456544_at | transmembrane protein 38B | *Tmem38b* | 1.82 | 0.75 |
| 1429502_at | heat shock protein 70 family, member 13 | *Hspa13* | 1.45 | 0.75 |
| 1436540_at | microRNA let7d | *Mirlet7d* | 1.35 | 0.75 |
| 1442027_at | neurobeachin like 1 | *Nbeal1* | 1.38 | 0.75 |
| 1435306_a_at | kinesin family member 11 | *Kif11* | 2.17 | 0.75 |
| 1423870_at | axin interactor, dorsalization associated | *Aida* | 1.48 | 0.75 |
| 1439456_x_at | ATPase, H+ transporting, lysosomal accessory protein 2 | *Atp6ap2* | 2.22 | 0.75 |
| 1437748_at | fucosyltransferase 11 | *Fut11* | 1.55 | 0.75 |
| 1452856_at | CREB/ATF bZIP transcription factor | *Crebzf* | 1.34 | 0.75 |
| 1427439_s_at | protein arginine N-methyltransferase 5 | *Prmt5* | 1.57 | 0.75 |
| 1456070_at | protein tyrosine phosphatase, receptor type, G | *Ptprg* | 1.33 | 0.75 |
| 1428126_a_at | ataxin 7-like 3B | *Atxn7l3b* | 1.58 | 0.75 |
| 1434000_at | v-Ki-ras2 Kirsten rat sarcoma viral oncogene homolog | *Kras* | 1.45 | 0.75 |
| 1435023_at | intersectin 2 | *Itsn2* | 1.79 | 0.75 |
| 1416061_at | TBC1 domain family, member 15 | *Tbc1d15* | 1.35 | 0.75 |
| 1456626_a_at | SAP domain containing ribonucleoprotein | *Sarnp* | 1.40 | 0.75 |
| 1451292_at | Zinc finger protein 212 | *Zfp212* | 1.28 | 0.75 |
| 1460649_at | interleukin-1 receptor-associated kinase 1 | *Irak1* | 1.34 | 0.75 |
| 1450027_at | syndecan 3 | *Sdc3* | 1.50 | 0.75 |
| 1452991_at | chromodomain helicase DNA binding protein 2 | *Chd2* | 1.83 | 0.75 |
| 1436451_a_at | transmembrane emp24 domain trafficking protein 2 | *Tmed2* | 1.34 | 0.75 |
| 1419917_s_at | transmembrane emp24 protein transport domain containing 7 | *Tmed7* | 1.51 | 0.75 |
| 1429080_at | M-phase phosphoprotein 10 (U3 small nucleolar ribonucleoprotein) | *Mphosph10* | 1.44 | 0.75 |
| 1460036_at | adaptor-related protein complex 1, sigma 2 subunit | *Ap1s2* | 1.72 | 0.75 |
| 1438983_x_at | VPS33B interacting protein, apical-basolateral polarity regulator | *Vipar* | 1.51 | 0.75 |
| 1433521_at | ankyrin repeat domain 13c | *Ankrd13c* | 1.32 | 0.75 |
| 1434211_at | SH3 domain binding glutamic acid-rich protein like 2 | *Sh3bgrl2* | 1.53 | 0.75 |
| 1452880_at | zinc finger, HIT type 3 | *Znhit3* | 1.82 | 0.75 |
| 1421686_at | neuropeptide VF precursor | *Npvf* | 1.45 | 0.75 |
| 1419925_s_at | thioredoxin-related transmembrane protein 3 | *Tmx3* | 1.37 | 0.75 |
| 1428369_s_at | Rho GTPase activating protein 21 | *Arhgap21* | 1.39 | 0.75 |
| 1419753_at | nuclear transcription factor, X-box binding 1 | *Nfx1* | 1.26 | 0.75 |
| 1434571_at | vacuolar protein sorting 13B (yeast) | *Vps13b* | 1.24 | 0.75 |
| 1419436_at | complement factor H-related 1 | *Cfhr1* | 1.38 | 0.75 |
| 1433527_at | iron responsive element binding protein 2 | *Ireb2* | 1.37 | 0.75 |
| 1419162_s_at | DnaJ (Hsp40) homolog, subfamily C, member 3 | *Dnajc3* | 1.41 | 0.75 |
| 1439350_s_at | phosphatidylinositol glycan anchor biosynthesis, class U | *Pigu* | 1.31 | 0.75 |
| 1425058_at | zinc finger protein 472 | *Zfp472* | 1.97 | 0.75 |
| 1437016_x_at | RAP2C, member of RAS oncogene family | *Rap2c* | 1.23 | 0.75 |
| 1439015_at | glial cell line derived neurotrophic factor family receptor alpha 1 | *Gfra1* | 2.22 | 0.74 |
| 1434224_at | transducin (beta)-like 2 | *Tbl2* | 1.59 | 0.74 |
| 1455795_at | dermatan sulfate epimerase | *Dse* | 1.71 | 0.74 |
| 1433473_x_at | TM2 domain containing 2 | *Tm2d2* | 1.39 | 0.74 |
| 1428845_at | BCL2-associated transcription factor 1 | *Bclaf1* | 1.28 | 0.74 |
| 1454951_at | zinc finger protein 606 | *Zfp606* | 1.49 | 0.74 |
| 1450858_a_at | ubiquitin-conjugating enzyme E2D 3 (UBC4/5 homolog, yeast) | *Ube2d3* | 1.23 | 0.74 |
| 1452197_at | structural maintenance of chromosomes 4 | *Smc4* | 1.88 | 0.74 |
| 1415889_a_at | heat shock protein 90, beta (Grp94), member 1 | *Hsp90b1* | 1.64 | 0.74 |
| 1437932_a_at | claudin 1 | *Cldn1* | 1.79 | 0.74 |
| 1429521_at | alkB, alkylation repair homolog 8 (E. coli) | *Alkbh8* | 1.65 | 0.74 |
| 1418022_at | N(alpha)-acetyltransferase 15, NatA auxiliary subunit | *Naa15* | 1.98 | 0.74 |
| 1460576_at | exocyst complex component 6 | *Exoc6* | 1.31 | 0.74 |
| 1417365_a_at | calmodulin 1 | *Calm1* | 1.22 | 0.74 |
| 1433934_at | Sec24 related gene family, member A (S. cerevisiae) | *Sec24a* | 1.41 | 0.74 |
| 1433540_x_at | protein phosphatase 1, catalytic subunit, beta isoform | *Ppp1cb* | 1.34 | 0.74 |
| 1427089_at | cyclin T2 | *Ccnt2* | 2.02 | 0.74 |
| 1419508_at | receptor (TNFRSF)-interacting serine-threonine kinase 1 | *Ripk1* | 1.47 | 0.74 |
| 1417871_at | hydroxysteroid (17-beta) dehydrogenase 7 | *Hsd17b7* | 1.39 | 0.74 |
| 1451077_at | 60S ribosomal protein L5-like | *LOC100503670* | 1.26 | 0.74 |
| 1436816_at | nucleoporin 133 | *Nup133* | 1.45 | 0.74 |
| 1419622_at | UDP glucuronosyltransferase 2 family, polypeptide B5 | *Ugt2b5* | 1.28 | 0.74 |
| 1450853_at | transducin-like enhancer of split 4, homolog of Drosophila E(spl) | *Tle4* | 1.60 | 0.74 |
| 1444478_at | adaptor protein, phosphotyrosine interaction, PH domain and leucine zipper containing 1 | *Appl1* | 1.40 | 0.74 |
| 1434088_at | zinc finger with KRAB and SCAN domains 17 | *Zkscan17* | 1.25 | 0.74 |
| 1450051_at | alpha thalassemia/mental retardation syndrome X-linked homolog (human) | *Atrx* | 1.62 | 0.74 |
| 1421333_a_at | myoneurin | *Mynn* | 1.31 | 0.74 |
| 1428094_at | lysosomal-associated membrane protein 2 | *Lamp2* | 1.46 | 0.74 |
| 1425114_at | retinoblastoma binding protein 6 | *Rbbp6* | 1.50 | 0.74 |
| 1426266_s_at | zinc finger and BTB domain containing 8 opposite strand | *Zbtb8os* | 1.25 | 0.74 |
| 1448764_a_at | fatty acid binding protein 1, liver | *Fabp1* | 1.59 | 0.74 |
| 1428942_at | metallothionein 2 | *Mt2* | 70.84 | 0.74 |
| 1449306_at | heat shock factor 2 | *Hsf2* | 2.89 | 0.74 |
| 1426666_a_at | Sad1 and UNC84 domain containing 1 | *Sun1* | 1.41 | 0.74 |
| 1427269_at | serine/arginine-rich splicing factor 11 | *Srsf11* | 1.31 | 0.74 |
| 1450105_at | a disintegrin and metallopeptidase domain 10 | *Adam10* | 1.67 | 0.74 |
| 1434019_at | PDGFA associated protein 1 | *Pdap1* | 1.29 | 0.74 |
| 1449885_at | transmembrane protein 47 | *Tmem47* | 1.72 | 0.74 |
| 1459860_x_at | tripartite motif-containing 2 | *Trim2* | 1.50 | 0.74 |
| 1434569_at | transcriptional adaptor 2B | *Tada2b* | 1.38 | 0.74 |
| 1454641_at | CGG triplet repeat binding protein 1 | *Cggbp1* | 1.97 | 0.74 |
| 1417495_x_at | ceruloplasmin | *Cp* | 2.47 | 0.74 |
| 1454691_at | neurexin I | *Nrxn1* | 1.71 | 0.74 |
| 1425537_at | protein phosphatase 1A, magnesium dependent, alpha isoform | *Ppm1a* | 1.30 | 0.74 |
| 1460242_at | CD55 antigen | *Cd55* | 2.47 | 0.74 |
| 1423535_at | striatin, calmodulin binding protein 3 | *Strn3* | 1.62 | 0.74 |
| 1418640_at | sirtuin 1 (silent mating type information regulation 2, homolog) 1 (S. cerevisiae) | *Sirt1* | 1.96 | 0.74 |
| 1428616_at | zinc finger protein 131 | *Zfp131* | 1.47 | 0.74 |
| 1418762_at | CD55 antigen | *Cd55* | 2.24 | 0.74 |
| 1416859_at | FK506 binding protein 3 | *Fkbp3* | 1.38 | 0.74 |
| 1425598_a_at | Yamaguchi sarcoma viral (v-yes-1) oncogene homolog | *Lyn* | 1.51 | 0.74 |
| 1423423_at | protein disulfide isomerase associated 3 | *Pdia3* | 1.56 | 0.74 |
| 1427405_s_at | RAB11 family interacting protein 5 (class I) | *Rab11fip5* | 1.41 | 0.74 |
| 1452646_at | transformation related protein 53 inducible nuclear protein 2 | *Trp53inp2* | 1.20 | 0.74 |
| 1439411_a_at | exportin 7 | *Xpo7* | 1.22 | 0.74 |
| 1428079_at | fibrinogen beta chain | *Fgb* | 1.84 | 0.74 |
| 1455332_x_at | Fc receptor, IgG, low affinity IIb | *Fcgr2b* | 1.68 | 0.74 |
| 1428187_at | CD47 antigen (Rh-related antigen, integrin-associated signal transducer) | *Cd47* | 1.36 | 0.74 |
| 1434070_at | jagged 1 | *Jag1* | 1.71 | 0.74 |
| 1445882_at | CD300 antigen like family member B | *Cd300lb* | 1.39 | 0.74 |
| 1418209_a_at | profilin 2 | *Pfn2* | 2.31 | 0.74 |
| 1460591_at | estrogen receptor 1 (alpha) | *Esr1* | 1.71 | 0.74 |
| 1434484_at | chemokine (C-X-C motif) ligand 9 | *Cxcl9* | 1.75 | 0.74 |
| 1416985_at | signal-regulatory protein alpha | *Sirpa* | 1.65 | 0.74 |
| 1430520_at | copine VIII | *Cpne8* | 4.23 | 0.74 |
| 1426405_at | ring finger protein 11 | *Rnf11* | 1.72 | 0.74 |
| 1447898_s_at | serine/arginine-rich splicing factor 6 | *Srsf6* | 1.98 | 0.74 |
| 1446325_at | prenylcysteine oxidase 1 | *Pcyox1* | 1.96 | 0.74 |
| 1454963_at | phosphodiesterase 12 | *Pde12* | 1.70 | 0.74 |
| 1429193_at | ankyrin repeat and IBR domain containing 1 | *Ankib1* | 1.43 | 0.74 |
| 1419976_s_at | nuclear factor of activated T-cells, cytoplasmic, calcineurin-dependent 3 | *Nfatc3* | 1.37 | 0.74 |
| 1421818_at | B-cell leukemia/lymphoma 6 | *Bcl6* | 1.86 | 0.74 |
| 1416497_at | protein disulfide isomerase associated 4 | *Pdia4* | 2.38 | 0.74 |
| 1434846_at | DENN/MADD domain containing 4C | *Dennd4c* | 1.69 | 0.74 |
| 1424115_at | protein phosphatase 5, catalytic subunit | *Ppp5c* | 1.24 | 0.73 |
| 1441870_s_at | polycystic kidney disease 2 | *Pkd2* | 1.75 | 0.73 |
| 1439830_at | mitogen-activated protein kinase kinase kinase 5 | *Map3k5* | 1.26 | 0.73 |
| 1454722_at | phosphatase and tensin homolog | *Pten* | 1.35 | 0.73 |
| 1448680_at | serine (or cysteine) peptidase inhibitor, clade A, member 1C | *Serpina1c* | 1.30 | 0.73 |
| 1419523_at | cytochrome P450, family 3, subfamily a, polypeptide 13 | *Cyp3a13* | 2.92 | 0.73 |
| 1426349_s_at | thymopoietin | *Tmpo* | 1.43 | 0.73 |
| 1426721_s_at | TCDD-inducible poly(ADP-ribose) polymerase | *Tiparp* | 1.42 | 0.73 |
| 1423684_at | heterogeneous nuclear ribonucleoprotein K | *Hnrnpk* | 1.96 | 0.73 |
| 1451154_a_at | CUGBP, Elav-like family member 2 | *Celf2* | 1.41 | 0.73 |
| 1416214_at | minichromosome maintenance deficient 4 homolog (S. cerevisiae) | *Mcm4* | 2.10 | 0.73 |
| 1430519_a_at | CCR4-NOT transcription complex, subunit 7 | *Cnot7* | 1.39 | 0.73 |
| 1455362_at | angel homolog 2 (Drosophila) | *Angel2* | 2.56 | 0.73 |
| 1427314_at | transmembrane emp24 protein transport domain containing 7 | *Tmed7* | 1.87 | 0.73 |
| 1436596_at | H2A histone family, member V | *H2afv* | 1.81 | 0.73 |
| 1438669_at | DDB1 and CUL4 associated factor 12 | *Dcaf12* | 1.45 | 0.73 |
| 1428796_at | bobby sox homolog (Drosophila) | *Bbx* | 1.25 | 0.73 |
| 1434181_at | fermitin family homolog 2 (Drosophila) | *Fermt2* | 1.42 | 0.73 |
| 1442804_at | Gardner-Rasheed feline sarcoma viral (Fgr) oncogene homolog | *Fgr* | 1.30 | 0.73 |
| 1448502_at | solute carrier family 16 (monocarboxylic acid transporters), member 7 | *Slc16a7* | 1.81 | 0.73 |
| 1418486_at | vanin 1 | *Vnn1* | 1.25 | 0.73 |
| 1453030_at | male-specific lethal 2 homolog (Drosophila) | *Msl2* | 1.35 | 0.73 |
| 1419749_at | tRNA aspartic acid methyltransferase 1 | *Trdmt1* | 1.24 | 0.73 |
| 1435908_at | neurexin II | *Nrxn2* | 1.37 | 0.73 |
| 1434148_at | transcription factor 4 | *Tcf4* | 1.98 | 0.73 |
| 1452035_at | collagen, type IV, alpha 1 | *Col4a1* | 2.34 | 0.73 |
| 1416407_at | phosphoprotein enriched in astrocytes 15A | *Pea15a* | 1.43 | 0.73 |
| 1437843_s_at | nucleoporin like 1 | *Nupl1* | 2.72 | 0.73 |
| 1416551_at | ATPase, Ca++ transporting, cardiac muscle, slow twitch 2 | *Atp2a2* | 1.33 | 0.73 |
| 1436299_at | glutaminase | *Gls* | 1.80 | 0.73 |
| 1428103_at | a disintegrin and metallopeptidase domain 10 | *Adam10* | 2.03 | 0.73 |
| 1437226_x_at | MARCKS-like 1 | *Marcksl1* | 2.33 | 0.73 |
| 1451252_at | interferon regulatory factor 2 binding protein 1 | *Irf2bp1* | 2.01 | 0.73 |
| 1423641_s_at | CCR4-NOT transcription complex, subunit 7 | *Cnot7* | 1.33 | 0.73 |
| 1430802_at | neuregulin 1 | *Nrg1* | 1.41 | 0.73 |
| 1416166_a_at | peroxiredoxin 4 | *Prdx4* | 1.44 | 0.73 |
| 1421023_at | phosphatidylinositol 3-kinase, C2 domain containing, alpha polypeptide | *Pik3c2a* | 1.37 | 0.73 |
| 1434468_at | OTU domain containing 4 | *Otud4* | 1.61 | 0.73 |
| 1424998_at | EGF-like module containing, mucin-like, hormone receptor-like sequence 4 | *Emr4* | 1.53 | 0.73 |
| 1456699_s_at | YTH domain containing 1 | *Ythdc1* | 1.65 | 0.73 |
| 1435753_a_at | nuclear casein kinase and cyclin-dependent kinase substrate 1 | *Nucks1* | 1.47 | 0.73 |
| 1415767_at | YTH domain family 1 | *Ythdf1* | 1.34 | 0.73 |
| 1420872_at | guanylate cyclase 1, soluble, beta 3 | *Gucy1b3* | 1.74 | 0.73 |
| 1425241_a_at | WD repeat and SOCS box-containing 1 | *Wsb1* | 2.42 | 0.73 |
| 1447850_x_at | zinc finger, AN1-type domain 3 | *Zfand3* | 1.75 | 0.73 |
| 1415729_at | 3-phosphoinositide dependent protein kinase 1 | *Pdpk1* | 1.25 | 0.73 |
| 1422614_s_at | biogenesis of lysosome-related organelles complex-1, subunit 1 | *Bloc1s1* | 1.91 | 0.73 |
| 1460335_at | LysM, putative peptidoglycan-binding, domain containing 3 | *Lysmd3* | 1.91 | 0.73 |
| 1426645_at | heat shock protein 90, alpha (cytosolic), class A member 1 | *Hsp90aa1* | 1.39 | 0.73 |
| 1435440_at | PDZ domain containing 8 | *Pdzd8* | 1.40 | 0.73 |
| 1428407_at | heterogeneous nuclear ribonucleoprotein A0 | *Hnrnpa0* | 1.43 | 0.73 |
| 1450629_at | LIM domain and actin binding 1 | *Lima1* | 1.35 | 0.73 |
| 1433444_at | 3-hydroxy-3-methylglutaryl-Coenzyme A synthase 1 | *Hmgcs1* | 2.04 | 0.73 |
| 1428270_at | glycosyltransferase 8 domain containing 1 | *Glt8d1* | 1.68 | 0.73 |
| 1453118_s_at | ribosomal protein L22 | *Rpl22* | 1.46 | 0.73 |
| 1423613_at | sperm specific antigen 2 | *Ssfa2* | 1.49 | 0.73 |
| 1426798_a_at | protein phosphatase 1, regulatory (inhibitor) subunit 15b | *Ppp1r15b* | 1.24 | 0.73 |
| 1415764_at | zinc finger CCCH type containing 11A | *Zc3h11a* | 1.43 | 0.73 |
| 1437513_a_at | serine incorporator 1 | *Serinc1* | 1.62 | 0.73 |
| 1448061_at | macrophage scavenger receptor 1 | *Msr1* | 2.78 | 0.73 |
| 1435477_s_at | Fc receptor, IgG, low affinity IIb | *Fcgr2b* | 2.08 | 0.73 |
| 1428830_at | ataxia telangiectasia mutated homolog (human) | *Atm* | 1.40 | 0.73 |
| 1457707_at | multiple C2 domains, transmembrane 2 | *Mctp2* | 1.69 | 0.73 |
| 1416094_at | a disintegrin and metallopeptidase domain 9 (meltrin gamma) | *Adam9* | 1.25 | 0.73 |
| 1426052_at | mutL homolog 3 (E coli) | *Mlh3* | 1.33 | 0.73 |
| 1437334_x_at | poly(A)-specific ribonuclease (deadenylation nuclease) | *Parn* | 1.54 | 0.73 |
| 1422717_at | acid phosphatase 1, soluble | *Acp1* | 1.26 | 0.73 |
| 1437108_at | LSM6 homolog, U6 small nuclear RNA associated (S. cerevisiae) | *Lsm6* | 1.66 | 0.73 |
| 1425731_at | ankyrin repeat domain 24 | *Ankrd24* | 1.64 | 0.73 |
| 1419089_at | tissue inhibitor of metalloproteinase 3 | *Timp3* | 1.23 | 0.73 |
| 1419258_at | transcription elongation factor A (SII) 1 | *Tcea1* | 1.22 | 0.73 |
| 1439847_s_at | Kruppel-like factor 12 | *Klf12* | 1.43 | 0.73 |
| 1426407_at | CUGBP, Elav-like family member 1 | *Celf1* | 1.32 | 0.73 |
| 1440279_at | thioredoxin-related transmembrane protein 3 | *Tmx3* | 1.34 | 0.73 |
| 1425149_a_at | phosducin-like | *Pdcl* | 1.40 | 0.73 |
| 1428817_at | MON2 homolog (yeast) | *Mon2* | 1.43 | 0.73 |
| 1429947_a_at | Z-DNA binding protein 1 | *Zbp1* | 3.52 | 0.73 |
| 1419693_at | collectin sub-family member 12 | *Colec12* | 1.36 | 0.73 |
| 1438365_x_at | lysosomal-associated protein transmembrane 4B | *Laptm4b* | 1.33 | 0.73 |
| 1434674_at | lysosomal trafficking regulator | *Lyst* | 1.32 | 0.73 |
| 1439511_at | cyclin-dependent kinase 7 | *Cdk7* | 1.23 | 0.72 |
| 1428259_at | peroxidasin homolog (Drosophila) | *Pxdn* | 1.29 | 0.72 |
| 1434791_at | ATPase, H+ transporting, lysosomal V0 subunit A2 | *Atp6v0a2* | 1.76 | 0.72 |
| 1456615_a_at | bromodomain PHD finger transcription factor | *Bptf* | 1.27 | 0.72 |
| 1419971_s_at | solute carrier family 35, member A5 | *Slc35a5* | 1.26 | 0.72 |
| 1452238_at | ArfGAP with FG repeats 1 | *Agfg1* | 1.55 | 0.72 |
| 1435653_at | abhydrolase domain containing 2 | *Abhd2* | 2.17 | 0.72 |
| 1456790_at | zinc finger protein 800 | *Zfp800* | 1.89 | 0.72 |
| 1452534_a_at | high mobility group box 2 | *Hmgb2* | 1.99 | 0.72 |
| 1423962_at | WD repeat domain 26 | *Wdr26* | 1.35 | 0.72 |
| 1428173_at | echinoderm microtubule associated protein like 2 | *Eml2* | 1.23 | 0.72 |
| 1456028_x_at | myristoylated alanine rich protein kinase C substrate | *Marcks* | 1.75 | 0.72 |
| 1433442_at | kelch-like 9 (Drosophila) | *Klhl9* | 1.57 | 0.72 |
| 1428412_at | transmembrane 9 superfamily member 3 | *Tm9sf3* | 1.31 | 0.72 |
| 1450779_at | fatty acid binding protein 7, brain | *Fabp7* | 3.71 | 0.72 |
| 1448162_at | vascular cell adhesion molecule 1 | *Vcam1* | 2.86 | 0.72 |
| 1451644_a_at | DNA-damage regulated autophagy modulator 1 | *Dram1* | 1.46 | 0.72 |
| 1421182_at | C-type lectin domain family 1, member b | *Clec1b* | 2.30 | 0.72 |
| 1453416_at | growth arrest-specific 2 like 3 | *Gas2l3* | 1.52 | 0.72 |
| 1435972_at | calpastatin | *Cast* | 1.32 | 0.72 |
| 1421828_at | karyopherin (importin) alpha 3 | *Kpna3* | 1.22 | 0.72 |
| 1453587_at | gamma-glutamyltransferase 6 | *Ggt6* | 1.37 | 0.72 |
| 1429432_at | HLA-B associated transcript 2-like 2 | *Bat2l2* | 1.21 | 0.72 |
| 1455822_x_at | surfeit gene 4 | *Surf4* | 1.64 | 0.72 |
| 1422659_at | calcium/calmodulin-dependent protein kinase II, delta | *Camk2d* | 1.75 | 0.72 |
| 1428277_at | OTU domain containing 6B | *Otud6b* | 1.30 | 0.72 |
| 1424211_at | solute carrier family 25, member 33 | *Slc25a33* | 1.45 | 0.72 |
| 1455462_at | adenylate cyclase 2 | *Adcy2* | 1.37 | 0.72 |
| 1424402_at | RUN and FYVE domain containing 3 | *Rufy3* | 1.53 | 0.72 |
| 1417770_s_at | proteasome (prosome, macropain) 26S subunit, ATPase, 6 | *Psmc6* | 1.24 | 0.72 |
| 1428668_at | acyl-Coenzyme A binding domain containing 3 | *Acbd3* | 1.30 | 0.72 |
| 1448894_at | aldo-keto reductase family 1, member B8 | *Akr1b8* | 1.45 | 0.72 |
| 1428251_at | SMC hinge domain containing 1 | *Smchd1* | 1.93 | 0.72 |
| 1436954_at | WAS/WASL interacting protein family, member 1 | *Wipf1* | 1.63 | 0.72 |
| 1454989_at | centrosome and spindle pole associated protein 1 | *Cspp1* | 1.30 | 0.72 |
| 1434833_at | mitogen-activated protein kinase kinase kinase kinase 2 | *Map4k2* | 1.54 | 0.72 |
| 1438473_at | ADP-ribosylation factor-like 15 | *Arl15* | 1.51 | 0.72 |
| 1429004_at | pleckstrin homology domain interacting protein | *Phip* | 2.26 | 0.72 |
| 1448617_at | CD53 antigen | *Cd53* | 2.54 | 0.72 |
| 1437735_at | protein phosphatase 1, regulatory (inhibitor) subunit 12A | *Ppp1r12a* | 1.66 | 0.72 |
| 1449616_s_at | golgi autoantigen, golgin subfamily a, 3 | *Golga3* | 1.86 | 0.72 |
| 1452852_at | TWIST neighbor | *Twistnb* | 1.65 | 0.72 |
| 1439088_at | PDZ domain containing 8 | *Pdzd8* | 1.60 | 0.72 |
| 1428389_s_at | WD repeat domain 43 | *Wdr43* | 1.52 | 0.72 |
| 1455738_at | coiled-coil domain containing 55 | *Ccdc55* | 1.94 | 0.72 |
| 1444437_at | ubiquitin specific peptidase 34 | *Usp34* | 2.25 | 0.72 |
| 1433848_at | cell division cycle 27 homolog (S. cerevisiae) | *Cdc27* | 1.28 | 0.72 |
| 1423675_at | ubiquitin specific peptidase 1 | *Usp1* | 1.97 | 0.72 |
| 1426425_at | SGT1, suppressor of G2 allele of SKP1 (S. cerevisiae) | *Sugt1* | 1.31 | 0.72 |
| 1424206_at | SWI/SNF related, matrix associated, actin dependent regulator of chromatin, subfamily a, member 5 | *Smarca5* | 1.29 | 0.72 |
| 1434339_at | formin binding protein 1-like | *Fnbp1l* | 1.72 | 0.72 |
| 1433747_at | leucyl/cystinyl aminopeptidase | *Lnpep* | 1.24 | 0.72 |
| 1419585_at | retinitis pigmentosa 2 homolog (human) | *Rp2h* | 2.42 | 0.72 |
| 1420249_s_at | chemokine (C-C motif) ligand 6 | *Ccl6* | 6.12 | 0.72 |
| 1437119_at | endoplasmic reticulum (ER) to nucleus signalling 1 | *Ern1* | 1.37 | 0.72 |
| 1415790_at | ubiquitin-like domain containing CTD phosphatase 1 | *Ublcp1* | 1.26 | 0.72 |
| 1455256_at | TRAF2 and NCK interacting kinase | *Tnik* | 1.59 | 0.72 |
| 1433868_at | BTB (POZ) domain containing 3 | *Btbd3* | 1.67 | 0.72 |
| 1434272_at | cytoplasmic polyadenylation element binding protein 2 | *Cpeb2* | 1.39 | 0.72 |
| 1427468_at | protein phosphatase 3, catalytic subunit, beta isoform | *Ppp3cb* | 1.79 | 0.72 |
| 1448419_at | processing of precursor 4, ribonuclease P/MRP family, (S. cerevisiae) | *Pop4* | 1.25 | 0.72 |
| 1431808_a_at | inter alpha-trypsin inhibitor, heavy chain 4 | *Itih4* | 3.26 | 0.72 |
| 1428269_a_at | glycosyltransferase 8 domain containing 1 | *Glt8d1* | 1.48 | 0.71 |
| 1433770_at | dihydropyrimidinase-like 2 | *Dpysl2* | 1.81 | 0.71 |
| 1424706_at | zinc finger protein 51 | *Zfp51* | 2.04 | 0.71 |
| 1443816_s_at | phosphoinositide-3-kinase, regulatory subunit 6 | *Pik3r6* | 1.26 | 0.71 |
| 1416950_at | tumor necrosis factor, alpha-induced protein 8 | *Tnfaip8* | 3.69 | 0.71 |
| 1443059_at | hydroxysteroid (17-beta) dehydrogenase 11 | *Hsd17b11* | 1.54 | 0.71 |
| 1450642_at | SECIS binding protein 2-like | *Secisbp2l* | 1.28 | 0.71 |
| 1425481_at | CCR4-NOT transcription complex, subunit 6-like | *Cnot6l* | 1.40 | 0.71 |
| 1428968_at | centrosomal protein 57 | *Cep57* | 1.36 | 0.71 |
| 1438386_x_at | methionine adenosyltransferase II, alpha | *Mat2a* | 1.76 | 0.71 |
| 1437200_at | FCH domain only 2 | *Fcho2* | 1.97 | 0.71 |
| 1428755_at | cAMP responsive element binding protein 1 | *Creb1* | 1.91 | 0.71 |
| 1427090_at | zinc finger, BED domain containing 4 | *Zbed4* | 1.58 | 0.71 |
| 1456433_at | regulator of chromosome condensation (RCC1) and BTB (POZ) domain containing protein 1 | *Rcbtb1* | 1.35 | 0.71 |
| 1456700_x_at | myristoylated alanine rich protein kinase C substrate | *Marcks* | 2.89 | 0.71 |
| 1444952_a_at | nuclear casein kinase and cyclin-dependent kinase substrate 1 | *Nucks1* | 1.31 | 0.71 |
| 1425610_s_at | UDP-N-acetyl-alpha-D-galactosamine:polypeptide N-acetylgalactosaminyltransferase 2 | *Galnt2* | 1.32 | 0.71 |
| 1447824_x_at | heat shock protein 5 | *Hspa5* | 1.24 | 0.71 |
| 1429437_at | PRP40 pre-mRNA processing factor 40 homolog A (yeast) | *Prpf40a* | 1.56 | 0.71 |
| 1421058_at | alcohol dehydrogenase 7 (class IV), mu or sigma polypeptide | *Adh7* | 1.93 | 0.71 |
| 1420175_at | Tax1 (human T-cell leukemia virus type I) binding protein 1 | *Tax1bp1* | 1.27 | 0.71 |
| 1422062_at | macrophage scavenger receptor 1 | *Msr1* | 2.61 | 0.71 |
| 1428733_at | guanine nucleotide binding protein (G protein), gamma transducing activity polypeptide 2 | *Gngt2* | 1.54 | 0.71 |
| 1452294_at | protocadherin 1 | *Pcdh1* | 1.24 | 0.71 |
| 1457674_at | vacuolar protein sorting 13A (yeast) | *Vps13a* | 1.57 | 0.71 |
| 1418227_at | origin recognition complex, subunit 2 | *Orc2* | 1.46 | 0.71 |
| 1415766_at | SEC22 vesicle trafficking protein homolog B (S. cerevisiae) | *Sec22b* | 1.32 | 0.71 |
| 1451384_at | jumonji domain containing 5 | *Jmjd5* | 1.48 | 0.71 |
| 1454608_x_at | transthyretin | *Ttr* | 1.46 | 0.71 |
| 1436034_at | centrosomal protein 68 | *Cep68* | 1.35 | 0.71 |
| 1430154_at | vacuolar protein sorting 13A (yeast) | *Vps13a* | 1.32 | 0.71 |
| 1455441_at | mitogen-activated protein kinase kinase kinase 7 | *Map3k7* | 1.21 | 0.71 |
| 1454838_s_at | protein kinase domain containing, cytoplasmic | *Pkdcc* | 2.57 | 0.71 |
| 1436899_at | zinc finger with UFM1-specific peptidase domain | *Zufsp* | 1.35 | 0.71 |
| 1452192_at | nuclear assembly factor 1 homolog (S. cerevisiae) | *Naf1* | 1.94 | 0.71 |
| 1431564_at | breast cancer anti-estrogen resistance 3 | *Bcar3* | 1.49 | 0.71 |
| 1456156_at | leptin receptor | *Lepr* | 1.76 | 0.71 |
| 1454920_at | ubiquitin-like, containing PHD and RING finger domains 2 | *Uhrf2* | 1.35 | 0.71 |
| 1447931_at | Wolf-Hirschhorn syndrome candidate 1-like 1 (human) | *Whsc1l1* | 1.55 | 0.71 |
| 1445888_x_at | poly (ADP-ribose) polymerase family, member 3 | *Parp3* | 1.50 | 0.71 |
| 1429427_s_at | transcription factor 7-like 2, T-cell specific, HMG-box | *Tcf7l2* | 1.21 | 0.71 |
| 1435923_at | 2-aminoethanethiol (cysteamine) dioxygenase | *Ado* | 1.67 | 0.71 |
| 1454711_at | triple functional domain (PTPRF interacting) | *Trio* | 2.31 | 0.71 |
| 1435079_at | serine/arginine-rich splicing factor 18 | *Sfrs18* | 1.34 | 0.71 |
| 1453406_a_at | RAB28, member RAS oncogene family | *Rab28* | 1.61 | 0.71 |
| 1417749_a_at | tight junction protein 1 | *Tjp1* | 1.43 | 0.71 |
| 1434384_at | nuclear receptor interacting protein 1 | *Nrip1* | 1.32 | 0.71 |
| 1418180_at | trans-acting transcription factor 1 | *Sp1* | 1.47 | 0.71 |
| 1417444_at | E2F transcription factor 5 | *E2f5* | 1.37 | 0.71 |
| 1417820_at | torsin family 1, member B | *Tor1b* | 1.20 | 0.71 |
| 1450868_at | heparan-alpha-glucosaminide N-acetyltransferase | *Hgsnat* | 1.29 | 0.71 |
| 1441975_at | acid phosphatase, prostate | *Acpp* | 1.39 | 0.71 |
| 1433512_at | Friend leukemia integration 1 | *Fli1* | 2.09 | 0.71 |
| 1440282_at | tubby like protein 4 | *Tulp4* | 1.33 | 0.71 |
| 1456293_s_at | cyclin H | *Ccnh* | 1.24 | 0.71 |
| 1416925_at | karyopherin (importin) beta 1 | *Kpnb1* | 1.47 | 0.71 |
| 1434967_at | zinc finger, SWIM domain containing 6 | *Zswim6* | 1.97 | 0.71 |
| 1427151_at | glutamine and serine rich 1 | *Qser1* | 1.72 | 0.71 |
| 1434386_at | ATPase, Ca++-sequestering | *Atp2c1* | 1.84 | 0.71 |
| 1422903_at | lymphocyte antigen 86 | *Ly86* | 2.25 | 0.71 |
| 1418204_s_at | allograft inflammatory factor 1 | *Aif1* | 1.96 | 0.71 |
| 1449044_at | eukaryotic translation elongation factor 1 epsilon 1 | *Eef1e1* | 1.30 | 0.71 |
| 1450994_at | Rho-associated coiled-coil containing protein kinase 1 | *Rock1* | 1.73 | 0.71 |
| 1417763_at | signal sequence receptor, alpha | *Ssr1* | 1.59 | 0.71 |
| 1435543_at | adenomatosis polyposis coli | *Apc* | 1.40 | 0.71 |
| 1435483_x_at | solute carrier family 25, member 32 | *Slc25a32* | 1.44 | 0.71 |
| 1447985_s_at | ankyrin repeat and IBR domain containing 1 | *Ankib1* | 1.73 | 0.71 |
| 1422715_s_at | acid phosphatase 1, soluble | *Acp1* | 1.34 | 0.71 |
| 1434659_at | AVL9 homolog (S. cerevisiase) | *Avl9* | 1.32 | 0.71 |
| 1456058_at | RNA binding motif protein 27 | *Rbm27* | 1.67 | 0.71 |
| 1448109_a_at | ribosomal protein L26 | *Rpl26* | 1.28 | 0.71 |
| 1425045_at | jumonji domain containing 7 | *Jmjd7* | 1.51 | 0.71 |
| 1428998_at | PHD finger protein 3 | *Phf3* | 1.75 | 0.71 |
| 1419059_at | serum amyloid P-component | *Apcs* | 6.82 | 0.71 |
| 1460231_at | interferon regulatory factor 5 | *Irf5* | 1.26 | 0.70 |
| 1426722_at | solute carrier family 38, member 2 | *Slc38a2* | 1.77 | 0.70 |
| 1426622_a_at | glutaminyl-peptide cyclotransferase (glutaminyl cyclase) | *Qpct* | 1.56 | 0.70 |
| 1426491_at | hect (homologous to the E6-AP (UBE3A) carboxyl terminus) domain and RCC1 (CHC1)-like domain (RLD) 2 | *Herc2* | 1.49 | 0.70 |
| 1449372_at | DnaJ (Hsp40) homolog, subfamily C, member 3 | *Dnajc3* | 1.37 | 0.70 |
| 1448270_at | DEAD (Asp-Glu-Ala-Asp) box polypeptide 21 | *Ddx21* | 1.80 | 0.70 |
| 1440285_at | protein phosphatase 1, regulatory (inhibitor) subunit 9A | *Ppp1r9a* | 1.45 | 0.70 |
| 1419397_at | polymerase (DNA directed), alpha 1 | *Pola1* | 2.37 | 0.70 |
| 1452713_a_at | small nuclear ribonucleoprotein 40 (U5) | *Snrnp40* | 1.32 | 0.70 |
| 1420940_x_at | regulator of G-protein signaling 5 | *Rgs5* | 1.73 | 0.70 |
| 1431089_at | cleavage and polyadenylation specific factor 2 | *Cpsf2* | 1.22 | 0.70 |
| 1442135_at | 2-aminoethanethiol (cysteamine) dioxygenase | *Ado* | 1.55 | 0.70 |
| 1427971_at | cell division cycle 73, Paf1/RNA polymerase II complex component, homolog (S. cerevisiae) | *Cdc73* | 2.09 | 0.70 |
| 1420916_at | PRP40 pre-mRNA processing factor 40 homolog A (yeast) | *Prpf40a* | 2.19 | 0.70 |
| 1433445_x_at | 3-hydroxy-3-methylglutaryl-Coenzyme A synthase 1 | *Hmgcs1* | 1.93 | 0.70 |
| 1429728_at | gypsy retrotransposon integrase 1 | *Gin1* | 1.32 | 0.70 |
| 1428854_at | transmembrane emp24 domain containing 8 | *Tmed8* | 1.89 | 0.70 |
| 1418771_a_at | carboxypeptidase B2 (plasma) | *Cpb2* | 2.00 | 0.70 |
| 1434884_at | metadherin | *Mtdh* | 1.36 | 0.70 |
| 1428582_at | methyltransferase like 10 | *Mettl10* | 2.12 | 0.70 |
| 1448737_at | tetraspanin 7 | *Tspan7* | 1.35 | 0.70 |
| 1429108_at | male-specific lethal 2 homolog (Drosophila) | *Msl2* | 1.61 | 0.70 |
| 1426501_a_at | TRAF-interacting protein with forkhead-associated domain | *Tifa* | 2.96 | 0.70 |
| 1434277_a_at | yippee-like 2 (Drosophila) | *Ypel2* | 1.74 | 0.70 |
| 1439465_x_at | ATP/GTP binding protein-like 5 | *Agbl5* | 1.34 | 0.70 |
| 1448242_at | Sec61 alpha 1 subunit (S. cerevisiae) | *Sec61a1* | 1.83 | 0.70 |
| 1451402_at | ecdysoneless homolog (Drosophila) | *Ecd* | 1.23 | 0.70 |
| 1434060_at | hect (homologous to the E6-AP (UBE3A) carboxyl terminus) domain and RCC1 (CHC1)-like domain (RLD) 1 | *Herc1* | 1.52 | 0.70 |
| 1456489_at | cleavage and polyadenylation factor subunit homolog (S. cerevisiae) | *Pcf11* | 1.28 | 0.70 |
| 1423042_at | DEAD/H (Asp-Glu-Ala-Asp/His) box polypeptide 3, X-linked | *Ddx3x* | 2.12 | 0.70 |
| 1422247_a_at | ubiquitously transcribed tetratricopeptide repeat gene, Y chromosome | *Uty* | 1.32 | 0.70 |
| 1452401_at | Wilms' tumour 1-associating protein | *Wtap* | 1.49 | 0.70 |
| 1452913_at | Purkinje cell protein 4-like 1 | *Pcp4l1* | 1.22 | 0.70 |
| 1437632_at | mediator complex subunit 14 | *Med14* | 2.01 | 0.70 |
| 1435028_at | WD repeat domain 7 | *Wdr7* | 1.60 | 0.70 |
| 1455156_at | striatin, calmodulin binding protein | *Strn* | 1.40 | 0.70 |
| 1438744_at | ankyrin repeat and SOCS box-containing 7 | *Asb7* | 1.27 | 0.70 |
| 1419506_at | geranylgeranyl diphosphate synthase 1 | *Ggps1* | 1.39 | 0.70 |
| 1428119_a_at | RAP2C, member of RAS oncogene family | *Rap2c* | 1.33 | 0.70 |
| 1455817_x_at | zinc finger, X-linked, duplicated B | *Zxdb* | 1.40 | 0.70 |
| 1450701_a_at | general transcription factor II H, polypeptide 2 | *Gtf2h2* | 1.49 | 0.70 |
| 1417931_at | N-deacetylase/N-sulfotransferase (heparan glucosaminyl) 2 | *Ndst2* | 1.41 | 0.70 |
| 1453055_at | sema domain, transmembrane domain (TM), and cytoplasmic domain, (semaphorin) 6D | *Sema6d* | 1.51 | 0.70 |
| 1418847_at | arginase type II | *Arg2* | 2.57 | 0.70 |
| 1423157_at | glucosamine-phosphate N-acetyltransferase 1 | *Gnpnat1* | 1.40 | 0.70 |
| 1433803_at | Janus kinase 1 | *Jak1* | 1.51 | 0.70 |
| 1450897_at | Rho GTPase activating protein 5 | *Arhgap5* | 1.42 | 0.70 |
| 1448545_at | syndecan 2 | *Sdc2* | 1.51 | 0.70 |
| 1436041_at | heart and neural crest derivatives expressed transcript 2 | *Hand2* | 1.75 | 0.70 |
| 1455105_at | protein tyrosine phosphatase, non-receptor type 12 | *Ptpn12* | 1.99 | 0.70 |
| 1434180_at | fermitin family homolog 2 (Drosophila) | *Fermt2* | 1.48 | 0.70 |
| 1428890_at | fem-1 homolog c (C.elegans) | *Fem1c* | 1.51 | 0.70 |
| 1426915_at | death associated protein kinase 1 | *Dapk1* | 1.28 | 0.70 |
| 1425494_s_at | bone morphogenetic protein receptor, type 1A | *Bmpr1a* | 2.40 | 0.70 |
| 1434592_at | solute carrier family 16 (monocarboxylic acid transporters), member 10 | *Slc16a10* | 1.37 | 0.70 |
| 1443779_s_at | ligand dependent nuclear receptor corepressor | *Lcor* | 1.71 | 0.70 |
| 1416659_at | eukaryotic translation initiation factor 3, subunit A | *Eif3a* | 1.82 | 0.70 |
| 1418655_at | interleukin 18 binding protein | *Il18bp* | 1.48 | 0.70 |
| 1425225_at | glutathione reductase | *Gsr* | 2.50 | 0.70 |
| 1423039_a_at | basic leucine zipper and W2 domains 1 | *Bzw1* | 1.57 | 0.70 |
| 1435768_at | AT rich interactive domain 4B (RBP1-like) | *Arid4b* | 2.01 | 0.70 |
| 1428388_at | tankyrase, TRF1-interacting ankyrin-related ADP-ribose polymerase 2 | *Tnks2* | 1.25 | 0.70 |
| 1454890_at | angiomotin | *Amot* | 1.99 | 0.70 |
| 1455913_x_at | transthyretin | *Ttr* | 1.55 | 0.70 |
| 1426485_at | UBX domain protein 4 | *Ubxn4* | 1.28 | 0.70 |
| 1450430_at | mannose receptor, C type 1 | *Mrc1* | 2.62 | 0.70 |
| 1426787_at | Sfi1 homolog, spindle assembly associated (yeast) | *Sfi1* | 1.38 | 0.70 |
| 1433754_at | muscleblind-like 2 | *Mbnl2* | 2.11 | 0.70 |
| 1425261_at | CCAAT/enhancer binding protein (C/EBP), gamma | *Cebpg* | 1.87 | 0.70 |
| 1417008_at | carnitine acetyltransferase | *Crat* | 1.50 | 0.70 |
| 1424981_at | neurolysin (metallopeptidase M3 family) | *Nln* | 2.12 | 0.70 |
| 1434112_at | latrophilin 2 | *Lphn2* | 1.38 | 0.69 |
| 1416201_at | v-crk sarcoma virus CT10 oncogene homolog (avian) | *Crk* | 1.48 | 0.69 |
| 1424842_a_at | Rho GTPase activating protein 24 | *Arhgap24* | 1.23 | 0.69 |
| 1431802_a_at | ethanolaminephosphotransferase 1 (CDP-ethanolamine-specific) | *Ept1* | 1.35 | 0.69 |
| 1434383_at | praja 2, RING-H2 motif containing | *Pja2* | 1.44 | 0.69 |
| 1426271_at | structural maintenance of chromosomes 5 | *Smc5* | 1.48 | 0.69 |
| 1453139_at | nudix (nucleoside diphosphate linked moiety X)-type motif 12 | *Nudt12* | 1.24 | 0.69 |
| 1433679_at | far upstream element (FUSE) binding protein 3 | *Fubp3* | 1.72 | 0.69 |
| 1436183_at | zinc finger CCCH type, antiviral 1 | *Zc3hav1* | 1.31 | 0.69 |
| 1431064_at | dipeptidylpeptidase 8 | *Dpp8* | 1.31 | 0.69 |
| 1422491_a_at | BCL2/adenovirus E1B interacting protein 2 | *Bnip2* | 1.79 | 0.69 |
| 1426419_at | RNA binding motif protein 26 | *Rbm26* | 1.58 | 0.69 |
| 1455336_at | THAP domain containing, apoptosis associated protein 2 | *Thap2* | 1.39 | 0.69 |
| 1439837_at | GRB10 interacting GYF protein 2 | *Gigyf2* | 2.22 | 0.69 |
| 1418990_at | membrane-spanning 4-domains, subfamily A, member 4D | *Ms4a4d* | 1.40 | 0.69 |
| 1418925_at | cadherin, EGF LAG seven-pass G-type receptor 1 (flamingo homolog, Drosophila) | *Celsr1* | 1.57 | 0.69 |
| 1451005_at | SMT3 suppressor of mif two 3 homolog 1 (yeast) | *Sumo1* | 1.28 | 0.69 |
| 1418826_at | membrane-spanning 4-domains, subfamily A, member 6B | *Ms4a6b* | 3.34 | 0.69 |
| 1417848_at | zinc finger protein 704 | *Zfp704* | 1.79 | 0.69 |
| 1429435_x_at | phosphatidylinositol 3-kinase, catalytic, alpha polypeptide | *Pik3ca* | 1.21 | 0.69 |
| 1416635_at | sphingomyelin phosphodiesterase, acid-like 3A | *Smpdl3a* | 2.13 | 0.69 |
| 1435325_at | ubiquitin specific peptidase 46 | *Usp46* | 1.38 | 0.69 |
| 1453160_at | mediator complex subunit 13 | *Med13* | 1.45 | 0.69 |
| 1428227_at | RE1-silencing transcription factor | *Rest* | 1.65 | 0.69 |
| 1427164_at | interleukin 13 receptor, alpha 1 | *Il13ra1* | 3.43 | 0.69 |
| 1426628_at | transmembrane protein 184C | *Tmem184c* | 2.05 | 0.69 |
| 1451054_at | orosomucoid 1 | *Orm1* | 2.94 | 0.69 |
| 1420387_at | MpV17 mitochondrial inner membrane protein | *Mpv17* | 1.61 | 0.69 |
| 1428674_at | PRP38 pre-mRNA processing factor 38 (yeast) domain containing B | *Prpf38b* | 1.44 | 0.69 |
| 1428945_at | ubiquitin-like modifier activating enzyme 6 | *Uba6* | 1.27 | 0.69 |
| 1428087_at | dynamin 1-like | *Dnm1l* | 1.43 | 0.69 |
| 1448888_at | protein phosphatase 1, regulatory (inhibitor) subunit 7 | *Ppp1r7* | 1.61 | 0.69 |
| 1433999_at | STE20-like kinase (yeast) | *Slk* | 1.69 | 0.69 |
| 1420621_a_at | amyloid beta (A4) precursor protein | *App* | 1.56 | 0.69 |
| 1457700_at | cytochrome P450, family 4, subfamily f, polypeptide 39 | *Cyp4f39* | 1.29 | 0.69 |
| 1451524_at | F-box and WD-40 domain protein 2 | *Fbxw2* | 1.49 | 0.69 |
| 1419042_at | interferon inducible GTPase 1 | *Iigp1* | 1.48 | 0.69 |
| 1436999_at | phosphotyrosine interaction domain containing 1 | *Pid1* | 1.43 | 0.69 |
| 1454616_at | ubiquitin protein ligase E3 component n-recognin 7 (putative) | *Ubr7* | 1.37 | 0.69 |
| 1435571_at | myelin protein zero-like 3 | *Mpzl3* | 1.53 | 0.69 |
| 1439401_x_at | protein phosphatase 2, regulatory subunit B (B56), epsilon isoform | *Ppp2r5e* | 1.41 | 0.69 |
| 1457566_at | zinc finger protein 677 | *Zfp677* | 1.38 | 0.69 |
| 1424033_at | serine/arginine-rich splicing factor 7 | *Srsf7* | 1.49 | 0.69 |
| 1436367_at | protein tyrosine phosphatase, receptor type, B | *Ptprb* | 1.43 | 0.69 |
| 1454717_at | ankyrin repeat domain 27 (VPS9 domain) | *Ankrd27* | 1.49 | 0.69 |
| 1455284_x_at | phosphatidylinositol glycan anchor biosynthesis, class X | *Pigx* | 1.45 | 0.69 |
| 1450788_at | serum amyloid A 1 | *Saa1* | 7.80 | 0.69 |
| 1436443_a_at | KDEL (Lys-Asp-Glu-Leu) containing 1 | *Kdelc1* | 1.72 | 0.69 |
| 1423431_a_at | MYB binding protein (P160) 1a | *Mybbp1a* | 1.32 | 0.69 |
| 1429359_s_at | RNA binding protein gene with multiple splicing | *Rbpms* | 1.38 | 0.69 |
| 1423141_at | lysosomal acid lipase A | *Lipa* | 1.34 | 0.69 |
| 1428976_at | thymopoietin | *Tmpo* | 2.57 | 0.69 |
| 1434267_at | NIMA (never in mitosis gene a)-related expressed kinase 1 | *Nek1* | 1.22 | 0.69 |
| 1423213_at | plexin C1 | *Plxnc1* | 1.27 | 0.69 |
| 1422519_at | calcium/calmodulin-dependent serine protein kinase (MAGUK family) | *Cask* | 1.54 | 0.69 |
| 1417026_at | prefoldin 1 | *Pfdn1* | 1.79 | 0.69 |
| 1448829_at | structural maintenance of chromosomes 6 | *Smc6* | 1.22 | 0.69 |
| 1455746_at | kinesin family member 13A | *Kif13a* | 1.53 | 0.69 |
| 1418660_at | circadian locomotor output cycles kaput | *Clock* | 1.61 | 0.69 |
| 1428625_a_at | RMI1, RecQ mediated genome instability 1, homolog (S. cerevisiae) | *Rmi1* | 1.25 | 0.69 |
| 1422533_at | cytochrome P450, family 51 | *Cyp51* | 2.59 | 0.69 |
| 1418960_at | PHD finger protein 20-like 1 | *Phf20l1* | 2.66 | 0.69 |
| 1423336_at | origin recognition complex, subunit 4 | *Orc4* | 1.51 | 0.69 |
| 1429056_at | N(alpha)-acetyltransferase 16, NatA auxiliary subunit | *Naa16* | 1.35 | 0.69 |
| 1453054_at | secretory carrier membrane protein 1 | *Scamp1* | 1.70 | 0.69 |
| 1415920_at | cleavage stimulation factor, 3' pre-RNA subunit 2, tau | *Cstf2t* | 1.24 | 0.69 |
| 1434961_at | ankyrin repeat and SOCS box-containing 1 | *Asb1* | 1.45 | 0.69 |
| 1422551_at | zinc finger with KRAB and SCAN domains 3 | *Zkscan3* | 1.24 | 0.69 |
| 1435181_at | lin-54 homolog (C. elegans) | *Lin54* | 1.85 | 0.68 |
| 1434188_at | solute carrier family 16 (monocarboxylic acid transporters), member 12 | *Slc16a12* | 2.15 | 0.68 |
| 1435947_at | Protein prenyltransferase alpha subunit repeat containing 1 | *Ptar1* | 1.97 | 0.68 |
| 1460729_at | Rho-associated coiled-coil containing protein kinase 1 | *Rock1* | 1.28 | 0.68 |
| 1417494_a_at | interleukin 1 receptor-like 1 | *Il1rl1* | 2.74 | 0.68 |
| 1452292_at | adaptor-related protein complex 2, beta 1 subunit | *Ap2b1* | 1.53 | 0.68 |
| 1454636_at | chromobox homolog 5 (Drosophila HP1a) | *Cbx5* | 1.37 | 0.68 |
| 1426861_at | aftiphilin | *Aftph* | 1.43 | 0.68 |
| 1429169_at | RNA binding motif protein 3 | *Rbm3* | 1.40 | 0.68 |
| 1416161_at | RAD21 homolog (S. pombe) | *Rad21* | 1.24 | 0.68 |
| 1426725_s_at | E26 avian leukemia oncogene 1, 5' domain | *Ets1* | 1.66 | 0.68 |
| 1437203_at | Casitas B-lineage lymphoma-like 1 | *Cbll1* | 1.73 | 0.68 |
| 1429652_at | proline-rich coiled-coil 1 | *Prrc1* | 2.08 | 0.68 |
| 1448316_at | CKLF-like MARVEL transmembrane domain containing 3 | *Cmtm3* | 2.14 | 0.68 |
| 1436817_at | exocyst complex component 5 | *Exoc5* | 1.81 | 0.68 |
| 1450665_at | GA repeat binding protein, alpha | *Gabpa* | 1.37 | 0.68 |
| 1455350_at | transmembrane protein 62 | *Tmem62* | 1.81 | 0.68 |
| 1436921_at | ATPase, Cu++ transporting, alpha polypeptide | *Atp7a* | 1.80 | 0.68 |
| 1454769_at | TatD DNase domain containing 2 | *Tatdn2* | 1.35 | 0.68 |
| 1426395_s_at | eukaryotic translation initiation factor 3, subunit J | *Eif3j* | 1.48 | 0.68 |
| 1451473_a_at | crystallin, zeta (quinone reductase)-like 1 | *Cryzl1* | 1.63 | 0.68 |
| 1423208_at | transmembrane protein 167 | *Tmem167* | 2.04 | 0.68 |
| 1441238_at | PDS5, regulator of cohesion maintenance, homolog A (S. cerevisiae) | *Pds5a* | 2.28 | 0.68 |
| 1452253_at | cysteine rich transmembrane BMP regulator 1 (chordin like) | *Crim1* | 1.95 | 0.68 |
| 1435291_at | LEM domain containing 3 | *Lemd3* | 1.74 | 0.68 |
| 1449262_s_at | lin-7 homolog C (C. elegans) | *Lin7c* | 1.34 | 0.68 |
| 1435234_at | nuclear receptor coactivator 2 | *Ncoa2* | 1.28 | 0.68 |
| 1452079_s_at | DCN1, defective in cullin neddylation 1, domain containing 1 (S. cerevisiae) | *Dcun1d1* | 1.56 | 0.68 |
| 1424191_a_at | transmembrane protein 41a | *Tmem41a* | 2.81 | 0.68 |
| 1426819_at | homeodomain interacting protein kinase 3 | *Hipk3* | 1.52 | 0.68 |
| 1426714_at | solute carrier family 46, member 1 | *Slc46a1* | 1.21 | 0.68 |
| 1424698_s_at | grancalcin | *Gca* | 1.91 | 0.68 |
| 1418129_at | 24-dehydrocholesterol reductase | *Dhcr24* | 1.90 | 0.68 |
| 1428657_at | ras responsive element binding protein 1 | *Rreb1* | 1.48 | 0.68 |
| 1434489_at | engulfment and cell motility 3, ced-12 homolog (C. elegans) | *Elmo3* | 1.22 | 0.68 |
| 1451775_s_at | interleukin 13 receptor, alpha 1 | *Il13ra1* | 2.70 | 0.68 |
| 1417399_at | growth arrest specific 6 | *Gas6* | 3.84 | 0.68 |
| 1451437_at | zinc finger, DHHC domain containing 20 | *Zdhhc20* | 1.41 | 0.68 |
| 1419493_a_at | tumor protein D52 | *Tpd52* | 2.29 | 0.68 |
| 1419668_at | sarcoglycan, beta (dystrophin-associated glycoprotein) | *Sgcb* | 1.35 | 0.68 |
| 1455506_at | solute carrier family 25, member 34 | *Slc25a34* | 1.52 | 0.68 |
| 1426914_at | MARVEL (membrane-associating) domain containing 2 | *Marveld2* | 1.96 | 0.68 |
| 1440831_at | BTB and CNC homology 1 | *Bach1* | 1.26 | 0.68 |
| 1437035_x_at | ring finger protein 14 | *Rnf14* | 1.37 | 0.68 |
| 1455819_at | ROD1 regulator of differentiation 1 (S. pombe) | *Rod1* | 1.55 | 0.68 |
| 1424632_a_at | REV3-like, catalytic subunit of DNA polymerase zeta RAD54 like (S. cerevisiae) | *Rev3l* | 1.49 | 0.68 |
| 1436373_at | mitogen-activated protein kinase kinase kinase 10 | *Map3k10* | 1.40 | 0.68 |
| 1450234_at | membrane-spanning 4-domains, subfamily A, member 6C | *Ms4a6c* | 3.31 | 0.68 |
| 1418565_at | serpine1 mRNA binding protein 1 | *Serbp1* | 1.99 | 0.68 |
| 1442359_at | six transmembrane epithelial antigen of prostate 2 | *Steap2* | 1.78 | 0.68 |
| 1456607_at | valosin containing protein (p97)/p47 complex interacting protein 1 | *Vcpip1* | 1.45 | 0.68 |
| 1455029_at | kinesin family member 21A | *Kif21a* | 1.68 | 0.68 |
| 1443832_s_at | serum deprivation response | *Sdpr* | 1.30 | 0.68 |
| 1416035_at | hypoxia inducible factor 1, alpha subunit | *Hif1a* | 1.84 | 0.68 |
| 1437032_x_at | RNA binding motif protein 14 | *Rbm14* | 1.77 | 0.68 |
| 1423961_at | WD repeat domain 26 | *Wdr26* | 1.36 | 0.68 |
| 1454243_at | intestinal cell kinase | *Ick* | 1.23 | 0.68 |
| 1449335_at | tissue inhibitor of metalloproteinase 3 | *Timp3* | 1.27 | 0.68 |
| 1425051_at | isochorismatase domain containing 1 | *Isoc1* | 1.20 | 0.68 |
| 1455209_at | Phosphoenolpyruvate carboxykinase 1, cytosolic | *Pck1* | 3.00 | 0.68 |
| 1417188_s_at | ubiquitin-conjugating enzyme E2K (UBC1 homolog, yeast) | *Ube2k* | 1.26 | 0.68 |
| 1447448_s_at | Kruppel-like factor 6 | *Klf6* | 2.78 | 0.68 |
| 1425972_a_at | zinc finger protein X-linked | *Zfx* | 1.20 | 0.68 |
| 1428274_s_at | abhydrolase domain containing 13 | *Abhd13* | 1.46 | 0.68 |
| 1437104_at | ADP-ribosylation factor guanine nucleotide-exchange factor 1(brefeldin A-inhibited) | *Arfgef1* | 1.50 | 0.68 |
| 1434106_at | EPM2A (laforin) interacting protein 1 | *Epm2aip1* | 2.39 | 0.68 |
| 1419562_at | baculoviral IAP repeat-containing 6 | *Birc6* | 1.51 | 0.68 |
| 1416811_s_at | cytotoxic T lymphocyte-associated protein 2 alpha | *Ctla2a* | 4.41 | 0.68 |
| 1428643_at | mannoside acetylglucosaminyltransferase 5 | *Mgat5* | 2.87 | 0.68 |
| 1420408_a_at | mesoderm specific transcript | *Mest* | 1.71 | 0.67 |
| 1437533_at | X-linked inhibitor of apoptosis | *Xiap* | 1.48 | 0.67 |
| 1434099_at | peroxisome proliferative activated receptor, gamma, coactivator 1 alpha | *Ppargc1a* | 1.81 | 0.67 |
| 1426587_a_at | signal transducer and activator of transcription 3 | *Stat3* | 2.05 | 0.67 |
| 1418151_at | myotubularin related protein 4 | *Mtmr4* | 1.24 | 0.67 |
| 1438349_at | zinc finger protein | *Zfp229* | 1.86 | 0.67 |
| 1428558_at | O-sialoglycoprotein endopeptidase-like 1 | *Osgepl1* | 1.30 | 0.67 |
| 1452166_a_at | keratin 10 | *Krt10* | 1.31 | 0.67 |
| 1434304_s_at | nuclear undecaprenyl pyrophosphate synthase 1 homolog (S. cerevisiae) | *Nus1* | 1.45 | 0.67 |
| 1424231_s_at | exocyst complex component 6 | *Exoc6* | 1.34 | 0.67 |
| 1450901_a_at | SMEK homolog 2, suppressor of mek1 (Dictyostelium) | *Smek2* | 1.24 | 0.67 |
| 1436597_at | ankyrin repeat and KH domain containing 1 | *Ankhd1* | 2.68 | 0.67 |
| 1438530_at | tissue factor pathway inhibitor | *Tfpi* | 1.50 | 0.67 |
| 1417250_at | ring finger protein, LIM domain interacting | *Rlim* | 1.54 | 0.67 |
| 1421879_at | myotubularin related protein 1 | *Mtmr1* | 1.34 | 0.67 |
| 1450647_at | Hermansky-Pudlak syndrome 3 homolog (human) | *Hps3* | 1.53 | 0.67 |
| 1437466_at | activated leukocyte cell adhesion molecule | *Alcam* | 1.62 | 0.67 |
| 1451628_a_at | ankyrin 3, epithelial | *Ank3* | 1.26 | 0.67 |
| 1426114_at | heterogeneous nuclear ribonucleoprotein A/B | *Hnrnpab* | 1.30 | 0.67 |
| 1419209_at | chemokine (C-X-C motif) ligand 5 | *Cxcl5* | 12.60 | 0.67 |
| 1437609_at | ubiquitin-conjugating enzyme E2U (putative) | *Ube2u* | 1.87 | 0.67 |
| 1435070_at | AE binding protein 2 | *Aebp2* | 1.24 | 0.67 |
| 1436027_at | oxysterol binding protein-like 11 | *Osbpl11* | 1.37 | 0.67 |
| 1444141_at | sorting nexin 13 | *Snx13* | 1.38 | 0.67 |
| 1455246_at | SWI/SNF related, matrix associated, actin dependent regulator of chromatin, subfamily c, member 1 | *Smarcc1* | 1.58 | 0.67 |
| 1435884_at | intersectin 1 (SH3 domain protein 1A) | *Itsn1* | 2.09 | 0.67 |
| 1417842_at | calcium modulating ligand | *Caml* | 1.34 | 0.67 |
| 1416808_at | nidogen 1 | *Nid1* | 2.22 | 0.67 |
| 1452384_at | ectonucleotide pyrophosphatase/phosphodiesterase 3 | *Enpp3* | 1.70 | 0.67 |
| 1447947_at | zinc finger, FYVE domain containing 16 | *Zfyve16* | 1.99 | 0.67 |
| 1456389_at | zinc finger E-box binding homeobox 2 | *Zeb2* | 1.49 | 0.67 |
| 1438832_x_at | DEAH (Asp-Glu-Ala-His) box polypeptide 30 | *Dhx30* | 2.08 | 0.67 |
| 1423760_at | CD44 antigen | *Cd44* | 2.42 | 0.67 |
| 1426451_at | spastic paraplegia 11 | *Spg11* | 1.48 | 0.67 |
| 1433966_x_at | asparagine synthetase | *Asns* | 4.09 | 0.67 |
| 1427144_at | heterogeneous nuclear ribonucleoprotein L-like | *Hnrpll* | 1.89 | 0.67 |
| 1456763_at | myosin phosphatase Rho interacting protein | *Mprip* | 1.40 | 0.67 |
| 1419372_at | golgi SNAP receptor complex member 2 | *Gosr2* | 1.25 | 0.67 |
| 1416802_a_at | cell division cycle associated 5 | *Cdca5* | 1.76 | 0.67 |
| 1428729_at | KRIT1, ankyrin repeat containing | *Krit1* | 1.45 | 0.67 |
| 1420116_s_at | golgi phosphoprotein 3 | *Golph3* | 1.38 | 0.67 |
| 1455520_at | protein phosphatase 2, regulatory subunit B (B56), gamma isoform | *Ppp2r5c* | 1.45 | 0.67 |
| 1428579_at | formin-like 2 | *Fmnl2* | 2.08 | 0.67 |
| 1452759_s_at | PTPRF interacting protein, binding protein 1 (liprin beta 1) | *Ppfibp1* | 1.44 | 0.67 |
| 1430805_s_at | RMI1, RecQ mediated genome instability 1, homolog (S. cerevisiae) | *Rmi1* | 1.40 | 0.67 |
| 1433778_at | tankyrase, TRF1-interacting ankyrin-related ADP-ribose polymerase | *Tnks* | 1.51 | 0.67 |
| 1434784_s_at | transmembrane protein 106C | *Tmem106c* | 1.24 | 0.67 |
| 1420021_s_at | suppressor of zeste 12 homolog (Drosophila) | *Suz12* | 1.42 | 0.67 |
| 1428982_at | ATPase family, AAA domain containing 2B | *Atad2b* | 1.35 | 0.67 |
| 1451003_at | TGF-beta activated kinase 1/MAP3K7 binding protein 2 | *Tab2* | 1.39 | 0.67 |
| 1456914_at | Solute carrier family 16 (monocarboxylic acid transporters), member 4 | *Slc16a4* | 2.68 | 0.67 |
| 1437812_x_at | alpha glucosidase 2 alpha neutral subunit | *Ganab* | 1.44 | 0.67 |
| 1428907_at | RNA binding motif protein 25 | *Rbm25* | 1.65 | 0.67 |
| 1452689_at | zinc finger protein 512 | *Zfp512* | 1.30 | 0.67 |
| 1435824_at | YY1 transcription factor | *Yy1* | 1.25 | 0.67 |
| 1452969_at | ATPase, Ca++ transporting, plasma membrane 1 | *Atp2b1* | 1.67 | 0.67 |
| 1429184_at | GTPase, very large interferon inducible 1 | *Gvin1* | 2.72 | 0.67 |
| 1455130_at | SPT2, Suppressor of Ty, domain containing 1 (S. cerevisiae) | *Spty2d1* | 1.80 | 0.67 |
| 1419922_s_at | attractin like 1 | *Atrnl1* | 1.31 | 0.67 |
| 1421868_a_at | pancreatic lipase | *Pnlip* | 3.44 | 0.67 |
| 1434298_at | zinc finger E-box binding homeobox 2 | *Zeb2* | 2.18 | 0.67 |
| 1438773_at | six transmembrane epithelial antigen of prostate 2 | *Steap2* | 1.38 | 0.67 |
| 1418981_at | caspase 12 | *Casp12* | 1.30 | 0.67 |
| 1435748_at | guanine deaminase | *Gda* | 2.45 | 0.67 |
| 1437658_a_at | small nucleolar RNA host gene (non-protein coding) 1 | *Snhg1* | 1.46 | 0.67 |
| 1449626_s_at | acyl-Coenzyme A binding domain containing 4 | *Acbd4* | 1.27 | 0.67 |
| 1428192_at | kelch repeat and BTB (POZ) domain containing 7 | *Kbtbd7* | 1.45 | 0.67 |
| 1453221_at | golgi associated PDZ and coiled-coil motif containing | *Gopc* | 1.52 | 0.67 |
| 1452231_x_at | myeloid nuclear differentiation antigen like | *Mndal* | 2.49 | 0.67 |
| 1456132_x_at | TSC22 domain family, member 1 | *Tsc22d1* | 1.94 | 0.67 |
| 1426877_a_at | polybromo 1 | *Pbrm1* | 1.31 | 0.67 |
| 1424390_at | nucleoporin like 1 | *Nupl1* | 1.24 | 0.67 |
| 1423331_a_at | poliovirus receptor-related 3 | *Pvrl3* | 1.42 | 0.67 |
| 1434512_x_at | serine/arginine-rich splicing factor 3 | *Srsf3* | 1.53 | 0.66 |
| 1427831_s_at | zinc finger protein 260 | *Zfp260* | 1.42 | 0.66 |
| 1438673_at | solute carrier family 4, sodium bicarbonate cotransporter, member 7 | *Slc4a7* | 1.53 | 0.66 |
| 1441967_at | Parkinson disease 7 domain containing 1 | *Pddc1* | 1.29 | 0.66 |
| 1422005_at | eukaryotic translation initiation factor 2-alpha kinase 2 | *Eif2ak2* | 1.47 | 0.66 |
| 1444726_at | KRR1, small subunit (SSU) processome component, homolog (yeast) | *Krr1* | 1.42 | 0.66 |
| 1420668_a_at | Yip1 domain family, member 2 | *Yipf2* | 1.29 | 0.66 |
| 1434447_at | met proto-oncogene | *Met* | 1.71 | 0.66 |
| 1426756_at | UDP-N-acetyl-alpha-D-galactosamine:polypeptide N-acetylgalactosaminyltransferase 2 | *Galnt2* | 1.28 | 0.66 |
| 1460596_at | angiotensin II, type I receptor-associated protein | *Agtrap* | 1.22 | 0.66 |
| 1433645_at | solute carrier family 44, member 1 | *Slc44a1* | 1.79 | 0.66 |
| 1434312_at | ADP-ribosylation factor 6 | *Arf6* | 1.81 | 0.66 |
| 1455054_a_at | DCN1, defective in cullin neddylation 1, domain containing 1 (S. cerevisiae) | *Dcun1d1* | 1.27 | 0.66 |
| 1437761_at | LUC7-like 2 (S. cerevisiae) | *Luc7l2* | 1.26 | 0.66 |
| 1448037_at | Zinc finger, CCHC domain containing 6 | *Zcchc6* | 1.44 | 0.66 |
| 1426799_at | RAB8B, member RAS oncogene family | *Rab8b* | 2.21 | 0.66 |
| 1453283_at | phosphoglucomutase 1 | *Pgm1* | 1.67 | 0.66 |
| 1455687_at | intestinal cell kinase | *Ick* | 1.70 | 0.66 |
| 1455032_at | cyclin Y-like 1 | *Ccnyl1* | 1.72 | 0.66 |
| 1420817_at | tyrosine 3-monooxygenase/tryptophan 5-monooxygenase activation protein, gamma polypeptide | *Ywhag* | 1.37 | 0.66 |
| 1433702_at | endoplasmic reticulum metallopeptidase 1 | *Ermp1* | 2.28 | 0.66 |
| 1428154_s_at | phosphatidic acid phosphatase type 2 domain containing 1B | *Ppapdc1b* | 2.85 | 0.66 |
| 1431207_at | metallo-beta-lactamase domain containing 2 | *Mblac2* | 1.23 | 0.66 |
| 1449116_a_at | deoxythymidylate kinase | *Dtymk* | 1.50 | 0.66 |
| 1450850_at | ezrin | *Ezr* | 1.63 | 0.66 |
| 1455260_at | ligand dependent nuclear receptor corepressor-like | *Lcorl* | 1.39 | 0.66 |
| 1426263_at | cell adhesion molecule 4 | *Cadm4* | 1.23 | 0.66 |
| 1427670_a_at | transcription factor 12 | *Tcf12* | 1.71 | 0.66 |
| 1456485_at | nuclear protein in the AT region | *Npat* | 2.92 | 0.66 |
| 1416904_at | muscleblind-like 1 (Drosophila) | *Mbnl1* | 1.63 | 0.66 |
| 1433856_at | diphosphoinositol pentakisphosphate kinase 2 | *Ppip5k2* | 1.65 | 0.66 |
| 1434076_at | WD repeat domain 37 | *Wdr37* | 2.17 | 0.66 |
| 1451903_at | kynureninase (L-kynurenine hydrolase) | *Kynu* | 1.28 | 0.66 |
| 1452789_at | stannin | *Snn* | 1.91 | 0.66 |
| 1435437_at | SET domain containing (lysine methyltransferase) 7 | *Setd7* | 2.03 | 0.66 |
| 1451263_a_at | fatty acid binding protein 4, adipocyte | *Fabp4* | 2.30 | 0.66 |
| 1455206_at | ribosomal protein S6 kinase polypeptide 3 | *Rps6ka3* | 1.82 | 0.66 |
| 1458218_s_at | phosphodiesterase 7A | *Pde7a* | 1.88 | 0.66 |
| 1427177_at | FYVE and coiled-coil domain containing 1 | *Fyco1* | 1.23 | 0.66 |
| 1460044_at | one cut domain, family member 2 | *Onecut2* | 2.09 | 0.66 |
| 1460430_at | RAP2C, member of RAS oncogene family | *Rap2c* | 1.23 | 0.66 |
| 1419144_at | ceruloplasmin | *Cp* | 3.11 | 0.66 |
| 1451012_a_at | cold shock domain protein A | *Csda* | 1.53 | 0.66 |
| 1417460_at | interferon induced transmembrane protein 2 | *Ifitm2* | 3.62 | 0.66 |
| 1426988_at | kelch domain containing 5 | *Klhdc5* | 1.93 | 0.66 |
| 1417483_at | nuclear factor of kappa light polypeptide gene enhancer in B-cells inhibitor, zeta | *Nfkbiz* | 5.87 | 0.66 |
| 1435640_x_at | spermidine synthase | *Srm* | 1.76 | 0.66 |
| 1418099_at | tumor necrosis factor receptor superfamily, member 1b | *Tnfrsf1b* | 1.67 | 0.66 |
| 1447967_at | transmembrane protein 69 | *Tmem69* | 1.33 | 0.66 |
| 1452161_at | TCDD-inducible poly(ADP-ribose) polymerase | *Tiparp* | 1.34 | 0.66 |
| 1434022_at | zinc finger and BTB domain containing 33 | *Zbtb33* | 1.99 | 0.66 |
| 1452261_at | SNF2 histone linker PHD RING helicase | *Shprh* | 1.93 | 0.66 |
| 1457522_at | SMEK homolog 1, suppressor of mek1 (Dictyostelium) | *Smek1* | 1.34 | 0.66 |
| 1433851_at | protein phosphatase 4, regulatory subunit 2 | *Ppp4r2* | 1.25 | 0.66 |
| 1434901_at | zinc finger and BTB domain containing 2 | *Zbtb2* | 1.20 | 0.66 |
| 1454878_at | DAZ interacting protein 3, zinc finger | *Dzip3* | 2.14 | 0.66 |
| 1424443_at | transmembrane 6 superfamily member 1 | *Tm6sf1* | 4.52 | 0.65 |
| 1438583_at | Endoplasmic reticulum (ER) to nucleus signalling 1 | *Ern1* | 2.04 | 0.65 |
| 1438069_a_at | RNA binding motif protein 5 | *Rbm5* | 1.78 | 0.65 |
| 1418576_at | Yip1 domain family, member 5 | *Yipf5* | 1.90 | 0.65 |
| 1455102_at | La ribonucleoprotein domain family, member 4 | *Larp4* | 1.88 | 0.65 |
| 1426293_at | zinc finger protein 790 | *Zfp790* | 1.38 | 0.65 |
| 1427076_at | macrophage expressed gene 1 | *Mpeg1* | 2.83 | 0.65 |
| 1451335_at | placenta-specific 8 | *Plac8* | 2.20 | 0.65 |
| 1419586_at | retinitis pigmentosa 2 homolog (human) | *Rp2h* | 1.90 | 0.65 |
| 1456702_x_at | methionine adenosyltransferase II, alpha | *Mat2a* | 1.76 | 0.65 |
| 1438016_at | dyskeratosis congenita 1, dyskerin homolog (human) | *Dkc1* | 1.45 | 0.65 |
| 1416213_x_at | surfeit gene 4 | *Surf4* | 1.46 | 0.65 |
| 1435327_at | lysophosphatidylglycerol acyltransferase 1 | *Lpgat1* | 2.47 | 0.65 |
| 1435937_at | serine palmitoyltransferase, long chain base subunit 2 | *Sptlc2* | 1.88 | 0.65 |
| 1448550_at | lipopolysaccharide binding protein | *Lbp* | 6.00 | 0.65 |
| 1420475_at | myotrophin | *Mtpn* | 1.30 | 0.65 |
| 1426376_at | receptor accessory protein 5 | *Reep5* | 1.48 | 0.65 |
| 1426471_at | zinc finger protein 52 | *Zfp52* | 2.30 | 0.65 |
| 1433585_at | transportin 1 | *Tnpo1* | 1.50 | 0.65 |
| 1417472_at | myosin, heavy polypeptide 9, non-muscle | *Myh9* | 1.83 | 0.65 |
| 1429617_at | cylindromatosis (turban tumor syndrome) | *Cyld* | 1.66 | 0.65 |
| 1437207_at | ring finger protein 170 | *Rnf170* | 1.85 | 0.65 |
| 1423742_at | RNA binding motif protein 10 | *Rbm10* | 1.55 | 0.65 |
| 1419029_at | ERO1-like (S. cerevisiae) | *Ero1l* | 1.68 | 0.65 |
| 1433901_at | cell cycle associated protein 1 | *Caprin1* | 2.13 | 0.65 |
| 1417266_at | chemokine (C-C motif) ligand 6 | *Ccl6* | 3.48 | 0.65 |
| 1460085_at | Zinc finger protein 281 | *Zfp281* | 1.76 | 0.65 |
| 1439093_at | heat shock protein 4 like | *Hspa4l* | 1.35 | 0.65 |
| 1438686_at | eukaryotic translation initiation factor 4, gamma 1 | *Eif4g1* | 1.25 | 0.65 |
| 1452864_at | mediator of RNA polymerase II transcription, subunit 12 homolog (yeast)-like | *Med12l* | 1.93 | 0.65 |
| 1452454_at | SDA1 domain containing 1 | *Sdad1* | 1.70 | 0.65 |
| 1451396_at | protein-O-mannosyltransferase 2 | *Pomt2* | 1.63 | 0.65 |
| 1450786_x_at | PDZ and LIM domain 5 | *Pdlim5* | 1.57 | 0.65 |
| 1424333_at | RNA (guanine-9-) methyltransferase domain containing 1 | *Rg9mtd1* | 1.42 | 0.65 |
| 1426625_at | zinc finger protein 623 | *Zfp623* | 1.28 | 0.65 |
| 1434514_at | RNA binding motif protein 15 | *Rbm15* | 1.98 | 0.65 |
| 1426904_s_at | DnaJ (Hsp40) homolog, subfamily C, member 10 | *Dnajc10* | 1.88 | 0.65 |
| 1455234_at | UDP-Gal:betaGlcNAc beta 1,3-galactosyltransferase, polypeptide 1 | *B3galt1* | 2.44 | 0.65 |
| 1417786_a_at | regulator of G-protein signaling 19 | *Rgs19* | 1.89 | 0.65 |
| 1455165_at | RAR-related orphan receptor alpha | *Rora* | 1.39 | 0.65 |
| 1444004_at | THO complex 2 | *Thoc2* | 1.60 | 0.65 |
| 1423414_at | prostaglandin-endoperoxide synthase 1 | *Ptgs1* | 2.88 | 0.64 |
| 1454862_at | pleckstrin homology-like domain, family B, member 2 | *Phldb2* | 1.93 | 0.64 |
| 1434418_at | LAG1 homolog, ceramide synthase 6 | *Lass6* | 2.54 | 0.64 |
| 1434521_at | regulatory factor X, 7 | *Rfx7* | 1.46 | 0.64 |
| 1452163_at | E26 avian leukemia oncogene 1, 5' domain | *Ets1* | 1.45 | 0.64 |
| 1448703_at | N(alpha)-acetyltransferase 38, NatC auxiliary subunit | *Naa38* | 1.26 | 0.64 |
| 1436993_x_at | profilin 2 | *Pfn2* | 3.64 | 0.64 |
| 1419931_at | ATP-binding cassette, sub-family B (MDR/TAP), member 7 | *Abcb7* | 2.06 | 0.64 |
| 1454836_at | transmembrane protein 18 | *Tmem18* | 1.30 | 0.64 |
| 1437553_at | BRCA1/BRCA2-containing complex, subunit 3 | *Brcc3* | 1.37 | 0.64 |
| 1427604_a_at | ATPase, class II, type 9A | *Atp9a* | 1.49 | 0.64 |
| 1445534_at | Filamin, beta | *Flnb* | 2.05 | 0.64 |
| 1448320_at | stromal interaction molecule 1 | *Stim1* | 2.14 | 0.64 |
| 1439050_at | glutamate-cysteine ligase, modifier subunit | *Gclm* | 1.21 | 0.64 |
| 1435389_at | RALBP1 associated Eps domain containing protein 2 | *Reps2* | 1.75 | 0.64 |
| 1416603_at | ribosomal protein L22 | *Rpl22* | 1.77 | 0.64 |
| 1423152_at | vesicle-associated membrane protein, associated protein B and C | *Vapb* | 1.30 | 0.64 |
| 1448838_at | topoisomerase I binding, arginine/serine-rich | *Topors* | 1.30 | 0.64 |
| 1434839_s_at | transducin (beta)-like 1X-linked receptor 1 | *Tbl1xr1* | 1.47 | 0.64 |
| 1438413_at | SUMO1/sentrin specific peptidase 7 | *Senp7* | 1.48 | 0.64 |
| 1429796_at | kalirin, RhoGEF kinase | *Kalrn* | 1.21 | 0.64 |
| 1448763_at | ATPase family, AAA domain containing 1 | *Atad1* | 1.33 | 0.64 |
| 1420889_at | holocytochrome c synthetase | *Hccs* | 1.27 | 0.64 |
| 1435233_at | nuclear receptor coactivator 2 | *Ncoa2* | 1.55 | 0.64 |
| 1452126_at | zinc finger protein 160 | *Zfp160* | 2.05 | 0.64 |
| 1442176_at | AT rich interactive domain 5B (MRF1-like) | *Arid5b* | 1.21 | 0.64 |
| 1457753_at | toll-like receptor 13 | *Tlr13* | 3.00 | 0.64 |
| 1429216_at | progestin and adipoQ receptor family member III | *Paqr3* | 1.29 | 0.64 |
| 1419641_at | purine rich element binding protein B | *Purb* | 1.22 | 0.64 |
| 1450904_at | transmembrane protein 167 | *Tmem167* | 2.12 | 0.64 |
| 1428287_at | cullin 5 | *Cul5* | 1.25 | 0.64 |
| 1428453_at | N(alpha)-acetyltransferase 30, NatC catalytic subunit | *Naa30* | 1.50 | 0.64 |
| 1420498_a_at | disabled homolog 2 (Drosophila) | *Dab2* | 1.77 | 0.64 |
| 1449394_at | solute carrier organic anion transporter family, member 1b2 | *Slco1b2* | 1.24 | 0.64 |
| 1428546_at | synaptotagmin binding, cytoplasmic RNA interacting protein | *Syncrip* | 1.80 | 0.64 |
| 1423444_at | Rho-associated coiled-coil containing protein kinase 1 | *Rock1* | 1.64 | 0.64 |
| 1430145_at | leucine rich repeat containing 28 | *Lrrc28* | 1.22 | 0.64 |
| 1451204_at | glycoprotein m6b | *Gpm6b* | 7.66 | 0.64 |
| 1433730_at | ELMO domain containing 2 | *Elmod2* | 1.93 | 0.64 |
| 1435241_at | HEAT repeat containing 5A | *Heatr5a* | 1.45 | 0.64 |
| 1448775_at | interferon activated gene 203 | *Ifi203* | 2.09 | 0.64 |
| 1426771_at | taurine upregulated gene 1 | *Tug1* | 1.56 | 0.64 |
| 1420760_s_at | N-myc downstream regulated gene 1 | *Ndrg1* | 1.60 | 0.64 |
| 1451350_a_at | leptin receptor overlapping transcript | *Leprot* | 1.30 | 0.64 |
| 1421840_at | ATP-binding cassette, sub-family A (ABC1), member 1 | *Abca1* | 1.63 | 0.64 |
| 1438435_at | alkaline ceramidase 3 | *Acer3* | 1.38 | 0.64 |
| 1449414_at | zinc finger protein 53 | *Zfp53* | 2.31 | 0.64 |
| 1452857_at | CREB/ATF bZIP transcription factor | *Crebzf* | 2.15 | 0.64 |
| 1452657_at | adaptor-related protein complex 1, sigma 2 subunit | *Ap1s2* | 2.38 | 0.63 |
| 1456060_at | avian musculoaponeurotic fibrosarcoma (v-maf) AS42 oncogene homolog | *Maf* | 2.27 | 0.63 |
| 1434344_at | G patch domain and KOW motifs | *Gpkow* | 1.67 | 0.63 |
| 1418502_a_at | oxidation resistance 1 | *Oxr1* | 1.31 | 0.63 |
| 1427159_at | cleavage and polyadenylation factor subunit homolog (S. cerevisiae) | *Pcf11* | 1.26 | 0.63 |
| 1435077_at | additional sex combs like 1 (Drosophila) | *Asxl1* | 1.69 | 0.63 |
| 1421054_at | exportin 4 | *Xpo4* | 2.32 | 0.63 |
| 1448548_at | tubby like protein 4 | *Tulp4* | 1.40 | 0.63 |
| 1455899_x_at | suppressor of cytokine signaling 3 | *Socs3* | 3.26 | 0.63 |
| 1455437_at | SIK family kinase 3 | *Sik3* | 1.51 | 0.63 |
| 1417357_at | emerin | *Emd* | 1.40 | 0.63 |
| 1420618_at | cytoplasmic polyadenylation element binding protein 4 | *Cpeb4* | 1.64 | 0.63 |
| 1427100_at | meteorin, glial cell differentiation regulator | *Metrn* | 2.00 | 0.63 |
| 1448985_at | dual specificity phosphatase 22 | *Dusp22* | 1.39 | 0.63 |
| 1434111_at | latrophilin 2 | *Lphn2* | 1.81 | 0.63 |
| 1428490_at | core 1 synthase, glycoprotein-N-acetylgalactosamine 3-beta-galactosyltransferase, 1 | *C1galt1* | 1.25 | 0.63 |
| 1428839_at | WD repeat domain 53 | *Wdr53* | 1.98 | 0.63 |
| 1426545_at | trinucleotide repeat containing 6b | *Tnrc6b* | 1.27 | 0.63 |
| 1450688_at | ral guanine nucleotide dissociation stimulator-like 2 | *Rgl2* | 2.29 | 0.63 |
| 1417241_at | archaelysin family metallopeptidase 2 | *Amz2* | 1.49 | 0.63 |
| 1428762_at | TGF-beta activated kinase 1/MAP3K7 binding protein 3 | *Tab3* | 1.44 | 0.63 |
| 1417985_at | Notch-regulated ankyrin repeat protein | *Nrarp* | 1.23 | 0.63 |
| 1452360_a_at | lysine (K)-specific demethylase 5A | *Kdm5a* | 1.41 | 0.63 |
| 1460200_s_at | leucine zipper transcription factor-like 1 | *Lztfl1* | 1.64 | 0.63 |
| 1435248_a_at | BTAF1 RNA polymerase II, B-TFIID transcription factor-associated, (Mot1 homolog, S. cerevisiae) | *Btaf1* | 1.96 | 0.63 |
| 1420381_a_at | ribosomal protein L31 | *Rpl31* | 2.19 | 0.63 |
| 1426489_s_at | bifunctional apoptosis regulator | *Bfar* | 1.25 | 0.63 |
| 1438018_at | hook homolog 1 (Drosophila) | *Hook1* | 2.07 | 0.63 |
| 1437267_x_at | Heterogeneous nuclear ribonucleoprotein H1 | *Hnrnph1* | 1.21 | 0.63 |
| 1436042_at | talin 1 | *Tln1* | 1.44 | 0.63 |
| 1424986_s_at | F-box and WD-40 domain protein 7 | *Fbxw7* | 1.33 | 0.63 |
| 1453796_a_at | ERGIC and golgi 2 | *Ergic2* | 1.31 | 0.63 |
| 1422094_a_at | zinc finger protein 329 | *Zfp329* | 1.33 | 0.63 |
| 1453172_at | heat shock protein 70 family, member 13 | *Hspa13* | 1.70 | 0.63 |
| 1454882_at | l(3)mbt-like 3 (Drosophila) | *L3mbtl3* | 1.95 | 0.63 |
| 1458831_at | NAD(P) dependent steroid dehydrogenase-like | *Nsdhl* | 1.28 | 0.63 |
| 1429152_at | zinc finger with KRAB and SCAN domains 1 | *Zkscan1* | 1.91 | 0.63 |
| 1433568_at | PAP associated domain containing 4 | *Papd4* | 1.50 | 0.63 |
| 1420928_at | beta galactoside alpha 2,6 sialyltransferase 1 | *St6gal1* | 3.18 | 0.63 |
| 1449791_x_at | solute carrier family 38, member 9 | *Slc38a9* | 1.93 | 0.63 |
| 1419279_at | phosphatidylinositol-5-phosphate 4-kinase, type II, alpha | *Pip4k2a* | 2.43 | 0.63 |
| 1418561_at | splicing factor 3b, subunit 1 | *Sf3b1* | 1.57 | 0.63 |
| 1417111_at | mannosidase 1, alpha | *Man1a* | 1.51 | 0.63 |
| 1456097_a_at | integrin beta 3 binding protein (beta3-endonexin) | *Itgb3bp* | 1.65 | 0.63 |
| 1450644_at | zinc finger protein 36, C3H type-like 1 | *Zfp36l1* | 1.72 | 0.63 |
| 1434458_at | follistatin | *Fst* | 1.40 | 0.63 |
| 1437983_at | sal-like 1 (Drosophila) | *Sall1* | 1.81 | 0.62 |
| 1434005_at | RNA binding motif, single stranded interacting protein 1 | *Rbms1* | 1.28 | 0.62 |
| 1433649_at | lysine (K)-specific demethylase 1B | *Kdm1b* | 1.52 | 0.62 |
| 1418516_at | metal response element binding transcription factor 2 | *Mtf2* | 1.36 | 0.62 |
| 1442048_at | ring finger protein 11 | *Rnf11* | 1.28 | 0.62 |
| 1435775_at | circadian locomotor output cycles kaput | *Clock* | 1.93 | 0.62 |
| 1449839_at | caspase 3 | *Casp3* | 1.43 | 0.62 |
| 1439010_at | La ribonucleoprotein domain family, member 4 | *Larp4* | 1.56 | 0.62 |
| 1455688_at | discoidin domain receptor family, member 2 | *Ddr2* | 2.85 | 0.62 |
| 1426609_at | DIS3 mitotic control homolog (S. cerevisiae) | *Dis3* | 1.53 | 0.62 |
| 1456162_x_at | adducin 3 (gamma) | *Add3* | 1.97 | 0.62 |
| 1422549_at | ADP-ribosylation factor-like 2 | *Arl2* | 1.30 | 0.62 |
| 1434170_at | DDB1 and CUL4 associated factor 12-like 1 | *Dcaf12l1* | 1.38 | 0.62 |
| 1454740_at | mindbomb homolog 1 (Drosophila) | *Mib1* | 1.80 | 0.62 |
| 1452214_at | SKI-like | *Skil* | 2.38 | 0.62 |
| 1419589_at | CD93 antigen | *Cd93* | 2.10 | 0.62 |
| 1424474_a_at | calcium/calmodulin-dependent protein kinase kinase 2, beta | *Camkk2* | 1.30 | 0.62 |
| 1424684_at | RAB5C, member RAS oncogene family | *Rab5c* | 2.11 | 0.62 |
| 1452139_at | solute carrier family 35, member C1 | *Slc35c1* | 1.91 | 0.62 |
| 1435010_at | ankyrin repeat and SOCS box-containing 7 | *Asb7* | 1.70 | 0.62 |
| 1429711_at | serine/threonine/tyrosine interaction protein | *Styx* | 1.86 | 0.62 |
| 1427434_at | NLR family, apoptosis inhibitory protein 6 | *Naip6* | 2.01 | 0.62 |
| 1453004_at | solute carrier family 22, member 23 | *Slc22a23* | 1.42 | 0.62 |
| 1441855_x_at | BMP-binding endothelial regulator | *Bmper* | 9.63 | 0.62 |
| 1457248_x_at | hydroxysteroid (17-beta) dehydrogenase 7 | *Hsd17b7* | 1.84 | 0.62 |
| 1450034_at | signal transducer and activator of transcription 1 | *Stat1* | 1.92 | 0.62 |
| 1442003_at | diaphanous homolog 2 (Drosophila) | *Diap2* | 1.26 | 0.62 |
| 1430820_a_at | bobby sox homolog (Drosophila) | *Bbx* | 1.22 | 0.62 |
| 1450039_at | ubiquitin specific peptidase 9, X chromosome | *Usp9x* | 1.32 | 0.62 |
| 1426357_at | TAO kinase 1 | *Taok1* | 1.54 | 0.62 |
| 1431134_at | inhibitor of growth family, member 5 | *Ing5* | 1.47 | 0.62 |
| 1427114_at | tetratricopeptide repeat domain 19 | *Ttc19* | 1.45 | 0.62 |
| 1421962_at | DnaJ (Hsp40) homolog, subfamily B, member 5 | *Dnajb5* | 1.50 | 0.62 |
| 1415941_s_at | zinc finger, AN1-type domain 2A | *Zfand2a* | 2.69 | 0.62 |
| 1434284_at | B double prime 1, subunit of RNA polymerase III transcription initiation factor IIIB | *Bdp1* | 1.64 | 0.62 |
| 1451179_a_at | quaking | *Qk* | 1.97 | 0.62 |
| 1437197_at | sorbin and SH3 domain containing 2 | *Sorbs2* | 1.85 | 0.62 |
| 1436705_at | membrane magnesium transporter 1 | *Mmgt1* | 1.49 | 0.62 |
| 1440223_at | RNA binding motif protein 6 | *Rbm6* | 1.76 | 0.62 |
| 1455320_at | nicotinamide phosphoribosyltransferase | *Nampt* | 1.42 | 0.62 |
| 1437313_x_at | high mobility group box 2 | *Hmgb2* | 2.15 | 0.62 |
| 1425603_at | transmembrane protein 176A | *Tmem176a* | 4.53 | 0.62 |
| 1441973_at | zinc finger protein 295 | *Zfp295* | 1.57 | 0.62 |
| 1429045_at | SMAD specific E3 ubiquitin protein ligase 2 | *Smurf2* | 1.50 | 0.62 |
| 1418930_at | selectin, platelet | *Selp* | 1.25 | 0.62 |
| 1442873_at | fidgetin | *Fign* | 2.95 | 0.61 |
| 1436270_at | cytochrome b5 domain containing 1 | *Cyb5d1* | 1.42 | 0.61 |
| 1451004_at | activin receptor IIA | *Acvr2a* | 1.63 | 0.61 |
| 1431830_at | zinc finger protein 329 | *Zfp329* | 2.03 | 0.61 |
| 1416031_s_at | minichromosome maintenance deficient 7 (S. cerevisiae) | *Mcm7* | 2.07 | 0.61 |
| 1455393_at | transforming growth factor, beta receptor III | *Tgfbr3* | 3.37 | 0.61 |
| 1435717_at | solute carrier family 30 (zinc transporter), member 7 | *Slc30a7* | 1.94 | 0.61 |
| 1435597_at | ATPase family, AAA domain containing 5 | *Atad5* | 1.55 | 0.61 |
| 1441033_at | transmembrane and tetratricopeptide repeat containing 2 | *Tmtc2* | 1.49 | 0.61 |
| 1428639_at | lin-9 homolog (C. elegans) | *Lin9* | 2.00 | 0.61 |
| 1437586_at | CCR4-NOT transcription complex, subunit 4 | *Cnot4* | 1.42 | 0.61 |
| 1426346_at | prolyl endopeptidase-like | *Prepl* | 1.87 | 0.61 |
| 1452418_at | major facilitator superfamily domain containing 2A | *Mfsd2a* | 3.34 | 0.61 |
| 1436161_at | PDS5, regulator of cohesion maintenance, homolog B (S. cerevisiae) | *Pds5b* | 1.54 | 0.61 |
| 1434706_at | valosin containing protein (p97)/p47 complex interacting protein 1 | *Vcpip1* | 2.12 | 0.61 |
| 1423033_at | STT3, subunit of the oligosaccharyltransferase complex, homolog A (S. cerevisiae) | *Stt3a* | 2.09 | 0.61 |
| 1422146_at | sema domain, seven thrombospondin repeats (type 1 and type 1-like), transmembrane domain (TM) and short cytoplasmic domain, (semaphorin) 5B | *Sema5b* | 1.31 | 0.61 |
| 1433738_at | PAP associated domain containing 5 | *Papd5* | 1.41 | 0.61 |
| 1452154_at | isoleucine-tRNA synthetase | *Iars* | 1.66 | 0.61 |
| 1424344_s_at | eukaryotic translation initiation factor 1A | *Eif1a* | 2.11 | 0.61 |
| 1454956_at | ribosomal protein S6 kinase, polypeptide 1 | *Rps6kb1* | 1.86 | 0.61 |
| 1450876_at | complement component factor h | *Cfh* | 2.84 | 0.61 |
| 1454703_x_at | small nucleolar RNA host gene (non-protein coding) 1 | *Snhg1* | 1.53 | 0.61 |
| 1437467_at | activated leukocyte cell adhesion molecule | *Alcam* | 1.81 | 0.61 |
| 1439272_at | ligand dependent nuclear receptor corepressor-like | *Lcorl* | 1.45 | 0.61 |
| 1416731_at | topoisomerase (DNA) II beta | *Top2b* | 1.76 | 0.61 |
| 1431293_a_at | claudin domain containing 1 | *Cldnd1* | 1.44 | 0.61 |
| 1456102_a_at | cullin 5 | *Cul5* | 1.38 | 0.61 |
| 1417292_at | interferon gamma inducible protein 47 | *Ifi47* | 1.75 | 0.61 |
| 1455324_at | phosphatidylinositol-specific phospholipase C, X domain containing 2 | *Plcxd2* | 1.51 | 0.61 |
| 1438661_a_at | ADP-ribosylation factor 2 | *Arf2* | 1.33 | 0.61 |
| 1437992_x_at | gap junction protein, alpha 1 | *Gja1* | 3.46 | 0.61 |
| 1455009_at | carboxypeptidase D | *Cpd* | 1.98 | 0.61 |
| 1449852_a_at | EH-domain containing 4 | *Ehd4* | 2.44 | 0.61 |
| 1421977_at | transferrin receptor | *Tfrc* | 1.46 | 0.61 |
| 1437375_at | regulatory factor X, 3 (influences HLA class II expression) | *Rfx3* | 1.28 | 0.61 |
| 1435254_at | plexin B1 | *Plxnb1* | 1.29 | 0.61 |
| 1423349_at | suppressor of cytokine signaling 5 | *Socs5* | 1.32 | 0.61 |
| 1416611_at | secretory carrier membrane protein 2 | *Scamp2* | 1.28 | 0.61 |
| 1444157_a_at | lysine (K)-specific demethylase 5C | *Kdm5c* | 1.23 | 0.61 |
| 1426682_at | CCR4-NOT transcription complex, subunit 6 | *Cnot6* | 1.62 | 0.61 |
| 1448944_at | neuropilin 1 | *Nrp1* | 1.26 | 0.60 |
| 1460402_at | bromodomain and PHD finger containing, 1 | *Brpf1* | 1.49 | 0.60 |
| 1419248_at | regulator of G-protein signaling 2 | *Rgs2* | 5.55 | 0.60 |
| 1457644_s_at | interleukin 1 receptor, type I | *Il1r1* | 19.73 | 0.60 |
| 1426613_a_at | U2 small nuclear ribonucleoprotein B | *Snrpb2* | 1.24 | 0.60 |
| 1450681_at | zinc finger protein 143 | *Zfp143* | 1.56 | 0.60 |
| 1450090_at | zinc finger protein 101 | *Zfp101* | 1.26 | 0.60 |
| 1427730_a_at | zinc finger protein 148 | *Zfp148* | 1.44 | 0.60 |
| 1423257_at | cytochrome P450, family 4, subfamily a, polypeptide 14 | *Cyp4a14* | 9.49 | 0.60 |
| 1439109_at | coiled-coil domain containing 68 | *Ccdc68* | 1.88 | 0.60 |
| 1425722_at | interferon inducible GTPase 1B | *Iigp1b* | 1.58 | 0.60 |
| 1436558_at | TATA box binding protein (Tbp)-associated factor, RNA polymerase I, D | *Taf1d* | 2.38 | 0.60 |
| 1458504_at | zinc finger CCCH type containing 12D | *Zc3h12d* | 1.30 | 0.60 |
| 1456533_at | dpy-19-like 1 (C. elegans) | *Dpy19l1* | 1.43 | 0.60 |
| 1428446_at | dynein cytoplasmic 2 light intermediate chain 1 | *Dync2li1* | 1.50 | 0.60 |
| 1428095_a_at | C2 calcium-dependent domain containing 2-like | *C2cd2l* | 1.38 | 0.60 |
| 1428721_at | kelch-like 28 (Drosophila) | *Klhl28* | 1.36 | 0.60 |
| 1453097_a_at | upstream binding transcription factor, RNA polymerase I | *Ubtf* | 1.33 | 0.60 |
| 1421322_a_at | interferon regulatory factor 9 | *Irf9* | 1.38 | 0.60 |
| 1434179_at | myeloid/lymphoid or mixed-lineage leukemia 3 | *Mll3* | 1.24 | 0.60 |
| 1457357_at | tousled-like kinase 2 (Arabidopsis) | *Tlk2* | 1.89 | 0.60 |
| 1427881_at | deoxynucleotidyltransferase, terminal, interacting protein 2 | *Dnttip2* | 1.64 | 0.60 |
| 1454736_at | ankyrin repeat domain 57 | *Ankrd57* | 2.36 | 0.60 |
| 1420549_at | guanylate binding protein 1 | *Gbp1* | 1.64 | 0.60 |
| 1429434_at | phosphatidylinositol 3-kinase, catalytic, alpha polypeptide | *Pik3ca* | 1.36 | 0.60 |
| 1453253_a_at | RNA pseudouridylate synthase domain containing 1 | *Rpusd1* | 1.44 | 0.60 |
| 1436595_at | RNA binding motif protein 34 | *Rbm34* | 1.69 | 0.60 |
| 1421480_a_at | adenosine deaminase, RNA-specific, B1 | *Adarb1* | 1.54 | 0.60 |
| 1420384_at | collagen, type IV, alpha 3 (Goodpasture antigen) binding protein | *Col4a3bp* | 1.28 | 0.60 |
| 1439058_at | splicing factor proline/glutamine rich (polypyrimidine tract binding protein associated) | *Sfpq* | 1.53 | 0.60 |
| 1439662_at | homer homolog 1 (Drosophila) | *Homer1* | 1.27 | 0.60 |
| 1428829_at | DENN/MADD domain containing 1B | *Dennd1b* | 1.53 | 0.59 |
| 1435009_at | solute carrier family 9 (sodium/hydrogen exchanger), member 6 | *Slc9a6* | 1.70 | 0.59 |
| 1451518_at | zinc finger protein 709 | *Zfp709* | 1.86 | 0.59 |
| 1435801_at | fukutin | *Fktn* | 1.75 | 0.59 |
| 1452008_at | tetratricopeptide repeat domain 39B | *Ttc39b* | 1.69 | 0.59 |
| 1418210_at | profilin 2 | *Pfn2* | 2.72 | 0.59 |
| 1452784_at | integrin alpha V | *Itgav* | 3.22 | 0.59 |
| 1448885_at | RAP2B, member of RAS oncogene family | *Rap2b* | 1.68 | 0.59 |
| 1429455_at | GTPase activating protein and VPS9 domains 1 | *Gapvd1* | 1.35 | 0.59 |
| 1428847_a_at | microtubule-actin crosslinking factor 1 | *Macf1* | 1.99 | 0.59 |
| 1435701_at | cysteine and tyrosine-rich protein 1 | *Cyyr1* | 1.29 | 0.59 |
| 1435043_at | phospholipase C, beta 1 | *Plcb1* | 1.97 | 0.59 |
| 1455189_at | tripartite motif-containing 33 | *Trim33* | 1.21 | 0.59 |
| 1417743_at | CDC-like kinase 2 | *Clk2* | 1.37 | 0.59 |
| 1417926_at | non-SMC condensin II complex, subunit G2 | *Ncapg2* | 2.53 | 0.59 |
| 1425435_at | macrophage scavenger receptor 1 | *Msr1* | 1.95 | 0.59 |
| 1427883_a_at | collagen, type III, alpha 1 | *Col3a1* | 1.65 | 0.59 |
| 1429082_at | ankyrin repeat and LEM domain containing 2 | *Ankle2* | 1.40 | 0.59 |
| 1449217_at | caspase 8 associated protein 2 | *Casp8ap2* | 1.67 | 0.59 |
| 1434394_at | NEDD4 binding protein 2 | *N4bp2* | 1.43 | 0.59 |
| 1433759_at | dpy-19-like 1 (C. elegans) | *Dpy19l1* | 1.94 | 0.59 |
| 1455191_x_at | phosphatidylinositol-4-phosphate 5-kinase, type 1 alpha | *Pip5k1a* | 1.54 | 0.59 |
| 1424084_at | ROD1 regulator of differentiation 1 (S. pombe) | *Rod1* | 1.82 | 0.59 |
| 1455851_at | bone morphogenetic protein 5 | *Bmp5* | 1.27 | 0.59 |
| 1438397_a_at | RNA binding motif protein 39 | *Rbm39* | 1.51 | 0.59 |
| 1437357_at | YTH domain containing 2 | *Ythdc2* | 1.84 | 0.59 |
| 1452353_at | G protein-coupled receptor 155 | *Gpr155* | 1.47 | 0.59 |
| 1453136_at | F-box protein 30 | *Fbxo30* | 2.04 | 0.59 |
| 1426841_at | YTH domain family 3 | *Ythdf3* | 1.46 | 0.59 |
| 1436707_x_at | non-SMC condensin I complex, subunit H | *Ncaph* | 2.83 | 0.59 |
| 1423194_at | Rho GTPase activating protein 5 | *Arhgap5* | 1.32 | 0.59 |
| 1421266_s_at | nuclear factor of kappa light polypeptide gene enhancer in B-cells inhibitor, beta | *Nfkbib* | 1.52 | 0.59 |
| 1419417_at | vascular endothelial growth factor C | *Vegfc* | 1.68 | 0.58 |
| 1451777_at | DEAD (Asp-Glu-Ala-Asp) box polypeptide 60 | *Ddx60* | 1.28 | 0.58 |
| 1456288_at | schlafen 5 | *Slfn5* | 1.60 | 0.58 |
| 1455505_at | GATA zinc finger domain containing 2A | *Gatad2a* | 1.31 | 0.58 |
| 1422553_at | phosphatase and tensin homolog | *Pten* | 1.61 | 0.58 |
| 1452901_at | cAMP responsive element binding protein 1 | *Creb1* | 1.46 | 0.58 |
| 1420941_at | regulator of G-protein signaling 5 | *Rgs5* | 1.44 | 0.58 |
| 1433443_a_at | 3-hydroxy-3-methylglutaryl-Coenzyme A synthase 1 | *Hmgcs1* | 2.65 | 0.58 |
| 1451168_a_at | Rho GDP dissociation inhibitor (GDI) alpha | *Arhgdia* | 1.41 | 0.58 |
| 1421321_a_at | neuroepithelial cell transforming gene 1 | *Net1* | 1.93 | 0.58 |
| 1431804_a_at | trans-acting transcription factor 3 | *Sp3* | 1.87 | 0.58 |
| 1428715_at | glutamine fructose-6-phosphate transaminase 1 | *Gfpt1* | 2.44 | 0.58 |
| 1434933_at | RING CCCH (C3H) domains 1 | *Rc3h1* | 1.55 | 0.58 |
| 1425514_at | phosphatidylinositol 3-kinase, regulatory subunit, polypeptide 1 (p85 alpha) | *Pik3r1* | 1.66 | 0.58 |
| 1430999_a_at | short coiled-coil protein | *Scoc* | 1.64 | 0.58 |
| 1436427_at | PRP4 pre-mRNA processing factor 4 homolog B (yeast) | *Prpf4b* | 1.71 | 0.58 |
| 1451783_a_at | kinesin-associated protein 3 | *Kifap3* | 1.36 | 0.58 |
| 1438268_at | ring finger and CCCH-type zinc finger domains 2 | *Rc3h2* | 1.92 | 0.58 |
| 1422706_at | prostate transmembrane protein, androgen induced 1 | *Pmepa1* | 3.69 | 0.58 |
| 1418017_at | pumilio 2 (Drosophila) | *Pum2* | 1.41 | 0.58 |
| 1456087_at | nuclear factor I/A | *Nfia* | 1.24 | 0.58 |
| 1431241_at | coiled-coil-helix-coiled-coil-helix domain containing 3 | *Chchd3* | 1.28 | 0.58 |
| 1450392_at | ATP-binding cassette, sub-family A (ABC1), member 1 | *Abca1* | 1.31 | 0.58 |
| 1419575_s_at | zinc finger protein 292 | *Zfp292* | 1.59 | 0.58 |
| 1426911_at | desmocollin 2 | *Dsc2* | 1.42 | 0.58 |
| 1419565_a_at | zinc finger protein X-linked | *Zfx* | 1.55 | 0.58 |
| 1423220_at | eukaryotic translation initiation factor 4E | *Eif4e* | 1.34 | 0.58 |
| 1427273_at | ring finger protein 214 | *Rnf214* | 1.49 | 0.58 |
| 1439089_at | zinc finger and BTB domain containing 41 homolog | *Zbtb41* | 2.08 | 0.58 |
| 1419984_s_at | zinc finger protein 644 | *Zfp644* | 1.77 | 0.58 |
| 1455966_s_at | nudix (nucleoside diphosphate linked moiety X)-type motif 21 | *Nudt21* | 1.20 | 0.58 |
| 1448828_at | structural maintenance of chromosomes 6 | *Smc6* | 1.21 | 0.58 |
| 1452462_a_at | BTG3 associated nuclear protein | *Banp* | 1.69 | 0.58 |
| 1460614_at | mesoderm induction early response 1, family member 3 | *Mier3* | 1.23 | 0.58 |
| 1454084_a_at | SUMO/sentrin specific peptidase 8 | *Senp8* | 1.25 | 0.58 |
| 1454961_at | synaptojanin 1 | *Synj1* | 2.27 | 0.58 |
| 1436217_at | zinc finger protein 148 | *Zfp148* | 1.24 | 0.58 |
| 1449558_at | coagulation factor VIII | *F8* | 1.24 | 0.58 |
| 1453000_at | calmodulin regulated spectrin-associated protein 1-like 1 | *Camsap1l1* | 1.29 | 0.58 |
| 1448406_at | EP300 interacting inhibitor of differentiation 1 | *Eid1* | 1.94 | 0.57 |
| 1455037_at | plexin A2 | *Plxna2* | 1.57 | 0.57 |
| 1417379_at | IQ motif containing GTPase activating protein 1 | *Iqgap1* | 2.34 | 0.57 |
| 1436186_at | E2F transcription factor 8 | *E2f8* | 2.79 | 0.57 |
| 1431932_s_at | tripartite motif-containing 44 | *Trim44* | 1.34 | 0.57 |
| 1426503_a_at | ring finger protein 121 | *Rnf121* | 4.22 | 0.57 |
| 1416682_at | ubiquitin protein ligase E3A | *Ube3a* | 1.88 | 0.57 |
| 1433640_at | far upstream element (FUSE) binding protein 1 | *Fubp1* | 2.35 | 0.57 |
| 1428779_at | zinc finger and BTB domain containing 41 homolog | *Zbtb41* | 1.68 | 0.57 |
| 1434746_at | MAX gene associated | *Mga* | 2.12 | 0.57 |
| 1424880_at | tribbles homolog 1 (Drosophila) | *Trib1* | 1.82 | 0.57 |
| 1424884_at | F-box and WD-40 domain protein 2 | *Fbxw2* | 1.47 | 0.57 |
| 1420522_at | coiled-coil domain containing 50 | *Ccdc50* | 1.50 | 0.57 |
| 1431146_a_at | copine VIII | *Cpne8* | 3.25 | 0.57 |
| 1415976_a_at | calcium regulated heat stable protein 1 | *Carhsp1* | 1.21 | 0.57 |
| 1455534_s_at | oxysterol binding protein-like 11 | *Osbpl11* | 1.46 | 0.57 |
| 1460602_at | deleted in liver cancer 1 | *Dlc1* | 1.35 | 0.57 |
| 1429060_at | metastasis associated lung adenocarcinoma transcript 1 (non-coding RNA) | *Malat1* | 1.66 | 0.57 |
| 1454834_at | nuclear factor I/B | *Nfib* | 1.69 | 0.57 |
| 1417976_at | adenosine deaminase | *Ada* | 2.72 | 0.57 |
| 1424709_at | sterol-C5-desaturase (fungal ERG3, delta-5-desaturase) homolog (S. cerevisae) | *Sc5d* | 1.63 | 0.57 |
| 1434705_at | C-terminal binding protein 2 | *Ctbp2* | 1.67 | 0.57 |
| 1426314_at | endothelin receptor type B | *Ednrb* | 1.20 | 0.57 |
| 1437728_at | alkB, alkylation repair homolog 5 (E. coli) | *Alkbh5* | 1.27 | 0.57 |
| 1437329_at | protein tyrosine phosphatase-like (proline instead of catalytic arginine), member b | *Ptplb* | 1.24 | 0.57 |
| 1438404_at | ring finger protein 144A | *Rnf144a* | 1.29 | 0.57 |
| 1422631_at | aryl-hydrocarbon receptor | *Ahr* | 3.76 | 0.57 |
| 1428234_at | cleavage and polyadenylation specific factor 6 | *Cpsf6* | 1.86 | 0.57 |
| 1455251_at | integrin alpha 1 | *Itga1* | 2.12 | 0.57 |
| 1434827_at | THO complex 6 homolog (Drosophila) | *Thoc6* | 2.07 | 0.57 |
| 1421392_a_at | baculoviral IAP repeat-containing 3 | *Birc3* | 1.26 | 0.57 |
| 1439260_a_at | ectonucleotide pyrophosphatase/phosphodiesterase 3 | *Enpp3* | 1.68 | 0.57 |
| 1453215_at | ribonuclease, RNase A family, 10 (non-active) | *Rnase10* | 1.72 | 0.57 |
| 1423674_at | ubiquitin specific peptidase 1 | *Usp1* | 1.84 | 0.57 |
| 1456316_a_at | acyl-Coenzyme A binding domain containing 3 | *Acbd3* | 1.58 | 0.57 |
| 1460438_at | LysM, putative peptidoglycan-binding, domain containing 1 | *Lysmd1* | 1.35 | 0.57 |
| 1420514_at | transmembrane protein 47 | *Tmem47* | 4.57 | 0.57 |
| 1452445_at | solute carrier family 41, member 2 | *Slc41a2* | 8.00 | 0.57 |
| 1435137_s_at | cytochrome P450, family 4, subfamily f, polypeptide 18 | *Cyp4f18* | 1.64 | 0.57 |
| 1450928_at | inhibitor of DNA binding 4 | *Id4* | 2.50 | 0.57 |
| 1423143_at | GTP binding protein 4 | *Gtpbp4* | 1.24 | 0.57 |
| 1423153_x_at | complement component factor h | *Cfh* | 3.08 | 0.57 |
| 1458399_at | leucine rich repeat containing 3 | *Lrrc3* | 2.25 | 0.56 |
| 1423620_at | centromere protein Q | *Cenpq* | 1.61 | 0.56 |
| 1427007_at | SAM and SH3 domain containing 3 | *Sash3* | 2.35 | 0.56 |
| 1429000_at | PHD finger protein 3 | *Phf3* | 1.73 | 0.56 |
| 1449933_a_at | tRNA splicing endonuclease 15 homolog (S. cerevisiae) | *Tsen15* | 1.23 | 0.56 |
| 1458550_at | myosin ID | *Myo1d* | 1.50 | 0.56 |
| 1448793_a_at | syndecan 4 | *Sdc4* | 1.51 | 0.56 |
| 1425379_at | hepatocyte growth factor | *Hgf* | 1.80 | 0.56 |
| 1418774_a_at | ATPase, Cu++ transporting, alpha polypeptide | *Atp7a* | 1.67 | 0.56 |
| 1438693_at | transmembrane protein 110 | *Tmem110* | 1.74 | 0.56 |
| 1459971_at | potassium channel, subfamily T, member 2 | *Kcnt2* | 7.61 | 0.56 |
| 1453435_a_at | flavin containing monooxygenase 2 | *Fmo2* | 1.72 | 0.56 |
| 1420106_at | Seven in absentia 1A | *Siah1a* | 1.76 | 0.56 |
| 1423291_s_at | hypoxia up-regulated 1 | *Hyou1* | 1.26 | 0.56 |
| 1429505_at | neurobeachin like 1 | *Nbeal1* | 1.79 | 0.56 |
| 1454904_at | X-linked myotubular myopathy gene 1 | *Mtm1* | 1.40 | 0.56 |
| 1418585_at | cyclin H | *Ccnh* | 2.15 | 0.56 |
| 1453993_a_at | BCL2/adenovirus E1B interacting protein 2 | *Bnip2* | 1.94 | 0.56 |
| 1450943_at | mago-nashi homolog B (Drosophila) | *Magohb* | 1.31 | 0.56 |
| 1455556_at | Notch gene homolog 2 (Drosophila) | *Notch2* | 2.01 | 0.56 |
| 1455150_at | HECT, C2 and WW domain containing E3 ubiquitin protein ligase 2 | *Hecw2* | 2.00 | 0.56 |
| 1417073_a_at | quaking | *Qk* | 2.93 | 0.55 |
| 1455335_at | X-ray repair complementing defective repair in Chinese hamster cells 2 | *Xrcc2* | 1.75 | 0.55 |
| 1449360_at | colony stimulating factor 2 receptor, beta 2, low-affinity (granulocyte-macrophage) | *Csf2rb2* | 2.10 | 0.55 |
| 1437303_at | interleukin 6 signal transducer | *Il6st* | 1.85 | 0.55 |
| 1429313_at | receptor tyrosine kinase-like orphan receptor 1 | *Ror1* | 1.95 | 0.55 |
| 1423635_at | bone morphogenetic protein 2 | *Bmp2* | 1.57 | 0.55 |
| 1420410_at | nuclear receptor subfamily 5, group A, member 2 | *Nr5a2* | 1.42 | 0.55 |
| 1436293_x_at | immunoglobulin-like domain containing receptor 2 | *Ildr2* | 4.41 | 0.55 |
| 1421186_at | chemokine (C-C motif) receptor 2 | *Ccr2* | 2.58 | 0.55 |
| 1460271_at | triggering receptor expressed on myeloid cells 3 | *Trem3* | 1.89 | 0.55 |
| 1434020_at | PDGFA associated protein 1 | *Pdap1* | 1.40 | 0.55 |
| 1422535_at | cyclin E2 | *Ccne2* | 2.46 | 0.55 |
| 1457632_s_at | Meis homeobox 2 | *Meis2* | 2.57 | 0.55 |
| 1453238_s_at | carbohydrate sulfotransferase 11 | *Chst11* | 1.83 | 0.55 |
| 1422810_at | zinc finger protein 191 | *Zfp191* | 1.23 | 0.55 |
| 1451674_at | solute carrier family 12, member 5 | *Slc12a5* | 1.26 | 0.55 |
| 1456791_at | zinc finger protein 800 | *Zfp800* | 1.74 | 0.55 |
| 1439766_x_at | vascular endothelial growth factor C | *Vegfc* | 3.40 | 0.55 |
| 1418285_at | ephrin B1 | *Efnb1* | 1.29 | 0.55 |
| 1438803_s_at | sorting nexin 16 | *Snx16* | 2.31 | 0.55 |
| 1438419_at | RNA binding motif protein 16 | *Rbm16* | 1.29 | 0.55 |
| 1424658_at | TAO kinase 1 | *Taok1* | 2.45 | 0.55 |
| 1416081_at | MAD homolog 1 (Drosophila) | *Smad1* | 1.33 | 0.55 |
| 1437604_x_at | adenomatosis polyposis coli down-regulated 1 | *Apcdd1* | 1.38 | 0.55 |
| 1453260_a_at | protein phosphatase 2 (formerly 2A), regulatory subunit B (PR 52), alpha isoform | *Ppp2r2a* | 1.30 | 0.55 |
| 1452817_at | SET and MYND domain containing 3 | *Smyd3* | 1.76 | 0.55 |
| 1418431_at | kinesin family member 5B | *Kif5b* | 1.67 | 0.55 |
| 1425775_at | zinc finger protein 820 | *Zfp820* | 3.33 | 0.55 |
| 1427276_at | structural maintenance of chromosomes 4 | *Smc4* | 1.49 | 0.55 |
| 1447520_at | lipopolysaccharide binding protein | *Lbp* | 2.52 | 0.54 |
| 1455164_at | Rho GTPase activating protein 31 | *Arhgap31* | 1.64 | 0.54 |
| 1434310_at | bone morphogenic protein receptor, type II (serine/threonine kinase) | *Bmpr2* | 1.93 | 0.54 |
| 1431939_a_at | myc induced nuclear antigen | *Mina* | 1.97 | 0.54 |
| 1427186_a_at | myocyte enhancer factor 2A | *Mef2a* | 1.55 | 0.54 |
| 1449121_at | serine/arginine-rich splicing factor 10 | *Srsf10* | 2.18 | 0.54 |
| 1434509_at | Rap guanine nucleotide exchange factor (GEF) 6 | *Rapgef6* | 1.57 | 0.54 |
| 1419381_at | telomeric repeat binding factor 2, interacting protein | *Terf2ip* | 1.34 | 0.54 |
| 1435462_at | phosphatidylinositol-specific phospholipase C, X domain containing 2 | *Plcxd2* | 1.54 | 0.54 |
| 1418545_at | WASP family 1 | *Wasf1* | 1.21 | 0.54 |
| 1451530_at | epidermal growth factor receptor | *Egfr* | 1.44 | 0.54 |
| 1438556_a_at | tropomodulin 3 | *Tmod3* | 2.09 | 0.54 |
| 1451982_at | mitogen-activated protein kinase kinase 4 | *Map2k4* | 1.36 | 0.54 |
| 1453288_at | activating transcription factor 6 | *Atf6* | 1.57 | 0.54 |
| 1424207_at | SWI/SNF related, matrix associated, actin dependent regulator of chromatin, subfamily a, member 5 | *Smarca5* | 1.37 | 0.54 |
| 1455488_at | HAUS augmin-like complex, subunit 6 | *Haus6* | 1.52 | 0.54 |
| 1426063_a_at | GTP binding protein (gene overexpressed in skeletal muscle) | *Gem* | 3.46 | 0.54 |
| 1421163_a_at | nuclear factor I/A | *Nfia* | 1.41 | 0.54 |
| 1422556_at | guanine nucleotide binding protein, alpha 13 | *Gna13* | 1.25 | 0.54 |
| 1448780_at | solute carrier family 12, member 2 | *Slc12a2* | 1.65 | 0.54 |
| 1433784_at | zinc finger protein 871 | *Zfp871* | 1.45 | 0.54 |
| 1458385_at | heat shock protein 4 like | *Hspa4l* | 1.90 | 0.53 |
| 1439697_at | interleukin 1 receptor accessory protein | *Il1rap* | 1.65 | 0.53 |
| 1439515_at | SET domain containing 5 | *Setd5* | 1.81 | 0.53 |
| 1458943_at | methylthioadenosine phosphorylase | *Mtap* | 3.10 | 0.53 |
| 1437953_at | glycerophosphocholine phosphodiesterase GDE1 homolog (S. cerevisiae) | *Gpcpd1* | 1.34 | 0.53 |
| 1422528_a_at | zinc finger protein 36, C3H type-like 1 | *Zfp36l1* | 1.30 | 0.53 |
| 1449292_at | RB1-inducible coiled-coil 1 | *Rb1cc1* | 1.50 | 0.53 |
| 1429655_at | NudC domain containing 1 | *Nudcd1* | 1.25 | 0.53 |
| 1446737_a_at | hook homolog 3 (Drosophila) | *Hook3* | 1.34 | 0.53 |
| 1418280_at | Kruppel-like factor 6 | *Klf6* | 2.15 | 0.53 |
| 1453137_at | F-box protein 30 | *Fbxo30* | 1.79 | 0.53 |
| 1424031_at | sorting nexin 11 | *Snx11* | 1.38 | 0.53 |
| 1435793_at | anterior pharynx defective 1b homolog (C. elegans) | *Aph1b* | 2.97 | 0.53 |
| 1419394_s_at | lactotransferrin | *Ltf* | 39.13 | 0.53 |
| 1434917_at | cordon-bleu | *Cobl* | 1.73 | 0.53 |
| 1439033_at | zinc finger, CCHC domain containing 7 | *Zcchc7* | 1.81 | 0.53 |
| 1454959_s_at | guanine nucleotide binding protein (G protein), alpha inhibiting 1 | *Gnai1* | 1.36 | 0.53 |
| 1424012_at | tetratricopeptide repeat domain 30A1 | *Ttc30a1* | 1.29 | 0.53 |
| 1422595_s_at | suppressor of IKBKE 1 | *Sike1* | 1.69 | 0.53 |
| 1440152_x_at | endothelial differentiation-related factor 1 | *Edf1* | 1.81 | 0.53 |
| 1452137_at | acyl-Coenzyme A binding domain containing 3 | *Acbd3* | 1.26 | 0.53 |
| 1441214_at | exophilin 5 | *Exph5* | 2.22 | 0.53 |
| 1444395_at | DIX domain containing 1 | *Dixdc1* | 1.22 | 0.52 |
| 1443620_at | Glypican 4 | *Gpc4* | 1.90 | 0.52 |
| 1452193_a_at | Wiskott-Aldrich syndrome-like (human) | *Wasl* | 1.59 | 0.52 |
| 1436514_at | glypican 4 | *Gpc4* | 1.45 | 0.52 |
| 1418264_at | centromere protein K | *Cenpk* | 3.02 | 0.52 |
| 1453578_at | phosphotriesterase related | *Pter* | 4.41 | 0.52 |
| 1456726_x_at | glutaminyl-tRNA synthetase | *Qars* | 1.26 | 0.52 |
| 1455179_at | membrane protein, palmitoylated 7 (MAGUK p55 subfamily member 7) | *Mpp7* | 1.29 | 0.52 |
| 1425973_at | lysosomal trafficking regulator | *Lyst* | 1.77 | 0.52 |
| 1454665_at | interferon regulatory factor 2 binding protein 2 | *Irf2bp2* | 2.16 | 0.52 |
| 1428974_s_at | leucine zipper transcription factor-like 1 | *Lztfl1* | 2.46 | 0.52 |
| 1419459_a_at | magnesium transporter 1 | *Magt1* | 1.37 | 0.52 |
| 1429371_at | zinc finger protein 788 | *Zfp788* | 1.48 | 0.52 |
| 1438941_x_at | adenosine monophosphate deaminase 2 | *Ampd2* | 1.90 | 0.52 |
| 1435811_a_at | unc-50 homolog (C. elegans) | *Unc50* | 4.68 | 0.52 |
| 1425745_a_at | transforming, acidic coiled-coil containing protein 2 | *Tacc2* | 2.16 | 0.52 |
| 1419864_x_at | transportin 1 | *Tnpo1* | 1.28 | 0.52 |
| 1426301_at | activated leukocyte cell adhesion molecule | *Alcam* | 2.20 | 0.52 |
| 1446939_at | tripartite motif-containing 12A | *Trim12a* | 1.21 | 0.52 |
| 1455593_at | apolipoprotein B | *Apob* | 1.48 | 0.52 |
| 1437638_at | serine/arginine repetitive matrix 2 | *Srrm2* | 1.98 | 0.52 |
| 1452074_at | transmembrane protein 135 | *Tmem135* | 1.26 | 0.52 |
| 1435632_at | nuclear fragile X mental retardation protein interacting protein 2 | *Nufip2* | 1.75 | 0.52 |
| 1449858_at | CD86 antigen | *Cd86* | 2.53 | 0.52 |
| 1436155_at | nicotinamide nucleotide adenylyltransferase 2 | *Nmnat2* | 1.20 | 0.52 |
| 1449824_at | proteoglycan 4 (megakaryocyte stimulating factor, articular superficial zone protein) | *Prg4* | 6.58 | 0.52 |
| 1437154_at | centrosomal protein 170 | *Cep170* | 3.26 | 0.52 |
| 1427117_at | myotubularin related protein 3 | *Mtmr3* | 1.91 | 0.52 |
| 1451730_at | zinc finger protein 62 | *Zfp62* | 1.61 | 0.51 |
| 1434446_at | insulin receptor | *Insr* | 1.61 | 0.51 |
| 1443860_at | protein tyrosine phosphatase, receptor type, D | *Ptprd* | 1.47 | 0.51 |
| 1434405_at | folliculin interacting protein 1 | *Fnip1* | 1.65 | 0.51 |
| 1443798_at | phosphatidylinositol 3-kinase catalytic delta polypeptide | *Pik3cd* | 1.28 | 0.51 |
| 1443665_at | DENN/MADD domain containing 5B | *Dennd5b* | 1.35 | 0.51 |
| 1425663_at | interleukin 1 receptor antagonist | *Il1rn* | 2.50 | 0.51 |
| 1435051_at | WD repeat domain 44 | *Wdr44* | 2.11 | 0.51 |
| 1439908_at | zinc finger with KRAB and SCAN domains 1 | *Zkscan1* | 1.67 | 0.51 |
| 1418529_at | O-sialoglycoprotein endopeptidase | *Osgep* | 1.46 | 0.51 |
| 1454823_at | WD repeat domain 37 | *Wdr37* | 2.25 | 0.51 |
| 1434704_at | myeloid/lymphoid or mixed-lineage leukemia 5 | *Mll5* | 2.00 | 0.51 |
| 1435946_at | Sep (O-phosphoserine) tRNA:Sec (selenocysteine) tRNA synthase | *Sepsecs* | 1.29 | 0.51 |
| 1453012_at | TSC22 domain family, member 2 | *Tsc22d2* | 1.52 | 0.51 |
| 1426677_at | filamin, alpha | *Flna* | 1.35 | 0.51 |
| 1453684_s_at | zinc finger CCCH-type containing 15 | *Zc3h15* | 1.63 | 0.51 |
| 1417116_at | solute carrier family 6 (neurotransmitter transporter, creatine), member 8 | *Slc6a8* | 2.26 | 0.50 |
| 1437512_x_at | EBNA1 binding protein 2 | *Ebna1bp2* | 1.57 | 0.50 |
| 1451752_at | forkhead box K1 | *Foxk1* | 1.34 | 0.50 |
| 1442029_at | KCNQ1 overlapping transcript 1 | *Kcnq1ot1* | 1.59 | 0.50 |
| 1458515_at | zinc finger protein 128 | *Zfp128* | 1.22 | 0.50 |
| 1459838_s_at | BTB (POZ) domain containing 11 | *Btbd11* | 1.34 | 0.50 |
| 1429769_at | protein geranylgeranyltransferase type I, beta subunit | *Pggt1b* | 1.40 | 0.50 |
| 1438953_at | c-fos induced growth factor | *Figf* | 1.28 | 0.50 |
| 1437158_at | Nipped-B homolog (Drosophila) | *Nipbl* | 1.47 | 0.50 |
| 1428194_at | ubiquitin specific peptidase 9, X chromosome | *Usp9x* | 1.24 | 0.50 |
| 1427290_at | keratin 81 | *Krt81* | 1.43 | 0.50 |
| 1452761_a_at | RNA binding motif, single stranded interacting protein | *Rbms3* | 1.34 | 0.50 |
| 1431578_at | WBP2 N-terminal like | *Wbp2nl* | 1.39 | 0.50 |
| 1435741_at | phosphodiesterase 8B | *Pde8b* | 2.46 | 0.50 |
| 1438432_at | leucyl/cystinyl aminopeptidase | *Lnpep* | 1.28 | 0.50 |
| 1450038_s_at | ubiquitin specific peptidase 9, X chromosome | *Usp9x* | 1.45 | 0.50 |
| 1437743_at | AE binding protein 2 | *Aebp2* | 1.70 | 0.50 |
| 1452366_at | chondroitin sulfate N-acetylgalactosaminyltransferase 1 | *Csgalnact1* | 1.35 | 0.50 |
| 1449423_at | microtubule associated serine/threonine kinase 1 | *Mast1* | 1.43 | 0.50 |
| 1453596_at | inhibitor of DNA binding 2 | *Id2* | 1.30 | 0.50 |
| 1452077_at | DEAD (Asp-Glu-Ala-Asp) box polypeptide 3, Y-linked | *Ddx3y* | 1.62 | 0.50 |
| 1434644_at | transducin (beta)-like 1 X-linked | *Tbl1x* | 1.72 | 0.49 |
| 1427467_a_at | retinitis pigmentosa GTPase regulator | *Rpgr* | 2.50 | 0.49 |
| 1452011_a_at | UDP-glucuronate decarboxylase 1 | *Uxs1* | 2.30 | 0.49 |
| 1443773_at | YLP motif containing 1 | *Ylpm1* | 2.22 | 0.49 |
| 1425320_at | zinc finger protein 605 | *Zfp605* | 1.99 | 0.49 |
| 1434668_at | nuclear casein kinase and cyclin-dependent kinase substrate 1 | *Nucks1* | 1.69 | 0.49 |
| 1452783_at | fibronectin type III domain containing 3B | *Fndc3b* | 3.22 | 0.49 |
| 1437105_at | lysine (K)-specific demethylase 5A | *Kdm5a* | 1.62 | 0.49 |
| 1416147_at | heat shock protein 4 | *Hspa4* | 1.50 | 0.49 |
| 1424188_at | RAB GTPase activating protein 1 | *Rabgap1* | 1.67 | 0.49 |
| 1437101_at | large tumor suppressor 2 | *Lats2* | 1.45 | 0.49 |
| 1422643_at | monooxygenase, DBH-like 1 | *Moxd1* | 1.42 | 0.49 |
| 1435224_at | CREB binding protein | *Crebbp* | 1.48 | 0.49 |
| 1419563_at | baculoviral IAP repeat-containing 6 | *Birc6* | 1.30 | 0.49 |
| 1455634_at | Son DNA binding protein | *Son* | 2.52 | 0.49 |
| 1427253_s_at | suppressor of zeste 12 homolog (Drosophila) | *Suz12* | 1.96 | 0.49 |
| 1427456_at | WD repeat and FYVE domain containing 3 | *Wdfy3* | 1.88 | 0.49 |
| 1442038_at | RNA binding motif protein 26 | *Rbm26* | 1.48 | 0.49 |
| 1442368_at | potassium channel tetramerisation domain containing 12b | *Kctd12b* | 2.70 | 0.49 |
| 1420852_a_at | UDP-GlcNAc:betaGal beta-1,3-N-acetylglucosaminyltransferase 2 | *B3gnt2* | 1.26 | 0.49 |
| 1458518_at | cytoplasmic polyadenylation element binding protein 2 | *Cpeb2* | 1.27 | 0.49 |
| 1429464_at | protein kinase, AMP-activated, alpha 2 catalytic subunit | *Prkaa2* | 1.32 | 0.49 |
| 1426068_at | solute carrier family 7 (cationic amino acid transporter, y+ system), member 4 | *Slc7a4* | 1.52 | 0.48 |
| 1422748_at | zinc finger E-box binding homeobox 2 | *Zeb2* | 1.32 | 0.48 |
| 1450291_s_at | membrane-spanning 4-domains, subfamily A, member 4C | *Ms4a4c* | 1.64 | 0.48 |
| 1437304_at | Casitas B-lineage lymphoma b | *Cblb* | 1.78 | 0.48 |
| 1434096_at | solute carrier family 4 (anion exchanger), member 4 | *Slc4a4* | 2.48 | 0.48 |
| 1455836_at | poly (A) polymerase alpha | *Papola* | 1.32 | 0.48 |
| 1451526_at | Rho GTPase activating protein 12 | *Arhgap12* | 2.10 | 0.48 |
| 1417069_a_at | glia maturation factor, beta | *Gmfb* | 1.36 | 0.48 |
| 1436303_at | myeloid/lymphoid or mixed-lineage leukemia (trithorax homolog, Drosophila); translocated to, 4 | *Mllt4* | 2.72 | 0.48 |
| 1435936_at | solute carrier family 13 (sodium-dependent citrate transporter), member 5 | *Slc13a5* | 2.44 | 0.48 |
| 1436533_at | TROVE domain family, member 2 | *Trove2* | 2.17 | 0.48 |
| 1447875_x_at | zinc finger protein 800 | *Zfp800* | 1.93 | 0.48 |
| 1428936_at | ATPase, Ca++ transporting, plasma membrane 1 | *Atp2b1* | 1.35 | 0.48 |
| 1429537_at | serine/arginine-rich splicing factor 18 | *Sfrs18* | 1.60 | 0.48 |
| 1440787_s_at | Bardet-Biedl syndrome 10 (human) | *Bbs10* | 1.96 | 0.48 |
| 1417851_at | haptoglobin | *Hp* | 6.71 | 0.48 |
| 1437204_a_at | transcription factor 25 (basic helix-loop-helix) | *Tcf25* | 1.23 | 0.48 |
| 1460197_a_at | STEAP family member 4 | *Steap4* | 9.95 | 0.47 |
| 1421275_s_at | suppressor of cytokine signaling 4 | *Socs4* | 2.05 | 0.47 |
| 1424850_at | mitogen-activated protein kinase kinase kinase 1 | *Map3k1* | 2.09 | 0.47 |
| 1436300_at | dual serine/threonine and tyrosine protein kinase | *Dstyk* | 1.36 | 0.47 |
| 1449122_at | UBX domain protein 2B | *Ubxn2b* | 2.16 | 0.47 |
| 1434178_at | myeloid/lymphoid or mixed-lineage leukemia 3 | *Mll3* | 1.68 | 0.47 |
| 1424468_s_at | pleckstrin homology-like domain, family B, member 1 | *Phldb1* | 1.61 | 0.47 |
| 1440343_at | ribosomal protein S6 kinase, polypeptide 5 | *Rps6ka5* | 1.34 | 0.47 |
| 1436746_at | WNK lysine deficient protein kinase 1 | *Wnk1* | 1.45 | 0.47 |
| 1443396_at | survival motor neuron domain containing 1 | *Smndc1* | 1.49 | 0.47 |
| 1448756_at | transferrin receptor | *Tfrc* | 21.69 | 0.47 |
| 1438976_x_at | methionine adenosyltransferase II, alpha | *Mat2a* | 2.30 | 0.47 |
| 1460509_at | UDP-Gal:betaGlcNAc beta 1,3-galactosyltransferase, polypeptide 1 | *B3galt1* | 1.43 | 0.47 |
| 1453023_at | ankyrin repeat and KH domain containing 1 | *Ankhd1* | 1.91 | 0.47 |
| 1418674_at | oncostatin M receptor | *Osmr* | 7.07 | 0.46 |
| 1436984_at | abl-interactor 2 | *Abi2* | 2.56 | 0.46 |
| 1424484_at | MOB1, Mps One Binder kinase activator-like 1B (yeast) | *Mobkl1b* | 1.94 | 0.46 |
| 1426371_at | fatty acyl CoA reductase 1 | *Far1* | 2.28 | 0.46 |
| 1420959_at | aspartate-beta-hydroxylase | *Asph* | 2.95 | 0.46 |
| 1426532_at | zinc finger, MYND domain containing 11 | *Zmynd11* | 1.63 | 0.46 |
| 1419648_at | myosin IC | *Myo1c* | 1.82 | 0.46 |
| 1452331_s_at | glutamine and serine rich 1 | *Qser1* | 1.27 | 0.46 |
| 1425829_a_at | STEAP family member 4 | *Steap4* | 4.53 | 0.46 |
| 1456731_x_at | polymerase (RNA) III (DNA directed) polypeptide K | *Polr3k* | 1.28 | 0.46 |
| 1442827_at | toll-like receptor 4 | *Tlr4* | 1.84 | 0.46 |
| 1418024_at | N(alpha)-acetyltransferase 15, NatA auxiliary subunit | *Naa15* | 1.65 | 0.45 |
| 1450479_x_at | protein tyrosine phosphatase, non-receptor type 12 | *Ptpn12* | 1.99 | 0.45 |
| 1453581_at | centrosomal protein 170 | *Cep170* | 1.36 | 0.45 |
| 1417792_at | zinc finger, matrin-like | *Zfml* | 3.14 | 0.45 |
| 1419655_at | transducin-like enhancer of split 3, homolog of Drosophila E(spl) | *Tle3* | 1.59 | 0.45 |
| 1449661_at | Suppressor of zeste 12 homolog (Drosophila) | *Suz12* | 1.88 | 0.45 |
| 1445824_at | zinc finger protein 458 | *Zfp458* | 1.51 | 0.45 |
| 1438040_a_at | heat shock protein 90, beta (Grp94), member 1 | *Hsp90b1* | 1.91 | 0.45 |
| 1420438_at | orosomucoid 2 | *Orm2* | 99.06 | 0.45 |
| 1448508_at | TRAF3 interacting protein 2 | *Traf3ip2* | 1.79 | 0.45 |
| 1441396_at | UDP-Gal:betaGlcNAc beta 1,3-galactosyltransferase, polypeptide 1 | *B3galt1* | 4.18 | 0.44 |
| 1423750_a_at | splicing factor 1 | *Sf1* | 1.39 | 0.44 |
| 1450101_a_at | membrane associated guanylate kinase, WW and PDZ domain containing 3 | *Magi3* | 1.67 | 0.44 |
| 1428867_at | exocyst complex component 3-like 2 | *Exoc3l2* | 3.48 | 0.44 |
| 1457264_at | PHD finger protein 20-like 1 | *Phf20l1* | 1.71 | 0.44 |
| 1450083_at | CCR4-NOT transcription complex, subunit 4 | *Cnot4* | 1.69 | 0.44 |
| 1419100_at | leucine-rich alpha-2-glycoprotein 1 | *Lrg1* | 8.85 | 0.44 |
| 1437894_at | prospero-related homeobox 1 | *Prox1* | 1.94 | 0.44 |
| 1438719_at | mitogen-activated protein kinase kinase kinase 2 | *Map3k2* | 1.45 | 0.44 |
| 1416846_a_at | PDZ domain containing RING finger 3 | *Pdzrn3* | 2.22 | 0.44 |
| 1447543_at | WD repeat and FYVE domain containing 1 | *Wdfy1* | 1.68 | 0.44 |
| 1425206_a_at | Ubiquitin protein ligase E3A | *Ube3a* | 1.21 | 0.44 |
| 1460567_at | regulatory factor X, 7 | *Rfx7* | 1.82 | 0.44 |
| 1446483_at | aldo-keto reductase family 1, member E1 | *Akr1e1* | 1.77 | 0.44 |
| 1456712_at | ligand dependent nuclear receptor corepressor-like | *Lcorl* | 1.52 | 0.43 |
| 1422206_at | UDP-Gal:betaGlcNAc beta 1,3-galactosyltransferase, polypeptide 1 | *B3galt1* | 2.22 | 0.43 |
| 1435474_at | TAF5 RNA polymerase II, TATA box binding protein (TBP)-associated factor | *Taf5* | 1.26 | 0.43 |
| 1429410_at | enhancer of yellow 2 homolog (Drosophila) | *Eny2* | 1.48 | 0.43 |
| 1451038_at | apelin | *Apln* | 2.77 | 0.43 |
| 1427747_a_at | phospholipase A2, group VII (platelet-activating factor acetylhydrolase, plasma) | *Pla2g7* | 249.95 | 0.43 |
| 1420908_at | CD2-associated protein | *Cd2ap* | 1.95 | 0.43 |
| 1418824_at | ADP-ribosylation factor 6 | *Arf6* | 1.47 | 0.43 |
| 1434643_at | transducin (beta)-like 1 X-linked | *Tbl1x* | 1.32 | 0.43 |
| 1456951_at | myeloblastosis oncogene-like 1 | *Mybl1* | 4.01 | 0.43 |
| 1448160_at | lymphocyte cytosolic protein 1 | *Lcp1* | 1.55 | 0.43 |
| 1433210_at | repetin | *Rptn* | 1.29 | 0.43 |
| 1434751_at | iduronate 2-sulfatase | *Ids* | 1.42 | 0.43 |
| 1422868_s_at | guanine deaminase | *Gda* | 1.58 | 0.43 |
| 1437206_at | SET domain containing 5 | *Setd5* | 1.20 | 0.43 |
| 1435663_at | estrogen receptor 1 (alpha) | *Esr1* | 1.81 | 0.42 |
| 1430981_s_at | GC-rich promoter binding protein 1 | *Gpbp1* | 2.81 | 0.42 |
| 1417139_at | DSN1, MIND kinetochore complex component, homolog (S. cerevisiae) | *Dsn1* | 1.46 | 0.42 |
| 1434856_at | ankyrin repeat domain 44 | *Ankrd44* | 1.38 | 0.42 |
| 1452071_at | solute carrier family 4 (anion exchanger), member 4 | *Slc4a4* | 1.82 | 0.42 |
| 1437884_at | ADP-ribosylation factor-like 5B | *Arl5b* | 1.53 | 0.42 |
| 1427037_at | eukaryotic translation initiation factor 4, gamma 1 | *Eif4g1* | 1.44 | 0.42 |
| 1415988_at | high density lipoprotein (HDL) binding protein | *Hdlbp* | 1.40 | 0.42 |
| 1416190_a_at | Sec61 alpha 1 subunit (S. cerevisiae) | *Sec61a1* | 1.72 | 0.42 |
| 1415989_at | vascular cell adhesion molecule 1 | *Vcam1* | 2.31 | 0.42 |
| 1418023_at | N(alpha)-acetyltransferase 15, NatA auxiliary subunit | *Naa15* | 1.87 | 0.41 |
| 1439241_x_at | steroid 5 alpha-reductase 3 | *Srd5a3* | 5.99 | 0.41 |
| 1433804_at | Janus kinase 1 | *Jak1* | 1.45 | 0.41 |
| 1426326_at | zinc finger protein 91 | *Zfp91* | 1.88 | 0.41 |
| 1435521_at | Musashi homolog 2 (Drosophila) | *Msi2* | 1.86 | 0.41 |
| 1424768_at | caldesmon 1 | *Cald1* | 1.54 | 0.41 |
| 1443485_at | Eph receptor A7 | *Epha7* | 1.43 | 0.41 |
| 1419470_at | guanine nucleotide binding protein (G protein), beta 4 | *Gnb4* | 1.58 | 0.41 |
| 1455387_at | nuclear fragile X mental retardation protein interacting protein 2 | *Nufip2* | 1.37 | 0.40 |
| 1420570_x_at | T-cell leukemia/lymphoma 1B, 3 | *Tcl1b3* | 1.36 | 0.40 |
| 1450530_at | UDP-Gal:betaGlcNAc beta 1,3-galactosyltransferase, polypeptide 1 | *B3galt1* | 2.82 | 0.40 |
| 1434765_at | E1A binding protein p300 | *Ep300* | 2.26 | 0.40 |
| 1449971_a_at | CD209f antigen | *Cd209f* | 1.49 | 0.40 |
| 1418018_at | carboxypeptidase D | *Cpd* | 1.92 | 0.40 |
| 1421251_at | zinc finger protein 40 | *Zfp40* | 4.30 | 0.39 |
| 1433833_at | fibronectin type III domain containing 3B | *Fndc3b* | 6.12 | 0.39 |
| 1416200_at | interleukin 6 | *Il6* | 3.03 | 0.39 |
| 1439077_at | zinc finger, X-linked, duplicated A | *Zxda* | 1.75 | 0.39 |
| 1424598_at | DEAD (Asp-Glu-Ala-Asp) box polypeptide 6 | *Ddx6* | 1.21 | 0.39 |
| 1423417_at | SWI/SNF related, matrix associated, actin dependent regulator of chromatin, subfamily c, member 1 | *Smarcc1* | 1.26 | 0.39 |
| 1435168_at | zinc finger protein 710 | *Zfp710* | 1.37 | 0.39 |
| 1422237_at | melanocortin 3 receptor | *Mc3r* | 1.43 | 0.39 |
| 1449615_s_at | high density lipoprotein (HDL) binding protein | *Hdlbp* | 1.41 | 0.38 |
| 1434208_at | ring finger protein 169 | *Rnf169* | 1.26 | 0.38 |
| 1450035_a_at | PRP40 pre-mRNA processing factor 40 homolog A (yeast) | *Prpf40a* | 1.24 | 0.38 |
| 1427408_a_at | thyroid hormone receptor associated protein 3 | *Thrap3* | 1.39 | 0.38 |
| 1420957_at | adenomatosis polyposis coli | *Apc* | 2.35 | 0.38 |
| 1431098_at | CAP-GLY domain containing linker protein 1 | *Clip1* | 1.31 | 0.38 |
| 1439942_at | prolyl endopeptidase | *Prep* | 1.79 | 0.37 |
| 1428941_at | zinc finger, MYM-type 2 | *Zmym2* | 1.44 | 0.37 |
| 1437071_at | eukaryotic translation initiation factor 1A, X-linked | *Eif1ax* | 1.27 | 0.37 |
| 1437102_at | YTH domain family 1 | *Ythdf1* | 1.62 | 0.37 |
| 1460597_at | additional sex combs like 2 (Drosophila) | *Asxl2* | 1.99 | 0.37 |
| 1424350_s_at | lysophosphatidylglycerol acyltransferase 1 | *Lpgat1* | 2.08 | 0.37 |
| 1457292_at | TATA box binding protein (Tbp)-associated factor, RNA polymerase I, D | *Taf1d* | 1.54 | 0.36 |
| 1437177_at | La ribonucleoprotein domain family, member 4 | *Larp4* | 1.70 | 0.36 |
| 1450113_at | membrane protein, palmitoylated 5 (MAGUK p55 subfamily member 5) | *Mpp5* | 1.94 | 0.36 |
| 1434554_at | tripartite motif-containing 37 | *Trim37* | 1.46 | 0.36 |
| 1424325_at | establishment of cohesion 1 homolog 1 (S. cerevisiae) | *Esco1* | 1.36 | 0.36 |
| 1435163_at | zinc finger protein 871 | *Zfp871* | 1.49 | 0.36 |
| 1453270_a_at | PHD finger protein 14 | *Phf14* | 1.38 | 0.36 |
| 1430834_at | GPRIN family member 3 | *Gprin3* | 1.36 | 0.36 |
| 1436221_at | immunoglobulin-like domain containing receptor 2 | *Ildr2* | 8.28 | 0.36 |
| 1417736_at | structural maintenance of chromosomes 6 | *Smc6* | 1.50 | 0.36 |
| 1423184_at | intersectin 2 | *Itsn2* | 1.49 | 0.36 |
| 1455171_at | suppressor of variegation 4-20 homolog 1 (Drosophila) | *Suv420h1* | 1.33 | 0.35 |
| 1416484_at | tetratricopeptide repeat domain 3 | *Ttc3* | 1.30 | 0.35 |
| 1417268_at | CD14 antigen | *Cd14* | 7.10 | 0.35 |
| 1440146_at | vacuolar protein sorting 13A (yeast) | *Vps13a* | 1.45 | 0.35 |
| 1421055_at | large tumor suppressor 2 | *Lats2* | 1.25 | 0.34 |
| 1417084_at | eukaryotic translation initiation factor 4E binding protein 2 | *Eif4ebp2* | 1.78 | 0.34 |
| 1456088_at | X-linked inhibitor of apoptosis | *Xiap* | 1.72 | 0.34 |
| 1434911_s_at | Rho GTPase activating protein 19 | *Arhgap19* | 2.22 | 0.33 |
| 1448859_at | signal-regulatory protein beta 1A | *Sirpb1a* | 8.45 | 0.33 |
| 1455960_at | multiple EGF-like-domains 9 | *Megf9* | 2.35 | 0.33 |
| 1419587_s_at | retinitis pigmentosa 2 homolog (human) | *Rp2h* | 3.15 | 0.33 |
| 1421425_a_at | regulator of calcineurin 2 | *Rcan2* | 1.32 | 0.32 |
| 1457554_at | apolipoprotein B | *Apob* | 1.88 | 0.32 |
| 1434248_at | protein kinase C, eta | *Prkch* | 2.40 | 0.32 |
| 1417496_at | chemokine (C-X-C motif) ligand 13 | *Cxcl13* | 4.91 | 0.31 |
| 1420946_at | alpha thalassemia/mental retardation syndrome X-linked homolog (human) | *Atrx* | 2.02 | 0.29 |
| 1459864_at | G protein-coupled receptor 146 | *Gpr146* | 1.57 | 0.28 |
| 1443375_at | GNAS (guanine nucleotide binding protein, alpha stimulating) complex locus | *Gnas* | 1.51 | 0.27 |
| 1443017_at | cytoplasmic polyadenylation element binding protein 2 | *Cpeb2* | 2.04 | 0.26 |
| 1450703_at | solute carrier family 7 (cationic amino acid transporter, y+ system), member 2 | *Slc7a2* | 1.66 | 0.25 |
| 1424856_at | ATPase, Na+/K+ transporting, alpha 3 polypeptide | *Atp1a3* | 3.56 | 0.25 |
| 1434069_at | phosphatidylinositol-3,4,5-trisphosphate-dependent Rac exchange factor 1 | *Prex1* | 2.20 | 0.24 |
| 1457262_at | SMG1 homolog, phosphatidylinositol 3-kinase-related kinase (C. elegans) | *Smg1* | 1.65 | 0.21 |
| 1452378_at | metastasis associated lung adenocarcinoma transcript 1 (non-coding RNA) | *Malat1* | 1.28 | 0.17 |
| 1439122_at | DEAD (Asp-Glu-Ala-Asp) box polypeptide 6 | *Ddx6* | 5.93 | 0.17 |
| 1435039_a_at | phosphatidylinositol-4-phosphate 5-kinase, type 1 alpha | *Pip5k1a* | 1.57 | 0.16 |
| 1450826_a_at | proviral integration site 1 | *Pim1* | 32.95 | 0.13 |
