# Supplementary material for: Integrated omics profiling of dextran sodium sulfate-induced colitic mice supplemented with Wolfberry (Lycium barbarum)
Source: NPJ Sci Food. 2020 Mar 31;4:5. doi: 10.1038/s41538-020-0065-5 (PMC7109062; doi:10.1038/s41538-020-0065-5)
Supplement: Supplementary file 4 — Supplementary Table 3 Differentially regulated proteins in liver [file 41538_2020_65_MOESM4_ESM.docx]

Supplementary Table 3 Differentially expressed proteins in liver

75 up-regulated proteins in DSSWOL as compared to DSS

|  |  |  | Fold Change | Fold Change |
| --- | --- | --- | --- | --- |
| Accession Number | Name | Gene Symbol | DSS vs. CON | DSSWOL vs. DSS |
| P05977 | Myosin light chain 1/3, skeletal muscle isoform | *Myl1* | 0.2 | 9.1 |
| P09542 | Myosin light chain 3 | *Myl3* | 0.7 | 7.4 |
| P21550 | Beta-enolase | *Eno3* | 0.4 | 5.7 |
| P07309 | Transthyretin | *Ttr* | 0.5 | 2.1 |
| P31786 | Acyl-CoA-binding protein | *Dbi* | 0.5 | 2.1 |
| O35215 | D-dopachrome decarboxylase | *Ddt* | 0.5 | 2.0 |
| P08228 | Superoxide dismutase [Cu-Zn] | *Sod1* | 0.6 | 1.9 |
| Q9QXD6 | Fructose-1,6-bisphosphatase 1 | *Fbp1* | 0.6 | 1.9 |
| Q8BK30 | NADH dehydrogenase [ubiquinone] flavoprotein 3, mitochondrial | *Ndufv3* | 0.5 | 1.8 |
| P56212 | cAMP-regulated phosphoprotein 19 | *Arpp19* | 0.6 | 1.8 |
| P12787 | Cytochrome c oxidase subunit 5A, mitochondrial | *Cox5a* | 0.5 | 1.8 |
| Q9CR21 | Acyl carrier protein, mitochondrial | *Ndufab1* | 0.6 | 1.7 |
| P46938 | Yorkie homolog | *Yap1* | 0.7 | 1.7 |
| P33622 | Apolipoprotein C-III | *Apoc3* | 0.6 | 1.7 |
| P61458 | Pterin-4-alpha-carbinolamine dehydratase | *Pcbd1* | 0.6 | 1.7 |
| Q9DCU9 | Dihydrodipicolinate synthase-like, mitochondrial | *Dhdpsl* | 0.6 | 1.7 |
| Q9CPU0 | Lactoylglutathione lyase | *Glo1* | 0.6 | 1.6 |
| Q62426 | Cystatin-B | *Cstb* | 0.8 | 1.6 |
| O08997 | Copper transport protein ATOX1 | *Atox1* | 0.7 | 1.6 |
| P01942 | Hemoglobin subunit alpha | *Hba* | 0.5 | 1.6 |
| P11352 | Glutathione peroxidase 1 | *Gpx1* | 0.5 | 1.6 |
| P19536 | Cytochrome c oxidase subunit 5B, mitochondrial | *Cox5b* | 0.6 | 1.6 |
| P52503 | NADH dehydrogenase [ubiquinone] iron-sulfur protein 6, mitochondrial | *Ndufs6* | 0.6 | 1.6 |
| Q8CHP5 | Partner of Y14 and mago | *Wibg* | 0.8 | 1.6 |
| Q8VBW8 | Tetratricopeptide repeat protein 36 | *Ttc36* | 0.6 | 1.6 |
| P97352 | Protein S100-A13 | *S100a13* | 0.6 | 1.6 |
| Q3TGF2 | Protein FAM107B | *Fam107b* | 0.7 | 1.6 |
| P62897 | Cytochrome c, somatic | *Cycs* | 0.7 | 1.6 |
| Q6NSR8 | Probable aminopeptidase NPEPL1 | *Npepl1* | 0.7 | 1.5 |
| O35490 | Betaine--homocysteine S-methyltransferase 1 | *Bhmt* | 0.7 | 1.5 |
| P56391 | Cytochrome c oxidase subunit 6B1 | *Cox6b1* | 0.6 | 1.5 |
| Q9JL35 | High mobility group nucleosome-binding domain-containing protein 5 | *Hmgn5* | 0.6 | 1.5 |
| Q9D855 | Cytochrome b-c1 complex subunit 7 | *Uqcrb* | 0.6 | 1.5 |
| Q4VAA2-2 | Isoform A of Protein CDV3 | *OS* | 0.7 | 1.5 |
| O08585 | Clathrin light chain A | *Clta* | 0.7 | 1.5 |
| P60840 | Alpha-endosulfine | *Ensa* | 0.8 | 1.5 |
| P62075 | Mitochondrial import inner membrane translocase subunit Tim13 | *Timm13* | 0.7 | 1.5 |
| P62991 | Ubiquitin | *Rps27a* | 0.8 | 1.5 |
| O55022 | Membrane-associated progesterone receptor component 1 | *Pgrmc1* | 0.7 | 1.5 |
| Q9CY62 | E3 ubiquitin-protein ligase RNF18 | *Rnf181* | 0.7 | 1.5 |
| P70296 | Phosphatidylethanolamine-binding protein 1 | *Pebp1* | 0.8 | 1.5 |
| Q78J03 | Methionine-R-sulfoxide reductase B2, mitochondrial | *Msrb2* | 0.7 | 1.4 |
| Q9DCX2 | ATP synthase subunit d, mitochondrial | *Atp5h* | 0.8 | 1.4 |
| Q64433 | 10 kDa heat shock protein, mitochondrial | *Hspe1* | 0.7 | 1.4 |
| P70349 | Histidine triad nucleotide-binding protein 1 | *Hint1* | 0.8 | 1.4 |
| P10639 | Thioredoxin | *Txn* | 0.7 | 1.4 |
| Q9DCL8 | Protein phosphatase inhibitor 2 | *Ppp1r2* | 0.6 | 1.4 |
| Q9DB15 | 39S ribosomal protein L12, mitochondrial | *Mrpl12* | 0.7 | 1.4 |
| Q9CZL5 | Pterin-4-alpha-carbinolamine dehydratase 2 | *Pcbd2* | 0.6 | 1.4 |
| Q11136 | Xaa-Pro dipeptidase | *Pepd* | 0.8 | 1.4 |
| Q9CQE1 | Protein NipSnap homolog 3A | *Nipsnap3a* | 0.7 | 1.4 |
| P02088 | Hemoglobin subunit beta-1 | *Hbb-b1* | 0.6 | 1.4 |
| P99028 | Cytochrome b-c1 complex subunit 6, mitochondrial | *Uqcrh* | 0.7 | 1.4 |
| P48428 | Tubulin-specific chaperone A | *Tbca* | 0.6 | 1.4 |
| Q99N84 | 28S ribosomal protein S18b, mitochondrial | *Mrps18b* | 0.8 | 1.4 |
| Q8K183 | Pyridoxal kinase | *Pdxk* | 0.6 | 1.4 |
| Q8VCI5 | Peroxisomal biogenesis factor 19 | *Pex19* | 0.8 | 1.4 |
| P46414 | Cyclin-dependent kinase inhibitor 1B | *Cdkn1b* | 0.8 | 1.4 |
| P17563 | Selenium-binding protein 1 | *Selenbp1* | 0.6 | 1.3 |
| Q9D8S9 | BolA-like protein 1 | *Bola1* | 0.7 | 1.3 |
| P62073 | Mitochondrial import inner membrane translocase subunit Tim10 | *Timm10* | 0.6 | 1.3 |
| Q9CQX2 | Cytochrome b5 type B | *Cyb5b* | 0.7 | 1.3 |
| Q64213-2 | Isoform CW17E of Splicing factor 1 | *OS* | 0.8 | 1.3 |
| Q9D8Z2 | TP53-regulated inhibitor of apoptosis 1 | *Triap1* | 0.7 | 1.3 |
| Q8BFS6 | Calcineurin-like phosphoesterase domain-containing protein 1 | *Cpped1* | 0.8 | 1.3 |
| Q91WJ8 | Far upstream element-binding protein 1 | *Fubp1* | 0.8 | 1.3 |
| Q9CR98 | Protein FAM136A | *Fam136a* | 0.7 | 1.3 |
| Q9JLV1 | BAG family molecular chaperone regulator 3 | *Bag3* | 0.8 | 1.3 |
| Q9D6J6 | NADH dehydrogenase [ubiquinone] flavoprotein 2, mitochondrial | *Ndufv2* | 0.7 | 1.3 |
| Q8VCR7 | Abhydrolase domain-containing protein 14B | *Abhd14b* | 0.8 | 1.3 |
| P17182 | Alpha-enolase | *Eno1* | 0.6 | 1.2 |
| P56812 | Programmed cell death protein 5 | *Pdcd5* | 0.7 | 1.2 |
| Q60864 | Stress-induced-phosphoprotein 1 | *Stip1* | 0.8 | 1.2 |
| P05201 | Aspartate aminotransferase, cytoplasmic | *Got1* | 0.7 | 1.2 |
| P70441 | Na(+)/H(+) exchange regulatory cofactor NHE-RF1 | *Slc9a3r1* | 0.8 | 1.2 |

58 up-regulated proteins in DSSWOL as compared to DSS

|  |  |  | Fold Change | Fold Change |
| --- | --- | --- | --- | --- |
| Accession Number | Name | Gene Symbol | DSS vs. CON | DSSWOL vs. DSS |
| Q8K0E8 | Fibrinogen beta chain | *Fgb* | 1.4 | 0.8 |
| Q91X72 | Hemopexin | *Hpx* | 1.3 | 0.8 |
| P24369 | Peptidyl-prolyl cis-trans isomerase B | *Ppib* | 1.4 | 0.8 |
| P62242 | 40S ribosomal protein S8 | *Rps8* | 1.5 | 0.8 |
| Q922R8 | Protein disulfide-isomerase A6 | *Pdia6* | 1.5 | 0.8 |
| P05784 | Keratin, type I cytoskeletal 18 | *Krt18* | 1.4 | 0.7 |
| Q9D8E6 | 60S ribosomal protein L4 | *Rpl4* | 1.3 | 0.7 |
| P68369 | Tubulin alpha-1A chain | *Tuba1a* | 1.4 | 0.7 |
| P09103 | Protein disulfide-isomerase | *P4hb* | 1.6 | 0.7 |
| P14211 | Calreticulin | *Calr* | 1.5 | 0.7 |
| P68040 | Guanine nucleotide-binding protein subunit beta-2-like 1 | *Gnb2l1* | 1.2 | 0.7 |
| P11679 | Keratin, type II cytoskeletal 8 | *Krt8* | 1.6 | 0.7 |
| P01027 | Complement C3 | *C3* | 1.6 | 0.7 |
| P62908 | 40S ribosomal protein S3 | *Rps3* | 1.3 | 0.7 |
| Q8BHN3 | Neutral alpha-glucosidase AB | *Ganab* | 1.3 | 0.7 |
| P58710 | L-gulonolactone oxidase | *Gulo* | 1.2 | 0.6 |
| Q8VCM7 | Fibrinogen gamma chain | *Fgg* | 1.8 | 0.6 |
| Q63880 | Liver carboxylesterase 31 | *Es31* | 1.2 | 0.6 |
| Q8VDD5 | Myosin-9 | *Myh9* | 1.4 | 0.6 |
| P14869 | 60S acidic ribosomal protein P0 | *Rplp0* | 1.2 | 0.6 |
| P08113 | Endoplasmin | *Hsp90b1* | 1.6 | 0.6 |
| O54734 | Dolichyl-diphosphooligosaccharide--protein glycosyltransferase 48 kDa subunit | *Ddost* | 1.3 | 0.6 |
| Q80XN0 | D-beta-hydroxybutyrate dehydrogenase, mitochondrial | *Bdh1* | 1.2 | 0.6 |
| P58252 | Elongation factor 2 | *Eef2* | 1.3 | 0.6 |
| P62270 | 40S ribosomal protein S18 | *Rps18* | 1.5 | 0.6 |
| P63101 | 14-3-3 protein zeta/delta | *Ywhaz* | 1.4 | 0.6 |
| Q921I1 | Serotransferrin | *Tf* | 1.7 | 0.6 |
| Q61838 | Alpha-2-macroglobulin | *A2m* | 1.4 | 0.6 |
| P35564 | Calnexin | *Canx* | 1.3 | 0.6 |
| P48036 | Annexin A5 | *Anxa5* | 1.5 | 0.6 |
| P97872 | Dimethylaniline monooxygenase [N-oxide-forming] 5 | *Fmo5* | 1.3 | 0.6 |
| P62918 | 60S ribosomal protein L8 | *Rpl8* | 1.5 | 0.6 |
| Q91YQ5 | Dolichyl-diphosphooligosaccharide--protein glycosyltransferase subunit 1 | *Rpn1* | 1.4 | 0.6 |
| Q9Z2V4 | Phosphoenolpyruvate carboxykinase, cytosolic [GTP] | *Pck1* | 1.5 | 0.6 |
| P08003 | Protein disulfide-isomerase A4 | *Pdia4* | 1.6 | 0.6 |
| Q91X83 | S-adenosylmethionine synthase isoform type-1 | *Mat1a* | 1.5 | 0.6 |
| Q05421 | Cytochrome P450 2E1 | *Cyp2e1* | 1.2 | 0.6 |
| P06151 | L-lactate dehydrogenase A chain | *Ldha* | 1.6 | 0.6 |
| Q8CFX1 | GDH/6PGL endoplasmic bifunctional protein | *H6pd* | 1.4 | 0.6 |
| Q61335 | B-cell receptor-associated protein 31 | *Bcap31* | 1.4 | 0.6 |
| P14148 | 60S ribosomal protein L7 | *Rpl7* | 1.3 | 0.6 |
| Q9CZS1 | Aldehyde dehydrogenase X, mitochondrial | *Aldh1b1* | 1.8 | 0.6 |
| O08601 | Microsomal triglyceride transfer protein large subunit | *Mttp* | 1.2 | 0.5 |
| P54869 | Hydroxymethylglutaryl-CoA synthase, mitochondrial | *Hmgcs2* | 1.2 | 0.5 |
| Q9DBG6 | Dolichyl-diphosphooligosaccharide--protein glycosyltransferase subunit 2 | *Rpn2* | 1.4 | 0.5 |
| P25688 | Uricase | *Uox* | 1.2 | 0.5 |
| Q9D8N0 | Elongation factor 1-gamma | *Eef1g* | 1.2 | 0.5 |
| P55264 | Adenosine kinase | *Adk* | 1.2 | 0.5 |
| P53026 | 60S ribosomal protein L10a | *Rpl10a* | 1.3 | 0.5 |
| Q8CIM7 | Cytochrome P450 2D26 | *Cyp2d26* | 1.3 | 0.5 |
| P10126 | Elongation factor 1-alpha 1 | *Eef1a1* | 1.3 | 0.5 |
| P21981 | Protein-glutamine gamma-glutamyltransferase 2 | *Tgm2* | 1.6 | 0.5 |
| P00688 | Pancreatic alpha-amylase | *Amy2* | 1.9 | 0.5 |
| O35488 | Very long-chain acyl-CoA synthetase | *Slc27a2* | 1.3 | 0.5 |
| Q9DD20 | Methyltransferase-like protein 7B | *Mettl7b* | 1.4 | 0.5 |
| P62245 | 40S ribosomal protein S15a | *Rps15a* | 1.4 | 0.5 |
| Q8VCR2-2 | Isoform 2 of 17-beta-hydroxysteroid dehydrogenase 13 | *OS* | 2.1 | 0.4 |
| P62806 | Histone H4 | *Hist1h4a* | 1.4 | 0.3 |
