# Supplementary material for: Integrated omics profiling of dextran sodium sulfate-induced colitic mice supplemented with Wolfberry (Lycium barbarum)
Source: NPJ Sci Food. 2020 Mar 31;4:5. doi: 10.1038/s41538-020-0065-5 (PMC7109062; doi:10.1038/s41538-020-0065-5)
Supplement: Supplementary file 5 — Supplementary Table 4 Differentially expressed proteins in colon [file 41538_2020_65_MOESM5_ESM.docx]

Supplementary Table 4 Differentially expressed proteins in colon

271 up-regulated proteins in DSSWOL as compared to DSS

|  |  |  | Fold Change | Fold Change |
| --- | --- | --- | --- | --- |
| Accession Number | Name | Gene Symbol | DSS vs. CON | DSSWOL vs. DSS |
| O35643 | AP-1 complex subunit beta-1 | *Ap1b1* | 0.8 | 1.6 |
| P80315 | T-complex protein 1 subunit delta | *Tcpd* | 0.8 | 2.0 |
| P62196 | 26S protease regulatory subunit 8 | *Psmc5* | 0.8 | 3.7 |
| O70325 | Phospholipid hydroperoxide glutathione peroxidase, mitochondrial | *Gpx4* | 0.8 | 1.5 |
| Q6DFW4 | Nucleolar protein 58 | *Nop58* | 0.8 | 1.4 |
| Q8CHH9 | Septin-8 | *Sept8* | 0.8 | 1.3 |
| O08740 | DNA-directed RNA polymerase II subunit RPB11 | *Polr2j* | 0.8 | 1.3 |
| P45952 | Medium-chain specific acyl-CoA dehydrogenase, mitochondrial | *Acadm* | 0.8 | 1.6 |
| P55258 | Ras-related protein Rab-8A | *Rab8a* | 0.8 | 2.0 |
| Q9D4K7 | REVERSED Coiled-coil domain-containing protein 105 | *Ccdc105* | 0.8 | 1.3 |
| P56389 | Cytidine deaminase | *Cda* | 0.8 | 1.3 |
| Q99JX3 | Golgi reassembly-stacking protein 2 | *Gorasp2* | 0.7 | 1.2 |
| Q01405 | Protein transport protein Sec23A | *Sec23a* | 0.7 | 2.0 |
| P29416 | Beta-hexosaminidase subunit alpha | *Hexa* | 0.7 | 1.4 |
| Q61187 | Tumor susceptibility gene 101 protein | *Tsg101* | 0.7 | 1.3 |
| O88569 | Heterogeneous nuclear ribonucleoproteins A2/B1 | *Hnrnpa2b1* | 0.7 | 1.9 |
| P62849 | 40S ribosomal protein S24 | *Rps24* | 0.7 | 1.2 |
| Q91ZR2 | Sorting nexin-18 | *Snx18* | 0.7 | 1.2 |
| Q3TDE8 | Zinc finger protein 691 | *Znf691* | 0.7 | 1.3 |
| P09528 | Ferritin heavy chain | *Fth1* | 0.7 | 1.4 |
| Q9DCL9 | Multifunctional protein ADE2 | *Paics* | 0.7 | 1.3 |
| Q8K1I7 | WAS/WASL-interacting protein family member 1 | *Wipf1* | 0.7 | 1.5 |
| P99024 | Tubulin beta-5 chain | *Tubb5* | 0.7 | 1.2 |
| P14131 | 40S ribosomal protein S16 | *Rps16* | 0.7 | 3.5 |
| P68040 | Guanine nucleotide-binding protein subunit beta-2-like 1 | *Gnb2l1* | 0.7 | 1.5 |
| Q6P1F6 | Serine/threonine-protein phosphatase 2A 55 kDa regulatory subunit B alpha isoform | *Ppp2r2a* | 0.7 | 1.3 |
| Q9CZ44-3 | Isoform 3 of NSFL1 cofactor p47 | *Nsfl1c* | 0.7 | 1.3 |
| Q9Z2I8 | Succinyl-CoA ligase [GDP-forming] subunit beta, mitochondrial | *Suclg2* | 0.7 | 4.9 |
| Q8BG32 | 26S proteasome non-ATPase regulatory subunit 11 | *Psmd11* | 0.7 | 2.5 |
| Q9DBU0 | Transmembrane 9 superfamily member 1 | *Tm9sf1* | 0.6 | 1.7 |
| Q9CQV8 | 14-3-3 protein beta/alpha | *Ywhab* | 0.6 | 2.6 |
| Q8WTY4 | Anamorsin | *Ciapin1* | 0.6 | 1.2 |
| Q7TQG1 | Pleckstrin homology domain-containing family A member 6 | *Plekha6* | 0.6 | 1.2 |
| Q3UBX0 | Transmembrane protein 109 | *Tmem109* | 0.6 | 1.6 |
| Q8BWU5 | Probable O-sialoglycoprotein endopeptidase | *Osgep* | 0.6 | 1.4 |
| Q9CQX2 | Cytochrome b5 type B | *Cyb5b* | 0.6 | 1.3 |
| P47857 | 6-phosphofructokinase, muscle type | *Pfkm* | 0.6 | 1.4 |
| Q9DCG9 | tRNA methyltransferase 112 homolog | *Trmt112* | 0.6 | 1.7 |
| P46460 | Vesicle-fusing ATPase | *Nsf* | 0.6 | 1.5 |
| Q8R1B4 | Eukaryotic translation initiation factor 3 subunit C | *Eif3c* | 0.6 | 1.8 |
| P36993 | Protein phosphatase 1B | *Ppm1b* | 0.6 | 1.3 |
| Q8JZQ5 | Amiloride-sensitive amine oxidase [copper-containing] | *Abp1* | 0.6 | 1.3 |
| Q923D2 | Flavin reductase | *Blvrb* | 0.6 | 1.9 |
| P00329 | Alcohol dehydrogenase 1 | *Adh1* | 0.6 | 1.9 |
| Q8C878 | NEDD8-activating enzyme E1 catalytic subunit | *Uba3* | 0.6 | 1.3 |
| Q9WUZ9 | Ectonucleoside triphosphate diphosphohydrolase 5 | *Entpd5* | 0.6 | 3.9 |
| Q99L45 | Eukaryotic translation initiation factor 2 subunit 2 | *Eif2s2* | 0.6 | 1.2 |
| Q9Z2Q5 | 39S ribosomal protein L40, mitochondrial | *Mrpl40* | 0.6 | 1.4 |
| Q91X88-3 | Isoform 3 of Protein O-linked-mannose beta-1,2-N-acetylglucosaminyltransferase 1 | *Pomgnt1* | 0.6 | 1.2 |
| P61222 | ATP-binding cassette sub-family E member 1 | *Abce1* | 0.6 | 1.2 |
| P62301 | 40S ribosomal protein S13 | *Rps13* | 0.6 | 1.3 |
| Q922D8 | C-1-tetrahydrofolate synthase, cytoplasmic | *Mthfd1* | 0.6 | 1.3 |
| Q9DBG6 | Dolichyl-diphosphooligosaccharide--protein glycosyltransferase subunit 2 | *Rpn2* | 0.6 | 1.2 |
| A2AG50-2 | Isoform 2 of MAP7 domain-containing protein 2 | *Map7d2* | 0.6 | 1.2 |
| O70493 | Sorting nexin-12 | *Snx12* | 0.6 | 1.5 |
| P47963 | 60S ribosomal protein L13 | *Rpl13* | 0.6 | 1.2 |
| Q8CEK3 | Serine protease inhibitor kazal-like protein, minor form | *Spinkl* | 0.6 | 1.3 |
| Q64521 | Glycerol-3-phosphate dehydrogenase, mitochondrial | *Gpd2* | 0.6 | 1.4 |
| Q9R1T2 | SUMO-activating enzyme subunit 1 | *Sae1* | 0.6 | 2.2 |
| Q9D7G0 | Ribose-phosphate pyrophosphokinase 1 | *Prps1* | 0.6 | 1.5 |
| Q9CZC8 | Secernin-1 | *Scrn1* | 0.5 | 1.7 |
| Q9D5V6 | Synapse-associated protein 1 | *Syap1* | 0.5 | 1.3 |
| P26369 | Splicing factor U2AF 65 kDa subunit | *U2af2* | 0.5 | 1.3 |
| P63166 | Small ubiquitin-related modifier 1 | *Sumo1* | 0.5 | 1.4 |
| Q8R081 | Heterogeneous nuclear ribonucleoprotein L | *Hnrnpl* | 0.5 | 1.4 |
| P80317 | T-complex protein 1 subunit zeta | *Cct6a* | 0.5 | 2.5 |
| Q9D727 | Uncharacterized protein C6orf226 homolog |  | 0.5 | 1.5 |
| Q9CX34 | Suppressor of G2 allele of SKP1 homolog | *Sugt1* | 0.5 | 1.4 |
| P47962 | 60S ribosomal protein L5 | *Rpl5* | 0.5 | 1.8 |
| P84099 | 60S ribosomal protein L19 | *Rpl19* | 0.5 | 1.7 |
| P62806 | Histone H4 | *Hist1h4a* | 0.5 | 1.3 |
| P97825 | Hematological and neurological expressed 1 protein | *Hn1* | 0.5 | 1.6 |
| Q64520 | Guanylate kinase | *Guk1* | 0.5 | 1.3 |
| Q9CRA8 | Exosome complex exonuclease RRP46 | *Exosc5* | 0.5 | 2.2 |
| O35245 | Polycystin-2 | *Pkd2* | 0.5 | 1.2 |
| Q569Z6 | Thyroid hormone receptor-associated protein 3 | *Thrap3* | 0.5 | 1.3 |
| P62082 | 40S ribosomal protein S7 | *Rps7* | 0.5 | 2.8 |
| Q64324 | Syntaxin-binding protein 2 | *Stxbp2* | 0.5 | 1.3 |
| Q9CR41 | Huntingtin-interacting protein K | *Hypk* | 0.5 | 1.3 |
| Q80YR5 | Scaffold attachment factor B2 | *Safb2* | 0.5 | 1.3 |
| P26231 | Catenin alpha-1 | *Ctnna1* | 0.5 | 1.3 |
| Q9R1Q9 | V-type proton ATPase subunit S1 | *Atp6ap1* | 0.5 | 1.3 |
| Q9D4H8 | Cullin-2 | *Cul2* | 0.4 | 1.5 |
| Q9JKB1 | Ubiquitin carboxyl-terminal hydrolase isozyme L3 | *Uchl3* | 0.4 | 1.6 |
| P84084 | ADP-ribosylation factor 5 | *Arf5* | 0.4 | 1.6 |
| O35226 | 26S proteasome non-ATPase regulatory subunit 4 | *Psmd4* | 0.4 | 1.3 |
| P63011 | Ras-related protein Rab-3A | *Rab3a_* | 0.4 | 2.6 |
| Q6URW6-2 | Isoform 2 of Myosin-14 | *Myh14* | 0.4 | 1.3 |
| Q71RI9 | Kynurenine--oxoglutarate transaminase 3 | *Ccbl2* | 0.4 | 1.5 |
| Q8BIJ6 | Isoleucyl-tRNA synthetase, mitochondrial | *Iars2* | 0.4 | 2.7 |
| Q3UH06-2 | Isoform 2 of Ras-responsive element-binding protein 1 | *Rreb1* | 0.4 | 1.5 |
| Q61753 | D-3-phosphoglycerate dehydrogenase | *Phgdh* | 0.4 | 1.3 |
| Q62277 | Synaptophysin | *Syph* | 0.4 | 1.5 |
| P62071 | Ras-related protein R-Ras2 | *Rras2* | 0.4 | 1.5 |
| Q60668-4 | Isoform 4 of Heterogeneous nuclear ribonucleoprotein D0 | *Hnrnpd* | 0.4 | 1.4 |
| P97864 | Caspase-7 | *Casp7* | 0.4 | 1.4 |
| Q91W90 | Thioredoxin domain-containing protein 5 | *Txnd5* | 0.4 | 3.8 |
| Q8BTI8-3 | Isoform 3 of Serine/arginine repetitive matrix protein 2 | *Srrm2* | 0.4 | 1.4 |
| P35922 | Fragile X mental retardation protein 1 homolog | *Fmr1* | 0.4 | 1.3 |
| Q60739 | BAG family molecular chaperone regulator 1 | *Bag1* | 0.4 | 1.4 |
| Q00PI9 | Heterogeneous nuclear ribonucleoprotein U-like protein 2 | *Hnrnpul2* | 0.4 | 2.4 |
| Q9CZX8 | 40S ribosomal protein S19 | *Rps19* | 0.4 | 1.5 |
| P62748 | Hippocalcin-like protein 1 | *Hpcal1* | 0.4 | 1.4 |
| Q63934 | POU domain, class 4, transcription factor 2 | *Pou4f2* | 0.4 | 1.2 |
| Q9CPY7 | Cytosol aminopeptidase | *Lap3* | 0.4 | 3.0 |
| P80318 | T-complex protein 1 subunit gamma | *Cct3* | 0.4 | 3.2 |
| P43024 | Cytochrome c oxidase subunit 6A1, mitochondrial | *Cox6a1* | 0.4 | 1.3 |
| Q06185 | ATP synthase subunit e, mitochondrial | *Atp5i* | 0.4 | 1.3 |
| P19096 | Fatty acid synthase | *Fasn* | 0.3 | 1.6 |
| Q922H4 | Mannose-1-phosphate guanyltransferase alpha | *Gmppa* | 0.3 | 1.6 |
| Q8VCI5 | Peroxisomal biogenesis factor 19 | *Pex19* | 0.3 | 1.5 |
| P61979-3 | Isoform 3 of Heterogeneous nuclear ribonucleoprotein K | *Hnrnpk* | 0.3 | 1.4 |
| Q64213-3 | Isoform 3 of Splicing factor 1 | *Sf1* | 0.3 | 1.2 |
| P62754 | 40S ribosomal protein S6 | *Rps6* | 0.3 | 1.5 |
| Q9JIF7 | Coatomer subunit beta | *Copb1* | 0.3 | 1.5 |
| Q9QZ08 | N-acetyl-D-glucosamine kinase | *Nagk* | 0.3 | 1.8 |
| O70133-3 | Isoform 3 of ATP-dependent RNA helicase A | *Dhx9* | 0.3 | 1.9 |
| P29391 | Ferritin light chain 12 | *Ftl1* | 0.3 | 1.6 |
| P12023-2 | Isoform APP695 of Amyloid beta A4 protein | *App* | 0.3 | 2.9 |
| Q99MR6 | Serrate RNA effector molecule homolog | *Srrt* | 0.3 | 1.8 |
| P63276 | 40S ribosomal protein S17 | *Rps17* | 0.3 | 1.5 |
| Q68FD5 | Clathrin heavy chain 1 | *Cltc* | 0.3 | 3.7 |
| P98078-3 | Isoform p67 of Disabled homolog 2 | *Dab2* | 0.3 | 1.6 |
| P63038 | 60 kDa heat shock protein, mitochondrial | *Hspd1* | 0.3 | 3.7 |
| Q64133 | Amine oxidase [flavin-containing] A | *Maoa* | 0.3 | 1.4 |
| Q91VJ5 | REVERSED Polyglutamine-binding protein 1 | *Pqbp1* | 0.3 | 1.6 |
| Q5SYL1 | Uncharacterized serine/threonine-protein kinase SgK494 | *Sgk494* | 0.3 | 3.2 |
| Q91WJ8 | Far upstream element-binding protein 1 | *Fubp1* | 0.3 | 1.4 |
| P11352 | Glutathione peroxidase 1 | *Gpx1* | 0.3 | 1.3 |
| Q91VH2 | Sorting nexin-9 | *Snx9* | 0.3 | 1.4 |
| P62908 | 40S ribosomal protein S3 | *Fau* | 0.3 | 3.5 |
| Q9DB20 | ATP synthase subunit O, mitochondrial | *Atp5o* | 0.3 | 2.8 |
| Q9ERS2 | NADH dehydrogenase [ubiquinone] 1 alpha subcomplex subunit 13 | *Ndufa13* | 0.3 | 1.7 |
| Q8R1X6 | Spartin | *Spg20* | 0.3 | 1.4 |
| P62305 | Small nuclear ribonucleoprotein E | *Snrpe* | 0.3 | 2.1 |
| O08709 | Peroxiredoxin-6 | *Prdx6* | 0.3 | 1.5 |
| Q60598 | Src substrate cortactin | *Cttn* | 0.3 | 1.2 |
| P59325 | Eukaryotic translation initiation factor 5 | *Eif5* | 0.3 | 2.2 |
| Q8CI75 | DIS3-like exonuclease 2 | *Dis3l2* | 0.3 | 1.7 |
| O55106 | Striatin | *Strn* | 0.3 | 2.1 |
| P28653 | Biglycan | *Bgn* | 0.3 | 1.5 |
| P47911 | 60S ribosomal protein L6 | *Rpl6* | 0.2 | 7.0 |
| Q9D8Y0 | EF-hand domain-containing protein D2 | *Efhd2* | 0.2 | 2.2 |
| Q9CR68 | Cytochrome b-c1 complex subunit Rieske, mitochondrial | *Uqcrfs1* | 0.2 | 1.6 |
| O35215 | D-dopachrome decarboxylase | *Ddt* | 0.2 | 1.6 |
| Q8VEH3 | ADP-ribosylation factor-like protein 8A | *Arl8a* | 0.2 | 5.1 |
| Q3THW5 | Histone H2A.V O | *H2afv* | 0.2 | 1.5 |
| Q6PDL0 | Cytoplasmic dynein 1 light intermediate chain 2 | *Dync1li2* | 0.2 | 1.3 |
| O88735 | Ensconsin | *Map7* | 0.2 | 1.2 |
| Q05D44 | Eukaryotic translation initiation factor 5B | *Eif5b* | 0.2 | 1.7 |
| Q8C570 | mRNA export factor | *Rae1* | 0.2 | 2.2 |
| Q66JS6 | Eukaryotic translation initiation factor 3 subunit J | *Eif3j* | 0.2 | 1.3 |
| P63325 | 40S ribosomal protein S10 | *Rps10* | 0.2 | 1.9 |
| Q99K48 | Non-POU domain-containing octamer-binding protein | *Nono* | 0.2 | 2.3 |
| Q99020 | Heterogeneous nuclear ribonucleoprotein A/B | *Hnrnpab* | 0.2 | 1.3 |
| Q9Z2H7 | PDZ domain-containing protein GIPC2 | *Gipc2* | 0.2 | 1.6 |
| Q91XA2 | Golgi membrane protein 1 | *Golm1* | 0.2 | 1.8 |
| Q9CR98 | Protein FAM136A | *Fam136a* | 0.2 | 1.6 |
| Q00493 | Carboxypeptidase E | *Cpe* | 0.2 | 2.1 |
| O89017 | Legumain | *Lgmn* | 0.2 | 1.4 |
| Q8BH64 | EH domain-containing protein 2 | *Ehd2* | 0.2 | 1.3 |
| P08207 | Protein S100-A10 | *S100a10* | 0.2 | 1.4 |
| Q99PG2 | Opioid growth factor receptor | *Ogfr* | 0.2 | 1.4 |
| P62270 | 40S ribosomal protein S18 | *Rps18* | 0.2 | 3.2 |
| Q7TPV4 | Myb-binding protein 1A | *Mybbp1a* | 0.2 | 3.0 |
| Q91YN9 | BAG family molecular chaperone regulator 2 | *Bag2* | 0.2 | 1.6 |
| Q8VIJ6 | Splicing factor, proline- and glutamine-rich | *Sfpq* | 0.2 | 1.4 |
| Q9JII5 | DAZ-associated protein 1 | *Dazap1* | 0.2 | 1.8 |
| Q91WD5 | NADH dehydrogenase [ubiquinone] iron-sulfur protein 2, mitochondrial | *Ndufs2* | 0.2 | 2.1 |
| Q8BVE3 | V-type proton ATPase subunit H | *Atp6v1h* | 0.2 | 4.2 |
| Q9D8S9 | BolA-like protein 1 | *Bola1* | 0.2 | 1.2 |
| Q99MD9-2 | Isoform sNASP of Nuclear autoantigenic sperm protein | *Nasp* | 0.2 | 2.6 |
| P20065 | Thymosin beta-4 | *Tmsb4x* | 0.2 | 1.6 |
| Q63810 | Calcineurin subunit B type 1 | *Ppp3r1* | 0.2 | 1.3 |
| Q8CGN5 | Perilipin-1 | *Plin1* | 0.2 | 1.7 |
| Q8BX09 | Retinoblastoma-binding protein 5 | *Rbbp5* | 0.2 | 1.4 |
| O35658 | Complement component 1 Q subcomponent-binding protein, mitochondrial | *C1qbp* | 0.2 | 1.8 |
| P68369 | Tubulin alpha-1A chain | *Tuba1a* | 0.2 | 2.1 |
| Q62468 | Villin-1 | *Vil1* | 0.2 | 3.2 |
| P15331-3 | Isoform 5b of Peripherin | *Prph* | 0.2 | 1.5 |
| Q9WUK2 | Eukaryotic translation initiation factor 4H | *Eif4h* | 0.2 | 2.2 |
| P41105 | 60S ribosomal protein L28 | *Rpl28* | 0.2 | 1.7 |
| P63242 | Eukaryotic translation initiation factor 5A-1 | *Eif5a* | 0.2 | 1.3 |
| P04441 | H-2 class II histocompatibility antigen gamma chain | *Cd74* | 0.2 | 1.5 |
| P62242 | 40S ribosomal protein S8 | *Rps8* | 0.2 | 3.0 |
| P01592 | Immunoglobulin J chain | *Igj* | 0.2 | 1.2 |
| Q91VC7 | Protein phosphatase 1 regulatory subunit 14A | *Ppp1r14a* | 0.2 | 1.4 |
| Q80X50-2 | Isoform 2 of Ubiquitin-associated protein 2-like | *Ubap2l* | 0.2 | 2.6 |
| Q91YE8 | Synaptopodin-2 | *Synpo2* | 0.1 | 1.5 |
| Q62393-3 | Isoform 3 of Tumor protein D52 | *Tpd52* | 0.1 | 1.7 |
| Q80WW9 | DDRGK domain-containing protein 1 | *Ddrgk1* | 0.1 | 1.7 |
| P27546 | Microtubule-associated protein 4 | *Map4* | 0.1 | 1.8 |
| Q9JL35 | High mobility group nucleosome-binding domain-containing protein 5 | *Hmgn5* | 0.1 | 1.2 |
| P34022 | Ran-specific GTPase-activating protein | *Ranbp1* | 0.1 | 2.5 |
| Q8BMF4 | Dihydrolipoyllysine-residue acetyltransferase component of pyruvate dehydrogenase complex, mitochondrial | *Dlat* | 0.1 | 2.7 |
| Q80XU3 | Nuclear ubiquitous casein and cyclin-dependent kinases substrate | *Nucks1* | 0.1 | 1.9 |
| Q8R5F7-2 | Isoform 2 of Interferon-induced helicase C domain-containing protein 1 | *Ifih1* | 0.1 | 4.6 |
| Q9D7Z6 | Calcium-activated chloride channel regulator 1 | *Clca1* | 0.1 | 1.3 |
| P60879 | Synaptosomal-associated protein 25 | *Snap25* | 0.1 | 1.9 |
| P68372 | Tubulin beta-2C chain | *Tubb2c* | 0.1 | 4.2 |
| Q9ERG0 | LIM domain and actin-binding protein 1 | *Lima1* | 0.1 | 1.2 |
| Q9QYB5-2 | Isoform Short of Gamma-adducin | *Add3* | 0.1 | 1.7 |
| P62855 | 40S ribosomal protein S26 | *Rps26* | 0.1 | 3.3 |
| O70251 | Elongation factor 1-beta | *Eef1b* | 0.1 | 3.1 |
| Q91XC8 | Death-associated protein 1 | *Dap* | 0.1 | 1.5 |
| P07091 | Protein S100-A4 | *S100a4* | 0.1 | 1.7 |
| P54227 | Stathmin | *Stmn1* | 0.1 | 2.0 |
| P26350 | Prothymosin alpha | *Ptma* | 0.1 | 1.6 |
| P00405 | Cytochrome c oxidase subunit 2 | *Mtco2* | 0.1 | 1.4 |
| P57776 | Elongation factor 1-delta | *Eef1d* | 0.1 | 2.9 |
| Q8BFR5 | Elongation factor Tu, mitochondrial | *Tufm* | 0.1 | 1.5 |
| P62737 | Actin, aortic smooth muscle | *Acta2* | 0.1 | 2.0 |
| P28667 | MARCKS-related protein | *Marcksl1* | 0.1 | 1.3 |
| Q8C5W0-3 | Isoform Gamma of Calmin | *Clmn* | 0.1 | 1.4 |
| Q9D0E1-2 | Isoform 2 of Heterogeneous nuclear ribonucleoprotein M | *Hnrnpm* | 0.1 | 4.9 |
| Q80VY9 | REVERSED Putative ATP-dependent RNA helicase DHX33 | *Dhx33* | 0.1 | 1.4 |
| Q9DCV7 | Keratin, type II cytoskeletal 7 | *Krt7* | 0.1 | 2.0 |
| Q9JMG7-2 | Isoform 2 of Hepatoma-derived growth factor-related protein 3 | *Hdgfrp3* | 0.1 | 1.2 |
| P53996 | Cellular nucleic acid-binding protein | *Cnbp* | 0.1 | 1.4 |
| Q6ZQM8 | UDP-glucuronosyltransferase 1-7C | *Ugt1a7c* | 0.1 | 2.2 |
| Q9DCX2 | ATP synthase subunit d, mitochondrial | *Atp5h* | 0.1 | 2.0 |
| Q64356 | Seminal vesicle secretory protein 6 | *Svs6* | 0.1 | 2.6 |
| P52840 | Sulfotransferase 1A1 | *Sut1a1* | 0.1 | 5.8 |
| Q64475 | Histone H2B type 1-B | *Hist1h2bb* | 0.1 | 1.2 |
| P62843 | 40S ribosomal protein S15 | *Rps15* | 0.1 | 2.5 |
| P84228 | Histone H3.2 | *Hist1h3b* | 0.1 | 2.2 |
| P70349 | Histidine triad nucleotide-binding protein 1 | *Hint1* | 0.1 | 1.6 |
| P62751 | 60S ribosomal protein L23a | *Rpl23a* | 0.1 | 1.6 |
| Q80WJ7 | Protein LYRIC | *Mtdh* | 0.1 | 2.1 |
| O08583 | THO complex subunit 4 | *Thoc4* | 0.1 | 1.3 |
| O35887 | Calumenin | *Calu* | 0.1 | 1.4 |
| P47955 | 60S acidic ribosomal protein P1 | *Rplp1* | 0.1 | 1.8 |
| P55012 | Solute carrier family 12 member 2 | *Slc12a2* | 0.1 | 1.7 |
| Q05186 | Reticulocalbin-1 | *Rcn1* | 0.1 | 6.1 |
| P26339 | Chromogranin-A | *Chga* | 0.1 | 2.4 |
| Q8R1U2 | Cell growth regulator with EF hand domain protein 1 | *Cgref1* | 0.1 | 1.4 |
| P16014 | Secretogranin-1 | *Chgb* | 0.1 | 1.5 |
| P68368 | Tubulin alpha-4A chain | *Tuba4a* | 0.1 | 8.0 |
| P63037 | DnaJ homolog subfamily A member 1 | *Dnaja1* | 0.1 | 5.2 |
| Q9CR86 | Calcium-regulated heat stable protein 1 | *Carhsp1* | 0.1 | 1.8 |
| Q9D2N4-7 | Isoform 7 of Dystrobrevin alpha | *Dtna* | 0.1 | 2.3 |
| Q9CR21 | Acyl carrier protein, mitochondrial | *Ndufab1* | 0.1 | 1.2 |
| P22005 | Proenkephalin-A | *Penk* | 0.1 | 2.8 |
| Q9QXV0 | ProSAAS | *Pcsk1n* | 0.1 | 2.2 |
| Q925B0-2 | Isoform P33 of PRKC apoptosis WT1 regulator protein | *Pawr* | 0.1 | 1.4 |
| P62960 | Nuclease-sensitive element-binding protein 1 | *Ybx1* | 0.1 | 1.5 |
| Q9D8B3 | Charged multivesicular body protein 4b | *Chmp4b* | 0.1 | 1.6 |
| O08664 | B-cell CLL/lymphoma 7 protein family member C | *Bcl7c* | 0.1 | 6.8 |
| Q70IV5-2 | Isoform M of Synemin | *Synem* | 0.1 | 1.4 |
| Q6P8I4 | PEST proteolytic signal-containing nuclear protein | *Pcnp* | 0.0 | 2.6 |
| Q62395 | Trefoil factor 3 | *Tff3* | 0.0 | 11.4 |
| Q80YN3 | Breast carcinoma-amplified sequence 1 homolog | *Bcas1* | 0.0 | 2.9 |
| O54724 | Polymerase I and transcript release factor | *Ptrf* | 0.0 | 2.2 |
| P57016 | Ladinin-1 | *Lad1* | 0.0 | 1.4 |
| Q8BJW5-2 | REVERSED Isoform 2 of Nucleolar protein 11 | *Nol11* | 0.0 | 1.4 |
| P10922 | Histone H1.0 | *H1f0* | 0.0 | 2.4 |
| P97450 | ATP synthase-coupling factor 6, mitochondrial | *Atp5j* | 0.0 | 1.3 |
| P62862 | 40S ribosomal protein S30 | *Fau* | 0.0 | 1.6 |
| O54962 | Barrier-to-autointegration factor | *Banf1* | 0.0 | 1.4 |
| O88312 | Anterior gradient protein 2 homolog | *Agr2* | 0.0 | 2.4 |
| P11087 | Collagen alpha-1(I) chain | *Col1a1* | 0.0 | 1.7 |
| Q60829 | Protein phosphatase 1 regulatory subunit 1B | *Ppp1r1b* | 0.0 | 1.7 |
| O35143 | ATPase inhibitor, mitochondrial | *Atpif1* | 0.0 | 1.3 |
| Q8CGP7 | Histone H2A type 1-K | *Hist1h2ak* | 0.0 | 1.5 |
| P63254 | Cysteine-rich protein 1 | *Crip1* | 0.0 | 1.2 |
| P47915 | 60S ribosomal protein L29 | *Rpl29* | 0.0 | 1.9 |
| P15864 | Histone H1.2 | *Hist1h1c* | 0.0 | 1.4 |
| P60840 | Alpha-endosulfine | *Ensa* | 0.0 | 1.9 |
| Q62523 | Zyxin | *Zyx* | 0.0 | 1.2 |
| P43276 | Histone H1.5 | *Hist1h1b* | 0.0 | 3.2 |
| Q7TNC4 | Putative RNA-binding protein Luc7-like 2 | *Luc7l2* | 0.0 | 29.1 |

217 down-regulated proteins in DSSWOL as compared to DSS

|  |  |  | Fold Change | Fold Change |
| --- | --- | --- | --- | --- |
| Accession Number | Name | Gene Symbol | DSS vs. CON | DSSWOL vs. DSS |
| O35400 | Sulfotransferase family cytosolic 2B member 1 | *Sult2b1* | 87.9 | 0.6 |
| P07361 | Alpha-1-acid glycoprotein 2 | *Orm2* | 54.0 | 0.1 |
| Q91WS0 | CDGSH iron sulfur domain-containing protein 1 | *Cisd1* | 42.1 | 0.6 |
| Q60590 | Alpha-1-acid glycoprotein 1 | *Orm1* | 37.3 | 0.3 |
| P35230 | Regenerating islet-derived protein 3-beta | *Reg3b* | 35.0 | 0.6 |
| Q8VCT4 | Carboxylesterase 3 | *Ces3* | 31.9 | 0.8 |
| P02762 | Major urinary protein 6 | *Mup6* | 31.9 | 0.3 |
| Q00898 | Alpha-1-antitrypsin 1-5 | *Serpina1e* | 31.3 | 0.6 |
| P18242 | Cathepsin D | *Ctsd* | 29.9 | 0.8 |
| P11589 | Major urinary protein 2 | *Mup2* | 25.6 | 0.3 |
| P22599 | Alpha-1-antitrypsin 1-2 | *Serpina1b* | 25.4 | 0.8 |
| P28665 | Murinoglobulin-1 | *Mug1* | 24.4 | 0.7 |
| P07759 | Serine protease inhibitor A3K | *Serpina3k* | 24.2 | 0.6 |
| O09061 | Proteasome subunit beta type-1 | *Psmb1* | 24.2 | 0.7 |
| P29758 | Ornithine aminotransferase, mitochondrial | *Oat* | 24.0 | 0.8 |
| P04117 | Fatty acid-binding protein, adipocyte | *Fabp4* | 24.0 | 0.8 |
| P30412 | Peptidyl-prolyl cis-trans isomerase C | *Ppic* | 23.3 | 0.7 |
| Q9Z0E6 | Interferon-induced guanylate-binding protein 2 | *Gbp2* | 23.1 | 0.7 |
| Q00897 | Alpha-1-antitrypsin 1-4 | *Serpina1d* | 22.5 | 0.8 |
| Q6PFG8 | REVERSED Oligodendrocyte transcription factor 3 | *Olig3* | 22.5 | 0.8 |
| O35744 | Chitinase-3-like protein 3 | *Chi3l3* | 22.3 | 0.6 |
| P17742 | Peptidyl-prolyl cis-trans isomerase A | *Ppia* | 21.5 | 0.8 |
| P52480-2 | Isoform M1 of Pyruvate kinase isozymes M1/M2 | *Pkm2* | 20.1 | 0.7 |
| Q9JJH1 | Ribonuclease 4 | *Rnase4* | 19.8 | 0.5 |
| O70423 | Membrane primary amine oxidase | *Aoc3* | 18.7 | 0.7 |
| P02088 | Hemoglobin subunit beta-1 | *Hbb-b1* | 18.7 | 0.8 |
| P45591 | Cofilin-2 | *Cfl2* | 18.0 | 0.6 |
| Q01339 | Beta-2-glycoprotein 1 | *Apoh* | 17.7 | 0.6 |
| Q9Z315 | U4/U6.U5 tri-snRNP-associated protein 1 | *Sart1* | 17.5 | 0.7 |
| Q61704 | Inter-alpha-trypsin inhibitor heavy chain H3 | *Itih3* | 17.4 | 0.5 |
| Q8R2Y2-2 | Isoform S-gicerin of Cell surface glycoprotein MUC18 | *Mcam* | 16.6 | 0.6 |
| P12246 | Serum amyloid P-component | *Apcs* | 16.6 | 0.5 |
| Q9CPV4 | Glyoxalase domain-containing protein 4 | *Glod4* | 16.4 | 0.8 |
| Q9D1A2 | Cytosolic non-specific dipeptidase | *Cndp2* | 16.1 | 0.8 |
| P00493 | Hypoxanthine-guanine phosphoribosyltransferase | *Hprt1* | 16.1 | 0.8 |
| P97426 | Eosinophil cationic protein 1 | *Ear1* | 15.0 | 0.5 |
| O88851 | Putative hydrolase RBBP9 | *Rbbp9* | 14.7 | 0.7 |
| Q91WP6 | Serine protease inhibitor A3N | *Serpina3n* | 14.5 | 0.4 |
| Q60634 | Flotillin-2 | *Flot2* | 13.4 | 0.6 |
| P97290 | Plasma protease C1 inhibitor | *Serping1* | 12.8 | 0.6 |
| Q9DC61 | Mitochondrial-processing peptidase subunit alpha | *Pmpca* | 12.7 | 0.7 |
| P01027 | Complement C3 | *C3* | 12.4 | 0.5 |
| Q05816 | Fatty acid-binding protein, epidermal | *Fabp5* | 12.4 | 0.5 |
| Q920A5 | Retinoid-inducible serine carboxypeptidase | *Scpep1* | 12.4 | 0.8 |
| Q9R1P3 | Proteasome subunit beta type-2 | *Psmb2* | 11.9 | 0.8 |
| O08677-2 | Isoform LMW of Kininogen-1 | *Kng1* | 11.7 | 0.6 |
| Q9Z2U1 | Proteasome subunit alpha type-5 | *Psma5* | 11.4 | 0.8 |
| Q61206 | Platelet-activating factor acetylhydrolase IB subunit beta | *Pafah1b2* | 11.3 | 0.5 |
| P04186 | Complement factor B | *Cfb* | 11.0 | 0.6 |
| Q8VCA8 | Secernin-2 | *Scrn2* | 10.8 | 0.7 |
| P07356 | Annexin A2 | *Anxa2* | 10.1 | 0.7 |
| P11672 | Neutrophil gelatinase-associated lipocalin | *Lcn2* | 10.1 | 0.4 |
| Q64727 | Vinculin | *Vcl* | 9.8 | 0.7 |
| Q3U1J4 | DNA damage-binding protein 1 | *Ddb1* | 9.8 | 0.7 |
| Q8CFX1 | GDH/6PGL endoplasmic bifunctional protein | *H6pd* | 9.5 | 0.7 |
| Q91WQ3 | Tyrosyl-tRNA synthetase, cytoplasmic | *Yars* | 9.5 | 0.5 |
| Q04447 | Creatine kinase B-type | *Ckb* | 9.1 | 0.6 |
| O89020 | Afamin | *Afm* | 9.0 | 0.5 |
| P97816 | Protein S100-G | *S100g* | 8.6 | 0.7 |
| P24369 | Peptidyl-prolyl cis-trans isomerase B | *Ppib* | 8.4 | 0.6 |
| P16675 | Lysosomal protective protein | *Ctsa* | 8.4 | 0.7 |
| Q8CAY6 | Acetyl-CoA acetyltransferase, cytosolic | *Acat2* | 8.0 | 0.8 |
| P12265 | Beta-glucuronidase | *Gusb* | 8.0 | 0.7 |
| Q9JK53 | Prolargin | *Prelp* | 7.9 | 0.6 |
| P56528 | ADP-ribosyl cyclase 1 | *Cd38* | 7.9 | 0.7 |
| Q8CC86 | Nicotinate phosphoribosyltransferase | *Naprt1* | 7.9 | 0.8 |
| Q61735 | Leukocyte surface antigen CD47 | *Cd47* | 7.8 | 0.6 |
| Q91ZW2 | GDP-fucose protein O-fucosyltransferase 1 | *Pofut1* | 7.7 | 0.5 |
| Q8K0E8 | Fibrinogen beta chain | *Fgb* | 7.6 | 0.5 |
| O35639 | Annexin A3 | *Anxa3* | 7.5 | 0.6 |
| O09172 | Glutamate--cysteine ligase regulatory subunit | *Gclm* | 7.5 | 0.5 |
| P62880 | Guanine nucleotide-binding protein G(I)/G(S)/G(T) subunit beta-2 | *Gnb2* | 7.3 | 0.7 |
| Q60817 | Nascent polypeptide-associated complex subunit alpha | *Naca* | 7.2 | 0.7 |
| O88653 | Mitogen-activated protein kinase scaffold protein 1 | *Mapksp1* | 7.2 | 0.7 |
| Q8BVI4 | Dihydropteridine reductase | *Qdpr* | 7.1 | 0.8 |
| Q9QXC1 | Fetuin-B | *Fetub* | 7.1 | 0.7 |
| P05201 | Aspartate aminotransferase, cytoplasmic | *Got1* | 7.0 | 0.6 |
| Q9D1D4 | Transmembrane emp24 domain-containing protein 10 | *Tmed10* | 6.9 | 0.7 |
| P07758 | Alpha-1-antitrypsin 1-1 | *Serpina1a* | 6.8 | 0.8 |
| Q9WUM4 | Coronin-1C | *Coro1c* | 6.6 | 0.7 |
| Q61147 | Ceruloplasmin | *Cp* | 6.5 | 0.5 |
| Q61830 | Macrophage mannose receptor 1 | *Mrc1* | 6.4 | 0.7 |
| Q99L13 | 3-hydroxyisobutyrate dehydrogenase, mitochondrial | *Hibadh* | 6.4 | 0.7 |
| Q61096 | Myeloblastin | *Prtn3* | 6.4 | 0.6 |
| Q5SUR0 | Phosphoribosylformylglycinamidine synthase | *Pfas* | 6.3 | 0.7 |
| Q8R0Y6 | 10-formyltetrahydrofolate dehydrogenase | *Aldh1l1* | 6.2 | 0.6 |
| Q91YT0 | NADH dehydrogenase [ubiquinone] flavoprotein 1, mitochondrial | *Nduv1* | 6.1 | 0.6 |
| Q9DBF1 | Alpha-aminoadipic semialdehyde dehydrogenase | *Aldh7a1* | 6.1 | 0.5 |
| P97797 | Tyrosine-protein phosphatase non-receptor type substrate 1 | *Sirpa* | 6.1 | 0.6 |
| P11588 | Major urinary protein 1 | *Mup1* | 6.0 | 0.5 |
| Q9DCN2 | NADH-cytochrome b5 reductase 3 | *Cyb5r3* | 5.8 | 0.8 |
| Q00519 | Xanthine dehydrogenase/oxidase | *Xdh* | 5.7 | 0.7 |
| P11627 | Neural cell adhesion molecule L1 | *L1cam* | 5.7 | 0.6 |
| P08226 | Apolipoprotein E | *Apoe* | 5.5 | 0.5 |
| Q923B0 | AIG2-like domain-containing protein 1 | *A2ld1* | 5.4 | 0.7 |
| Q8R180 | ERO1-like protein alpha | *Ero1l* | 5.4 | 0.8 |
| O88668 | Protein CREG1 | *Creg1* | 5.3 | 0.8 |
| P29699 | Alpha-2-HS-glycoprotein | *Ahsg* | 5.2 | 0.6 |
| P11438 | Lysosome-associated membrane glycoprotein 1 | *Lamp1* | 5.0 | 0.7 |
| P31725 | Protein S100-A9 | *S100a9* | 4.9 | 0.6 |
| Q9CQ65 | S-methyl-5'-thioadenosine phosphorylase | *Mtap* | 4.6 | 0.8 |
| Q3UM45 | Protein phosphatase 1 regulatory subunit 7 | *Ppp1r7* | 4.6 | 0.7 |
| Q07456 | Protein AMBP | *Ambp* | 4.6 | 0.5 |
| Q9CYR6 | Phosphoacetylglucosamine mutase | *Pgm3* | 4.5 | 0.8 |
| P61082 | NEDD8-conjugating enzyme Ubc12 | *Ube2m* | 4.5 | 0.7 |
| Q3UE37 | Ubiquitin-conjugating enzyme E2 Z | *Ube2z* | 4.5 | 0.8 |
| Q9CQZ1 | Heat shock factor-binding protein 1 | *Hsbp1* | 4.4 | 0.6 |
| Q8BZF8 | Phosphoglucomutase-like protein 5 | *Pgm5* | 4.4 | 0.8 |
| Q99JI6 | Ras-related protein Rap-1b | *Rap1b* | 4.1 | 0.6 |
| Q8VCM7 | Fibrinogen gamma chain | *Fgg* | 4.1 | 0.6 |
| Q9JHS3 | Mitogen-activated protein-binding protein-interacting protein | *Robld3* | 4.1 | 0.8 |
| P19221 | Prothrombin | *F2* | 3.9 | 0.7 |
| P70362 | Ubiquitin fusion degradation protein 1 homolog | *Ufd1l* | 3.9 | 0.8 |
| P37040 | NADPH--cytochrome P450 reductase | *Por* | 3.9 | 0.8 |
| P25085 | Interleukin-1 receptor antagonist protein | *Il1rn* | 3.7 | 0.8 |
| P15379-9 | Isoform 5 of CD44 antigen | *Cd44* | 3.7 | 0.7 |
| Q8CHP8 | Phosphoglycolate phosphatase | *Pgp* | 3.6 | 0.7 |
| Q3V3R4 | Integrin alpha-1 | *Itga1* | 3.6 | 0.5 |
| Q9JK38 | Glucosamine 6-phosphate N-acetyltransferase | *Gnpnat1* | 3.5 | 0.8 |
| P49722 | Proteasome subunit alpha type-2 | *Psma2* | 3.4 | 0.6 |
| Q99KB8 | Hydroxyacylglutathione hydrolase, mitochondrial | *Hagh* | 3.4 | 0.5 |
| Q8C0M9 | L-asparaginase | *Asrgl1* | 3.4 | 0.5 |
| P20108 | Thioredoxin-dependent peroxide reductase, mitochondrial | *Prdx3* | 3.3 | 0.8 |
| P10518 | Delta-aminolevulinic acid dehydratase | *Alad* | 3.3 | 0.8 |
| P26041 | Moesin | *Msn* | 3.2 | 0.7 |
| Q6P1B1 | Xaa-Pro aminopeptidase 1 | *Xpnpep1* | 3.2 | 0.7 |
| Q9QZ85 | Interferon-inducible GTPase 1 | *Iigp1* | 3.2 | 0.6 |
| Q9DAS9 | Guanine nucleotide-binding protein G(I)/G(S)/G(O) subunit gamma-12 | *Gng12* | 3.2 | 0.3 |
| P08071 | Lactotransferrin | *Ltf* | 3.2 | 0.7 |
| P01942 | Hemoglobin subunit alpha | *Hba* | 3.1 | 0.3 |
| P09055 | Integrin beta-1 | *Itgb1* | 3.1 | 0.7 |
| Q9ERF3 | WD repeat-containing protein 61 | *Wdr61* | 3.0 | 0.6 |
| P13595-2 | Isoform N-CAM 140 of Neural cell adhesion molecule 1 | *Pimt* | 3.0 | 0.7 |
| P23506 | Protein-L-isoaspartate(D-aspartate) O-methyltransferase | *Pcmt1* | 3.0 | 0.5 |
| P51162 | Gastrotropin | *Fabp6* | 3.0 | 0.8 |
| Q9JJU8 | SH3 domain-binding glutamic acid-rich-like protein | *Sh3bgrl* | 2.9 | 0.5 |
| Q8R0F8 | Fumarylacetoacetate hydrolase domain-containing protein 1 | *Fahd1* | 2.8 | 0.8 |
| O09117 | Synaptophysin-like protein 1 | *Sypl1* | 2.8 | 0.6 |
| O88958 | Glucosamine-6-phosphate isomerase 1 | *Gnpda1* | 2.7 | 0.7 |
| Q8K2I4 | Beta-mannosidase | *Manba* | 2.7 | 0.7 |
| P13745 | Glutathione S-transferase A1 | *Gsta1* | 2.7 | 0.7 |
| Q01065 | Calcium/calmodulin-dependent 3',5'-cyclic nucleotide phosphodiesterase 1B | *Pde1b* | 2.6 | 0.8 |
| Q9D8S4 | Oligoribonuclease, mitochondrial | *Rexo2* | 2.6 | 0.6 |
| Q9CQV6 | Microtubule-associated proteins 1A/1B light chain 3B | *Map1lc3b* | 2.5 | 0.5 |
| Q9Z2A9 | Gamma-glutamyltransferase 5 | *Ggt5* | 2.5 | 0.7 |
| P16045 | Galectin-1 | *Lgals1* | 2.3 | 0.5 |
| Q9CQJ8 | NADH dehydrogenase [ubiquinone] 1 beta subcomplex subunit 9 | *Ndufb9* | 2.3 | 0.2 |
| P08121 | Collagen alpha-1(III) chain | *Col3a1* | 2.2 | 0.6 |
| Q8C129 | Leucyl-cystinyl aminopeptidase | *Lnpep* | 2.2 | 0.8 |
| O08917 | Flotillin-1 | *Flot1* | 2.1 | 0.6 |
| O09164 | Extracellular superoxide dismutase [Cu-Zn] | *Sod3* | 2.1 | 0.5 |
| B2RRE7 | OTU domain-containing protein 4 | *Otud4* | 2.1 | 0.8 |
| P82198 | Transforming growth factor-beta-induced protein ig-h3 | *Tgfbi* | 2.1 | 0.7 |
| P51660 | Peroxisomal multifunctional enzyme type 2 | *Hsd17b4* | 2.0 | 0.7 |
| P56677 | Suppressor of tumorigenicity 14 protein homolog | *St14* | 2.0 | 0.8 |
| Q9R069 | Basal cell adhesion molecule | *Bcam* | 2.0 | 0.6 |
| Q8VE62 | Polyadenylate-binding protein-interacting protein 1 | *Paip1* | 2.0 | 0.7 |
| Q9D964 | Glycine amidinotransferase, mitochondrial | *Gatm* | 1.9 | 0.6 |
| Q9CQF9 | Prenylcysteine oxidase | *Pcyox1* | 1.9 | 0.8 |
| Q6P2B1 | REVERSED Transportin-3 | *Tnpo3* | 1.9 | 0.7 |
| P61087 | Ubiquitin-conjugating enzyme E2 K | *Ube2k* | 1.9 | 0.8 |
| O35114 | Lysosome membrane protein 2 | *Scarb2* | 1.9 | 0.7 |
| Q62426 | Cystatin-B | *Cstb* | 1.8 | 0.7 |
| P28076 | Proteasome subunit beta type-9 | *Psmb9* | 1.8 | 0.6 |
| Q61543 | Golgi apparatus protein 1 | *Glg1* | 1.8 | 0.8 |
| P61922 | 4-aminobutyrate aminotransferase, mitochondrial | *Abat* | 1.8 | 0.7 |
| Q9CXN7 | Phenazine biosynthesis-like domain-containing protein 2 | *Pbld2* | 1.8 | 0.7 |
| P32261 | Antithrombin-III | *Serpinc1* | 1.8 | 0.5 |
| Q62000 | Mimecan | *Ogn* | 1.8 | 0.7 |
| Q61166 | Microtubule-associated protein RP/EB family member 1 | *Mapre1* | 1.7 | 0.7 |
| Q9CQ62 | 2,4-dienoyl-CoA reductase, mitochondrial | *Decr1* | 1.7 | 0.8 |
| P97300 | Neuroplastin | *Nptn* | 1.7 | 0.5 |
| Q9CZM2 | 60S ribosomal protein L15 | *Rpl15* | 1.7 | 0.7 |
| Q9D7I5 | Phospholysine phosphohistidine inorganic pyrophosphate phosphatase | *Lhpp* | 1.7 | 0.7 |
| Q8C4M7 | Centromere protein U | *Mlf1ip* | 1.7 | 0.6 |
| P62774 | Myotrophin | *Mtpn* | 1.6 | 0.7 |
| P00688 | Pancreatic alpha-amylase | *Amy2* | 1.6 | 0.7 |
| O88693 | Ceramide glucosyltransferase | *Ugcg* | 1.6 | 0.2 |
| Q14BI7-3 | REVERSED Isoform 3 of Putative ATP-dependent RNA helicase TDRD9 | *Tdrd9* | 1.6 | 0.3 |
| P01837 | Ig kappa chain C region | *Igk* | 1.6 | 0.5 |
| Q8K3X4 | Enhanced at puberty protein 1 | *Eap1* | 1.6 | 0.7 |
| P19973 | Lymphocyte-specific protein 1 | *Lsp1* | 1.5 | 0.7 |
| Q78JE5 | F-box only protein 22 | *Fbxo22* | 1.5 | 0.7 |
| Q60870 | Receptor expression-enhancing protein 5 | *Reep5* | 1.5 | 0.5 |
| P63168 | Dynein light chain 1, cytoplasmic | *Dynll1* | 1.5 | 0.6 |
| Q9DBS1 | Transmembrane protein 43 | *Tmem43* | 1.5 | 0.7 |
| Q61001 | Laminin subunit alpha-5 | *Lama5* | 1.5 | 0.3 |
| O54786 | DNA fragmentation factor subunit alpha | *Dffa* | 1.4 | 0.6 |
| P03953 | Complement factor D | *Cfd* | 1.4 | 0.5 |
| Q9DCS2 | UPF0585 protein C16orf13 homolog |  | 1.4 | 0.6 |
| O88874 | Cyclin-K | *Ccnk* | 1.4 | 0.6 |
| P62311 | U6 snRNA-associated Sm-like protein LSm3 | *Lsm3* | 1.4 | 0.5 |
| Q8K411 | Presequence protease, mitochondrial | *Pitrm1* | 1.4 | 0.8 |
| Q7TNG5 | Echinoderm microtubule-associated protein-like 2 | *Eml2* | 1.4 | 0.4 |
| Q64735 | Complement regulatory protein Crry | *Crry* | 1.4 | 0.3 |
| Q8K4G1 | Latent-transforming growth factor beta-binding protein 4 | *Ltbp4* | 1.4 | 0.7 |
| P97379 | Ras GTPase-activating protein-binding protein 2 | *G3bp2* | 1.3 | 0.6 |
| Q9CQM5 | Thioredoxin domain-containing protein 17 | *Txndc17* | 1.3 | 0.6 |
| Q9CWK8 | Sorting nexin-2 | *Snx2* | 1.3 | 0.7 |
| P67871 | Casein kinase II subunit beta | *Csnk2b* | 1.3 | 0.5 |
| P62991 | Ubiquitin | *Rps27a* | 1.3 | 0.4 |
| Q9WVB0 | RNA-binding protein with multiple splicing | *Rbpms* | 1.3 | 0.3 |
| Q8BHC0 | Lymphatic vessel endothelial hyaluronic acid receptor 1 | *Lyve1* | 1.3 | 0.8 |
| P47212 | Galanin | *Gal* | 1.3 | 0.7 |
| P14483 | H-2 class II histocompatibility antigen, A beta chain | *H2-Ab1* | 1.3 | 0.6 |
| Q8BH58 | TIP41-like protein | *Tiprl* | 1.3 | 0.6 |
| P97927 | Laminin subunit alpha-4 | *Lama4* | 1.3 | 0.5 |
| Q9CQA3 | Succinate dehydrogenase [ubiquinone] iron-sulfur subunit, mitochondrial | *Sdhb* | 1.2 | 0.7 |
| Q99LS3 | Phosphoserine phosphatase | *Psph* | 1.2 | 0.7 |
| Q8CI85 | Carbonic anhydrase 12 | *Ca12* | 1.2 | 0.7 |
| Q60597-2 | Isoform 2 of 2-oxoglutarate dehydrogenase, mitochondrial | *Ogdh* | 1.2 | 0.8 |
| Q8K2B3 | Succinate dehydrogenase [ubiquinone] flavoprotein subunit, mitochondrial | *Sdha* | 1.2 | 0.8 |
| O35309 | N-myc-interactor | *Nmi* | 1.2 | 0.7 |
| O09049 | Regenerating islet-derived protein 3-gamma | *Reg3g* | 1.2 | 0.8 |
| Q9D952 | REVERSED Envoplakin | *Evpl* | 1.2 | 0.5 |
| Q9EPU0 | Regulator of nonsense transcripts 1 | *Upf1* | 1.2 | 0.4 |
| Q9R1Q7 | Proteolipid protein 2 | *Plp2* | 1.2 | 0.5 |
