# Supplementary material for: Integrated omics profiling of dextran sodium sulfate-induced colitic mice supplemented with Wolfberry (Lycium barbarum)
Source: NPJ Sci Food. 2020 Mar 31;4:5. doi: 10.1038/s41538-020-0065-5 (PMC7109062; doi:10.1038/s41538-020-0065-5)
Supplement: Supplementary file 6 — Supplementary Table 5 Metabolome [file 41538_2020_65_MOESM6_ESM.docx]

Supplementary Table 5a 10 differentially expressed metabolites in liver

|  | DSS vs. CON | | DSSWOL vs. DSS | |
| --- | --- | --- | --- | --- |
| Metabolite | Fold Change | p-value | Fold Change | p-value |
| beta-Ala-Lys | 1.3 | 0.05 | 1.3 | 0.01 |
| Glutathione(red) | 0.8 | 0.03 | 1.4 | 0.00 |
| Hydroxyproline | 0.7 | 0.01 | 1.3 | 0.04 |
| S-Lactoylglutathione | 0.8 | 0.02 | 1.3 | 0.00 |
| F6P | 0.6 | 0.00 | 6.4 | 0.01 |
| G6P | 0.5 | 0.00 | 2.2 | 0.01 |
| AMP | 0.7 | 0.00 | 1.3 | 0.02 |
| IMP | 0.7 | 0.04 | 2.0 | 0.00 |
| GMP | 0.8 | 0.01 | 1.2 | 0.04 |
| UDP-N-acetylglucosamine | 1.4 | 0.00 | 1.2 | 0.02 |

Supplementary Table 5b 4 differentially expressed metabolites in plasma

|  | DSS vs. CON | | DSSWOL vs. DSS | |
| --- | --- | --- | --- | --- |
| Metabolite | Fold Change | p-value | Fold Change | p-value |
| 2-Hydroxyisobutyrate | 0.4 | 0.01 | 3.1 | 0.06 |
| Dodecanoate | 0.2 | 0.02 | 1.1 | 0.07 |
| Arg | 1.1 | 0.02 | 0.8 | 0.06 |
| Creatine | 1.6 | 0.05 | 0.8 | 0.01 |
