# Supplementary material for: Integrated omics profiling of dextran sodium sulfate-induced colitic mice supplemented with Wolfberry (Lycium barbarum)
Source: NPJ Sci Food. 2020 Mar 31;4:5. doi: 10.1038/s41538-020-0065-5 (PMC7109062; doi:10.1038/s41538-020-0065-5)
Supplement: Supplementary file 7 — Supplementary Table 6 PCR Primer Sequences [file 41538_2020_65_MOESM7_ESM.docx]

|  | Control diet /kg | 2% Wolfberry powder supplemented diet / kg |
| --- | --- | --- |
| Cornstarch | 397.5 | 377.5 |
| Casein | 200 | 200 |
| Detrinized cornstarch | 132 | 132 |
| Sucrose | 102.5 | 102.5 |
| Soybean oil (no additives) | 70 | 70 |
| Fiber | 50 | 50 |
| Mineral mix (AIN 93G-MX) | 35 | 35 |
| Vitamin mix (AIN 93G-MX) | 10 | 10 |
| L-Cystine | 3 | 3 |
| Ground whole wolfberry | 0 | 20 |
|  |  |  |

Supplementary Table 6a Nutritional composition of diets

Supplementary Table 6b Oligonucleotide sequences used in real-time PCR analysis

| Gene Symbol | Full name |  | Oligonucleotide sequences |
| --- | --- | --- | --- |
| *Cd163* | cluster of Differentiation 163 | F | GGCAACAAATACGTGGCTCT |
|  |  | R | CACACGTCTCCGTGTTTCAC |
| *Elovl6* | elongation of very long chain fatty acid 6 | F | AGAGCCGGATTCATCCAATGTGCAG |
|  |  | R | CAATGTGCAGACAGCGTGTTCGCCT |
| *Fas* | fatty acid synthase | F | GTTCCCAGAGCCACTGACTTGGAGA |
|  |  | R | GACTTGGAGACACCCTGGTCTGTGA |
| *Hp* | haptoglobin | F | GCTGTTGTCACTCTCCTGCTCT |
|  |  | R | AAGTGCTCCACATAGCCGTTT |
| *Il6* | interleukin 6 | F | GCCAGAGTCCTTCAGAGAGATACA |
|  |  | R | CTTGGTCCTTAGCCACTCCTTC |
| *Jun* | Jun oncogene | F | CAACCTCTTTGCTGCATTATCCATA |
|  |  | R | TAGGGTGAAAGCTGTGTCCCCTGTC |
| *Mat2a* | methionine adenosyltransferase II, alpha | F | GTGTGGTACAGAGAACCCAGCTTGT |
|  |  | R | CAGAGAACCCAGCTTGTTTACATGC |
| *Me1* | malic enzyme 1, NADP(+)-dependent, cytosolic | F | GATATGGCTGCCTTCAACGAGCGGC |
|  |  | R | CAGAGCAGTGCTACAAGGTGACCAA |
| *Mmp3* | matrix metallopeptidase 3 | F | CCACCCTTGAGTCAACACCT |
|  |  | R | CCAGGACGGTGACACACATA |
| *Mmp10* | matrix metallopeptidase 10 | F | GCTCAGGTGATGCTTTGTGA |
|  |  | R | ATGTTACTGTGGGCTGTGG |
| *Mmp13* | matrix metallopeptidase 13 | F | CCTGGAATTGGCAACAAAGT |
|  |  | R | CCCACCCCATACATCTGAAA |
| *Ppia* | peptidylprolyl isomerase A | F | GCTGGACCAAACACAAACG |
|  |  | R | ATGCCTTCTTTCACCTTCCC |
| *Rplp1* | 60S acidic ribosomal protein P1 | F | TCATTCTCCACGACGACGA |
|  |  | R | CCCATGTCATCATCAGATTCC |
| *Saa1* | serum amyloid A1 | F | GCGAGCCTACACTGACATGA |
|  |  | R | GGCAGTCCAGGAGGTCTGTA |
| *Scd1* | stearoyl-Coenzyme A desaturase 1 | F | TCTTGCGATACACTCTGGTGCTCAA |
|  |  | R | AGTGCCGCGCATCTCTATGGATATC |
| *S100A8* | S100 calcium binding protein A8 (calgranulin A) | F | TACCTCATTTGTTGTGTGACTGAGT |
|  |  | R | ACTACTGTTGTGTCTAAAAAGTCGG |
| *Timp1* | tissue inhibitor of metalloproteinase 1 | F | CGACAAAAGCAATTCCAAGGG |
|  |  | R | TACCAATGTTACTGTGGGCTGG |
